# Identifying indicators sensitive to primary healthcare nurse practitioner practice: A review of systematic reviews

**DOI:** 10.1371/journal.pone.0290977

**Published:** 2023-09-07

**Authors:** Kelley Kilpatrick, Eric Tchouaket, Isabelle Savard, Maud-Christine Chouinard, Naima Bouabdillah, Bruno Provost-Bazinet, Gina Costanzo, Julie Houle, Geneviève St-Louis, Mira Jabbour, Renée Atallah

**Affiliations:** 1 Susan E. French Chair in Nursing Research and Innovative Practice, Ingram School of Nursing, Faculty of Medicine and Health Sciences, McGill University, Montreal, Quebec, Canada; 2 Centre Intégré Universitaire de Santé et de Services Sociaux de l’Est-de-l’Île-de-Montréal (CIUSSS-EMTL), Maisonneuve-Rosemont Hospital Site, Montréal, Québec, Canada; 3 Department of Nursing, Université du Québec en Outaouais (UQO), St-Jérôme Campus, Saint-Jérôme, Québec, Canada; 4 Ingram School of Nursing, Faculty of Medicine and Health Sciences, McGill University, Montréal, Québec, Canada; 5 Faculté des Sciences Infirmières, Université de Montréal, Montréal, Québec, Canada; 6 Centre Intégré Universitaire de Santé et de Services Sociaux du Nord-de-l’Île-de-Montréal (CIUSSS-NIM), Montréal, Québec, Canada; 7 Department of Nursing, Université du Québec à Trois-Rivières, Trois-Rivières, Québec, Canada; 8 Centre Intégré Universitaire de Santé et de Services Sociaux de la Mauricie-et-du-Centre-du-Québec (CIUSSS-MCQ), Trois-Rivières, Québec, Canada; 9 Support and Development of Professional Practices in Nursing and Assistance Care and Infection Prevention Associate Directorate, Centre Intégré Universitaire de Santé et de Services Sociaux de la Mauricie-et-du-Centre-du-Québec (CIUSSS-MCQ), Trois-Rivières, Québec, Canada; FMUP: Universidade do Porto Faculdade de Medicina, PORTUGAL

## Abstract

**Aim:**

To identify indicators sensitive to the practice of primary healthcare nurse practitioners (PHCNPs).

**Materials and methods:**

A review of systematic reviews was undertaken to identify indicators sensitive to PHCNP practice. Published and grey literature was searched from January 1, 2010 to December 2, 2022. Titles/abstracts (n = 4251) and full texts (n = 365) were screened independently by two reviewers, with a third acting as a tie-breaker. Reference lists of relevant publications were reviewed. Risk of bias was examined independently by two reviewers using AMSTAR-2. Data were extracted by one reviewer and verified by a second reviewer to describe study characteristics, indicators, and results. Indicators were recoded into categories. Findings were summarized using narrative synthesis.

**Results:**

Forty-four systematic reviews were retained including 271 indicators that were recoded into 26 indicator categories at the patient, provider and health system levels. Nineteen reviews were assessed to be at low risk of bias. *Patient indicator categories* included activities of daily living, adaptation to health conditions, clinical conditions, diagnosis, education-patient, mortality, patient adherence, quality of life, satisfaction, and signs and symptoms. *Provider indicator categories* included adherence to best practice-providers, education-providers, illness prevention, interprofessional team functioning, and prescribing. *Health system indicator categories* included access to care, consultations, costs, emergency room visits, healthcare service delivery, hospitalizations, length of stay, patient safety, quality of care, scope of practice, and wait times.

**Discussion:**

Equal to improved care for almost all indicators was found consistently for the PHCNP group. Very few indicators favoured the control group. No indicator was identified for high/low fidelity simulation, cultural safety and cultural sensitivity with people in vulnerable situations or Indigenous Peoples.

**Conclusion:**

This review of systematic reviews identified patient, provider and health system indicators sensitive to PHCNP practice. The findings help clarify how PHCNPs contribute to care outcomes.

**PROSPERO registration number:**

CRD42020198182.

## Introduction

The delivery of safe, efficient and effective primary healthcare (PHC) is a global imperative [1, 2]. System level characteristics including accessibility, comprehensiveness, coordination and continuity of care, equity, service integration, and patient-centeredness are key considerations when providing PHC [2–4]. Globally, the COVID-19 pandemic has highlighted that workforce data to measure the contributions of providers who are not physicians are desperately needed to adequately respond to unmet patient care needs [5]. Internationally, primary healthcare nurse practitioners (PHCNPs) have been introduced to improve access to care [6]. PHCNPs are nurses prepared at the graduate level with in-depth clinical expertise who practice in a wide range of healthcare settings (e.g., primary care, home care, long-term care), and provide PHC services to different populations [6–9].

A review of systematic reviews of PHC quality indicators by Ramalho et al. [4] identified 727 quality indicators where almost 75% of the indicators focused on processes of care (e.g., treatment). Several systematic reviews have been completed (e.g., Barker et al., 2018; Martin-Misener et al., 2015; Swan et al., 2015) [10–12] to understand how PHCNPs contribute to patient care. These reviews highlighted that PHCNPs provide care that is equal to or superior to the comparator group, often physicians. Different trends were noted for appointment times with longer appointments for PHCNPs favouring the physician group. In addition, systematic reviews reported few results documenting the impact of mental health services provided by PHCNPs. As early as 2009, Laurant and colleagues noted that an “exact description of the nurses’ roles was lacking in the majority of reviews” [p. 44S] [13] following their review of systematic reviews (n = 18 studies) of the effectiveness of nonphysician clinicians that included PHCNPs and other nursing roles. In 2014, Kilpatrick et al. [14] conducted an umbrella review to examine the impact of graduate-prepared nurses in advanced practice roles that included nurse practitioners (NPs) and clinical nurse specialists. This umbrella review identified four systematic reviews examining patient, provider and healthcare system outcomes in acute and primary care settings.

With the expansion of PHCNP roles into new areas including home care and long-term care to support the delivery of patient-centered care [15], an important gap remains in identifying indicators that have been used to document outcomes of PHCNP practice using recognized role definitions [6]. Thus, to understand how PHCNPs contribute to care and synthesize the available evidence, we conducted a review of systematic reviews of studies that incorporated recognized PHCNP role definitions. Our research question was: What indicators are sensitive to PHCNP practice from the patient, provider and health system perspectives? More specifically, our aims were: 1) To assess the quality of systematic reviews of the impact of PHCNP practice from the patient, provider and health system perspectives; and 2) To identify indicators sensitive to PHCNP practice from the patient, provider and health system perspectives.

## Materials and methods

To identify indicators sensitive to PHCNP practice, we conducted a review of systematic reviews according to the Preferred Reporting Items for Systematic review and Meta-Analysis (PRISMA) statement [16]. Also registered in PROSPERO (#CRD42020198182), the published review protocol was developed a priori and included keywords and examples of search strategies [17, 18].

### Inclusion and exclusion criteria

All relevant published and unpublished systematic reviews reported from January 1, 2010 up to December 2, 2022, with no restrictions on jurisdiction or language, were included. To be retained, authors needed to provide sufficient information so the reviewer could identify all the components of a research question (i.e., PICOS), inclusion and exclusion criteria, and use methods to identify relevant published and unpublished evidence to reduce the risk of bias [16]. We excluded reviews if the PHCNP impact was not reported separately from other types of nurses or team members or if no indicators could be identified. We also excluded reviews that addressed broad research questions (e.g., scoping reviews). Nurse midwives were excluded because these roles are not consistently identified as advanced practice nursing roles internationally, and regulatory bodies in different countries do not all require these roles to be filled by nurses.

We considered reviews that included all types of study designs to capture the impact of a complex intervention like the addition of PHCNPs in healthcare teams because different types of information are needed to inform decisions about role effectiveness [19]. Participants included patients of any age, groups or communities receiving PHC care in all types (e.g., teaching and non-teaching), sizes (e.g., small, large) and locations (e.g., rural, urban) of community or care agencies (e.g., primary care, home care, long-term care).

#### Interventions

We included reviews that examined the care provided by PHCNPs in different sectors. We adopted the International Council of Nurses definition of PHCNP roles regardless of the role title [6]. We focussed on registered nurses, educated at the Master’s level or above who have an in-depth clinical expertise, who possess and demonstrate the competencies to autonomously diagnose, order and interpret diagnostic tests, prescribe pharmaceuticals and perform specific procedures within their legislated scope of practice [6]. More specifically, for nurse-led services we carefully reviewed role descriptions to ascertain the level of decision-making autonomy of the nurses.

#### Controls

The comparator (i.e., control) group described the group to which NPs were compared. Comparators included usual care, care provided by another healthcare provider (e.g., physicians), best care, and no comparator.

#### Types of outcomes

We included any outcome indicator that measured the effectiveness of PHCNP roles. We extracted data related to effect sizes, including the actual effect size (i.e., odds ratio, relative risk, mean difference), confidence intervals, level of statistical significance, and the number of studies included in the analysis.

### Search strategy

We limited the search from January 1, 2010 up to December 2, 2022 to capture the most up-to-date trends. Pieper et al. [20] argue that evidence in about half of published reviews is outdated after five years. Small changes were made in the databases as proposed in the protocol as some were no longer available at the time data were collected [18]. The following electronic databases were searched: Cumulative Index to Nursing and Allied Health Literature (CINAHL), Cochrane Library Database of Systematic Reviews and Controlled Trials Register, Database of Abstracts of Reviews of Effects (DARE), Embase, Global Health, Joanna Briggs Institute (JBI) EBP, Ovid Healthstar, Ovid MEDLINE, PubMed, and Web of Science Core Collection. The search strategy combined subject headings and keywords related to primary healthcare, advanced practice nursing, and different types of outcomes. They were subsequently combined with a search filter we derived from the Canadian Agency for Drugs and Technologies in Health (CADTH)’s systematic reviews and meta-analyses search filter [21] and that designed by Lunny et al. for reviews of systematic reviews [22]. The search strategy was adapted for each database, and reviewed by an academic librarian (See S1 Appendix). In addition, the reference lists of relevant reviews were hand-searched.

The grey literature was searched for the same period, and included: CADTH Information Services, relevant websites from CADTH’s Grey Matters tool, Health Evidence, Heath Systems Evidence, OpenGrey Repository, Organization for Economic Co-operation and Development (OECD), PDQ-Evidence, ProQuest Dissertation and Theses, and World Health Organization. The PROSPERO International Prospective Register of Systematic Reviews was searched to identify registered review protocols. For each website, the content was searched using the same search terms (e.g., indicator AND (primary care) AND (nurse practitioner)) as those used for the published literature. If there was not an inherent search function on the website, a search was conducted of all webpages and weblinks. The search strategy for the unpublished literature is included in S2 Appendix.

### Study selection

All reviewers (eight in total, grouped into four pairs) were trained to use the screening instrument and the inclusion/exclusion criteria. Regular follow-up sessions were held to answer any questions. The retained studies were uploaded into the EndNote software and the RAYYAN web platform [23]. Duplicates were removed. Two reviewers independently screened titles and abstracts using the prespecified inclusion/exclusion criteria. Discrepancies were discussed among the reviewers. A third reviewer acted as a tie-breaker if the first reviewers could not come to a consensus. If no abstract was available, a full text review was completed. A full text review was completed for all the reviews included after the initial screening. The reviewers reached an agreement level of 84.3% using the abovementioned inclusion/exclusion criteria, corresponding to a fair inter-rater agreement (Cohen’s kappa: 0.394) [24]. Exclusions related to “wrong role” included systematic reviews with no PHCNP role or with a PHCNP role not meeting our pre-specified definition, focusing on the wrong patient population/setting (i.e., not PHC), or whose topic was irrelevant to answer our research question. The most important challenge for reviewers was to identify consistent PHCNP role definitions.

### Data extraction

For the included papers, all data were extracted by one coder and reviewed by a second coder. A structured tool was developed for the study and pilot-tested by the investigators [25]. The extracted data included the aim of the review; review characteristics (e.g., year); number of databases searched; countries; number of studies, date range and designs; population characteristics, intervention, comparator, and synthesis method. Specifications of patient, provider and health system outcomes, how the outcomes were measured, number of patients and providers in the intervention and control groups, effect sizes, p values, and funding source were recorded. The results of the meta-analyses, if conducted, were included. If the data were not available in the review, extractors indicated ‘not reported’ in the data extraction form.

### Risk of bias assessment

Each review’s methodological quality was assessed using AMSTAR 2 criteria [26]. Training sessions were conducted with the reviewers who conducted the assessments to enhance rater understanding of the items. The reviewers independently rated each review, and inter-rater agreement was estimated using the kappa statistic [24, 26]. Any disagreements were discussed among the reviewers until consensus was reached. A summary table with the AMSTAR 2 ratings can be found in Table 1. Inter-rater agreement was 91.1% and substantial for all the items using Cohen’s kappa (κ) (κ = 0,791). Estimates using Cohen’s kappa was assessed as fair to complete agreement with κ values ranging from 0.377 to 0.952 and perfect agreement. Disagreements between reviewers were noted for some elements of the AMSTAR 2 assessment tool (see Discussion). Nineteen studies were assessed to be at low risk of bias with four or fewer criteria that were not met and no critical domains missing [11, 27–45]. Four studies were deemed to be at high risk of bias [46–49]. The studies at a high risk of bias have a limited impact on review findings as Fraser [46] was one of 17 studies reporting on cost outcomes; Mileski [47] reported on how frequently a theme was reported in their review; Ness [48] was one of 15 studies reporting on prescribing; and Smith [49] was one of seven studies reporting on illness prevention. Funding sources were often not reported in the included reviews.

**Table 1:** Risk of bias assessment for included studies.

First author (year)	PICO compo-nents included	State-ment of review methods a priori	Justifica-tion of study design	Comprehensive litera-ture search	Dupli-cate study selec-tion	Dupli-cate extrac-tion	List of exclu-ded studies and justifica-tion	Descrip-tion of included studies	Risk of bias	Funding sources	Methods for meta-analysis	Impact of RoB and meta-analysis	RoB in interpre-tation	Hetero-geneity	Publi-cation bias	Conflict of interest
Ansell (2017) [65]	1	1	1	0	1	0	0	1	0	0	NA	NA	0	0	0	1
Barker (2018) [10]	1	1	1	1	0	1	0	1	1	0	NA	NA	1	1	0	1
Carranza (2020) [59]	1	1	1	1	1	0	0	1	1	0	NA	NA	1	1	0	1
Donald (2015) [27]	1	1	1	1	1	1	1	1	1	1	1	1	1	1	1	1
Donald (2013) [54]	1	1	1	1	0	1	0	1	1	0	NA	NA	1	1	0	1
Driscoll (2015) [28]	1	1	1	1	1	1	1	1	1	1	1	1	1	1	1	1
Elder (2015) [63]	1	0	1	0	0	0	0	1	0	0	NA	NA	1	0	0	1
Fraser (2018) [46]	1	0	1	0	0	0	0	0	0	0	NA	NA	0	0	0	0
Fung (2014) [55]	1	1	1	0	0	1	0	1	1	0	NA	NA	1	1	0	0
Galiana-Camacho(2018) [64]	1	1	1	0	0	0	0	1	1	0	NA	NA	1	0	0	1
Garner (2017) [56]	1	1	1	0	1	0	0	1	1	0	NA	NA	1	1	0	1
HQO (2013) [60]	1	0	1	0	0	0	0	1	1	0	NA	NA	1	1	0	0
Hyer (2019) [69]	1	1	0	0	0	0	0	1	0	0	NA	NA	0	1	0	1
Ismail (2013) [66]	1	1	1	0	1	0	0	1	1	0	NA	NA	1	1	0	1
Jennings (2015) [67]	1	1	1	0	0	1	0	1	0	0	NA	NA	1	1	0	1
Jeyaraman (2022) [45]	1	1	1	0	1	1	0	1	1	1	1	1	1	1	1	1
Kuethe (2013) [29]	1	1	1	1	1	1	1	1	1	1	1	1	1	1	1	1
Leduc (2021) [30]	1	1	1	0	1	1	0	1	1	1	NA	NA	1	1	1	1
Loescher (2018) [31]	1	1	1	0	1	1	0	1	1	0	NA	NA	1	1	1	1
Lovink (2017) [32]	1	1	1	1	1	1	0	1	1	0	NA	NA	1	1	0	1
Martin-Misener (2015) [11]	1	1	1	1	1	1	1	1	1	1	1	1	1	1	1	1
Martinez-Gonzalez (2014) [33]	1	1	1	0	1	1	1	1	1	1	1	1	1	1	1	1
McParland (2022) [52]	1	1	1	0	0	0	0	1	1	1	NA	NA	1	1	NA	1
Mileski (2020) [47]	0	0	0	0	0	0	0	1	0	0	NA	NA	0	0	0	1
Morilla-Herrera (2016) [34]	1	1	1	1	1	1	1	1	1	0	NA	NA	1	1	0	1
Ness (2016) [48]	1	1	1	0	0	0	0	1	0	0	NA	NA	0	0	0	0
Newhouse (2011)/ Stanik-Hutt (2013) [35, 36]	1	1	1	0	1	1	0	1	1	0	NA	NA	1	1	0	1
Norful (2019) [61]	1	1	1	0	1	0	0	1	1	0	NA	NA	1	1	0	0
Osakwe (2020) [37]	1	1	1	0	1	1	0	1	1	0	NA	NA	1	1	0	0
Patel (2019) [38]	1	1	1	0	1	1	0	1	1	0	NA	NA	1	1	0	1
Schadewaldt (2011) [39]	1	1	1	1	1	1	0	1	1	0	1	1	1	1	0	1
Scott (2011) [62]	1	1	1	1	0	0	0	1	1	0	NA	NA	1	0	0	0
Smigorowsky (2020) [57]	1	1	1	0	0	1	0	1	1	0	1	1	1	1	1	1
Smith (2014) [49]	1	0	1	0	0	0	0	1	0	0	NA	NA	0	0	0	1
Stratton (2020) [70]	1	0	1	0	0	1	0	1	0	0	NA	NA	0	0	0	1
Sun (2022) [53]	0	0	1	0	1	0	0	1	1	1	NA	NA	1	0	NA	1
Swan (2015) [12]	1	1	1	0	1	0	0	1	1	0	NA	NA	1	0	0	0
Thomas (2019) [40]	1	1	1	1	1	1	1	1	1	1	1	1	1	1	1	1
Tsiachristas (2015) [41]	1	1	1	1	1	1	0	1	1	0	NA	NA	1	1	0	0
Turi (2023) [44]	1	1	1	0	1	1	0	1	1	1	NA	NA	1	0	NA	1
van Vliet, (2020) [42]	1	1	1	1	1	1	1	1	1	0	NA	NA	1	1	1	1
Wu (2020) [68]	1	1	1	1	0	1	0	1	1	1	NA	NA	1	0	1	0
Yang (2020) [58]	1	1	1	0	1	0	0	1	1	0	NA	NA	1	1	0	1
Zhang (2020) [43]	1	1	1	0	1	1	0	1	1	0	1	1	1	1	1	1

### Outcomes

The primary outcomes of the review of reviews are those that assess patient, provider and health system outcomes directly evaluating PHCNP roles. According to Laurant and colleagues [13], those working predominantly in complementary roles provide additional services that are intended to complement or extend existing services, and those working in predominantly alternative roles provide similar services to those for whom they are substituting (usually physicians). We used these distinctions to ascertain if care was in favour of the comparator, equal to or superior to usual care.

### Analysis

Narrative synthesis was used to summarize the findings. As proposed by Olry de Labry et al. [50] and Ramalho et al. [4], outcomes were categorized at the patient (e.g., blood pressure), provider (e.g., knowledge) and health system (e.g., cost) levels. Given the large number of indicators, an iterative process was used to code indicators into categories to synthesize the findings. The indicators included in each category are presented below. Summary tables were developed to describe review characteristics (e.g., year of publication), outcomes, quality assessment, and findings. A record was kept of all review-related decisions (See S3 Appendix). As proposed by Smith et al. [51], no additional quantitative analyses were planned because of the potential overlap in studies included in more than one review.

## Results

**Fig 1:**
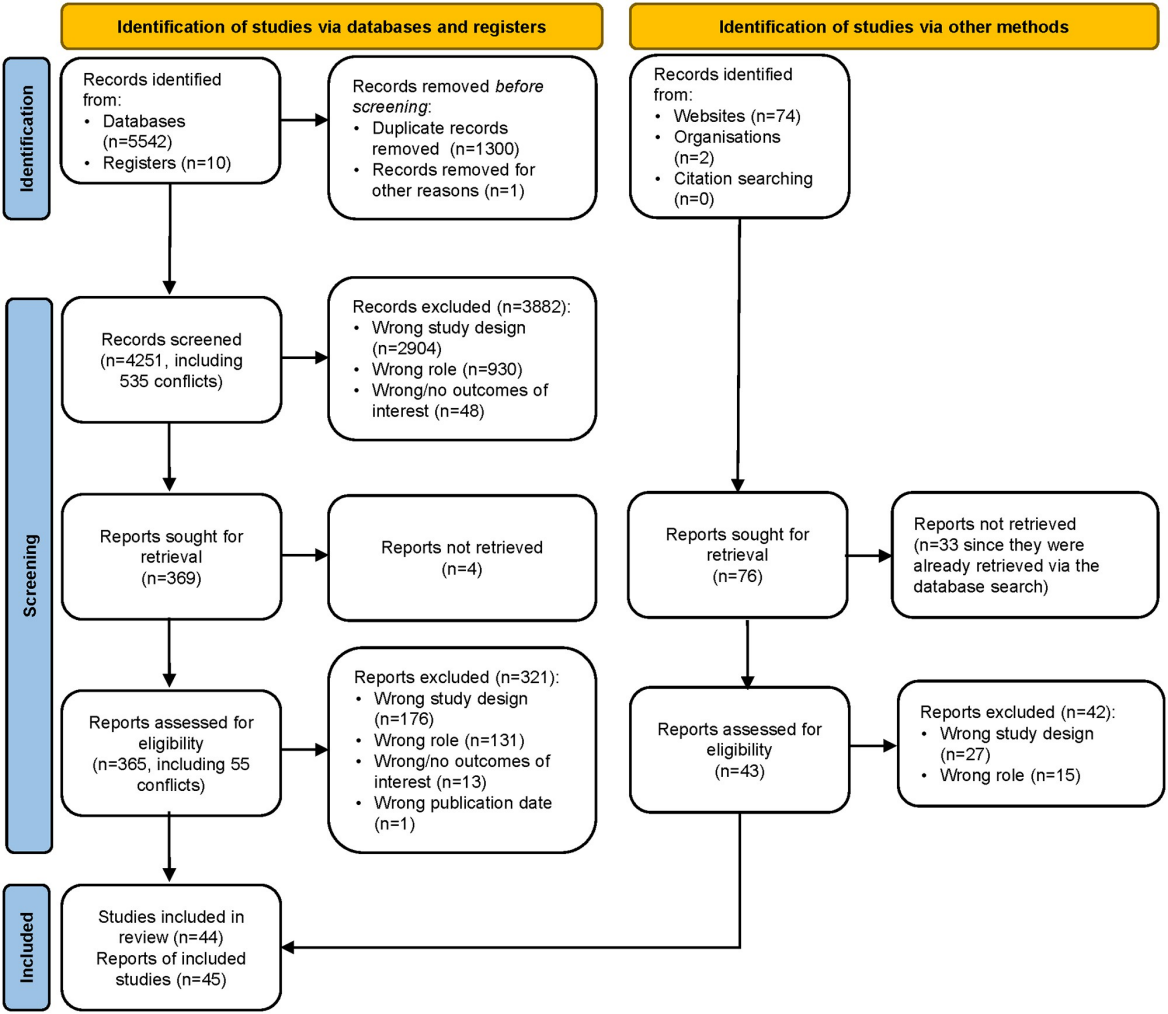
PRISMA flow diagram.

Searches of the electronic databases and registers retrieved 4251 unique records (Fig 1). Title and abstract screening resulted in the exclusion of 3882 records, leaving 365 for full-text screening as we were unable to retrieve four. A further 321 papers were excluded at this stage according to the reasons outlined in Fig 1. Searches of the grey literature and reference lists of included reviews retrieved 43 additional unique records, of which one was ultimately retained. Overall, we identified 44 systematic reviews that included 460 primary studies. We recoded the 271 indicators into 26 broader categories. Among the primary studies, 407 (88.3%) were unique citations and 11.7% (n = 47) were cited two or more times. The reviews were published in 45 papers between 2011 and 2023, with one review [35, 36] published in two papers.

On average, three countries were represented in the reviews (range: not reported to 9) (Table 2). The identified countries included Australia, Austria, Belgium, Canada, China (including Hong Kong), Denmark, England, Germany, Ireland, Israel, Japan, Lesoto, New Zealand, Norway, Portugal, Russia, Scotland, Singapore, South Africa, Spain, Sweden, Switzlerland, the Netherlands, the United States of America, the United Kingdom, and Wales.

**Table 2:** Descriptive table of included systematic reviews.

Study	Study Objectives	Data-bases	Countries	Number of Studies (date range/ designs)	Population Characteristics	Intervention/Comparator	Data Synthesis
Ansell (2017) [65]	To identify interventions designed to reduce wait times for primary care appointments.	n = 6	N = 3 Canada, USA, United Kingdom	N = 11 2004–2010 Controlled before/ after, uncontrolled before/after, survey	Patients in primary care, all health conditions N for intervention and control groups not reported	**Intervention:** Activities aimed at reducing wait times for primary care appointments **Comparator:** no intervention	Descriptive approach
Barker (2018) [10]	Investigate how health outcomes of older adults in LTC vary according to which professional group(s) provides first-line medical care.	N = 4	N = 9 Australia Austria Canada Germany Netherlands New Zealand Spain UK USA	N = 24 After 2000 RCTs, non-randomized intervention, observational.	Residents in LTC NP: n = not reported Physicians: n = 263 Patients: n = 96708	**Intervention**: Assessment of residents, prescription and monitoring of medication, pro-active outreach, education, clinical coaching, care coordination for high-risk patients (including comprehensive geriatric assessment, liaison with specialists), minor procedures, follow-up with families **Comparator:** Usual care, care provided by family physician	Narrative synthesis
Carranza (2021) [59]	Determine how care provided by NPs compares with that of physicians in patient satisfaction and clinical outcomes, for patients seeking specialized care in ambulatory settings.	N = 3	N = 4 USA UK, South Africa Netherlands	11 studies 1995–2016 Cross-sectional studies, cohort study, RCT, quasi-experimental	Patients from different specialities (chronic lung disease, hypertension, diabetes, rheumatology, cardiology, HIV, gastroenterology, pediatric cardiology, psychology and dermatology) NP: n = 1, others not reported Physicians: n = 239 Patients: n = 4529	**Intervention:** Care offered by NPs to patients or for specific disease processes (i.e., HIV, cardiovascular, or diabetes management within primary care), and conducted in an outpatient setting **Control**: usual care, care provided by physicians	Narrative synthesis
Donald (2015) [27]	Determine the cost-effectiveness NPs delivering transitional care in alternative or complementary roles	N = 10	N = 3 UK, Canada, United States.	N = 5 Only RCTs	N = 1171 adults including n = 154 acute asthma patients (>16 years) discharged from hospital. NPs: n = 7	**Intervention**: nurse practitioner-delivered transitional interventions (follow-up care for asthma) **Comparator:** usual care	Meta-analysis
Donald (2013) [54]	Examine effectiveness of APNs in meeting the healthcare needs of older adults living in long-term care.	N = 12	USA	N = 2 Prospective cohort study, quasi-experimental study with 2 control groups	Adults aged 60 years and older living in LTC residential settings, their families or LTC staff Patients: n = 1361	**Intervention:** Primary care provided by NP/MD team to nursing home residents **Comparator**: usual care by physician or usual care by Evercare	Narrative synthesis
Driscoll (2015) [28]	To assess the effects of nurse-led titration (NLT) of medications in patients with heart failure with reduced ejection fraction in terms of safety and patient outcomes.	N = 3	Not Reported	N total = 7 RCTs 2003–2015 **Extracted one NP study = 1 RCT** (Ansari 2003)	**NP study**: Patients diagnosed with heart failure that met the Framingham criteria and a LVEF ≤45% or moderate or severe left ventricular systolic dysfunction on their last visit. Other Professionals: n = 74; Patients: n = 105	**Intervention:** NLT of beta-blockers by study NP under cardiologist supervision **Comparator:** Health professionals were provided education about titration of beta-blockers	Meta-analysis
Elder (2015) [63]	To explore three key strategies designed to promote patient throughput in the ED (increased nursing scope of practice, physician-assisted triage [PAT] and medical assessment units (MAUs)).	N = 5	N = 7 Australia UK, Canada, USA, Ireland, Singapore Sweden.	N total = 21 1980–2014 N = 11 studies for NPs Prospective, retrospective, quasi-experimental, case control, pragmatic randomised cluster, systematic review and descriptive.	N total = 26,774 Patients presenting to the Emergency Department	**Intervention**: Clinical initiatives nurses (CIN) in the Emergency Department (ED) utilising advanced practice nursing including nurse-initiated (NI) activities, such as analgesia and X-rays. An ED NP is an independent practitioner who is able to assess, diagnose, treat, prescribe and refer to other health specialties.	Narrative
Fraser (2018) [46]	Examine methodologies employed in cost analyses of increasing scope of practice for APNs	N = 3	USA	N = 6 (1985–2015) Benefit cost analysis, cost minimization analyses	Reported in one study (n = 9,503). Primary care provided by NPs	Not specified	Economic impact analysis
Fung (2014) [55]	Review reported aspects of the role and performance of psychiatric APNs.	N = 11	N = 3 USA, Australia, Ireland	N = 14 1997 to 2012 English only NP studies: pre post test, RCT, descriptive study; longitudinal design; mixed methods	Community and homebound individuals with serious mental Illness (schizophrenia); after surgery for ovarian cancer, depressed low- income mothers, and adolescents exposed to catastrophic stress Other Professionals: n = 206 Patients: n = 1893	**Intervention:** Providing psychiatric nursing consultation service, psycho-social interventions, transitional care prior to discharge **Comparator:** Usual care, skilled nursing, social work, physical and occupational therapy	Narrative synthesis
Galiana-Camacho (2018) [64]	To show evidence on the results of APN models in the ED including professional competencies, cost-effectiveness results, patient safety and patient satisfaction for future implementation in Spain.	N = 8	N = 3 UK, Australia, USA	N _(Total)_ = 14 2006–2017 Descriptive studies, systematic review, cohort study, RCT	Patients qualified as non-urgent, using the Australasian Triage Scale with scores of 4–5 presenting to the ED or primary care. Most common diagnoses: soft tissue (trauma, loss of skin integrity) (35%) and bone fractures (11%) Patients: n = 6839	**Intervention:** Advanced practice nursing in emergency services, including different models of nurse prescribing and levels of autonomy, fast-track APN care, clinical initiative APN care, intervention and treatment by APNs **Comparator:** Care provided by the physician, other APNs. No comparison group in some studies	Narrative synthesis
Garner (2017) [56]	To assess the effect of nurse-led care (NLC) for patients with rheumatoid arthritis (RA) on quality of care	N = 3	N = 4 Denmark UK Sweden Netherlands	N = 17 1994–2015 RCT, qualitative studies, economic evaluations	Patients with rheumatoid arthritis Patients: n = 1569	**Intervention:** APN-led care in RA **Comparator:** Rheumatologist, junior doctor undergoing training in rheumatology, general practitioner, staff nurse working with rheumatologist, patients previous experience with other care model, inpatient care and day-patient team care	Narrative synthesis
Health Quality Ontario (2013) [60]	Determine effectiveness of specialized nurses with a clinical role in patient care to optimize chronic disease management for adults in the primary care.	N = 6	N = 3 US, UK, Netherlands	N = 6 Published before May 3^rd^ 2012 Only RCTs	Patients with a chronic disease including diabetes, hypertension, coronary artery disease, congestive heart failure Physicians: n = 48 Patients: n = 5088	**Intervention:** Model 1 (nurse alone) and Model 2 (nurse and physician versus physician alone). **Comparator:** Comparable outcomes between Model 1 (physician alone), or improved outcomes or efficiency in Model 2.	Narrative synthesis
Hyer (2019) [69]	Examine the practice patterns of nurse practitioners (NPs) related to weight management in primary care	N = 6	N = 4 North America, United Kingdom Netherlands	N = 13 **Quantitative: n = 8** RCT, quasi-experimental, self-report survey, retrospective data analysis **Qualitative: n = 5** interviews, focus groups	Patient in weight management (obesity/ overweight) practice patterns of NPs with PC adult patients. Excluded: children, adolescents, or pregnancy-related care. Patients: n = 3047	**Intervention:** practice patterns of NPs related to weight management in primary care **Comparator:** usual care (GP). No comparator in several studies which was consistent with the design.	Narrative synthesis
Ismail (2013) [66]	To review the evidence on primary care service interventions to reduce inappropriate A&E attendances	N = 4	N = 1 Australia for NP study	N total = 34 1986 to 2011 Extracted NP study: Before–after/ interrupted time series	Residents in long-term care. N for intervention and control groups not reported	Emergency NP providing first-line medical care to LTC residents	Economic analysis
Jennings (2015) [67]	Determine the impact of emergency NP services on cost, quality of care, satisfaction and waiting times in emergency departments	N = 4	N = 6 UK, Australia, New Zealand, USA, The Netherlands, Canada	N = 12, excluded 2 previous reviews Randomised pragmatic trial of equivalence; Observational studies; retrospective cohort study, audit and case series; survey; Case control; Descriptive design	Minor injuries clinic, walk in centres, emergency department, accident and emergency, casualty, primary care clinics. Patients: n = 36267	**Intervention:** Nurse practitioner services conducted on site in the Emergency department **Comparator:** Traditional emergency department clinical services that do not include nurse led care, only medical lead services	Narrative synthesis
Jeyaraman (2022) [45]	To identify, critically appraise and summarise evidence on the impact of primary healthcare professionals in the Emergency Department triage, on patient flow.	N = 4	N = 3 Australia, England USA	N = 40 studies reported in 44 papers. Extracted N = 14 for NP studies RCTs Pre–post studies	Level 4 and Level 5 triage scale adult and pediatric patients seen in the Emergency Department Patients: n = 4361	**Intervention:** NP triage team: NP located at the ED triage area working along side a triage nurse, either ordering investigations at triage before streaming to ED physician. **Comparator:** Traditional nurse-led triage model	Meta-analysis
Kuethe (2013) [29]	Review the effectiveness of nurse-led asthma care	N = 6	N = 3 Netherlands UK Australia	N = 5 2003–2011 Only RCTs	Asthma nurses, specialist nurses, respiratory nurses Primary care NP Patients with asthma (n = 570)	**Intervention:** Any aspect of asthma management, led by an allied health professional (specialised asthma nurse, NP, PA or specifically trained nursing professional), supervised by a physician **Control**: The same aspect of asthma management provided by a physician	Meta-analysis
Leduc (2021) [30]	Determine the effectiveness and safety of interventions to evaluate and treat patients in long-term care to avoid un-scheduled ED transport.	N = 3	N = 4 United States, Canada, Scotland, Norway	N = 22 2013–2018 4 RCTs and 19 observational studies.	Adult patients in long-term care N for intervention and control groups not reported. Patient numbers not calculated as authors report number of patients and number of beds, depending on the study	**Intervention:** Intervention included a set of tools called Interventions to Reduce Acute Care Transfers (INTERACT). **Comparator:** Standard care or transportation to the ED	Narrative review
Loescher (2018) [31]	Update (2010–2016) a SR (2000–2010) to examine the APNs’ skin cancer knowledge and attitudes, performance of and barriers to clinical skin examination (CSE), recognition of skin lesions, and related training activities.	N = 10	N = 2 USA England Not reported	N = 12 2011–2016 Case studies, Surveys, single-subject experiments, retrospective cross-sectional survey Mixed methods	NPs: N = 783 Patients/cases: N = 344 Control: N = 1255 Four studies reported participants’ age, which ranged from 27 to 64 years and averaged 41 years. Three studies reported years of practice ranging between two and 16+ years. One study included NP students.	**Intervention**: Educational activities and training program lasting between 15 minutes to several months. Some studies included direct feedback from physicians on referrals and assessments. **Comparator:** usual care or no comparator depending on the design	Descriptive
Lovink (2017) [32]	**Aim 1:** To evaluate the effects of substituting nurse practitioners, physician assistants or nurses for physicians in long-term care facilities and primary healthcare for the ageing population. **Aim 2**: To describe what influences the implementation of these roles	N = 6	N = 4 Canada, Japan, Sweden USA	N _(total)_ = 12 studies 1997–2015 RCTs, pre-test, posttest designs without a separate comparison group, posttest only with two group design and historical cohort with two or three group design.	Mean age of the older adults varied from 72 years to 86.3 years. Sample size varied from 114–2575. All patients ≥65 years old, or with a mean age of ≥70 years; Long-term care facilities and primary healthcare. NP: n = 49 Physicians: n = 273 Other Professional: n = 27 Patients: n = 4487	**Intervention:** Medical or preventive care for older patients provided by NPs, PAs, or nurses. **Comparator:** care as usual provided by a physician.	Narrative summary
Martin-Misener (2015) [11]	Determine the cost-effectiveness of nurse practitioners delivering primary and specialised ambulatory care.	N = 10	N = 4 Netherlands, UK, USA, Wales	N = 11 RCTs 1991–2011	Patients all ages in the ED, dermatitis, post-discharge lipid management, diabetes management, coordinate and manage medically unexplained symptoms and high use of primary care. NP: n = 61 Physicians: n = 98 Patients: n = 7497	**Intervention**: NPs see patients at first point of contact, same day appointment, initial visits, colonoscopy, telephone follow-up, post-discharge lipid management, diabetes management, manage medically unexplained symptoms and high use of primary care. **Comparator**: Physicians, usual care, care from dermatologist, gastroenterologists.	Meta-analysis
Martinez-Gonzalez- (2014) [33]	To assess the impact of physician-nurse substitution in primary care on clinical parameters.	N = 4	N = 5 UK, Netherlands, USA, South Africa Russia	N = 11 RCTs 2000–2011 N = 8 studies examining NP roles RCTs of parallel design, cluster RCTs	Nurses provided care for complex conditions including HIV, hypertension, heart failure, cerebrovascular diseases, diabetes, asthma, Parkinson’s disease and incontinence. Excluding non-NP studies n = 4361	**Intervention:** no clear definition of NP roles. Included nurse roles where nurses had no advanced education or decision-making autonomy as NP roles. **Comparator:** Physician care	Meta-analysis
McParland (2022) [52]	To identify types of nurse-led interventions for multimorbidity and which outcomes are positively affected by them.	N = 5	N = 8 Canada, Hong Kong, Israel, Portugal, Spain, Sweden, USA, United Kingdom	N = 20 reported in 28 publications Quasi-experimental: n = 10 RCTs: n = 3 Cohort studies: n = 4 Qualitative: n = 4 Ns don’t match up	Patients with multi-morbidity defined as the coexistence of two or more chronic conditions Average age of participants reported in 13 studies, range: 38.0 to 83.1 (median: 75.6, IQR: 73.9–76.5). Patients: n = 43 899 Providers: n = 19	**Interventions:** nurses in advanced practice develop care plans in partnership with patients, to simplify and improve the quality of care both in the long and short-term using case-management, transitional care interventions, support to self-manage conditions, and an emphasis on continuity of care. **Comparator:** physician-led care, usual care	Narrative synthesis
Mileski (2020) [47]	To increase the understanding of the role an NP has in reducing the risk of hospitalizations and improving quality outcomes among nursing facility residents.	N = 3	Not reported	N = 14 2004–2019 Cross-sectional study, RCTs, retrospective cohort study, Prospective single group intervention, retrospective quasi-experimental, mixed methods, qualitative study, retrospective study, pre/post, observational study	Patients in long-term care, aged care, skilled nursing facilities (SNF), Long term care in hospitals, inpatient rehabilitation Patients: n = 1562	**Intervention: not reported** **Comparison: not reported** The review **did not include comparisons** of physicians to nurse practitioners in relation to hospital readmissions	Rate of occurrence
Morilla-Herrera (2016) [34]	To identify, assess and summarize the evidence of the effect of APN interventions deployed when providing care to older people in different care settings, and to describe the roles and components of the interventions developed by these professionals.	N = 16	N = 8 Sweden, USA, New Zealand, UK, Denmark, China, Switzerland Sweden.	N _(total)_ = 15 RCTs 1990–2014 N = 14 were extracted All the studies were RCTs	Patients’ age was over 65 years old in all the studies. Health conditions more frequently reported: dementia, hip fractures, chronic heart disease and multimorbidity. Settings: transitional care, ambulatory care, home care, hospital care. Patients: n = 4749	**Intervention:** NPs completed health screening; consultations; case management; discharge planning; telephone follow-up; program development; referral; guidance and support through the health system for patients and caregivers; support for patients and caregivers; health education **Comparison**: usual care or control group	Narrative synthesis.
Ness (2016) [48]	To explore the influence on antimicrobial prescribing behaviour of independent nurse prescribers.	N = 8	N = 3 UK, USA, Lesoto	N = 7 Surveys, qualitative study	Study participants were nurse prescribers (NPs) who could independently prescribe. NP: n = 2022	**Intervention:** Nurse prescriber’s decision to prescribe an antimicrobial or not. **Comparator:** no comparator	Narrative synthesis
Newhouse/Stanik-Hutt(2013) [35, 36]	How do NPs affect patient outcomes on measures of care quality, safety, and effectiveness?	N = 4	USA only Studies conducted outside the US were excluded	Total: 37 studies RCTs, observational studies Extracted studies of NPs	Patients in the community or nursing homes, with coronary artery disease, diabetes, chronic conditions, HIV/AIDS, hypertension. Most studies were conducted in urban areas N for intervention and control groups not reported	**Intervention:** NPs worked autonomously or in collaboration with MDs **Comparator:** care provided by physicians and one study comparing NPs to PA	Narrative analysis
Norful (2019) [61]	To synthesize available studies that compare the effects of NP/MD co-management to MD management in primary care.	N = 5	United States only	n = 6 Studies less than 20 years old. RCTs, cross sectional study, case study	Patients in primary care Most common diagnoses included Alzheimer’s dementia, diabetes, hyperlipidemia, and hypertension. Incomplete reporting of n for intervention and control groups	**Intervention**: NP-physician co management teams **Comparator:** Individual physician-led care	Narrative synthesis.
Osakwe (2020) [37]	Examine the current evidence on health and healthcare utilization outcomes associated with NP-home visits.	N = 5	N = 3 Canada UK USA	N = 7 Published before April 2019 RCT, quasi-experimental, observational study, mixed methods study	Patients receiving NP home visits, assessment of medical, psychological and functional abilities of older adults, care coordination, education, and medication management Patients: n = 1757	**Intervention:** Patients receiving home visits from NPs **Comparator:** Usual care	Narrative synthesis
Patel (2019) [38]	To systematically examine the empirical evidence that links NP scope of practice and its impact on access to care.	N = 4	USA only	N = 13: 2013–2017 Retrospective cross-sectional study, survey, repeated cross-sections over time	Characteristics of study participants not reported. National sample used in nine studies, primary care services, community health centers, facilities with mammography services NP: n = 156 851 Physicians: n = 149 784 Other Professionals: n = 95 545 Patients: n = 4 528 200	**Intervention:** Scope of NP practice and access to care. **Comparator:** physicians, certified nurse midwives, physician assistants,	Narrative synthesis; content analysis
Schadewaldt (2011) [39]	To identify effective interventions in nurse-led cardiac clinics including patient education, risk factor assessment and continuity of care.	N = 30	N = 5 England, Scotland, Australia, China, Canada	N = 7 2002 to 2008 Only RCTs	Adults (aged >18 years) admitted to a hospital or a general practice with newly diagnosed or existing CHD, that is angina pectoris symptoms and myocardial infarctions. Patients: n = 3246	**Intervention:** Assessment, monitoring and consultation on risk factors, education sessions and promotion of regular intake of medication, and adherence to a healthy lifestyle. **Comparator:** Usual care provided by a physician	Narrative synthesis and meta-analysis
Scott (2011) [62]	(1) To determine the extent to which the ED is used to screen patients for undiagnosed hypertension; (2) estimate the incidence of undiagnosed hypertension in the ED population; (3) identify and describe the programs for ED hypertension screening; and (4) determine the feasibility of ED-based hypertension screening programs.	n = 7	N = 2 US, UK	N = 4 2003–2008 Prospective cohort studies	Participants age ≥18 years; no prior history of hypertension; minor ED setting; minimum of two ED BP measurements; systolic BP (SBP) ≥140 mmHg and/or diastolic BP (DBP) ≥90 mmHg; and post-discharge follow-up of BP. Patients: n = 2767	**Intervention:** Hypertension screening in the ED **Comparator:** Usual care	Meta-analysis was planned but not conducted
Smigorowsky (2020) [57]	To assess RCTs evaluating the impact of nurse practitioner led cardiovascular care.	N = 7	N = 3 Canada, USA, Netherlands	N = 5 English only 2007–2017 Only RCTs	Over 18 years of age, outpatient HF care, postoperative cardiac surgery, outpatient risk reduction clinic, diagnosed with cardiovascular disease (e.g. coronary artery disease, arrhythmias) Patients: n = 887	**Intervention:** NP-led care included completed assessments, diagnosed new findings, ordered and monitored medications/diagnostic tests The NP could also consult other healthcare providers to give specialty services. **Control:** Usual care	narrative synthesis, meta analysis GRADE
Smith (2014) [49]	Review current literature on participation and roles of APRN/PAs in providing cancer screening and prevention recommendations in primary care settings in the United States	N = 2	US	N _(total)_ = 15 1990–2011 N = 13 (excluded 2 studies reporting on registered nurse midwives)	NP: n = 1698 (reported intervention group only) Physicians: n = 3758 Other Professionals: n = 2197 (Intervention group only) Patients: n = 94611	**Intervention:** APRN/PAs, cervical, breast, or colorectal cancer screening, smoking cessation, diet and physical activity. **Comparator:** physicians, other provider, and no comparison group.	Not reported
Stratton (2020) [70]	Evaluate current interventions targeting clinical skin education (CSE) education for primary care NPs and/or other primary care providers.	N = 4	N = 3 Australia, UK, USA	N = 10 1998–2016 Case studies, pilot study, quasi-exp. studies, implementation study, RCT	NP: n = 5 Physicians: n = 502 Other Professionals: n = 1340 Patients: n = 628 Only 2 studies provided the sample gender and age range	**Intervention:** Didactic portion to review of epidemiology or skin lesion management using feedback from the NP’s prior dermatology referrals to guide her education. **Comparator:** None identified	Narrative
Sun (2022) [53]	To synthesize research evidence of NP visits in home-based primary care.	N = 6	US	N = 14 studies published in 17 papers RCTs: n = 4 Quasi-experimental studies: n = 8 qualitative studies: n = 2	Mainly homebound adults and older adults receiving home care services for heart failure, intellectual or developmental disability, poverty, uncontrolled diabetes, children with special healthcare needs, asthma Patients: n = 4957	**Intervention:** Nurse practitioners provided health assessments, education, care planning and coordination primarily by face-to-face home visits. Most studies included adults but two studies included children with special healthcare needs or asthma **Comparator:** no comparator in half the studies, physicians and/or social workers	Not reported
Swan (2015) [12]	To determine the safety and effectiveness of primary care provided by advanced practice nurses (APNs) and evaluate their potential to alleviate primary care shortages.	N = 3	N = 5 Canada, England, Wales, Netherlands US	N = 10 1974 = 2011 RCTs, RCT follow up study, economic evaluation of RCTs	Patients in primary care. Follow up single consultation, 2 to 4 weeks, or over 12 months. NP: n = 33 Physicians: n = 71 Patients: n = 9852	**Intervention:** Patients presented for a general or condition focused primary care (blood pressure and lipid management), same-day consultation for any reason, or a pre-defined list of conditions including asthma, diabetes mellitus and hypertension **Comparative:** general practitioner or physician	Narrative synthesis
Thomas (2019) [40]	To assess the effects of interventions for treating urinary incontinence after stroke in adults at least one-month post-stroke.	N = 8	Not reported	N = 20 trials 1996–2017 2000 for the One RCT extracted	Patients: n = 232	**Intervention:** Structured assessment and management by continence nurse practitioners **Comparator:** Control groups were ’usual care’ or no treatment.	Narrative synthesis
Tsiachristas (2015) [41]	Investigate the impact of new professional roles on a wide range of health service outcomes and costs	N = 3	N = 7 Australia Canada Norway Sweden Netherlands UK US	N (total) = 41 Included n = 16 1994–2012 Cross-sectional, RCT	Population with different disease including cancer, diabetes, rheumatoid arthritis, and cardiovascular risk. Studies evaluated skill-mix change in chronic diseases such as multiple sclerosis, chronic respiratory disease, Parkinson’s disease. A few studies focused on the general population or acute care. Patients: n = 11 334	**Intervention:** Interventions include consultations and follow-up by NPs, first contact point, follow-up, and case management. **Comparator:** Usual care	Narrative synthesis
Turi (2023) [44]	To synthesize evidence of effectiveness of NP-delivered care to patients with mental health conditions (anxiety, depression, substance use disorders) in primary care	N = 9	USA only	N = 17 studies Pre-test-post-test design, cross-sectional studies, RCT	Adult patients (18+ years of age) with diagnoses or symptoms of anxiety, depression, or substance use disorders, multiple drug use, or alcohol. Patients: n_total_ = 8 975 685	**Intervention:** NP-delivered care (any NP specialty) practicing in a U.S. primary care setting (self-identified as occurring in the primary care setting) **Comparator:** All existing alternative interventions (e.g., physician or physician assistant [PA]-delivered care), usual care	Narrative synthesis
van Vliet (2020) [42]	To describe the activities of NPs and physician assistants (PAs) working in ambulance care, and the effect of these activities on patient, processes of care, costs, and provider outcomes	N = 6	N = 3 UK, Netherlands USA	N = 4 2001–2019 Cross sectional, retrospective observational, action research, descriptive review	Professionals with a master’s degree in ambulance care. NP: n = 26 Other Professionals: n = 3 Patients: n = 2281	**Intervention:** NPs in ambulance care **Comparator:** Paramedics, nurses, usual care, no comparator	Meta-analysis was not possible. Results presented in tabular form.
Wu (2019) [68]	Explore the effectiveness of nurse-led interventions to prevent urinary tract infections in older adults living in residential aged care.	N = 8	USA	N _(total)_ = 4 N = 1 for NPs	Long-term care residents Mean age: 79.6 ± 8.07 (range 66–90) years Patients: n = 87	**Intervention:** Nurse-led interventions to prevent or manage urinary tract infection (UTI): (a) nursing education interventions to prevent UTIs, (b) complementary/alternative therapies to prevent UTIs, such as using cranberry products, (c) early detection of UTIs, and (d) urinary catheter care. NP provided evidence-based supportive management for residents with asymptomatic UTIs **Comparator:** Usual care	Narrative synthesis
Yang (2020) [58]	Synthesize evidence of the impact of state NP practice regulations on U.S. health care delivery outcomes	N = 4	USA only	N = 33 2000–2019 Cross-sectional, cross-sectional time series, retrospective cohort, and quasi-experimental	US Census data, administrative claims insurers, Medicare/ Medicaid patients, medical expenditure Panel Survey (1996–2008), retail clinics, American Diabetes Association, National Ambulatory Medical Care Survey, AMA, 2013, AANP, 2012 Census Block NP: n = 4453 Patients: n = 16 003	**Intervention:** Scope of practice regulation for care provided by NPs **Comparator:** Care provided by physicians, physician assistants	Narrative synthesis
Zhang (2020) [43]	To understand current status of non-physician providers in PrEP care implementation in the US	N = 4	US only	N = 26 No date specified.	NP: n = 2842 Physicians: n = 9789 Other Professionals: n = 5052	**Intervention**: Pre-exposure prophylaxis (PrEP) care provided by NPs **Comparator:** usual care provided by physicians	Meta-analysis GRADE

The indicators were recoded into 26 indicator categories. The indicator categories are subdivided into patient, provider and health system levels. *Patient indicator categories* (n = 10) include activities of daily living (ADLs), adaptation to health conditions, clinical conditions, diagnosis, education-patient, mortality, patient adherence, quality of life, satisfaction-patient and family, and signs and symptoms. *Provider indicator categories* (n = 5) included adherence to best practice-providers, education-providers, illness prevention, interprofessional team functioning, and prescribing. *Health system indicator categories* (n = 11) included access to care, consultations, costs, emergency room visits, healthcare service delivery, hospitalizations, length of stay, patient safety, quality of care, scope of practice, and wait times. Each indicator category is described below (see also S1 Table).

### Patient indicator categories

**Activities of daily living** were reported in seven studies [27, 32, 34–37, 52, 53], and included measures of health status and functional abilities in older adults and children with special needs. Donald [27] and Osakwe [37] noted significant improvements in ADLs (p = 0.04 and p = 0.02, respectively). Parents of children with special needs missed less work (pre vs post 26.3% vs 14.1%, p < .05) and children with special needs attended school significantly more often (missed school >20 days (pre vs post 10.4% vs 11.7%, p >.05) following the provision of NP home care visits [53]. Lovink [32], Morilla-Herrera (2016) [34] and Newhouse/Stanik Hutt (2013) [35, 36] found mixed results, with some studies showing statistically significant improvements in ADLs (e.g., basic ADLs), while other studies in their reviews found no difference (e.g., social functioning) between the intervention and control groups.

**Adaptation to health conditions** was reported in five studies [34, 52, 54–56]. This indicator was examined using 12 different measures. Significant improvements in the NP group were noted in adaptation-related goals [54], reduced disempowerment in treatment [55], life review therapy for homebound elderly patients with depression [55], reduced uncertainty for depressed women with cancer (p = 0.018) [55], reduced distress in women with cancer (p < 0.0001) and in adolescents (p < 0.0001) in the first 24 months (no difference at 36 months) [55], empowerment for patients with arthritis at 12 months (no difference at 24 months) [56], care planning (p = 0.005) [34], and reduced caregiver distress (p = 0.05) [34]. Non significant between-group differences were noted in overall goal-attainment for long-term care residents, and advance directive measures [54] and caregiver burden [52].

**Clinical conditions** represented the largest number of indicators to summarize. These indicators were categorized into health, cardiovascular, cancer care, diabetes, mental health, renal, respiratory, rheumatoid arthritis outcomes. Each one is detailed below:

- **Clinical/Health** outcomes were found in eight studies [12, 35, 36, 41, 42, 47, 53, 57, 58] and included health risk reduction, health status, self-reported perceived health, SF 36 physical composite score, physiologic measures, and clinical outcomes. Milesky et al. [47] reported that NPs improved health outcomes in almost one fifth of theme occurrences. No significant between-group differences were noted in three reviews [12, 35, 36, 57]. Improvements in health outcomes were noted in the NP group in three other reviews [41, 53, 58], but p values were not reported in two studies [41, 58]. Van Vliet et al. highlighted that little is known about the influence of NP roles in ambulance care [42].
- **Clinical/Cardiovascular** outcomes were identified in 10 studies [11, 32, 33, 35, 36, 39, 57, 59–62] and included blood pressure, lipids, heart failure, ejection fraction, N-terminal pro brain natriuretic peptide levels (marker of heart failure), functional exercise capacity (cardiac function), left-ventricular end-diastolic volume index, undetected hypertension, and vascular risk reduction. Significant improvements in favour of the NP group were found for *blood pressure outcomes* in four reviews [11, 33, 39, 60]. Trends towards improvement of metabolic outcomes and reduced vascular risk favouring the NP group were noted in studies by Carranza [59] and Smigorowsky [57] (no p value reported). Non significant results were noted in four studies [33, 39, 60, 61]. Rates of previously undetected high blood pressure ranged from 14% to 63% in one review (no p value reported) [62]. *Lipids* were measured using cholesterol, mean level of total cholesterol, reduction in high density lipoprotein and triglycerides. Trends in reduction of total cholesterol and high density lipoprotein favouring the NP group were found in studies by Schadewaldt [39] and Newhouse/Stanik-Hutt [35, 36], while no significant differences were noted in three studies [33, 39, 60]. Schadewaldt et al. [39] identified fluctuations in lipid control at 3, 6, 12 and 18 months. Mixed results were observed in two reviews, with equal to superior results for the NP group [35, 36, 39]. Statistically significant improvements were noted in the ejection fraction and composite score for patients with heart failure in favour of the NP group [32].
- **Clinical/Cancer care** was identified in two reviews [31, 49]. Measures included clinical examination of skin lesions, accuracy of skin lesion examinations, and post-treatment survivorship care. Loescher et al. [31] indicated a trend towards a greater number of examinations and a decrease in the number of referrals to a dermatologist and in the number of biopsies (no p value reported). Smith and colleagues [49] highlighted they were unable to identify studies involving NPs in their assessment of post-treatment survivorship care.
- **Clinical/Diabetes** outcomes were highlighted in seven studies [32, 33, 35, 36, 53, 59–61]. Indicators included HbA1C, HbA1C at 3 months, two or more HbA1C tests, glucose, diabetic control, foot examination, retinal exams, and annual eye exams. No significant differences between groups in HbA1C were noted in three reviews [32, 35, 36, 60], including a meta-analysis that included four studies (n = 589 patients) [33]. Mixed results were reported for HbA1C in one study [61]. Improvements in HbA1C were found in two studies [53, 59], but no p value was indicated in one review [59]. Glucose control was identified in two studies (no p value reported) [35, 36, 59], with a trend towards improvement in the NP group noted by Carranza (2021) [59]. Diabetes control was indicated in two studies [60, 61], with significant improvements favouring the intervention group in one study [61] and no differences found in the other study [60]. Retinal exams and foot exams were conducted more frequently in the NP group in one review [60] and no significant differences were noted in requests for annual exams by ophthalmologist in the second study [61].
- **Clinical/Mental health** outcomes were reported in eight reviews [12, 27, 34, 39, 44, 52, 55, 57]. Significant reductions were noted in the NP group in anxiety rehabilitation (p < 0.0001) [27], psychological stress [55], uncertainty for depressed women with cancer [55], depression (p = 0.0005) [55], and illicit drug use (p = 0.029, p = 0.046) [44]. NP-delivered motivational interviewing led to a significant increase in patient depression self managemen in one review (p ˂ 0.001) [44]. Trends towards reductions in anxiety and in cognitive impairment and severe depression at 12 months were noted but p values were reported inconsistently [44, 52]. There were no significant differences in: depression and anxiety in long-term care [34]; primary care at 1 and at 4 years [39]; the SF 36 mental composite score for patients with cardiovascular disease [57]; subjective health status [12]; and alcohol use disorder identification test scores [44]. Schadewaldt et al. [39] cautioned against a risk of a cross-over effect in the control group at 4 year with decreased odds for depression (p = 0.001).
- **Clinical/Renal** outcomes were identified in two studies [33, 60]. Significant differences favouring the NP group were noted in urinalysis results in one review (p < 0.01) [60], whereas no significant differences between groups were noted in mean urine sodium excretion and serum creatinine at six months in another review [33]. There was a trend towards higher levels of urinary albumin excretion favouring the control group, but no p value was reported.
- **Clinical/Respiratory** outcomes were outlined in six reviews [27, 29, 33, 35, 36, 53, 60]. No significant differences were noted between the intervention and control groups for change in maximal peak flow in asthma, emergency nebulization, frequency of exacerbation, 6-month follow-up, asthma severity, asthma symptoms, absence from school or work due to asthma, forced expiratory volume at one second (FEV1), peak flow rate, airway hyper-reactivity using PD/PC20 methacholine/histamine, as well as lung function at 12 and 24 months. Mixed results noted for asthma control. No significant difference noted by Donald et al. (2015). Significant reductions in nightime symptoms (t test: 3.966 df 19 p < .05), oral steroids (t test: 3.750 df 18, p < .05), better adherence to therapy (z score -3.272, p < .05), and level of control (z score -4.132, p < .001) noted in one review [53].
- **Clinical/Rheumatoid arthritis** outcomes were noted in two studies [56, 59]. Disease activity in rheumatoid arthritis was assessed in one review [56] with significant reductions in the NP group across all included studies (p < 0.05). Disease progression and severity were assessed in one review, with trends towards improvements noted [59]. Morning stiffness was examined in one review, with equal to significant improvements reported in the NP group (p = 0.01) [56].

**Diagnosis** was identified in three studies [59, 63, 64] and included diagnostic accuracy, most common diagnoses, and nurse-initiated X-Rays and treatments. The most common diagnoses in the Emergency department included soft tissue injuries and fractures. Improvements were noted in adenoma detection (p < 0.001), diagnosis and treatment (p < 0.001) with the NP group.

**Education-Patient** included seven studies [11, 34, 41, 59–61, 64], all of which noted positive trends with the NP group for this indicator category. Measures included patient information, health education, pharmaceutical treatment, symptom relief, discharge information, providing written documentation as a strategy, increased knowledge, information on who to contact if needed, dietary and activity recommendations including sodium reduction, moderation in alcohol consumption, weight control and reduction. Statistical significance in favour of the NP group was reached in four reviews [11, 34, 60, 61].

**Mortality** was examined in eleven studies [10, 27, 28, 30, 32, 34–36, 39, 52, 56, 59]. Seven reviews [27, 30, 32, 34–36, 52, 56] found no significant differences between groups in mortality, mortality at 90 days and 24 months. Significant reductions were noted for the NP group in three reviews [28, 52, 59] for mortality and mortality at 12 months. Mixed results were identified in two reviews, where one review found that 1 out of 4 included studies identified a lower risk of mortality in the control group [10]; the other review [39] found statistically significant reductions in total mortality for the NP group at the 4-year follow-up (p = 0.038). This trend became non significant at the 10-year follow-up because of the risk of a cross-over effect among participants [39].

**Patient Adherence** was examined in three studies [12, 27, 29]. Results favoured NP care [27] with increased patient adherence with beta-blockers, statins, and angiotensin-converting enzyme inhibitors, and attendance at cardiac rehabilitation. These estimates did not reach statistical significance. Swan and colleagues reported that patient adherence to follow-up was higher in the NP group, but did not report a p value [12].

**Quality of Life** (QOL) was identified in 12 reviews [27, 29, 32, 34, 37, 39, 41, 55, 57, 59–61]. Statistically significant improvements favouring NPs were highlighted in three reviews for physical and mental QOL over time (p < 0.001), diabetes [60], and QOL and general health perception [39]. No differences between groups were noted in asthma or respiratory QOL [27, 29], mental QOL [60], QOL at 18 months and four years [39], and quality-adjusted life years, as well as health-related QOL after cardiac surgery [57]. Kuethe [29] undertook a meta-analysis of two studies and found no between-group difference in QOL. Equal to superior findings on QOL was found in three reviews [34, 41, 61]. One review identified significant deterioration in physical QOL in the NP group (p = 0.4) [60].

**Satisfaction-Patient and Family** was examined in 18 reviews [11, 12, 27, 34–36, 38, 40, 41, 45, 52–54, 59, 60, 64–67]. Equal to improved patient and family satisfaction were noted consistently for all reviews. Statistical significance and p values reported inconsistently. Significant improvements favouring the NP group were specified in four reviews [11, 27, 40, 67]. Patel et al. [38] noted contradictory findings for patient satisfaction with usual care (control group) in jurisdictions in the United States with the least restrictive vs the most restrictive NP scope of practice policies.

**Signs and Symptoms** included nine reviews [27, 29, 34, 39, 40, 56, 59, 61, 68], and comprised: symptom management, symptom improvement, urinary tract infections, pain, fatigue, arthritis impact, symptom free days, symptoms of dementia, cognitive and behavioural changes, angina symptoms, continence after treatment, urinary symptoms (i.e., frequency, nocturia, urgency and urinary incontinence) at three and six months, daytime and night-time leakage, leakage severity at three months, total number of symptoms at three months, overall symptoms at six months, and management of asymptomatic urinary tact infection. Significant differences favouring the NP group were noted for symptom management [59], managing symptoms of dementia [34], all urinary symptoms, total number of urinary symptoms and daytime severity of leakage at three months [40] and managing asymptomatic urinary tract infactions with increased fluids (p < 0.001), frequent toileting (p < 0.001), and cranberry juice (p < 0.05). Equal to significant results favouring the NP group for pain and fatigue were reported [56]. All other findings showed no significant differences between the groups.

### Provider indicator categories

**Adherence to best practice-Providers** was identified in six studies [12, 27, 32, 39, 44, 61]. NPs were reported to improve adherence to best practice guidelines for medications including beta-blockers, statins, angiotensin-converting enzyme inhibitors, and aspirin intake at 1-year follow-up (p < 0.001) [27, 32, 39, 61]. No significant differences were noted for medications including aspirin and clopidogrel [27]. No significant differences were noted for NPs providing mental health guideline-recommended care for medications, counselling, cognitive behavioral and problem-solving therapy to older adults, patient monitoring and motivational interviewing in one review [44]. Mixed findings were observed for one review in long-term care [32] and another in primary care [12]. In long-term care, Lovink et al. [32] found no significant differences in adherence and compliance with guidelines including annual mandatory histories and physical examinations. Significant findings favouring the NP group were noted for care of vulnerable elders (p < 0.001), secondary prevention, patients with dementia, incontinence, and adherence to care and co-management [32, 61]. In primary care, Swan and colleagues [12] found that results favoured NPs in providing disease-appropriate care, informing patient on the cause of illness, symptom relief, and chances of illness recurrence in two out of three studies, with no significant differences in the third study.

**Education-Provider** included five studies [31, 32, 64, 69, 70]. Provider knowledge was assessed for the use of the Australasian Triage Scale, patient counselling on obesity, skin cancer detection and training, early detection of skin cancer, and orientation in long-term care. Two reviews [31, 70] reported that knowledge of clinical examination of skin lesions improved with training but no p value was indicated. Stratton et al. [70] identified that didactic sessions for providers in dermatology lasted 14 minutes and clinical apprenticeships were up to six months but no indication of timing or frequency was provided. The modes of delivery of the didactic interventions were face to face or using observations by experts.

**Illness Prevention** was identified in seven studies [27, 39, 49, 55, 56, 59, 60]. Illness prevention indicators included health behaviours (physical activity, diet/nutrition, smoking status, cessation and cessation up to 1 year), weight/body mass index and weight loss, screening women for depression, health assessment, screening for cancer (cervical, breast, colorectal) and human papillomavirus (HPV) vaccination. Significant differences favouring the NP group were noted in four reviews [49, 55, 56, 60]. Results favoured the NPs for weight loss in one study (no p value reported) [59]. Another review noted equal to significant findings for smoking cessation up to 1 year [39]. Smith and colleagues [49] highlighted that no studies examined HPV vaccination performed by NPs, and that two out of four studies showed that physicians reported more colorectal cancer screening than advanced practice nurses and physician assistants [49].

**Interprofessional Team Functioning** was examined in four reviews [32, 44, 52, 69]. Providers expressed positive perceptions of high functioning interprofessional teams [69] and improved communication (McParland, 2022) (no p value reported) [52]. In their review on long-term care, Lovink et al. stated that no provider outcomes were identified [32]. In mental health, NP-led collaborative care led to significant improvements in patients’ depressive symptoms [44]. In addition, a collaborative practice model between NPs and NP specialized in mental health increased the number of case reviews per month from 5 to 15, and reduced the number of referrals to the specialized NP in mental health from 19 to 5 (no p value reported) [44].

**Prescribing** included 15 reviews [10–12, 28, 29, 32, 34, 43, 44, 48, 56, 59, 60, 68, 69]. Significant results favouring NPs were found for the initiation, up-titration and target doses of beta-blockers (p < 0.001) [28], as well as for the prescription of cholinesterase inhibitors and antidepressants [34]. Prescription of anti-microbial agents favoured NPs in one review (no p value reported) [48]. No significant differences in prescribing were noted in six reviews [11, 12, 29, 32, 59, 68]. A meta-analysis comprising 26 studies by Zhang et al. [43] examined awareness of pre-exposure prophylaxis (PrEP) implementation cascade by NPs and physicians and found lower scores in the NP group (p < 0.05), no difference in willingness to prescribe PrEP, and higher odds of prescribing PrEP for NPs (p < 0.05). Equal to statistically significant results favouring the NP group were noted in the appropriateness of medication prescribing in two reviews [10, 60], and prescribing diagnostic tests [12]. Under-prescribing of weight-loss pharmaceuticals was noted by Hyer [69]. Mixed results were noted in one review [44] where NPs prescribed equal to significantly more anxiolytics (p ˂ 0.001) and significantly more anti-depressants. No differences were noted in the odds of being prescribed pharmacothrapy for alcohol use disorder [44].

### Health system indicator categories

**Access to care** was found in five reviews [47, 58, 59, 65, 70]. Improved access to care was noted using open access scheduling for the NP group (no p value reported) [65]. The addition of pediatric NPs increased the proportion of urgent appointments (p < 0.001) and access between visits (no p value reported) [59]. The implementation of an NP-led surveillance clinic increased access to treatment for precancerous lesions with an increase of 18.3% (n = 828) and 6.2% were treated for non melanoma skin cancer [70]. In their respective reviews, Yang [58] reported equal to significant improvements in primary care access (no p values reported), and Milesky [47] noted that the theme of increased access to health care occurred in 10.3% of theme occurrences.

**Consultations** were identified in 11 studies [11, 12, 27, 29, 31, 32, 42, 56, 58–60]. Results included significant reductions in the number and duration of consultation calls in rehabilitation (p = 0.05), and the total number and duration of consultations calls (p < 0.05) [27] for the NP group. Significantly higher number of visits and referrals to physicians were identified when there were restrictions on scope of practice [56, 58]. Referrals for echocardiographs among patients with presumed congestive heart failure were signifiantly higher in the NP group (p < 0.001). Consultation times were longer for NPs in several reviews [11, 56, 60]. A meta-analysis that included 2500 patients estimated an increase of 4.1 minutes in the intervention group (p < 0.0001) [11]. For patients with chronic conditions (diabetes and hypertension), one review [60] reported an average increase of 11 minutes in consultation time (p <0.001). No comparison in consultation times was presented in one review but consultation times decreased between the first and third visit [29], and one review did not report measures of consultation times [59]. No significant between-group differences were noted in the number of referrals in three reviews [11, 12, 42]. Unnecessary referrals to dermatologists and biopsies decreased [31] (no p value reported), and unplanned consultations for acute conditions in long-term care increased significantly in the NP group (p < 0.0001) [32].

**Costs** were reported in 17 studies [11, 12, 27, 29, 30, 32, 34, 41, 46, 47, 52–54, 56, 58, 59, 67]. Reductions in costs were noted in five studies for the NP group [11, 32, 34, 46, 47]. Martin-Misener [11] also completed a meta-analysis of two studies that reported costs (n = 2689 patients) and found a mean difference of -6.41 euros (2006 euros). No significant differences in costs were noted in five reviews [27, 52, 54, 56, 67]. In addition, Fraser et al. [46] noted that broader scope of practice was projected to have an important impact on state-level economic activity. Similarly, Yang and colleagues [58] found that fewer restrictions on scope of practice was associated with lower costs, and increased hourly earnings of NPs was associated with a decrease in physician earning between 2005–2010. Equal to lower costs were observed in three reviews for outpatient visits [29], laboratory services [12], and home-based primary care [53]. Costs were higher for NPs vs. physicians in one review of patients with lung disease (p < .001) [59]. Mixed results were noted in two reviews with both increased and decreased costs for patients requiring long-term care and end of life care [30] and a wide range of chronic conditions [41].

**Emergency Room Visits** were included in 14 studies [11, 28, 30, 32, 35–37, 42, 45, 53, 55, 60, 63, 66, 67]. Reductions in Emergency Room visits were noted in half of the reviews [30, 32, 37, 45, 60, 66, 67]. Statistical significance not always specified. Increases in the number of Emergency Room visits were identified in two reviews [32, 55], and the number of Emergency Room visits remained unchanged in studies reported in three reviews [11, 28, 35, 36]. One review reported equal to statistically significant reductions in Emergency Room visits [53]. Jeyaraman et al. noted an increase in the number of Emergency Room visits in the NP-led care model to improve patient flow through the Emergency Room compared with the control group (no p value reported) [45]. No data were available on non-conveyance rates (ambulance transport to the Emergency Room) for NPs [42].

**Healthcare Service Delivery** was outlined in 17 reviews [11, 12, 27, 30, 32, 34, 37, 41–43, 55, 56, 58–60, 65, 67], and was examined using: no show rates, continuity of care, additional interventions, transitional model of care for patients with schizophrenia, screening women with depression, home care, number of primary care visits, acceptability of nurse-led care, models of care, end-of-life care, hospital days, and number of outpatients contacts. Significant results favouring the NP group were noted in five reviews [30, 37, 42, 55, 59], and equal to superior care was noted in another eight reviews [12, 32, 34, 41, 56, 58, 65, 67]. Zhang et al. [43] described key barriers and ideal location to providing PrEP. A meta-analysis of three studies (n = 2562 patients) led by Martin-Misener [11] found that NPs were more likely to ask patients to return than physicians within two weeks of the index visit (p < 0.001), but return visits for any reason were equal at 1 year.

**Hospitalization** were included in 15 reviews [10, 11, 27–30, 32, 34–37, 47, 53, 57, 59, 60]. Reviews measured unplanned hospital transfers, hospital admission rates for patients with lung disease, rehospitalizations, index re-hospitalization up to 42 days, over 90 days and 180 days, any re-hospitalization up to 180 days, hospitalizations for congestive heart failure, hospital admissions, hospital days, hospital admission in long-term care, number of hospital days in long-term care, number of hospital admissions from primary care, at least one hospitalization, institutionalization for residential care, readmissions, 30-day readmission rates for heart failure. No significant differences were noted in five reviews [11, 28, 29, 37, 57]. No differences were found following a meta-analysis of index rehospitalizations up to 42 days and any rehospitalizations up to 180 days [27]. Equal to statistically significant outcomes favouring NPs were noted in nine reviews [10, 27, 30, 32, 34–37, 53, 60]. Reductions in nursing home admissions were noted in intervention groups in one review but no p value was reported [53]. One study included in one review found a significant reduction for hospital admissions rates for patients with lung disease in favour of the control group (66 patients in the NP group vs. 42 in the physician group, relative rate: 1.52, p = 0.03) [59].

**Length of Stay** was identified in seven studies [30, 34–36, 45, 60, 63, 64], and was assessed using time spent in the Emergency Room and for patients who were admitted to the hospital. Equal to statistically significant reductions in ED length of stay noted in one review (median: -28.50 minutes) [45]. Trends towards a decrease in the time to triage and a greater number of patient discharges from the ED at 60 minutes, 90 minutes, 4 hours and under 6 hours favour the NP group but level of significance was not indicated [45]. No significant between-group differences were noted in three reviews [35, 36, 60, 63]. Decreased length of stay was noted in the reviews led by Leduc [30] (no p value), and by Morilla-Herrera [34] (p < 0.001) in long-term care. Galiana-Camacho et al. [64] did not provide a comparison of the average length of stay, but the authors noted that 78.5% of patients were seen in less than four hours.

**Patient Safety** was reported in 11 reviews [27, 28, 31, 34, 52, 56, 58–60, 64, 67], and was examined using: treatment complications, adverse effects, falls, adverse events, unplanned readmissions, adequacy of diagnostic tests, medication interactions, unplanned visits to family physicians, hospitalization, risk factor management, inappropriate management, missed injuries, malpractice, unnecessary referrals, and unnecessary biopsies. Five reviews [27, 31, 34, 60, 64] favoured the NP group and five reviews [28, 52, 56, 59, 67] showed no significant differences between the intervention and control groups. Further, Yang and colleagues [58] highlighted that 31% lower malpractice payment occurred over a 13-year span per 1,000 physicians for states with full NP practice authority compared to those with a restricted practice authority.

**Quality of Care** was examined in six studies [29, 32, 41, 47, 58, 67]. Significant improvements in quality of care were noted for patients receiving medical care from Emergency Room NPs (p <0.02), asthma-specific care for children (p < 0.05), vulnerable elders (p < .001), and positive health outcomes [58]. Equal to statistically significant improvements were found in the NP group by Kuethe et al. [29] and Yang [58]. Improvements were highlighted in the Tsiachristas [41] review but no p value was reported. The Milesky review [47] indicated that NPs improved quality in almost one fifth of the theme occurrences identified in their sample.

**Scope of Practice** was identified in six reviews [38, 47, 58, 63, 64, 69]. Milesky and colleagues [47] reported that unrestricted or least restrictive scope of practice was mentioned in 7.35% of theme occurences in their sample. Positive trends and positive impacts reaching statistical significance were noted when less restrictive scope of practice regulations, as well as increased or full practice autonomy, were found in the reviews.

**Wait Times** were reported in seven studies [31, 42, 45, 59, 63, 65, 67]. Four studies reported reductions in wait times for appointments [45, 59, 63, 65] and a fifth [31] reported an increased number of patients seen in a timely manner with the NP group. Equal to statistically significant reductions in wait times in the Emergency Room were found for the NP group in two reviews [63, 67]. No comparison for on-scene treatment time were available [42]. Equal to statistically significant reductions in time to initial provider assessment (mean time difference: -3.00 min, 95%CI [-3.47, -2.43] to -50 min, 95%CI [-53.63, -46.37]) noted in one review [45]. Equal to statistically significant reductions in the number of patients who left the Emergency Room without being seen in one review (no p value reported) [45]. The number of patient who left against medical advice was unchanged in one review (no p value reported) [45].

## Discussion

The review of systematic reviews identified 44 systematic reviews with 460 primary studies documenting 271 indicators sensitive to PHCNP practice. The indicators were recoded into 26 indicator categories at the patient, provider, and health system level to summarize review findings. Indicators related to examining clinical conditions represented the largest portion of indicators. Additional knowledge was gleaned from one review examining the effectiveness of nurse practitioner care for patients with mental health conditions in primary care. The findings across reviews are very consistent with equal or improved outcomes for patients in primary care, home care and long-term care settings when PHCNPs provide services. Very few indicators in the reviews favoured the control group. Indicator categories pointed to equal or superior care for the PHCNP group, with the exception of the length of consultations, which were longer in the NP group. Other indicators favouring the control group (i.e., requests for echocardiograms for patients with newly diagnosed congestive heart failure, patients requiring follow-up for lung cancer, cancer screening) were identified at the level of single studies within the reviews. Thus, PHCNPs have been found to make significant contributions to safe, efficent and effective care for patients across primary care, home care and long-term care.

The review of reviews aimed to assess the quality of the included systematic reviews. Nineteen reviews were assessed to be at low risk of bias (i.e., high confidence in the results) and four reviews (9%) were assessed to be at high risk of bias with several evaluation criteria that were unmet. The remaining reviews did not meet between 4 and 9 criteria, and were at low to moderate confidence in the results (i.e., non-critical weaknesses). The studies at a higher risk of bias contributed data to outcomes related primarily to costs, prescribing and illness prevention. Several higher quality reviews (range: 7 to 17) also provided data for these indicators. Given the small number of studies at high risk of bias and the large number of studies reported in these indicator categories, we believe that these four reviews have no impact on our review findings. The studies were assessed using AMSTAR 2, where the focus is on identifying study weaknesses [26]. Key areas of disagreement among the research team reviewers were related to the interpretation of certain descriptors in the AMSTAR 2 guide. Some examples included the use of “and/or” within the same criterion, determining what constitutes a specific statement of duplicate extraction of study data in the published reviews, the comprehensiveness of the literature search, the justification of study exclusion and the development of a list of excluded potentially relevant studies, and the need to address several questions within each item. Among the six critical domains of risk of bias for systematic reviews described by Shea et al. [26], item 4 for the comprehensive literature search was scored as “not attained” if the search did not review trial registries. This criterion was not applicable to all the reviews and search strategies. For item 7, several reviews did not attain this criterion because a list of excluded potentially relevant studies was not generated. It was not clear how this could be assessed in studies where the research questions included broader concepts (e.g., scope of practice) and the list of potentials relevant studies were only determined at the last step of study identification. It was also challenging to determine the risk of bias for the included studies in our review as a whole, given the breadth of research questions and aims that were identified in the current review (e.g., ranging from specific clinical conditions to the impact of policies on scope of practice). Other instruments, such as the Critical Appraisal Skills Programme (CASP) Checklist, may be better suited for this type of review [71].

## Strengths and limitations

Our review of systematic reviews included a definition for NP roles that is recognized internationally. Our study strengthens the knowledge base and enhances the generalizability of our findings across countries. There were no language or geographical restrictions for studies included in the review of systematic reviews, which added to our ability to capture relevant reviews. However, because the review focused on studies that clearly defined the NP role and indicators, the overview may have excluded studies that examined NP roles but did not provide clear role definitions.

## Future research

The review of systematic reviews has allowed us to map areas of research where there are highly consistent findings across systematic reviews, in addition to identifying areas where there are gaps in the knowledge base. One review highlighted positive perceptions of interprofessional team functioning. It appears important to expand on this area of research to examine team functioning in other areas such as long-term care and home care, and include the perspective of patients and families. Additional research to understand the impact of specific factors such as role clarity, team processes (e.g., communication, care coordination) and role implementation also needs to be undertaken to make a clearer link between role implementation, scope of practice, team functioning and outcomes [15]. PHCNPs practice in a wide range of setting and with diverse patient populations, including vulnerable populations [72]. No indicators were identified to examine post-treatment survivorship care, the effects of high and low fidelity simulation, cultural safety and cultural sensitivity, and the integration of patients and families as partners in care for PHCNPs working PHC settings [73–75]. Turkelson et al. [76] demonstrated that using simulations with NP students increased cultural sensitivity with patients of Hispanic origins. No studies were identified examining experiences of Indigenous Peoples with regards to cultural safety. It is crucial to fill these knowledge gaps to give patients and families and people in vulnerable situations a voice in their healthcare.

## Conclusion

Our review of systematic reviews identified 44 systematic reviews. The findings across reviews are very consistent with equal or improved outcomes for patients in primary care, home care and long-term care settings for the PHCNP group. The identification of indicators sensitive to the practice of PHCNPs from the perspective of patients, providers and the healthcare system will allow patients, clinicians, researchers, and decision-makers to understand how these providers contribute to outcomes of care. Gaining an understanding of the patient perspective is particularly important in the context of patient-centered care and adapting services to the needs of vulnerable populations (e.g., residents in long-term care, patients with mental health conditions or low socio-economic status). PHCNPs, other clinicians and decision-makers can track these indicators and determine if PHCNP roles are used optimally to respond to patient care needs.

## Supporting information

S1 Checklist PRISMA 2020 checklist. (PDF)

S1 Appendix Search strategies for the published literature. (PDF)

S2 Appendix Search strategies for the grey literature. (PDF)

S3 Appendix Record of review-related decisions. (PDF)

S1 Table Extraction of review results by indicator category. (PDF)
