# Supplementary material for: Identifying indicators sensitive to primary healthcare nurse practitioner practice: A review of systematic reviews
Source: PLoS One. 2023 Sep 7;18(9):e0290977. doi: 10.1371/journal.pone.0290977 (PMC10484467; doi:10.1371/journal.pone.0290977)
Supplement: S1 Appendix — (PDF) [file pone.0290977.s002.pdf]

S1 Appendix. Search strategies for the published literature.

1. PubMed

((((("Ambulatory Care Facilities"[MH] OR "Ambulatory Care"[Mesh:NoExp] OR "Community Health Centers"[MH] OR "Community Health Nursing"[MH] OR "Community Health Planning"[MH] OR "Community Health Services"[MH] OR "Community Medicine"[MH] OR "Community Mental Health Services"[MH] OR "Delivery of Health Care"[MH] OR "Family Practice"[MH] OR "General Practice"[MH] OR "General Practitioners"[MH] OR "Group Practice"[MH] OR "Health Personnel"[MH] OR "Health Services Administration"[MH] OR "Home Care Services"[MH] OR "Home Nursing"[MH] OR "Homes for the Aged"[MH] OR "Housing for the Elderly"[MH] OR "Intermediate Care Facilities"[MH] OR "Long-Term Care"[MH] OR "Nurses, Community Health"[MH] OR "Nursing Care"[MH] OR "Nursing Homes"[MH] OR "Nursing Stations"[MH] OR "Nursing"[MH] OR "Nursing, Team"[MH] OR "Office Nursing"[MH] OR "Outpatients"[MH] OR "Patient Care Team"[MH] OR "Physicians, Family"[MH] OR "Physicians, Primary Care"[MH] OR "Primary Care Nursing"[MH] OR "Primary Health Care"[MH] OR "Professional Autonomy"[MH] OR "Professional Practice"[MH] OR "Remote Consultation"[MH] OR "Respite Care"[MH] OR "Rural Health Services"[MH] OR "Rural Health"[MH] OR "Rural Nursing"[MH] OR "Suburban Health Services"[MH] OR "Telemedicine"[MH] OR "Telenursing"[MH] OR "Telerehabilitation"[MH]) OR ("ambulatory care"[tiab] OR "Care continuity"[tiab] OR "CHSLD"[tiab] OR "CHSLDs"[tiab] OR "CLSC"[tiab] OR "CLSC's"[tiab] OR "CLSCs"[tiab] OR "Community care"[tiab] OR "Community clinic"[tiab] OR "Community clinics"[tiab] OR "Community doctor"[tiab] OR "Community doctors"[tiab] OR "Community facilities"[tiab] OR "Community facility"[tiab] OR "Community health center"[tiab] OR "Community health centers"[tiab] OR "Community health centre"[tiab] OR "Community health centres"[tiab] OR "Community health nurse"[tiab] OR "Community health nurses"[tiab] OR "Community health nursing"[tiab] OR "Community health plan"[tiab] OR "Community health planning"[tiab] OR "Community health plans"[tiab] OR "Community health service"[tiab] OR "Community health services"[tiab] OR "Community medicine"[tiab] OR "Community mental health service"[tiab] OR "Community mental health services"[tiab] OR "Community Nurse"[tiab] OR "Community Nurses"[tiab] OR "Community Nursing"[tiab] OR "Community physician"[tiab] OR "Community physicians"[tiab] OR "Community practice"[tiab] OR "Community practices"[tiab] OR "Community program"[tiab] OR "Community programs"[tiab] OR "Community service"[tiab] OR "Community services"[tiab] OR "Continuity of care"[tiab] OR "Counseling"[tiab] OR "Counselling"[tiab] OR "Delivery of health care"[tiab] OR "Delivery of healthcare"[tiab] OR "Distance Counseling"[tiab] OR "Distance Counselling"[tiab] OR "Elder care"[tiab] OR "Elderly nursing home"[tiab] OR "Elderly nursing homes"[tiab] OR "Family clinic"[tiab] OR "Family clinics"[tiab] OR "Family doctor"[tiab] OR "Family doctors"[tiab] OR "Family medicine group"[tiab] OR "Family medicine groups"[tiab] OR "Family medicine"[tiab] OR "Family physician"[tiab] OR "Family physicians"[tiab] OR "Family practice"[tiab] OR "Family practices"[tiab] OR "general practice"[tiab] OR "general practices"[tiab] OR "general practitioner"[tiab] OR "general practitioners"[tiab] OR "Group practice"[tiab] OR "Group practices"[tiab] OR "Health care delivery"[tiab] OR "Health care professional"[tiab] OR "Health care professionals"[tiab] OR "Health care provider"[tiab] OR "Health care providers"[tiab] OR "Health care team"[tiab] OR "Health care teams"[tiab] OR "Health personnel"[tiab] OR "Health professional"[tiab] OR "Health professionals"[tiab] OR "Health Services administration"[tiab] OR "Healthcare delivery"[tiab] OR "Healthcare professional"[tiab] OR "Healthcare professionals"[tiab] OR "Healthcare provider"[tiab] OR "Healthcare providers"[tiab] OR "Healthcare team"[tiab] OR "Healthcare teams"[tiab] OR "Home care"[tiab] OR "Home Health"[tiab] OR "Home Nurse"[tiab] OR "Home Nurses"[tiab] OR "Home Nursing"[tiab] OR "interactive consultation"[tiab] OR "interactive consultations"[tiab] OR "interactive service"[tiab] OR "interactive services"[tiab] OR "interdisciplinary team"[tiab] OR "interdisciplinary teams"[tiab] OR "Intermediate care facility"[tiab] OR "intermediate care"[tiab] OR "interprofessional team"[tiab] OR "interprofessional teams"[tiab] OR "Local community service center"[tiab] OR

"Local community service centers"[tiab] OR "Local community service centre"[tiab] OR "Local community service centres"[tiab] OR "Long-term care center"[tiab] OR "Long-term care centers"[tiab] OR "Long-term care centre"[tiab] OR "Long-term care centres"[tiab] OR "Long-term care home"[tiab] OR "Long-term care homes"[tiab] OR "multidisciplinary team"[tiab] OR "multidisciplinary teams"[tiab] OR "Multidiscipline team"[tiab] OR "multidiscipline teams"[tiab] OR "Nurse management"[tiab] OR "Nurse"[tiab] OR "Nurse-delivered"[tiab] OR "Nurse-led"[tiab] OR "Nurse-managed"[tiab] OR "Nurse-run"[tiab] OR "Nurses management"[tiab] OR "Nurses"[tiab] OR "Nurses-led"[tiab] OR "Nursing care"[tiab] OR "Nursing delivered"[tiab] OR "Nursing home"[tiab] OR "Nursing homes"[tiab] OR "Nursing management"[tiab] OR "Nursing"[tiab] OR "Nursing-delivered"[tiab] OR "Nursing-led"[tiab] OR "Nursing-managed"[tiab] OR "Nursing-run"[tiab] OR "Office nurse"[tiab] OR "Office nurses"[tiab] OR "Office nursing"[tiab] OR "Outpatient"[tiab] OR "Outpatients"[tiab] OR "Patient care team"[tiab] OR "Patient care teams" [tiab] OR "Primary care"[tiab] OR "Primary health care"[tiab] OR "Primary healthcare"[tiab] OR "Professional autonomy"[tiab] OR "Professional practice"[tiab] OR "Professional practices"[tiab] OR "Remote"[tiab] OR "Residential care center"[tiab] OR "Residential care centers"[tiab] OR "Residential care centre"[tiab] OR "Residential care centres"[tiab] OR "Residential care"[tiab] OR "Respite Care"[tiab] OR "Retirement home"[tiab] OR "Retirement homes"[tiab] OR "rural health"[tiab] OR "Rural nurse"[tiab] OR "Rural nurses"[tiab] OR "Rural nursing"[tiab] OR "Staff Nurse"[tiab] OR "Staff Nurses"[tiab] OR "Suburban Health Service"[tiab] OR "Suburban"[tiab] OR "team"[tiab] OR "teams"[tiab] OR "Teamwork"[tiab] OR "Telemedicine"[tiab] OR "tele-medicine"[tiab] OR "Telenurse"[tiab] OR "Tele-nurse"[tiab] OR "Telenurses"[tiab] OR "Tele-nurses"[tiab] OR "Telenursing"[tiab] OR "Tele-nursing"[tiab] OR "Telerehabilitation"[tiab] OR "Tele-rehabilitation"[tiab] OR "Walk-in clinic"[tiab] OR "Walk-in clinics"[tiab])) AND (("Advanced Practice Nursing"[MH] OR "Family Nurse Practitioners"[MH] OR "Nurse Practitioners"[MH]) OR ("advanced nurse practitioner"[tiab] OR "advanced nurse practitioners"[tiab] OR ("ANP"[tiab] AND "nurse"[tiab]) OR ("ANP"[tiab] AND "nurses"[tiab]) OR ("ANP"[tiab] AND "nursing"[tiab]) OR "advanced nursing practice"[tiab] OR "advanced nursing practices"[tiab] OR "advanced practice nurse"[tiab] OR "advanced practice nurses"[tiab] OR "advanced practice nursing"[tiab] OR ("APN"[tiab] AND "nurse"[tiab]) OR ("APN"[tiab] AND "nurses"[tiab]) OR ("APN"[tiab] AND "nursing"[tiab]) OR "advanced practice registered nurse"[tiab] OR "advanced practice registered nurses"[tiab] OR "advanced practice registered nursing"[tiab] OR ("APRN"[tiab] AND "nurse"[tiab]) OR ("APRN"[tiab] AND "nurses"[tiab]) OR ("APRN"[tiab] AND "nursing"[tiab]) OR "Emergency nurse practitioner"[tiab] OR "Emergency nurse practitioners"[tiab] OR ("ENP"[tiab] AND "Nurse"[tiab]) OR ("ENP"[tiab] AND "Nurses"[tiab]) OR ("ENP"[tiab] AND "Nursing"[tiab]) OR "Family nurse practitioner"[tiab] OR "Family nurse practitioners"[tiab] OR ("FNP"[tiab] AND "Nurse"[tiab]) OR ("FNP"[tiab] AND "Nurses"[tiab]) OR ("FNP"[tiab] AND "Nursing"[tiab]) OR "Nurse practitioner"[tiab] OR "Nurse practitioners"[tiab] OR ("NP"[tiab] AND "nurse"[tiab]) OR ("NP"[tiab] AND "nurses"[tiab]) OR ("NP"[tiab] AND "nursing"[tiab]) OR "Primary care nurse practitioner"[tiab] OR "Primary care nurse practitioners"[tiab] OR "Primary health care nurse practitioner"[tiab] OR ("PCNP"[tiab] AND "Nurse"[tiab]) OR ("PCNP"[tiab] AND "Nurses"[tiab]) OR ("PCNP"[tiab] AND "Nursing"[tiab]) OR "Primary health care nurse practitioners"[tiab] OR "Primary healthcare nurse practitioner"[tiab] OR "Primary health care nurse practitioner"[tiab] OR "Primary healthcare nurse practitioners"[tiab] OR "Primary health care nurse practitioners"[tiab] OR ("PHCNP"[tiab] AND "Nurse"[tiab]) OR ("PHCNP"[tiab] AND "Nurses"[tiab]) OR ("PHCNP"[tiab] AND "Nursing"[tiab])))) AND ((((((((((("Appointments and Schedules"[MH] OR "Biomarkers"[Mesh:NoExp] OR "Hypertension"[Mesh:NoExp] OR "Cholesterol"[MH] OR "Hypercholesterolemia"[MH] OR "Chronic Disease"[MH] OR "Consumer Behavior"[MH] OR "Costs and Cost Analysis"[MH] OR "Delivery of Health Care"[MH] OR "Diagnostic Tests, Routine"[MH] OR "Glycated Hemoglobin A"[MH] OR "Guideline Adherence"[MH] OR "Health Resources"[MH] OR "Health Services"[MH] OR "Health Status Indicators"[MH] OR "Health status"[MH] OR "Job Satisfaction"[MH] OR "Mortality"[MH] OR "Outcome and Process Assessment, Health Care"[MH] OR "Outcome Assessment, Health Care"[MH] OR "Pain"[Mesh:NoExp] OR "Patient Satisfaction"[MH] OR "Personal Satisfaction"[MH] OR "Prescriptions"[MH] OR "Professional Role"[MH] OR "Quality of Health Care"[MH] OR "Referral and

Consultation"[MH] OR "Task Performance and Analysis"[MH] OR "Treatment Adherence and Compliance"[MH]) OR ("Appointment"[tiab] OR "Appointments"[tiab] OR "Attendance"[tiab] OR "Biomarker"[tiab] OR "Biomarkers"[tiab] OR "Hypertension"[tiab] OR "Care Process"[tiab] OR "Care Processes"[tiab] OR "Cholesterol"[tiab] OR "Hypercholesterolemia"[tiab] OR "Chronic condition"[tiab] OR "Chronic conditions"[tiab] OR "Chronic disease"[tiab] OR "Chronic diseases"[tiab] OR "Chronic illness"[tiab] OR "Chronic illnesses"[tiab] OR "clinical outcome"[tiab] OR "clinical outcomes"[tiab] OR "clinical parameter"[tiab] OR "clinical parameters"[tiab] OR "Compliance"[tiab] OR "Compliant"[tiab] OR "Consultation"[tiab] OR "Consultations"[tiab] OR "Consumer Behavior"[tiab] OR "Consumer Behaviors"[tiab] OR "Consumer Behaviour"[tiab] OR "Consumer Behaviours"[tiab] OR "Cost analyses"[tiab] OR "Cost analysis"[tiab] OR "Cost"[tiab] OR "Costs"[tiab] OR "Death"[tiab] OR "Deaths"[tiab] OR "Delivery of health care"[tiab] OR "Delivery of healthcare"[tiab] OR "Diagnostic Test"[tiab] OR "Diagnostic Testing"[tiab] OR "Diagnostic Tests"[tiab] OR "economic analyses"[tiab] OR "economic analysis"[tiab] OR "economic evaluation"[tiab] OR "economic evaluations"[tiab] OR "Emergency Service"[tiab] OR "Emergency Services"[tiab] OR "Follow up appointment"[tiab] OR "Follow up appointments"[tiab] OR "follow up visit"[tiab] OR "follow up visits"[tiab] OR "Glycated Haemoglobin A"[tiab] OR "Glycated Hemoglobin A"[tiab] OR "Guideline Adherence"[tiab] OR "HbA1C"[tiab] OR "hdl"[tiab] OR "health care"[tiab] OR "Health Planning"[tiab] OR "Health related quality of life"[tiab] OR "Health Resource"[tiab] OR "Health Resources"[tiab] OR "Health Service"[tiab] OR "Health Services"[tiab] OR "Health status"[tiab] OR "Healthcare delivery"[tiab] OR "Healthcare process assessment"[tiab] OR "Healthcare Quality"[tiab] OR "healthcare"[tiab] OR "high density lipoprotein"[tiab] OR "hospital admission"[tiab] OR "hospital admissions"[tiab] OR "hospital referral"[tiab] OR "hospital referrals"[tiab] OR "HRQOL"[tiab] OR "Hypertension"[tiab] OR "indicator"[tiab] OR "indicators"[tiab] OR "Job Satisfaction"[tiab] OR "ldl"[tiab] OR "Lipid profile"[tiab] OR "Lipid profiles"[tiab] OR "low density lipoprotein"[tiab] OR "Manpower"[tiab] OR "Mortalities"[tiab] OR "Mortality"[tiab] OR "Outcome Assessment"[tiab] OR "Outcome Assessments"[tiab] OR "Pain"[tiab] OR "Pains"[tiab] OR "Patient Satisfaction"[tiab] OR "Personal Satisfaction"[tiab] OR "prefer"[tiab] OR "preference"[tiab] OR "preferences"[tiab] OR "preferred"[tiab] OR "Prescribe"[tiab] OR "Prescribed"[tiab] OR "prescription"[tiab] OR "prescriptions"[tiab] OR "Process of care"[tiab] OR "Processes of care"[tiab] OR "Professional Role"[tiab] OR "Professional Roles"[tiab] OR "QOL"[tiab] OR "quality indicator"[tiab] OR "quality indicators"[tiab] OR "Quality of Health Care"[tiab] OR "Quality of Healthcare"[tiab] OR "Quality of life"[tiab] OR "Referral"[tiab] OR "Referrals"[tiab] OR "return appointment"[tiab] OR "return appointments"[tiab] OR "return visit"[tiab] OR "return visits"[tiab] OR "Routine diagnostic"[tiab] OR "Routine test"[tiab] OR "Routine testing"[tiab] OR "Routine tests"[tiab] OR "satisfaction"[tiab] OR "satisfactory"[tiab] OR "satisfied"[tiab] OR "Schedule"[tiab] OR "Schedules"[tiab] OR "Task Analyses"[tiab] OR "Task Analysis"[tiab] OR "Task Performance"[tiab] OR "Treatment Adherence"[tiab] OR "treatment adherent"[tiab] OR "Treatment Compliance"[tiab] OR "Treatment compliant"[tiab] OR "Wellness"[tiab])) OR ("Anthropometry"[MH] OR "Body Weight"[MH] OR "Culturally Competent Care"[MH] OR "diet"[Mesh:NoExp] OR "Diet, Food, and Nutrition"[MH] OR "Exercise"[Mesh:NoExp] OR "Health Education"[MH] OR "Health Promotion"[MH] OR "Healthy Lifestyle"[MH] OR "Mental Health"[MH] OR "Nutrition Assessment"[MH] OR "Nutritional Status"[MH] OR "poverty"[MH] OR "Preventive Health Services"[MH] OR "Smoking Cessation"[MH] OR "Smoking Prevention"[MH] OR "Smoking"[MH] OR "Teaching"[MH] OR "Tobacco Smoke Pollution"[MH] OR "Tobacco Use"[MH])) OR ("anthropometrical"[tiab] OR "anthropometrically"[tiab] OR "anthropometrics"[tiab] OR "Anthropometry"[tiab] OR "Body Weight"[tiab] OR "Condition specific"[tiab] OR "Culturally Competent Care"[tiab] OR "culturally diverse"[tiab] OR "Diet"[tiab] OR "Dietary"[tiab] OR "Dietetical"[tiab] OR "Dietetically"[tiab] OR "Dietetics"[tiab] OR "Diets"[tiab] OR "emotional health"[tiab] OR "Exercise"[tiab] OR "Food regimen"[tiab] OR "Food regimens"[tiab] OR "Health Education"[tiab] OR "Health prevention"[tiab] OR "Health Promotion"[tiab] OR "healthy habit"[tiab] OR "healthy habits"[tiab] OR "Healthy life"[tiab] OR "Healthy life habits"[tiab] OR "healthy living"[tiab] OR "Life style"[tiab] OR "Lifestyle"[tiab] OR "low income"[tiab] OR "Mental Health"[tiab] OR "Nutrition Assessment"[tiab] OR "Nutrition"[tiab] OR "Nutritional Status"[tiab] OR

"nutritional"[tiab] OR "physical activity"[tiab] OR "poverty"[tiab] OR "Preventive Health Service"[tiab] OR "Preventive Health Services"[tiab] OR "promotion of health"[tiab] OR "Promotion"[tiab] OR "Quit smoking"[tiab] OR "Quitting smoking"[tiab] OR "second hand smoke"[tiab] OR "Smoke"[tiab] OR "Smoked"[tiab] OR "Smoker"[tiab] OR "Smokers"[tiab] OR "Smokes"[tiab] OR "Smoking"[tiab] OR "socioeconomic status"[tiab] OR "Stop smoking"[tiab] OR "Stopping smoking"[tiab] OR "stress level"[tiab] OR "stress levels"[tiab] OR "Taught"[tiab] OR "Teach"[tiab] OR "Teaching"[tiab] OR "Tobacco Use"[tiab] OR "Weight"[tiab])) OR ("Adolescent"[MH] OR "Child Development"[MH] OR "Human Development"[MH] OR "Immunization"[Mesh:NoExp] OR "Maternal Health Services"[MH] OR "Pediatrics"[MH] OR "Primary Prevention"[MH] OR "Secondary Prevention"[MH] OR "Sexually Transmitted Diseases"[Mesh:NoExp] OR "Vaccination"[MH])) OR ("adolescence"[tiab] OR "adolescent"[tiab] OR "adolescents"[tiab] OR "Child development"[tiab] OR "Childhood"[tiab] OR "healthy development"[tiab] OR "Human development"[tiab] OR "Immunisation"[tiab] OR "Immunise"[tiab] OR "Immunised"[tiab] OR "Immunises"[tiab] OR "Immunization"[tiab] OR "Immunize"[tiab] OR "Immunized"[tiab] OR "Immunizes"[tiab] OR "laboratory test"[tiab] OR "Maternal Health Service"[tiab] OR "Maternal Health Services"[tiab] OR "Paediatric"[tiab] OR "Paediatrics"[tiab] OR "Pediatric"[tiab] OR "Pediatrics"[tiab] OR "pregnancy"[tiab] OR "Prevent"[tiab] OR "Prevented"[tiab] OR "Prevention"[tiab] OR "Prevents"[tiab] OR "Primary Prevention"[tiab] OR "Secondary Prevention"[tiab] OR "sexually transmitted"[tiab] OR "Vaccinate"[tiab] OR "Vaccinated"[tiab] OR "Vaccinates"[tiab] OR "Vaccination"[tiab] OR "Vaccine"[tiab] OR "Vaccines"[tiab] OR "youth"[tiab] OR "youths"[tiab])) OR ("Acute Coronary Syndrome"[Mesh:NoExp] OR "Adjustment Disorders"[MH] OR "Alcohol Drinking"[MH] OR "Alcohol-Related Disorders"[MH] OR "Anxiety Disorders"[MH] OR "Blood Glucose"[MH] OR "Hyperglycemia"[MH] OR "Hypoglycemia"[MH] OR "Cardiovascular Diseases"[Mesh:NoExp] OR "Coronary Disease"[Mesh:NoExp] OR "Dementia"[Mesh:NoExp] OR "Depressive Disorder"[MH] OR "Diabetes Mellitus"[Mesh:NoExp] OR "diabetes, gestational"[MH] OR "Diabetic Foot"[MH] OR "Health Care Evaluation Mechanisms"[MH] OR "Health Information Interoperability"[MH] OR "Heart Failure"[Mesh:NoExp] OR "Myocardial Infarction"[MH] OR "Mental Disorders"[MH] OR "Neoplasms"[Mesh:NoExp] OR "pain management"[MH] OR "Pain"[Mesh:NoExp] OR "Papanicolaou Test"[MH] OR "Patient Reported Outcome Measures"[MH] OR "Physical Examination"[MH] OR "Pulmonary Disease, Chronic Obstructive"[MH] OR "Quality Indicators, Health Care"[MH] OR "Rehabilitation"[MH] OR "Self care"[MH] OR "Self Efficacy"[MH] OR "Stroke"[Mesh:NoExp])) OR ("ACS"[tiab] OR "ACSs"[tiab] OR "Acute Coronary Syndrome"[tiab] OR "Acute Coronary Syndromes"[tiab] OR "Adjustment Disorder"[tiab] OR "Adjustment Disorders"[tiab] OR "Alcohol consumption"[tiab] OR "Alcohol Drinking"[tiab] OR "Alcohol Related Disorder"[tiab] OR "Alcohol Related Disorders"[tiab] OR "Alcoholic"[tiab] OR "Alcoholism"[tiab] OR "Anxiety"[tiab] OR "anxious"[tiab] OR "Blood Glucose"[tiab] OR "Blood sugar"[tiab] OR "Blood sugars"[tiab] OR "Hyperglycemia"[tiab] OR "Hypoglycemia"[tiab] OR "Cancer"[tiab] OR "Cardiovascular disease"[tiab] OR "Cardiovascular diseases"[tiab] OR "cerebrovascular accident"[tiab] OR "cerebrovascular accidents"[tiab] OR "CHF"[tiab] OR "Chronic Obstructive Pulmonary Disease"[tiab] OR "Chronic Obstructive Pulmonary Disorder"[tiab] OR "Chronic Obstructive Pulmonary Disorders"[tiab] OR "complication"[tiab] OR "complications"[tiab] OR "Congestive heart failure"[tiab] OR "copd"[tiab] OR "Coronary artery disease"[tiab] OR "Coronary artery diseases"[tiab] OR "Coronary Disease"[tiab] OR "Coronary Diseases"[tiab] OR "CVA"[tiab] OR "CVAs"[tiab] OR "CVD"[tiab] OR "CVDs"[tiab] OR "Dementia"[tiab] OR "Dementias"[tiab] OR "depressed"[tiab] OR "depression"[tiab] OR "Depressive Disorder"[tiab] OR "Depressive Disorders"[tiab] OR "depressive"[tiab] OR "Diabetes Mellitus"[tiab] OR "Diabetes"[tiab] OR "Diabetic Foot"[tiab] OR "foot complication"[tiab] OR "foot complications"[tiab] OR "gestational diabetes"[tiab] OR "glycaemia"[tiab] OR "glycaemic"[tiab] OR "glycemia"[tiab] OR "glycemic"[tiab] OR "Health Care Evaluation Mechanisms"[tiab] OR "Health Care Quality Indicator"[tiab] OR "Health Care Quality Indicators"[tiab] OR "Health Information Interoperability"[tiab] OR "HealthCare Evaluation Mechanisms"[tiab] OR "Healthcare Quality Indicator"[tiab] OR "Healthcare Quality Indicators"[tiab] OR "Heart Failure"[tiab] OR "Interoperability"[tiab] OR "Interoperable"[tiab] OR "managed pain"[tiab] OR "management of pain"[tiab] OR "managing pain"[tiab] OR "Mental Disorder"[tiab] OR "Mental

Disorders"[tiab] OR "mental health"[tiab] OR "Mental illness"[tiab] OR "Mental illnesses"[tiab] OR "mentally healthy"[tiab] OR "Mentally ill"[tiab] OR "Myocardial Infarction"[tiab] OR "Neoplasm"[tiab] OR "Neoplasms"[tiab] OR "Oncologist"[tiab] OR "Oncologists"[tiab] OR "Oncology"[tiab] OR "pain management"[tiab] OR "pain"[tiab] OR "pains"[tiab] OR "Pap test"[tiab] OR "Pap tests"[tiab] OR "Papanicolaou Test"[tiab] OR "Papanicolaou Tests"[tiab] OR "Patient Reported Experience Measure"[tiab] OR "Patient Reported Experience Measures"[tiab] OR "Patient Reported Outcome Measure"[tiab] OR "Patient Reported Outcome Measures"[tiab] OR "Physical Exam"[tiab] OR "Physical Examination"[tiab] OR "Physical Examinations"[tiab] OR "Physical Exams"[tiab] OR "PREM"[tiab] OR "PREMs"[tiab] OR "PROM"[tiab] OR "promotion"[tiab] OR "PROMs"[tiab] OR "psycho social"[tiab] OR "psychosocial"[tiab] OR "Quality Indicator"[tiab] OR "Quality indicators of health care"[tiab] OR "Quality Indicators"[tiab] OR "Rehabilitation"[tiab] OR "self care"[tiab] OR "Self Efficacy"[tiab] OR "Stroke"[tiab] OR "Strokes"[tiab])) OR ("Allied Health Personnel"[MH] OR "Ambulatory Care Information Systems"[MH] OR "Ambulatory Care"[Mesh:NoExp] OR "Appointments and Schedules"[MH] OR "Clinical Competence"[MH] OR "Duration of Therapy"[MH] OR "Patient Education as Topic"[MH] OR "Evidence-Based Practice"[MH] OR "Harm Reduction"[MH] OR "Health Behavior"[MH] OR "Health Care Costs"[MH] OR "Health Care Economics and Organizations"[MH] OR "Health Care Facilities, Manpower, and Services"[MH] OR "Health Care Quality, Access, and Evaluation"[MH] OR "Health Knowledge, Attitudes, Practice"[MH] OR "Health Plan Implementation"[MH] OR "Health Services Accessibility"[MH] OR "Healthcare Disparities"[MH] OR "Hospital Shared Services"[MH] OR "House Calls"[MH] OR "Length of Stay"[MH] OR "Office Visits"[MH] OR "Patient Acceptance of Health Care"[MH] OR "patient admission"[MH] OR "Patient Advocacy"[MH] OR "Prescriptions"[MH] OR "Referral and Consultation"[MH] OR "Universal Health Care"[MH])) OR ("Access to care"[tiab] OR "Access to health care"[tiab] OR "Access to Health Services"[tiab] OR "Access to healthcare"[tiab] OR "Accessibility of Health Services"[tiab] OR "Allied Health Personnel"[tiab] OR "Ambulatory Care Information System"[tiab] OR "Ambulatory Care Information Systems"[tiab] OR "ambulatory care"[tiab] OR "Appointment"[tiab] OR "Appointments"[tiab] OR "care access"[tiab] OR "Clinical Competence"[tiab] OR "Clinical Competencies"[tiab] OR "Clinical Competency"[tiab] OR "Consultation"[tiab] OR "Consultations"[tiab] OR "Cost"[tiab] OR "Costs"[tiab] OR "Duration of Therapy"[tiab] OR "Patient Education as Topic"[tiab] OR "Patient teaching"[tiab] OR "Evaluated Health Care"[tiab] OR "Evaluated HealthCare"[tiab] OR "Evaluating Health Care"[tiab] OR "Evaluating HealthCare"[tiab] OR "Evaluation of Health Care"[tiab] OR "Evaluations of Health Care"[tiab] OR "Harm prevention"[tiab] OR "Harm Reduction"[tiab] OR "Health Attitude"[tiab] OR "Health Attitudes"[tiab] OR "Health Behavior"[tiab] OR "Health Behaviors"[tiab] OR "Health Behaviour"[tiab] OR "Health Behaviours"[tiab] OR "HealthCare"[tiab] OR "Health Care"[tiab] OR "Health Knowledge"[tiab] OR "Health Personnel"[tiab] OR "Health Plan Implementation"[tiab] OR "Health Service Accessibility"[tiab] OR "Health Services Accessibility"[tiab] OR "healthy environment"[tiab] OR "healthy environments"[tiab] OR "Hospital Shared Services"[tiab] OR "House Call"[tiab] OR "House Calls"[tiab] OR "indirect cost"[tiab] OR "indirect costs"[tiab] OR "Knowledge of Health"[tiab] OR "Length of Stay"[tiab] OR "Length of therapy"[tiab] OR "Lengths of Stay"[tiab] OR "LOS"[tiab] OR "Office Visit"[tiab] OR "Office Visits"[tiab] OR "Patient Acceptance"[tiab] OR "patient admission"[tiab] OR "patient admissions"[tiab] OR "Patient Advocacy"[tiab] OR "Prescription"[tiab] OR "Prescriptions"[tiab] OR "Prevention of harm"[tiab] OR "Quality Health Care"[tiab] OR "Quality HealthCare"[tiab] OR "Reduction of harm"[tiab] OR "Referral"[tiab] OR "Referrals"[tiab] OR "return visit"[tiab] OR "return visits"[tiab] OR "Schedule"[tiab] OR "Scheduled"[tiab] OR "Schedules"[tiab] OR "Therapy duration"[tiab] OR "Therapy durations"[tiab] OR "Therapy length"[tiab] OR "Universal Health Care"[tiab] OR "Universal HealthCare"[tiab] OR "unplanned"[tiab])) OR ("Delivery of Health Care"[MH] OR "Frailty"[MH] OR "Global Health"[MH] OR "Health Equity"[MH] OR "Health Literacy"[MH] OR "Health Status Disparities"[MH] OR "Healthcare Disparities"[MH] OR "Income"[MH] OR "Insurance Coverage"[MH] OR "International Classification of Functioning, Disability and Health"[MH] OR "Marital Status"[MH] OR "Minority Health"[MH] OR "Patient Selection"[MH] OR "Population Dynamics"[MH] OR "Population Groups"[MH] OR "Poverty Areas"[MH] OR "Refusal to Treat"[MH] OR

"Religion"[Mesh:NoExp] OR "Social Discrimination"[MH] OR "Socioeconomic Factors"[MH] OR "Treatment Failure"[MH])) OR ("Aboriginal"[tiab] OR "Communication Barriers"[tiab] OR "Delivery of Health Care"[tiab] OR "Delivery of HealthCare"[tiab] OR "Disability Evaluation"[tiab] OR "Discrimination"[tiab] OR "equity"[tiab] OR "Frailty"[tiab] OR "Gender Minorities"[tiab] OR "Gender Minority"[tiab] OR "Global Health"[tiab] OR "Health Care Delivery"[tiab] OR "Health care Disparities"[tiab] "Health Care Disparity"[tiab] OR "Health Equity"[tiab] OR "Health Literacy"[tiab] OR "Health Status Disparities"[tiab] OR "Health Status Disparity"[tiab] OR "HealthCare Delivery"[tiab] OR "Healthcare Disparities"[tiab] OR "Healthcare Disparity"[tiab] OR "Income"[tiab] OR "Incomes"[tiab] OR "inequal"[tiab] OR "inequalities"[tiab] OR "inequality"[tiab] OR "Insurance cover"[tiab] OR "Insurance coverage"[tiab] OR "Insurance coverages"[tiab] OR "Insurance covered"[tiab] OR "International Classification of Functioning, Disability and Health"[tiab] OR "Marital status"[tiab] OR "Minority Health"[tiab] OR "Oceanic Ancestry Group"[tiab] OR "Oceanic Ancestry Groups"[tiab] OR "Patient selection"[tiab] OR "population dynamics"[tiab] OR "Population group"[tiab] OR "Population groups"[tiab] OR "Poverty area"[tiab] OR "Poverty areas"[tiab] OR "Poverty"[tiab] OR "Refusal to Treat"[tiab] OR "Religion"[tiab] OR "Religions"[tiab] OR "safety net"[tiab] OR "Sexual Minorities"[tiab] OR "Sexual Minority"[tiab] OR "Sexual Orientation"[tiab] OR "Sexual Orientations"[tiab] OR "Social Discrimination"[tiab] OR "Social Discriminations"[tiab] OR "Social support"[tiab] OR "treatment failure"[tiab] OR "treatment failures"[tiab] OR "Treatment preference"[tiab] OR "Treatment preferences"[tiab] OR "Vulnerabilities"[tiab] OR "Vulnerability"[tiab] OR "Vulnerable"[tiab]))) AND ((systematic[sb] or meta-analysis[pt] or meta-analysis as topic[mh] or meta-analysis[mh] or meta analy*[tw] or metanaly*[tw] or metaanaly*[tw] or met analy*[tw] or research overview*[tiab] or systematic review*[tiab]) or ((overview*[ti] or review[ti] or synthesis[ti] or summary[ti] or cochrane[ti] or analysis[ti]) and (reviews[ti] or meta-analyses[ti] or articles[ti])) or (meta-review[tiab] or meta review[tiab]) or ((overview*[ti] or reviews[ti]) and (systematic[ti] or cochrane[ti])) or (reviews[tiab] and (meta [tiab] or published[tiab] or quality[tiab] or included[tiab] or summar*[tiab])) or ("cochrane reviews"[tiab]) or (evidence[ti] and (reviews[ti] or meta-analyses[ti])))) AND (("2010/01/01"[Date - Publication] : "2022/12/02"[Date - Publication]))

2. Ovid Medline

1. exp Ambulatory Care Facilities/ or Ambulatory Care/ or exp Community Health Centers/ or exp Community Health Nursing/ or exp Community Health Planning/ or exp Community Health services/ or exp Community medicine/ or Community Mental Health Services/ or exp Delivery of Health Care/ or exp family practice/ or exp General Practice/ or exp General Practitioners/ or exp group practice/ or exp Health Personnel/ or exp Health Services Administration/ or exp Home Care Services/ or exp Home Nursing/ or exp Homes for the Aged/ or exp Housing for the Elderly/ or exp Intermediate Care Facilities/ or exp Long-Term Care/ or exp Nurses, Community Health/ or exp Nursing Care/ or exp Nursing Homes/ or exp Nursing Stations/ or exp Nursing/ or exp Nursing, team/ or exp Office Nursing/ or exp outpatients/ or exp Patient Care Team/ or exp Physicians, Family/ or exp Physicians, Primary Care/ or exp Primary Care Nursing/ or exp Primary Health Care/ or exp Professional Autonomy/ or exp Professional Practice/ or exp Remote Consultation/ or exp Respite Care/ or exp Rural Health Services/ or exp Rural Health/ or exp Rural Nursing/ or exp Suburban Health Services/ or Telemedicine/ or exp Telenursing/ or exp Telerehabilitation/

2. (ambulatory care or Care Continuity or Chsld or Chslds or Clsc or Clsc or Community Care or Community Clinic or Community Clinics or Community Doctor or Community Doctors or Community Facilities or Community Facility or Community Health Center or Community Health Centers or Community Health Centre or Community Health Centres or Community Health Nurse or Community Health Nurses or Community Health Nursing or Community Health Plan or Community Health Planning or Community Health Plans or Community Health Service or Community Health Services or Community Medicine or Community Mental Health Service or Community Mental Health Services or Community Nurse or Community Nurses or Community Nursing or Community Physician or Community Physicians or Community Practice or Community Practices or Community Program or Community Programs or Community Service or Community Services or Continuity of Care or Counseling or Counselling or Delivery of Health Care or Delivery of Healthcare or Distance Counseling or Distance Counselling or Elder Care or Elderly Nursing Home or Elderly Nursing Homes or Family Clinic or Family Clinics or Family Doctor or Family Doctors or Family Medicine Group or Family Medicine Groups or Family Medicine or Family Physician or Family Physicians or Family Practice or Family Practices or General Practice or General Practices or General Practitioner or General Practitioners or Group Practice or Group Practices or Health Care Delivery or Health Care Professional or Health Care Professionals or Health Care Provider or Health Care Providers or Health Care Team or Health Care Teams or Health Personnel or Health Professional or Health Professionals or Health Services Administration or Healthcare Delivery or Healthcare Professional or Healthcare Professionals or Healthcare Provider or Healthcare Providers or Healthcare Team or Healthcare Teams or Home Care or Home Health or Home Nurse or Home Nurses or Home Nursing or Interactive Consultation or Interactive Consultations or Interactive Service or Interactive Services or Interdisciplinary Team or Interdisciplinary Teams or Intermediate Care Facility or Intermediate Care or Interprofessional Team or Interprofessional Teams or Local Community Service Center or Local Community Service Centers or Local Community Service Centre or Local Community Service Centres or Long-Term Care Center or Long-Term Care Centers or Long-Term Care Centre or Long-Term Care Centres or Long-Term Care Home or Long-Term Care Homes or Multidisciplinary Team or Multidisciplinary Teams or Multidiscipline Team or Multidiscipline Teams or Nurse Management or Nurse or Nurse-Delivered or Nurse-Led or Nurse-Managed or Nurse-Run or Nurses Management or Nurses or Nurses-Led or Nursing Care or Nursing Delivered or Nursing Home or Nursing Homes or Nursing Management or Nursing or Nursing-Delivered or Nursing-Led or Nursing-Managed or Nursing-Run or Office Nurse or Office Nurses or Office Nursing or Outpatient or Outpatients or Patient Care Team or Patient Care Teams or Primary Care or Primary Health Care or Primary Healthcare or Professional Autonomy or Professional Practice or Professional Practices or Remote or Residential Care Center or Residential Care Centers or Residential Care Centre or Residential Care Centres or Residential Care or Respite Care or Retirement Home or Retirement Homes or Rural Health or Rural Nurse or Rural Nurses or Rural Nursing or Staff Nurse or Staff Nurses or Suburban Health Service or Suburban or Team or

Teams or Teamwork or Telemedicine or Tele-Medicine or Telenurse or Tele-Nurse or Telenurses or Tele-Nurses or Telenursing or Tele-Nursing or Telerehabilitation or Tele-Rehabilitation or Walk-In Clinic or Walk-In Clinics).ab,ti.

3. 1 or 2

4. exp Advanced Practice Nursing/ or exp Family Nurse Practitioners/ or exp Nurse Practitioners/

5. (Advanced Nurse Practitioner or Advanced Nurse Practitioners or (ANP and Nurse) or (ANP and Nurses) or (ANP and Nursing) or Advanced Nursing Practice or Advanced Nursing Practices or Advanced Practice Nurse or Advanced Practice Nurses or Advanced Practice Nursing or (APN and Nurse) or (APN and Nurses) or (APN and Nursing) or Advanced Practice Registered Nurse or Advanced Practice Registered Nurses or Advanced Practice Registered Nursing or (APRN and Nurse) or (APRN and Nurses) or (APRN and Nursing) or Emergency Nurse Practitioner or Emergency Nurse Practitioners or (APRN and Nurse) or (APRN and Nurses) or (APRN and Nursing) or Family nurse practitioner or Family nurse practitioners or (FNP and Nurse) or (FNP and Nurses) or (FNP and Nursing) or Nurse Practitioner or Nurse Practitioners or (NP and Nurse) or (NP and Nurses) or (NP and Nursing) or Primary Care Nurse Practitioner or Primary Care Nurse Practitioners or Primary Health Care Nurse Practitioner or (PCNP and Nurse) or (PCNP and Nurses) or (PCNP and Nursing) or Primary Health Care Nurse Practitioners or Primary Healthcare Nurse Practitioner or Primary Health Care Nurse Practitioner or Primary Healthcare Nurse Practitioners or Primary Health care Nurse Practitioners or (PHCNP and Nurse) or (PHCNP and Nurses) or (PHCNP and Nursing)).ab,ti.

6. 4 or 5

7. exp "Appointments and Schedules"/ or Biomarkers/ or Hypertension/ or exp Cholesterol/ or exp Hypercholesterolemia/ or exp Chronic Disease/ or exp Consumer Behavior/ or exp "Costs and Cost Analysis"/ or exp Delivery of Health Care/ or exp Diagnostic Tests, Routine/ or exp Glycated Hemoglobin A/ or exp Guideline Adherence/ or exp Health Resources/ or exp Health Services/ or exp Health Status Indicators/ or exp Health Status/ or exp Job Satisfaction/ or exp Mortality/ or exp "Outcome and Process Assessment, Health Care"/ or exp Outcome Assessment, Health Care/ or Pain/ or exp Patient Satisfaction/ or exp Personal Satisfaction/ or exp Prescriptions/ or exp Professional Role/ or exp Quality of Health Care/ or exp "Referral and Consultation"/ or exp "Task Performance and Analysis"/ or exp "Treatment Adherence and Compliance"/

8. (Appointment or Appointments or Attendance or Biomarker or Biomarkers or Hypertension or Care Process or Care Processes or Cholesterol or Hypercholesterolemia or Chronic Condition or Chronic Conditions or Chronic Disease or Chronic Diseases or Chronic Illness or Chronic Illnesses or Clinical Outcome or Clinical Outcomes or Clinical Parameter or Clinical Parameters or Compliance or Compliant or Consultation or Consultations or Consumer Behavior or Consumer Behaviors or Consumer Behaviour or Consumer Behaviours or Cost Analyses or Cost Analysis or Cost or Costs or Death or Deaths or Delivery Of Health Care or Delivery Of Healthcare or Diagnostic Test or Diagnostic Testing or Diagnostic Tests or Economic Analyses or Economic Analysis or Economic Evaluation or Economic Evaluations or Emergency Service or Emergency Services or Follow Up Appointment or Follow Up Appointments or Follow Up Visit or Follow Up Visits or Glycated Haemoglobin A or Glycated Hemoglobin A or Guideline Adherence or HbA1C or Hdl or Health Care or Health Planning or Health Related Quality Of Life or Health Resource or Health Resources or Health Service or Health Services or Health Status or Healthcare Delivery or Healthcare Process Assessment or Healthcare Quality or Healthcare or High Density Lipoprotein or Hospital Admission or Hospital Admissions or Hospital Referral or Hospital Referrals or Hrqol or Hypertension or Indicator or Indicators or Job Satisfaction or Ldl or Lipid Profile or Lipid Profiles or Low Density Lipoprotein or Manpower or Mortalities or Mortality or Outcome Assessment or Outcome Assessments or Pain or Pains or Patient Satisfaction or Personal Satisfaction or Prefer or Preference or Preferences or

Preferred or Prescribe or Prescribed or Prescription or Prescriptions or Process Of Care or Processes Of Care or Professional Role or Professional Roles or Qol or Quality Indicator or Quality Indicators or Quality Of Health Care or Quality Of Healthcare or Quality Of Life or Referral or Referrals or Return Appointment or Return Appointments or Return Visit or Return Visits or Routine Diagnostic or Routine Test or Routine Testing or Routine Tests or Satisfaction or Satisfactory or Satisfied or Schedule or Schedules or Task Analyses or Task Analysis or Task Performance or Treatment Adherence or Treatment Adherent or Treatment Compliance or Treatment Compliant or Wellness).ab,ti.

9. 7 or 8

10. exp Anthropometry/ or exp Body Weight/ or exp Culturally Competent Care/ or Diet/ or exp "Diet, Food, and Nutrition"/ or Exercise/ or exp Health Education/ or exp Health Promotion/ or exp Healthy Lifestyle/ or exp Mental Health/ or exp Nutrition Assessment/ or exp Nutritional Status/ or exp Poverty/ or exp Preventive Health Services/ or exp Smoking Cessation/ or exp Smoking Prevention/ or exp Smoking/ or exp Teaching/ or exp Tobacco Smoke Pollution/

11. (Anthropometric or Anthropometrical or Anthropometrically or Anthropometrics or Anthropometry or Body Weight or Condition Specific or Culturally Competent Care or Culturally Diverse or Diet or Dietary or Dietetical or Dietetically or Dietetics or Diets or Emotional Health or Exercise or Food Regimen or Food Regimens or Health Education or Health Prevention or Health Promotion or Healthy Habit or Healthy Habits or Healthy Life or Healthy Life Habits or Healthy Living or Life Style or Lifestyle or Low Income or Mental Health or Nutrition Assessment or Nutrition or Nutritional Status or Nutritional or Physical Activity or Poverty or Preventive Health Service or Preventive Health Services or Promotion of Health or Promotion or Quit Smoking or Quitting Smoking or Second Hand Smoke or Smoke or Smoked or Smoker or Smokers or Smokes or Smoking or Socioeconomic Status or Stop Smoking or Stopping Smoking or Stress Level or Stress Levels or Taught or Teach or Teaching or Tobacco or Weight).ab,ti.

12. 10 or 11

13. exp Adolescent/ or exp Child Development/ or exp Human Development/ or exp Immunization/ or exp Maternal Health Services/ or exp Pediatrics/ or exp Primary Prevention/ or exp Secondary Prevention/ or Sexually Transmitted Diseases/ or exp Vaccination/

14. (Adolescence or Adolescent or Adolescents or Child Development or Childhood or Healthy Development or Human Development or Immunisation or Immunise or Immunised or Immunises or Immunization or Immunize or Immunized or Immunizes or Laboratory Test or Maternal Health Service or Maternal Health Services or Paediatric or Paediatrics or Pediatric or Pediatrics or Pregnancy or Prevent or Prevented or Prevention or Prevents or Primary Prevention or Secondary Prevention or Sexually Transmitted or Vaccinate or Vaccinated or Vaccinates or Vaccination or Vaccine or Vaccines or Youth or Youths).ab,ti.

15. 13 or 14

16. Acute Coronary Syndrome/ or exp Adjustment Disorders/ or exp Alcohol Drinking/ or exp Alcohol-Related Disorders/ or exp Anxiety Disorders/ or exp Blood Glucose/ or exp Hyperglycemia/ or exp Hypoglycemia/ or Cardiovascular Diseases/ or Dementia/ or exp Depressive Disorder/ or Diabetes Mellitus/ or exp Diabetes, Gestational/ or Diabetic Foot/ or exp Health Information Interoperability/ or heart failure/ or exp Myocardial Infarction/ or exp Mental Disorders/ or Neoplasms/ or exp Pain Management/ or Pain/ or exp Papanicolaou Test/ or exp Patient Reported Outcome Measures/ or exp Physical Examination/ or exp Pulmonary Disease, Chronic Obstructive/ or exp Quality Indicators, Health Care/ or exp Rehabilitation/ or exp Self Care/ or exp Self Efficacy/ or Stroke/

17. (Acs or Acss or Acute Coronary Syndrome or Acute Coronary Syndromes or Adjustment Disorder or Adjustment Disorders or Alcohol Consumption or Alcohol Drinking or Alcohol Related Disorder or Alcohol Related Disorders or Alcoholic or Alcoholism or Anxiety or Anxious or Blood Glucose or Blood Sugar or Blood Sugars or Hyperglycemia or Hypoglycemia or Cancer or Cardiovascular Disease or Cardiovascular Diseases or Cerebrovascular Accident or Cerebrovascular Accidents or Chf or Chronic Obstructive Pulmonary Disease or Chronic Obstructive Pulmonary Disorder or Chronic Obstructive Pulmonary Disorders or Complication or Complications or Congestive Heart Failure or Copd or Coronary Artery Disease or Coronary Artery Diseases or Coronary Disease or Coronary Diseases or Cva or Cvas or Cvd or Cvds or Dementia or Dementias or Depressed or Depression or Depressive Disorder or Depressive Disorders or Depressive or Diabetes Mellitus or Diabetes or Diabetic Foot or Foot Complication or Foot Complications or Gestational Diabetes or Glycaemia or Glycaemic or Glycemia or Glycemic or Health Care Evaluation Mechanisms or Health Care Quality Indicator or Health Care Quality Indicators or Health Information Interoperability or Healthcare Evaluation Mechanisms or Healthcare Quality Indicator or Healthcare Quality Indicators or Heart Failure or Interoperability or Interoperable or Managed Pain or Management Of Pain or Managing Pain or Mental Disorder or Mental Disorders or Mental Health or Mental Illness or Mental Illnesses or Mentally Healthy or Mentally Ill or Myocardial Infarction or Neoplasm or Neoplasms or Oncologist or Oncologists or Oncology or Pain Management or Pain or Pains or Pap Test or Pap Tests or Papanicolaou Test or Papanicolaou Tests or Patient Reported Experience Measure or Patient Reported Experience Measures or Patient Reported Outcome Measure or Patient Reported Outcome Measures or Physical Exam or Physical Examination or Physical Examinations or Physical Exams or Prem or Prems or Prom or Promotion or Proms or Psycho Social or Psychosocial or Quality Indicator or Quality indicators Of Health Care Quality Indicators or Rehabilitation or Self Care or Self Efficacy or Stroke or Strokes).ab,ti.

18. 16 or 17

19. exp Allied Health Personnel/ or exp Ambulatory Care Information Systems/ or Ambulatory Care/ or exp "Appointments and Schedules"/ or exp Clinical Competence/ or exp "Duration of Therapy"/ or exp Patient Education as Topic/ or exp Evidence-Based Practice/ or exp Harm Reduction/ or exp Health Behavior/ or exp Health Care Costs/ or exp "Health Care Economics and Organizations"/ or exp "Health Care Facilities, Manpower, and Services"/ or exp "Health Care Quality, Access, and Evaluation"/ or exp Health Knowledge, Attitudes, Practice/ or exp Health Plan Implementation/ or exp Health Services Accessibility/ or exp Healthcare Disparities/ or exp Hospital Shared Services/ or exp House Calls/ or exp Length of Stay/ or exp Office Visits/ or exp Patient Acceptance of Health Care/ or exp Patient Admission/ or exp Patient Advocacy/ or exp Prescriptions/ or exp "Referral and Consultation"/ or exp Universal Health Care/

20. (Access To Care or Access To Health Care or Access To Health Services or Access To Healthcare or Accessibility Of Health Services or Allied Health Personnel or Ambulatory Care Information System or Ambulatory Care Information Systems or Ambulatory Care or Appointment or Appointments or Care Access or Clinical Competence or Clinical Competencies or Clinical Competency or Consultation or Consultations or Cost or Costs or Duration Of Therapy or Patient Education or Patient Teaching or Evaluated Health Care or Evaluated Healthcare or Evaluating Health Care or Evaluating Healthcare or Evaluation Of Health Care or Evaluations Of Health Care or Harm Prevention or Harm Reduction or Health Attitude or Health Attitudes or Health Behavior or Health Behaviors or Health Behaviour or Health Behaviours or HealthCare or Health Care or Health Knowledge or Health Personnel or Health Plan Implementation or Health Service Accessibility or Health Services Accessibility or Healthy Environment or Healthy Environments or Hospital Shared Services or House Call or House Calls or Indirect Cost or indirect Costs or Knowledge Of Health or Length Of Stay or Length Of Therapy or Lengths Of Stay or Los or Office Visit or Office Visits or Patient Acceptance or Patient Admission or Patient Admissions or Patient Advocacy or Prescription or Prescriptions or Prevention Of Harm or Quality Health Care or Quality Healthcare or Reduction Of Harm or Referral or Referrals or Return Visit or Return Visits or Schedule or

Scheduled or Schedules or Therapy Duration or Therapy Durations or Therapy Length or Universal Health Care or Universal Healthcare or Unplanned).ab,ti.

21. 19 or 20

22. exp Delivery of Health Care/ or exp Frailty/ or exp Global Health/ or exp Health Equity/ or exp Health Literacy/ or exp Health Status Disparities/ or exp Healthcare Disparities/ or exp Income/ or exp Insurance Coverage/ or exp "International Classification of Functioning, Disability and Health"/ or exp Marital Status/ or exp Minority Health/ or exp Oceanic Ancestry Group/ or exp Patient Selection/ or exp Population Dynamics/ or exp Population Groups/ or exp Poverty Areas/ or exp Refusal to Treat/ or Religion/ or exp Social Discrimination/ or exp Socioeconomic Factors/ or exp Treatment Failure/

23. (Aboriginal or Communication Barriers or Delivery Of Health Care or Delivery Of Healthcare or Disability Evaluation or Discrimination or Equity or Frailty or Gender Minorities or Gender Minority or Global Health or Health Care Delivery or Health Care Disparities or Health Care Disparity or Health Equity or Health Literacy or Health Status Disparities or Health Status Disparity or Healthcare Delivery or Healthcare Disparities or Healthcare Disparity or Income or Incomes or Inequal or Inequalities or Inequality or Insurance Cover or Insurance Coverage or Insurance Coverages or Insurance Covered or "International Classification Of Functioning Disability and Health" or Marital Status or Minority Health or Oceanic Ancestry Group or Oceanic Ancestry Groups or Patient Selection or Population Dynamics or Population Group or Population Groups or Poverty Area or Poverty Areas or Poverty or Refusal To Treat or Religion or Religions or Safety Net or Sexual Minorities or Sexual Minority or Sexual Orientation or Sexual Orientations or Social Discrimination or Social Discriminations or Social Support or Treatment Failure or Treatment Failures or Treatment Preference or Treatment Preferences or Vulnerabilities or Vulnerability or Vulnerable).ab,ti.

24. 22 or 23

25. 9 or 12 or 15 or 18 or 21 or 24

26. ((Systematic* adj3 (Review* or Overview*)) or (Methodologic* adj3 (Review* or Overview*))).ti,ab,kf,kw.

27. Meta-Analysis.pt.

28. meta-analysis/ or systematic review/ or meta-analysis as topic/ or "meta analysis (topic)"/ or "systematic review (topic)"/

29. exp meta-analysis/

30. Meta Analy*.tw.

31. Metanaly*.tw.

32. Metaanaly*.tw.

33. Met Analy*.tw.

34. ((overview$ or review or synthesis or summary or cochrane or analysis) and (reviews or meta-analyses or articles)).ti.

35. (meta-review or metareview).ti,ab.

36. ((overview$ or reviews) and (systematic or cochrane)).ti.

37. (reviews adj2 (meta or published or quality or included or summar$)).ab.

38. cochrane reviews.ab.

39. (evidence and (reviews or meta-analyses)).ti.

40. 3 and 6 and 25

41. 26 or 27 or 28 or 29 or 30 or 31 or 32 or 33 or 34 or 35 or 36 or 37 or 38 or 39

42. 40 and 41

43. limit 42 to yr="2010 -Current"

3. EMBASE

1. ambulatory care/ or exp community care/ or exp community health nursing/ or exp community medicine/ or exp community mental health service/ or exp general practice/ or exp general practitioner/ or exp group practice/ or exp health care delivery/ or exp health care personnel/ or exp health care planning/ or exp health center/ or exp health service/ or exp home care/ or exp home for the aged/ or exp long term care/ or exp nurse/ or exp nursing care/ or exp nursing home/ or exp nursing station/ or exp nursing/ or exp outpatient department/ or exp outpatient/ or exp patient care/ or exp primary health care/ or exp professional practice/ or exp respite care/ or exp rural health care/ or exp rural health nursing/ or exp rural health/ or exp team nursing/ or exp teleconsultation/ or exp telemedicine/ or exp telenursing/ or exp telerehabilitation/

2. (ambulatory care or Care Continuity or Chsld or Chslds or Clsc or Clsc or Community Care or Community Clinic or Community Clinics or Community Doctor or Community Doctors or Community Facilities or Community Facility or Community Health Center or Community Health Centers or Community Health Centre or Community Health Centres or Community Health Nurse or Community Health Nurses or Community Health Nursing or Community Health Plan or Community Health Planning or Community Health Plans or Community Health Service or Community Health Services or Community Medicine or Community Mental Health Service or Community Mental Health Services or Community Nurse or Community Nurses or Community Nursing or Community Physician or Community Physicians or Community Practice or Community Practices or Community Program or Community Programs or Community Service or Community Services or Continuity of Care or Counseling or Counselling or Delivery of Health Care or Delivery of Healthcare or Distance Counseling or Distance Counselling or Elder Care or Elderly Nursing Home or Elderly Nursing Homes or Family Clinic or Family Clinics or Family Doctor or Family Doctors or Family Medicine Group or Family Medicine Groups or Family Medicine or Family Physician or Family Physicians or Family Practice or Family Practices or General Practice or General Practices or General Practitioner or General Practitioners or Group Practice or Group Practices or Health Care Delivery or Health Care Professional or Health Care Professionals or Health Care Provider or Health Care Providers or Health Care Team or Health Care Teams or Health Personnel or Health Professional or Health Professionals or Health Services Administration or Healthcare Delivery or Healthcare Professional or Healthcare Professionals or Healthcare Provider or Healthcare Providers or Healthcare Team or Healthcare Teams or Home Care or Home Health or Home Nurse or Home Nurses or Home Nursing or Interactive Consultation or Interactive Consultations or Interactive Service or Interactive Services or Interdisciplinary Team or Interdisciplinary Teams or Intermediate Care Facility or Intermediate Care or Interprofessional Team or Interprofessional Teams or Local Community Service Center or Local Community Service Centers or Local Community Service Centre or Local Community Service Centres or Long-Term Care Center or Long-Term Care Centers or Long-Term Care Centre or Long-Term Care Centres or Long-Term Care Home or Long-Term Care Homes or Multidisciplinary Team or Multidisciplinary Teams or Multidiscipline Team or Multidiscipline Teams or Nurse Management or Nurse or Nurse-Delivered or Nurse-Led or Nurse-Managed or Nurse-Run or Nurses Management or Nurses or Nurses-Led or Nursing Care or Nursing Delivered or Nursing Home or Nursing Homes or Nursing Management or Nursing or Nursing-Delivered or Nursing-Led or Nursing-Managed or Nursing-Run or Office Nurse or Office Nurses or Office Nursing or Outpatient or Outpatients or Patient Care Team or Patient Care Teams or Primary Care or Primary Health Care or Primary Healthcare or Professional Autonomy or Professional Practice or Professional Practices or Remote or Residential Care Center or Residential Care Centers or Residential Care Centre or Residential Care Centres or Residential Care or Respite Care or Retirement Home or Retirement Homes or Rural Health or Rural Nurse or Rural Nurses or Rural Nursing or Staff Nurse or Staff Nurses or Suburban Health Service or Suburban or Team or Teams or Teamwork or Telemedicine or Tele-Medicine or Telenurse or Tele-Nurse or Telenurses or Tele-Nurses or Telenursing or Tele-Nursing or Telerehabilitation or Tele-Rehabilitation or Walk-In Clinic or Walk-In Clinics).ab,ti.

3. 1 or 2

4. exp advanced practice nursing/ or exp family nurse practitioner/ or exp nurse practitioner/

5. (Advanced Nurse Practitioner or Advanced Nurse Practitioners or (ANP and Nurse) or (ANP and Nurses) or (ANP and Nursing) or Advanced Nursing Practice or Advanced Nursing Practices or Advanced Practice Nurse or Advanced Practice Nurses or Advanced Practice Nursing or (APN and Nurse) or (APN and Nurses) or (APN and Nursing) or Advanced Practice Registered Nurse or Advanced Practice Registered Nurses or Advanced Practice Registered Nursing or (APRN and Nurse) or (APRN and Nurses) or (APRN and Nursing) or Emergency Nurse Practitioner or Emergency Nurse Practitioners or (APRN and Nurse) or (APRN and Nurses) or (APRN and Nursing) or Family nurse practitioner or Family nurse practitioners or (FNP and Nurse) or (FNP and Nurses) or (FNP and Nursing) or Nurse Practitioner or Nurse Practitioners or (NP and Nurse) or (NP and Nurses) or (NP and Nursing) or Primary Care Nurse Practitioner or Primary Care Nurse Practitioners or Primary Health Care Nurse Practitioner or (PCNP and Nurse) or (PCNP and Nurses) or (PCNP and Nursing) or Primary Health Care Nurse Practitioners or Primary Healthcare Nurse Practitioner or Primary Health Care Nurse Practitioner or Primary Healthcare Nurse Practitioners or Primary Health care Nurse Practitioners or (PHCNP and Nurse) or (PHCNP and Nurses) or (PHCNP and Nursing)).ab,ti.

6. 4 or 5

7. exp consultation/ or exp patient referral/ or (exp glycosylated hemoglobin/ or exp hemoglobin A1c/) or biological marker/ or exp "cost benefit analysis"/ or exp "cost"/ or exp cholesterol/ or exp chronic disease/ or exp consumer attitude/ or exp diagnostic test/ or exp health care delivery/ or exp health care planning/ or exp health care quality/ or exp health service/ or exp health status indicator/ or exp health status/ or exp hospital management/ or exp hypercholesterolemia/ or exp job satisfaction/ or exp mortality/ or exp outcome assessment/ or exp prescription/ or exp professional standard/ or exp protocol compliance/ or exp satisfaction/ or exp task performance/ or exp treatment outcome/ or hypertension/ or pain/

8. (Appointment or Appointments or Attendance or Biomarker or Biomarkers or Hypertension or Care Process or Care Processes or Cholesterol or Hypercholesterolemia or Chronic Condition or Chronic Conditions or Chronic Disease or Chronic Diseases or Chronic Illness or Chronic Illnesses or Clinical Outcome or Clinical Outcomes or Clinical Parameter or Clinical Parameters or Compliance or Compliant or Consultation or Consultations or Consumer Behavior or Consumer Behaviors or Consumer Behaviour or Consumer Behaviours or Cost Analyses or Cost Analysis or Cost or Costs or Death or Deaths or Delivery Of Health Care or Delivery Of Healthcare or Diagnostic Test or Diagnostic Testing or Diagnostic Tests or Economic Analyses or Economic Analysis or Economic Evaluation or Economic Evaluations or Emergency Service or Emergency Services or Follow Up Appointment or Follow Up Appointments or Follow Up Visit or Follow Up Visits or Glycated Haemoglobin A or Glycated Hemoglobin A or Guideline Adherence or HbA1C or Hdl or Health Care or Health Planning or Health Related Quality Of Life or Health Resource or Health Resources or Health Service or Health Services or Health Status or Healthcare Delivery or Healthcare Process Assessment or Healthcare Quality or Healthcare or High Density Lipoprotein or Hospital Admission or Hospital Admissions or Hospital Referral or Hospital Referrals or Hrqol or Hypertension or Indicator or Indicators or Job Satisfaction or Ldl or Lipid Profile or Lipid Profiles or Low Density Lipoprotein or Manpower or Mortalities or Mortality or Outcome Assessment or Outcome Assessments or Pain or Pains or Patient Satisfaction or Personal Satisfaction or Prefer or Preference or Preferences or Preferred or Prescribe or Prescribed or Prescription or Prescriptions or Process Of Care or Processes Of Care or Professional Role or Professional Roles or Qol or Quality Indicator or Quality Indicators or Quality Of Health Care or Quality Of Healthcare or Quality Of Life or Referral or Referrals or Return Appointment or Return Appointments or Return Visit or Return Visits or Routine Diagnostic or Routine Test or Routine Testing or Routine Tests or Satisfaction or Satisfactory or Satisfied or Schedule or Schedules or Task Analyses or Task

Analysis or Task Performance or Treatment Adherence or Treatment Adherent or Treatment Compliance or Treatment Compliant or Wellness).ab,ti.

9. 7 or 8

10. exp anthropometry/ or exercise/ or exp tobacco dependence/ or exp body weight/ or exp diet/ or exp health education/ or exp health promotion/ or exp healthy lifestyle/ or exp mental health/ or exp nutritional assessment/ or exp nutritional status/ or exp passive smoking/ or exp poverty/ or exp preventive health service/ or exp smoking cessation/ or exp smoking prevention/ or exp smoking/ or exp teaching/ or exp transcultural care/

11. (Anthropometric or Anthropometrical or Anthropometrically or Anthropometrics or Anthropometry or Body Weight or Condition Specific or Culturally Competent Care or Culturally Diverse or Diet or Dietary or Dietetical or Dietetically or Dietetics or Diets or Emotional Health or Exercise or Food Regimen or Food Regimens or Health Education or Health Prevention or Health Promotion or Healthy Habit or Healthy Habits or Healthy Life or Healthy Life Habits or Healthy Living or Life Style or Lifestyle or Low Income or Mental Health or Nutrition Assessment or Nutrition or Nutritional Status or Nutritional or Physical Activity or Poverty or Preventive Health Service or Preventive Health Services or Promotion of Health or Promotion or Quit Smoking or Quitting Smoking or Second Hand Smoke or Smoke or Smoked or Smoker or Smokers or Smokes or Smoking or Socioeconomic Status or Stop Smoking or Stopping Smoking or Stress Level or Stress Levels or Taught or Teach or Teaching or Tobacco or Weight).ab,ti.

12. 10 or 11

13. exp adolescent/ or exp child development/ or exp human development/ or exp maternal health service/ or exp pediatrics/ or exp primary prevention/ or exp secondary prevention/ or exp vaccination/ or immunization/ or sexually transmitted disease/

14. (Adolescence or Adolescent or Adolescents or Child Development or Childhood or Healthy Development or Human Development or Immunisation or Immunise or Immunised or Immunises or Immunization or Immunize or Immunized or Immunizes or Laboratory Test or Maternal Health Service or Maternal Health Services or Paediatric or Paediatrics or Pediatric or Pediatrics or Pregnancy or Prevent or Prevented or Prevention or Prevents or Primary Prevention or Secondary Prevention or Sexually Transmitted or Vaccinate or Vaccinated or Vaccinates or Vaccination or Vaccine or Vaccines or Youth or Youths).ab,ti.

15. 13 or 14

16. acute coronary syndrome/ or (exp inferior myocardial infarction/ or exp anterior myocardial infarction/) or cardiovascular disease/ or cerebrovascular accident/ or coronary artery disease/ or dementia/ or diabetes mellitus/ or exp adjustment disorder/ or exp alcoholism/ or exp analgesia/ or exp anxiety disorder/ or exp chronic obstructive lung disease/ or exp data interoperability/ or exp depression/ or exp diabetic foot/ or exp drinking behavior/ or exp glucose blood level/ or exp health care quality/ or exp health care quality/ or exp hyperglycemia/ or exp hypoglycemia/ or exp mental disease/ or exp papanicolaou test/ or exp patient-reported outcome/ or exp physical examination/ or exp pregnancy diabetes mellitus/ or exp rehabilitation/ or exp self care/ or exp self concept/ or heart failure/ or neoplasm/ or pain/

17. (Acs or Acss or Acute Coronary Syndrome or Acute Coronary Syndromes or Adjustment Disorder or Adjustment Disorders or Alcohol Consumption or Alcohol Drinking or Alcohol Related Disorder or Alcohol Related Disorders or Alcoholic or Alcoholism or Anxiety or Anxious or Blood Glucose or Blood Sugar or Blood Sugars or Hyperglycemia or Hypoglycemia or Cancer or Cardiovascular Disease or Cardiovascular Diseases or Cerebrovascular Accident or Cerebrovascular Accidents or Chf or Chronic Obstructive Pulmonary Disease or

Chronic Obstructive Pulmonary Disorder or Chronic Obstructive Pulmonary Disorders or Complication or Complications or Congestive Heart Failure or Copd or Coronary Artery Disease or Coronary Artery Diseases or Coronary Disease or Coronary Diseases or Cva or Cvas or Cvd or Cvds or Dementia or Dementias or Depressed or Depression or Depressive Disorder or Depressive Disorders or Depressive or Diabetes Mellitus or Diabetes or Diabetic Foot or Foot Complication or Foot Complications or Gestational Diabetes or Glycaemia or Glycaemic or Glycemia or Glycemic or Health Care Evaluation Mechanisms or Health Care Quality Indicator or Health Care Quality Indicators or Health Information Interoperability or Healthcare Evaluation Mechanisms or Healthcare Quality Indicator or Healthcare Quality Indicators or Heart Failure or Interoperability or Interoperable or Managed Pain or Management Of Pain or Managing Pain or Mental Disorder or Mental Disorders or Mental Health or Mental Illness or Mental Illnesses or Mentally Healthy or Mentally Ill or Myocardial Infarction or Neoplasm or Neoplasms or Oncologist or Oncologists or Oncology or Pain Management or Pain or Pains or Pap Test or Pap Tests or Papanicolaou Test or Papanicolaou Tests or Patient Reported Experience Measure or Patient Reported Experience Measures or Patient Reported Outcome Measure or Patient Reported Outcome Measures or Physical Exam or Physical Examination or Physical Examinations or Physical Exams or Prem or Prems or Prom or Promotion or Proms or Psycho Social or Psychosocial or Quality Indicator or Quality indicators Of Health Care Quality Indicators or Rehabilitation or Self Care or Self Efficacy or Stroke or Strokes).ab,ti.

18. 16 or 17

19. exp "health care cost"/ or exp "length of stay"/ or (exp health care facility/ or exp health service/) or (exp patient referral/ or exp consultation/) or ambulatory care/ or exp "evaluation and follow up"/ or exp ambulatory care/ or exp attitude/ or exp clinical competence/ or exp evidence based practice/ or exp harm reduction/ or exp health behavior/ or exp health care access/ or exp health care disparity/ or exp health care planning/ or exp health care quality/ or exp hospital admission/ or exp hospital information system/ or exp hospital management/ or exp hospital service/ or exp paramedical personnel/ or exp patient advocacy/ or exp patient attitude/ or exp patient education/ or exp prescription/ or exp treatment duration/ or exp universal health care/

20. (Access To Care or Access To Health Care or Access To Health Services or Access To Healthcare or Accessibility Of Health Services or Allied Health Personnel or Ambulatory Care Information System or Ambulatory Care Information Systems or Ambulatory Care or Appointment or Appointments or Care Access or Clinical Competence or Clinical Competencies or Clinical Competency or Consultation or Consultations or Cost or Costs or Duration Of Therapy or Patient Education or Patient Teaching or Evaluated Health Care or Evaluated Healthcare or Evaluating Health Care or Evaluating Healthcare or Evaluation Of Health Care or Evaluations Of Health Care or Harm Prevention or Harm Reduction or Health Attitude or Health Attitudes or Health Behavior or Health Behaviors or Health Behaviour or Health Behaviours or HealthCare or Health Care or Health Knowledge or Health Personnel or Health Plan Implementation or Health Service Accessibility or Health Services Accessibility or Healthy Environment or Healthy Environments or Hospital Shared Services or House Call or House Calls or Indirect Cost or indirect Costs or Knowledge Of Health or Length Of Stay or Length Of Therapy or Lengths Of Stay or Los or Office Visit or Office Visits or Patient Acceptance or Patient Admission or Patient Admissions or Patient Advocacy or Prescription or Prescriptions or Prevention Of Harm or Quality Health Care or Quality Healthcare or Reduction Of Harm or Referral or Referrals or Return Visit or Return Visits or Schedule or Scheduled or Schedules or Therapy Duration or Therapy Durations or Therapy Length or Universal Health Care or Universal Healthcare or Unplanned).ab,ti.

21. 19 or 20

22. exp health care delivery/ or exp "International Classification of Diseases"/ or exp frailty/ or exp global health/ or exp health disparity/ or exp health equity/ or exp health literacy/ or exp income/ or exp insurance/ or

exp marriage/ or exp minority health/ or exp Oceanic ancestry group/ or exp patient selection/ or exp population dynamics/ or exp population group/ or exp poverty/ or exp social discrimination/ or exp socioeconomics/ or exp treatment failure/ or exp treatment refusal/ or religion/

23. (Aboriginal or Communication Barriers or Delivery Of Health Care or Delivery Of Healthcare or Disability Evaluation or Discrimination or Equity or Frailty or Gender Minorities or Gender Minority or Global Health or Health Care Delivery or Health Care Disparities or Health Care Disparity or Health Equity or Health Literacy or Health Status Disparities or Health Status Disparity or Healthcare Delivery or Healthcare Disparities or Healthcare Disparity or Income or Incomes or Inequal or Inequalities or Inequality or Insurance Cover or Insurance Coverage or Insurance Coverages or Insurance Covered or "International Classification Of Functioning Disability and Health" or Marital Status or Minority Health or Oceanic Ancestry Group or Oceanic Ancestry Groups or Patient Selection or Population Dynamics or Population Group or Population Groups or Poverty Area or Poverty Areas or Poverty or Refusal To Treat or Religion or Religions or Safety Net or Sexual Minorities or Sexual Minority or Sexual Orientation or Sexual Orientations or Social Discrimination or Social Discriminations or Social Support or Treatment Failure or Treatment Failures or Treatment Preference or Treatment Preferences or Vulnerabilities or Vulnerability or Vulnerable).ab,ti.

24. 22 or 23

25. 9 or 12 or 15 or 18 or 21 or 24

26. systematic.ti,ab,kw. or meta-analysis.pt. or exp meta analysis/ or meta analy*.tw. or metanaly*.tw. or metaanaly*.tw. or met analy*.tw. or integrative research.ab,ti. or research overview*.ab,ti. or systematic review*.ab,ti.

27. ((overview* or review or synthesis or summary or cochrane or analysis) and (reviews or meta-analyses or articles)).ti.

28. (meta-review or meta review).ab,ti.

29. ((overview* or reviews) and (systematic or cochrane)).ti.

30. (reviews and (meta or published or quality or included or summar*)).ab,ti.

31. "cochrane reviews".ab,ti.

32. (evidence and (reviews or meta-analyses)).ti.

33. 3 and 6 and 25

34. 26 or 27 or 28 or 29 or 30 or 31 or 32

35. 33 and 34

36. limit 35 to (embase and yr="2010 -Current")

4. Cochrane

1. exp Ambulatory Care Facilities/ or Ambulatory Care/ or exp Community Health Centers/ or exp Community Health Nursing/ or exp Community Health Planning/ or exp Community Health services/ or exp Community medicine/ or Community Mental Health Services/ or exp Delivery of Health Care/ or exp family practice/ or exp General Practice/ or exp General Practitioners/ or exp group practice/ or exp Health Personnel/ or exp Health Services Administration/ or exp Home Care Services/ or exp Home Nursing/ or exp Homes for the Aged/ or exp Housing for the Elderly/ or exp Intermediate Care Facilities/ or exp Long-Term Care/ or exp Nurses, Community Health/ or exp Nursing Care/ or exp Nursing Homes/ or exp Nursing Stations/ or exp Nursing/ or exp Nursing, team/ or exp Office Nursing/ or exp outpatients/ or exp Patient Care Team/ or exp Physicians, Family/ or exp Physicians, Primary Care/ or exp Primary Care Nursing/ or exp Primary Health Care/ or exp Professional Autonomy/ or exp Professional Practice/ or exp Remote Consultation/ or exp Respite Care/ or exp Rural Health Services/ or exp Rural Health/ or exp Rural Nursing/ or exp Suburban Health Services/ or Telemedicine/ or exp Telenursing/

2. (ambulatory care or Care Continuity or Chsld or Chslds or Clsc or Clsc or Community Care or Community Clinic or Community Clinics or Community Doctor or Community Doctors or Community Facilities or Community Facility or Community Health Center or Community Health Centers or Community Health Centre or Community Health Centres or Community Health Nurse or Community Health Nurses or Community Health Nursing or Community Health Plan or Community Health Planning or Community Health Plans or Community Health Service or Community Health Services or Community Medicine or Community Mental Health Service or Community Mental Health Services or Community Nurse or Community Nurses or Community Nursing or Community Physician or Community Physicians or Community Practice or Community Practices or Community Program or Community Programs or Community Service or Community Services or Continuity of Care or Counseling or Counselling or Delivery of Health Care or Delivery of Healthcare or Distance Counseling or Distance Counselling or Elder Care or Elderly Nursing Home or Elderly Nursing Homes or Family Clinic or Family Clinics or Family Doctor or Family Doctors or Family Medicine Group or Family Medicine Groups or Family Medicine or Family Physician or Family Physicians or Family Practice or Family Practices or General Practice or General Practices or General Practitioner or General Practitioners or Group Practice or Group Practices or Health Care Delivery or Health Care Professional or Health Care Professionals or Health Care Provider or Health Care Providers or Health Care Team or Health Care Teams or Health Personnel or Health Professional or Health Professionals or Health Services Administration or Healthcare Delivery or Healthcare Professional or Healthcare Professionals or Healthcare Provider or Healthcare Providers or Healthcare Team or Healthcare Teams or Home Care or Home Health or Home Nurse or Home Nurses or Home Nursing or Interactive Consultation or Interactive Consultations or Interactive Service or Interactive Services or Interdisciplinary Team or Interdisciplinary Teams or Intermediate Care Facility or Intermediate Care or Interprofessional Team or Interprofessional Teams or Local Community Service Center or Local Community Service Centers or Local Community Service Centre or Local Community Service Centres or Long-Term Care Center or Long-Term Care Centers or Long-Term Care Centre or Long-Term Care Centres or Long-Term Care Home or Long-Term Care Homes or Multidisciplinary Team or Multidisciplinary Teams or Multidiscipline Team or Multidiscipline Teams or Nurse Management or Nurse or Nurse-Delivered or Nurse-Led or Nurse-Managed or Nurse-Run or Nurses Management or Nurses or Nurses-Led or Nursing Care or Nursing Delivered or Nursing Home or Nursing Homes or Nursing Management or Nursing or Nursing-Delivered or Nursing-Led or Nursing-Managed or Nursing-Run or Office Nurse or Office Nurses or Office Nursing or Outpatient or Outpatients or Patient Care Team or Patient Care Teams or Primary Care or Primary Health Care or Primary Healthcare or Professional Autonomy or Professional Practice or Professional Practices or Remote or Residential Care Center or Residential Care Centers or Residential Care Centre or Residential Care Centres or Residential Care or Respite Care or Retirement Home or Retirement Homes or Rural Health or Rural Nurse or Rural Nurses or Rural Nursing or Staff Nurse or Staff Nurses or Suburban Health Service or Suburban or Team or

Teams or Teamwork or Telemedicine or Tele-Medicine or Telenurse or Tele-Nurse or Telenurses or Tele-Nurses or Telenursing or Tele-Nursing or Telerehabilitation or Tele-Rehabilitation or Walk-In Clinic or Walk-In Clinics).ab,ti.

3. 1 or 2

4. exp Advanced Practice Nursing/

5. exp Family Nurse Practitioners/

6. exp Nurse Practitioners/

7. 4 or 5 or 6

8. exp Advanced Practice Nursing/ or exp Family Nurse Practitioners/ or exp Nurse Practitioners/

9. (Advanced Nurse Practitioner or Advanced Nurse Practitioners or (ANP and Nurse) or (ANP and Nurses) or (ANP and Nursing) or Advanced Nursing Practice or Advanced Nursing Practices or Advanced Practice Nurse or Advanced Practice Nurses or Advanced Practice Nursing or (APN and Nurse) or (APN and Nurses) or (APN and Nursing) or Advanced Practice Registered Nurse or Advanced Practice Registered Nurses or Advanced Practice Registered Nursing or (APRN and Nurse) or (APRN and Nurses) or (APRN and Nursing) or Emergency Nurse Practitioner or Emergency Nurse Practitioners or (APRN and Nurse) or (APRN and Nurses) or (APRN and Nursing) or Family nurse practitioner or Family nurse practitioners or (FNP and Nurse) or (FNP and Nurses) or (FNP and Nursing) or Nurse Practitioner or Nurse Practitioners or (NP and Nurse) or (NP and Nurses) or (NP and Nursing) or Primary Care Nurse Practitioner or Primary Care Nurse Practitioners or Primary Health Care Nurse Practitioner or (PCNP and Nurse) or (PCNP and Nurses) or (PCNP and Nursing) or Primary Health Care Nurse Practitioners or Primary Healthcare Nurse Practitioner or Primary Health Care Nurse Practitioner or Primary Healthcare Nurse Practitioners or Primary Health care Nurse Practitioners or (PHCNP and Nurse) or (PHCNP and Nurses) or (PHCNP and Nursing)).ab,ti.

10. 8 or 9

11. exp "appointments and schedules"/

12. Biological Markers/

13. Hypertension/

14. exp Cholesterol/

15. exp Hypercholesterolemia/

16. exp chronic disease/

17. exp "costs and cost analysis"/

18. exp "Delivery of Health Care"/

19. exp diagnostic tests, routine/

20. exp hemoglobin a/ or exp hemoglobin a, glycosylated/

21. exp guideline adherence/

22. exp health resources/

23. exp Health Services/

24. exp health status indicators/

25. exp health status/

26. exp job satisfaction/

27. exp mortality/

28. exp "outcome and process assessment (health care)"/

29. exp "outcome assessment (health care)"/

30. pain/

31. exp patient satisfaction/

32. exp personal satisfaction/

33. exp Prescriptions/

34. exp professional role/

35. exp "quality of health care"/

36. exp "referral and consultation"/

37. exp "task performance and analysis"/

38. 11 or 12 or 13 or 14 or 15 or 16 or 17 or 18 or 19 or 20 or 21 or 22 or 23 or 24 or 25 or 26 or 27 or 28 or 29 or 30 or 31 or 32 or 33 or 34 or 35 or 36 or 37

39. exp "appointments and schedules"/ or Biological Markers/ or Hypertension/ or exp Cholesterol/ or exp Hypercholesterolemia/ or exp chronic disease/ or exp "costs and cost analysis"/ or exp "Delivery of Health Care"/ or exp diagnostic tests, routine/ or exp hemoglobin a/ or exp hemoglobin a, glycosylated/ or exp guideline adherence/ or exp health resources/ or exp Health Services/ or exp health status indicators/ or exp health status/ or exp job satisfaction/ or exp mortality/ or exp "outcome and process assessment (health care)"/ or exp "outcome assessment (health care)"/ or pain/ or exp patient satisfaction/ or exp personal satisfaction/ or exp Prescriptions/ or exp professional role/ or exp "quality of health care"/ or exp "referral and consultation"/ or exp "task performance and analysis"/ or exp "task performance and analysis"/

40. (Appointment or Appointments or Attendance or Biomarker or Biomarkers or Hypertension or Care Process or Care Processes or Cholesterol or Hypercholesterolemia or Chronic Condition or Chronic Conditions or Chronic Disease or Chronic Diseases or Chronic Illness or Chronic Illnesses or Clinical Outcome or Clinical Outcomes or Clinical Parameter or Clinical Parameters or Compliance or Compliant or Consultation or Consultations or Consumer Behavior or Consumer Behaviors or Consumer Behaviour or Consumer Behaviours or Cost Analyses or Cost Analysis or Cost or Costs or Death or Deaths or Delivery Of Health Care or Delivery Of Healthcare or Diagnostic Test or Diagnostic Testing or Diagnostic Tests or Economic Analyses or Economic Analysis or Economic Evaluation or Economic Evaluations or Emergency Service or Emergency Services or Follow Up Appointment or Follow Up Appointments or Follow Up Visit or Follow Up Visits or Glycated Haemoglobin A or Glycated Hemoglobin A or Guideline Adherence or HbA1C or Hdl or Health Care or Health Planning or Health Related Quality Of Life or Health Resource or Health Resources or Health Service or Health Services or Health Status or Healthcare Delivery or Healthcare Process Assessment or Healthcare Quality or Healthcare or High

Density Lipoprotein or Hospital Admission or Hospital Admissions or Hospital Referral or Hospital Referrals or Hrqol or Hypertension or Indicator or Indicators or Job Satisfaction or Ldl or Lipid Profile or Lipid Profiles or Low Density Lipoprotein or Manpower or Mortalities or Mortality or Outcome Assessment or Outcome Assessments or Pain or Pains or Patient Satisfaction or Personal Satisfaction or Prefer or Preference or Preferences or Preferred or Prescribe or Prescribed or Prescription or Prescriptions or Process Of Care or Processes Of Care or Professional Role or Professional Roles or Qol or Quality Indicator or Quality Indicators or Quality Of Health Care or Quality Of Healthcare or Quality Of Life or Referral or Referrals or Return Appointment or Return Appointments or Return Visit or Return Visits or Routine Diagnostic or Routine Test or Routine Testing or Routine Tests or Satisfaction or Satisfactory or Satisfied or Schedule or Schedules or Task Analyses or Task Analysis or Task Performance or Treatment Adherence or Treatment Adherent or Treatment Compliance or Treatment Compliant or Wellness).ab,ti.

41. 39 or 40

42. exp anthropometry/

43. exp body weight/

44. diet/

45. exp health education/

46. exp health promotion/

47. exp mental health/

48. exp nutrition assessment/

49. exp nutritional status/

50. exp poverty/

51. exp preventive health services/

52. exp smoking cessation/

53. exp smoking/

54. exp teaching/

55. exp Tobacco Smoke Pollution/

56. exp "Tobacco Use"/

57. 42 or 43 or 44 or 45 or 46 or 47 or 48 or 49 or 50 or 51 or 52 or 53 or 54 or 55 or 56

58. exp anthropometry/ or exp body weight/ or diet/ or exp health education/ or exp health promotion/ or exp mental health/ or exp nutrition assessment/ or exp nutritional status/ or exp poverty/ or exp preventive health services/ or exp smoking cessation/ or exp smoking/ or exp teaching/ or exp Tobacco Smoke Pollution/ or exp "Tobacco Use"/

59. (Anthropometric or Anthropometrical or Anthropometrically or Anthropometrics or Anthropometry or Body Weight or Condition Specific or Culturally Competent Care or Culturally Diverse or Diet or Dietary or Dietetical or Dietetically or Dietetics or Diets or Emotional Health or Exercise or Food Regimen or Food Regimens or Health Education or Health Prevention or Health Promotion or Healthy Habit or Healthy Habits or Healthy Life

or Healthy Life Habits or Healthy Living or Life Style or Lifestyle or Low Income or Mental Health or Nutrition Assessment or Nutrition or Nutritional Status or Nutritional or Physical Activity or Poverty or Preventive Health Service or Preventive Health Services or Promotion of Health or Promotion or Quit Smoking or Quitting Smoking or Second Hand Smoke or Smoke or Smoked or Smoker or Smokers or Smokes or Smoking or Socioeconomic Status or Stop Smoking or Stopping Smoking or Stress Level or Stress Levels or Taught or Teach or Teaching or Tobacco or Weight).ab,ti.

60. 58 or 59

61. exp adolescent/

62. exp child development/

63. exp Human Development/

64. immunization/

65. exp maternal health services/

66. exp pediatrics/

67. exp primary prevention/

68. exp secondary prevention/

69. Sexually Transmitted Diseases/

70. exp vaccination/

71. 61 or 62 or 63 or 64 or 65 or 66 or 67 or 68 or 69 or 70

72. exp adolescent/ or exp child development/ or exp Human Development/ or immunization/ or exp maternal health services/ or exp pediatrics/ or exp primary prevention/ or exp secondary prevention/ or Sexually Transmitted Diseases/ or exp vaccination/

73. (Adolescence or Adolescent or Adolescents or Child Development or Childhood or Healthy Development or Human Development or Immunisation or Immunise or Immunised or Immunises or Immunization or Immunize or Immunized or Immunizes or Laboratory Test or Maternal Health Service or Maternal Health Services or Paediatric or Paediatrics or Pediatric or Pediatrics or Pregnancy or Prevent or Prevented or Prevention or Prevents or Primary Prevention or Secondary Prevention or Sexually Transmitted or Vaccinate or Vaccinated or Vaccinates or Vaccination or Vaccine or Vaccines or Youth or Youths).ab,ti.

74. (Adolescence or Adolescent or Adolescents or Child Development or Childhood or Healthy Development or Human Development or Immunisation or Immunise or Immunised or Immunises or Immunization or Immunize or Immunized or Immunizes or Laboratory Test or Maternal Health Service or Maternal Health Services or Paediatric or Paediatrics or Pediatric or Pediatrics or Pregnancy or Prevent or Prevented or Prevention or Prevents or Primary Prevention or Secondary Prevention or Sexually Transmitted or Vaccinate or Vaccinated or Vaccinates or Vaccination or Vaccine or Vaccines or Youth or Youths).ab,ti.

75. 72 or 74

76. acute coronary syndrome/

77. exp Adjustment Disorders/

78. exp alcohol drinking/

79. exp Alcohol-Related Disorders/

80. exp Anxiety Disorders/

81. exp blood glucose/

82. exp hyperglycemia/

83. exp Hypoglycemia/

84. cardiovascular diseases/

85. coronary disease/

86. dementia/

87. exp depressive disorder/

88. Diabetes Mellitus/

89. exp diabetes, gestational/

90. exp Diabetic Foot/

91. exp health care evaluation mechanisms/

92. Heart Failure/

93. exp myocardial infarction/

94. exp mental disorders/

95. neoplasms/

96. exp pain management/

97. pain/

98. exp papanicolaou test/

99. exp physical examination/

100. exp Pulmonary Disease, Chronic Obstructive/

101. exp Quality Indicators, Health Care/

102. exp Rehabilitation/

103. exp self care/

104. exp self efficacy/

105. Stroke/

106. Acute Coronary Syndrome/ or exp Adjustment Disorders/ or exp Alcohol Drinking/ or exp Alcohol-Related Disorders/ or exp Anxiety Disorders/ or exp Blood Glucose/ or exp Hyperglycemia/ or exp Hypoglycemia/ or Cardiovascular Diseases/ or coronary disease/ or Dementia/ or exp Depressive Disorder/ or

Diabetes Mellitus/ or exp Diabetes, Gestational/ or Diabetic Foot/ or exp Health Care Evaluation Mechanisms/ or Heart Failure/ or exp Myocardial Infarction/ or exp Mental Disorders/ or Neoplasms/ or exp Pain Management/ or Pain/ or exp Papanicolaou Test/ or exp Physical Examination/ or exp Pulmonary Disease, Chronic Obstructive/ or exp Quality Indicators, Health Care/ or exp Rehabilitation/ or exp Self Care/ or exp Self Efficacy/ or Stroke/

107. acute coronary syndrome/ or exp Adjustment Disorders/ or exp alcohol drinking/ or exp Alcohol-Related Disorders/ or exp Anxiety Disorders/ or exp blood glucose/ or exp hyperglycemia/ or exp Hypoglycemia/ or cardiovascular diseases/ or coronary disease/ or dementia/ or exp depressive disorder/ or Diabetes Mellitus/ or exp diabetes, gestational/ or exp Diabetic Foot/ or exp health care evaluation mechanisms/ or Heart Failure/ or exp myocardial infarction/ or exp mental disorders/ or neoplasms/ or exp pain management/ or pain/ or exp papanicolaou test/ or exp physical examination/ or exp Pulmonary Disease, Chronic Obstructive/ or exp Quality Indicators, Health Care/ or exp Rehabilitation/ or exp self care/ or exp self efficacy/ or Stroke/

108. (Acs or Acss or Acute Coronary Syndrome or Acute Coronary Syndromes or Adjustment Disorder or Adjustment Disorders or Alcohol Consumption or Alcohol Drinking or Alcohol Related Disorder or Alcohol Related Disorders or Alcoholic or Alcoholism or Anxiety or Anxious or Blood Glucose or Blood Sugar or Blood Sugars or Hyperglycemia or Hypoglycemia or Cancer or Cardiovascular Disease or Cardiovascular Diseases or Cerebrovascular Accident or Cerebrovascular Accidents or Chf or Chronic Obstructive Pulmonary Disease or Chronic Obstructive Pulmonary Disorder or Chronic Obstructive Pulmonary Disorders or Complication or Complications or Congestive Heart Failure or Copd or Coronary Artery Disease or Coronary Artery Diseases or Coronary Disease or Coronary Diseases or Cva or Cvas or Cvd or Cvds or Dementia or Dementias or Depressed or Depression or Depressive Disorder or Depressive Disorders or Depressive or Diabetes Mellitus or Diabetes or Diabetic Foot or Foot Complication or Foot Complications or Gestational Diabetes or Glycaemia or Glycaemic or Glycemia or Glycemic or Health Care Evaluation Mechanisms or Health Care Quality Indicator or Health Care Quality Indicators or Health Information Interoperability or Healthcare Evaluation Mechanisms or Healthcare Quality Indicator or Healthcare Quality Indicators or Heart Failure or Interoperability or Interoperable or Managed Pain or Management Of Pain or Managing Pain or Mental Disorder or Mental Disorders or Mental Health or Mental Illness or Mental Illnesses or Mentally Healthy or Mentally Ill or Myocardial Infarction or Neoplasm or Neoplasms or Oncologist or Oncologists or Oncology or Pain Management or Pain or Pains or Pap Test or Pap Tests or Papanicolaou Test or Papanicolaou Tests or Patient Reported Experience Measure or Patient Reported Experience Measures or Patient Reported Outcome Measure or Patient Reported Outcome Measures or Physical Exam or Physical Examination or Physical Examinations or Physical Exams or Prem or Prems or Prom or Promotion or Proms or Psycho Social or Psychosocial or Quality Indicator or Quality indicators Of Health Care Quality Indicators or Rehabilitation or Self Care or Self Efficacy or Stroke or Strokes).ab,ti.

109. 107 or 108

110. exp Allied Health Personnel/

111. exp Ambulatory Care Information Systems/

112. ambulatory care/

113. exp "appointments and schedules"/

114. exp clinical competence/

115. exp patient education as topic/

116. exp evidence-based practice/

117. exp harm reduction/

118. exp health behavior/

119. exp health care costs/

120. exp "health care economics and organizations"/

121. exp "health care facilities, manpower, and services"/

122. exp "health care quality, access, and evaluation"/

123. exp health knowledge, attitudes, practice/

124. exp health plan implementation/

125. exp health services accessibility/

126. exp healthcare disparities/

127. exp Hospital Shared Services/

128. exp house calls/

129. exp "length of stay"/

130. exp office visits/

131. exp "patient acceptance of health care"/

132. exp patient admission/

133. exp Patient Advocacy/

134. exp Prescriptions/

135. exp "referral and consultation"/

136. 110 or 111 or 112 or 113 or 114 or 115 or 116 or 117 or 118 or 119 or 120 or 121 or 122 or 123 or 124 or 125 or 126 or 127 or 128 or 129 or 130 or 131 or 132 or 133 or 134 or 135

137. exp Allied Health Personnel/ or exp Ambulatory Care Information Systems/ or ambulatory care/ or exp "appointments and schedules"/ or exp clinical competence/ or exp patient education as topic/ or exp evidence-based practice/ or exp harm reduction/ or exp health behavior/ or exp health care costs/ or exp "health care economics and organizations"/ or exp "health care facilities, manpower, and services"/ or exp "health care quality, access, and evaluation"/ or exp health knowledge, attitudes, practice/ or exp health plan implementation/ or exp health services accessibility/ or exp healthcare disparities/ or exp Hospital Shared Services/ or exp house calls/ or exp "length of stay"/ or exp office visits/ or exp "patient acceptance of health care"/ or exp patient admission/ or exp Patient Advocacy/ or exp Prescriptions/ or exp "referral and consultation"/

138. (Access To Care or Access To Health Care or Access To Health Services or Access To Healthcare or Accessibility Of Health Services or Allied Health Personnel or Ambulatory Care Information System or Ambulatory Care Information Systems or Ambulatory Care or Appointment or Appointments or Care Access or Clinical Competence or Clinical Competencies or Clinical Competency or Consultation or Consultations or Cost or Costs or Duration Of Therapy or Patient Education or Patient Teaching or Evaluated Health Care or Evaluated Healthcare or Evaluating Health Care or Evaluating Healthcare or Evaluation Of Health Care or Evaluations Of Health Care or Harm Prevention or Harm Reduction or Health Attitude or Health Attitudes or Health Behavior or

Health Behaviors or Health Behaviour or Health Behaviours or HealthCare or Health Care or Health Knowledge or Health Personnel or Health Plan Implementation or Health Service Accessibility or Health Services Accessibility or Healthy Environment or Healthy Environments or Hospital Shared Services or House Call or House Calls or Indirect Cost or indirect Costs or Knowledge Of Health or Length Of Stay or Length Of Therapy or Lengths Of Stay or Los or Office Visit or Office Visits or Patient Acceptance or Patient Admission or Patient Admissions or Patient Advocacy or Prescription or Prescriptions or Prevention Of Harm or Quality Health Care or Quality Healthcare or Reduction Of Harm or Referral or Referrals or Return Visit or Return Visits or Schedule or Scheduled or Schedules or Therapy Duration or Therapy Durations or Therapy Length or Universal Health Care or Universal Healthcare or Unplanned).ab,ti.

139. 137 or 138

140. exp "Delivery of Health Care"/

141. exp health literacy/

142. exp health status disparities/

143. exp income/

144. exp Insurance Coverage/

145. exp "international classification of functioning, disability and health"/

146. exp marital status/

147. exp Minority Health/

148. exp oceanic ancestry group/

149. exp patient selection/

150. exp population dynamics/

151. exp Population Groups/

152. exp poverty areas/

153. exp refusal to treat/

154. religion/

155. exp social discrimination/

156. exp socioeconomic factors/

157. exp treatment failure/

158. 140 or 141 or 142 or 143 or 144 or 145 or 146 or 147 or 148 or 149 or 150 or 151 or 152 or 153 or 154 or 155 or 156 or 157

159. exp "Delivery of Health Care"/ or exp health literacy/ or exp health status disparities/ or exp income/ or exp Insurance Coverage/ or exp "international classification of functioning, disability and health"/ or exp marital status/ or exp Minority Health/ or exp oceanic ancestry group/ or exp patient selection/ or exp population dynamics/ or exp Population Groups/ or exp poverty areas/ or exp refusal to treat/ or religion/ or exp social discrimination/ or exp socioeconomic factors/ or exp treatment failure/

160. (Aboriginal or Communication Barriers or Delivery Of Health Care or Delivery Of Healthcare or Disability Evaluation or Discrimination or Equity or Frailty or Gender Minorities or Gender Minority or Global Health or Health Care Delivery or Health Care Disparities or Health Care Disparity or Health Equity or Health Literacy or Health Status Disparities or Health Status Disparity or Healthcare Delivery or Healthcare Disparities or Healthcare Disparity or Income or Incomes or Inequal or Inequalities or Inequality or Insurance Cover or Insurance Coverage or Insurance Coverages or Insurance Covered or "International Classification Of Functioning Disability and Health" or Marital Status or Minority Health or Oceanic Ancestry Group or Oceanic Ancestry Groups or Patient Selection or Population Dynamics or Population Group or Population Groups or Poverty Area or Poverty Areas or Poverty or Refusal To Treat or Religion or Religions or Safety Net or Sexual Minorities or Sexual Minority or Sexual Orientation or Sexual Orientations or Social Discrimination or Social Discriminations or Social Support or Treatment Failure or Treatment Failures or Treatment Preference or Treatment Preferences or Vulnerabilities or Vulnerability or Vulnerable).ab,ti.

161. 159 or 160

162. 41 or 60 or 75 or 109 or 139 or 161

163. exp meta-analysis/

164. Meta Analy*.tw.

165. Metanaly*.tw.

166. Metaanaly*.tw.

167. Met Analy*.tw.

168. systematic.sb.

169. ((overview$ or review or synthesis or summary or cochrane or analysis) and (reviews or meta-analyses or articles)).ti.

170. meta-analysis.pt.

171. (meta-review or metareview).ti,ab.

172. ((overview$ or reviews) and (systematic or cochrane)).ti.

173. (reviews adj2 (meta or published or quality or included or summar$)).ab.

174. cochrane reviews.ab.

175. (evidence and (reviews or meta-analyses)).ti.

176. 3 and 10 and 162

5. DARE

1. exp Ambulatory Care Facilities/ or Ambulatory Care/ or exp Community Health Centers/ or exp Community Health Nursing/ or exp Community Health Planning/ or exp Community Health services/ or exp Community medicine/ or Community Mental Health Services/ or exp Delivery of Health Care/ or exp family practice/ or exp General Practice/ or exp General Practitioners/ or exp group practice/ or exp Health Personnel/ or exp Health Services Administration/ or exp Home Care Services/ or exp Home Nursing/ or exp Homes for the Aged/ or exp Housing for the Elderly/ or exp Intermediate Care Facilities/ or exp Long-Term Care/ or exp Nurses, Community Health/ or exp Nursing Care/ or exp Nursing Homes/ or exp Nursing Stations/ or exp Nursing/ or exp Nursing, team/ or exp Office Nursing/ or exp outpatients/ or exp Patient Care Team/ or exp Physicians, Family/ or exp Physicians, Primary Care/ or exp Primary Care Nursing/ or exp Primary Health Care/ or exp Professional Autonomy/ or exp Professional Practice/ or exp Remote Consultation/ or exp Respite Care/ or exp Rural Health Services/ or exp Rural Health/ or exp Rural Nursing/ or exp Suburban Health Services/ or Telemedicine/ or exp Telenursing/

2. (ambulatory care or Care Continuity or Chsld or Chslds or Clsc or Clsc or Community Care or Community Clinic or Community Clinics or Community Doctor or Community Doctors or Community Facilities or Community Facility or Community Health Center or Community Health Centers or Community Health Centre or Community Health Centres or Community Health Nurse or Community Health Nurses or Community Health Nursing or Community Health Plan or Community Health Planning or Community Health Plans or Community Health Service or Community Health Services or Community Medicine or Community Mental Health Service or Community Mental Health Services or Community Nurse or Community Nurses or Community Nursing or Community Physician or Community Physicians or Community Practice or Community Practices or Community Program or Community Programs or Community Service or Community Services or Continuity of Care or Counseling or Counselling or Delivery of Health Care or Delivery of Healthcare or Distance Counseling or Distance Counselling or Elder Care or Elderly Nursing Home or Elderly Nursing Homes or Family Clinic or Family Clinics or Family Doctor or Family Doctors or Family Medicine Group or Family Medicine Groups or Family Medicine or Family Physician or Family Physicians or Family Practice or Family Practices or General Practice or General Practices or General Practitioner or General Practitioners or Group Practice or Group Practices or Health Care Delivery or Health Care Professional or Health Care Professionals or Health Care Provider or Health Care Providers or Health Care Team or Health Care Teams or Health Personnel or Health Professional or Health Professionals or Health Services Administration or Healthcare Delivery or Healthcare Professional or Healthcare Professionals or Healthcare Provider or Healthcare Providers or Healthcare Team or Healthcare Teams or Home Care or Home Health or Home Nurse or Home Nurses or Home Nursing or Interactive Consultation or Interactive Consultations or Interactive Service or Interactive Services or Interdisciplinary Team or Interdisciplinary Teams or Intermediate Care Facility or Intermediate Care or Interprofessional Team or Interprofessional Teams or Local Community Service Center or Local Community Service Centers or Local Community Service Centre or Local Community Service Centres or Long-Term Care Center or Long-Term Care Centers or Long-Term Care Centre or Long-Term Care Centres or Long-Term Care Home or Long-Term Care Homes or Multidisciplinary Team or Multidisciplinary Teams or Multidiscipline Team or Multidiscipline Teams or Nurse Management or Nurse or Nurse-Delivered or Nurse-Led or Nurse-Managed or Nurse-Run or Nurses Management or Nurses or Nurses-Led or Nursing Care or Nursing Delivered or Nursing Home or Nursing Homes or Nursing Management or Nursing or Nursing-Delivered or Nursing-Led or Nursing-Managed or Nursing-Run or Office Nurse or Office Nurses or Office Nursing or Outpatient or Outpatients or Patient Care Team or Patient Care Teams or Primary Care or Primary Health Care or Primary Healthcare or Professional Autonomy or Professional Practice or Professional Practices or Remote or Residential Care Center or Residential Care Centers or Residential Care Centre or Residential Care Centres or

Residential Care or Respite Care or Retirement Home or Retirement Homes or Rural Health or Rural Nurse or Rural Nurses or Rural Nursing or Staff Nurse or Staff Nurses or Suburban Health Service or Suburban or Team or Teams or Teamwork or Telemedicine or Tele-Medicine or Telenurse or Tele-Nurse or Telenurses or Tele-Nurses or Telenursing or Tele-Nursing or Telerehabilitation or Tele-Rehabilitation or Walk-In Clinic or Walk-In Clinics).ab,ti.

3. 1 or 2

4. exp Advanced Practice Nursing/

5. exp Family Nurse Practitioners/

6. exp Nurse Practitioners/

7. 4 or 5 or 6

8. exp Advanced Practice Nursing/ or exp Family Nurse Practitioners/ or exp Nurse Practitioners/

9. (Advanced Nurse Practitioner or Advanced Nurse Practitioners or (ANP and Nurse) or (ANP and Nurses) or (ANP and Nursing) or Advanced Nursing Practice or Advanced Nursing Practices or Advanced Practice Nurse or Advanced Practice Nurses or Advanced Practice Nursing or (APN and Nurse) or (APN and Nurses) or (APN and Nursing) or Advanced Practice Registered Nurse or Advanced Practice Registered Nurses or Advanced Practice Registered Nursing or (APRN and Nurse) or (APRN and Nurses) or (APRN and Nursing) or Emergency Nurse Practitioner or Emergency Nurse Practitioners or (APRN and Nurse) or (APRN and Nurses) or (APRN and Nursing) or Family nurse practitioner or Family nurse practitioners or (FNP and Nurse) or (FNP and Nurses) or (FNP and Nursing) or Nurse Practitioner or Nurse Practitioners or (NP and Nurse) or (NP and Nurses) or (NP and Nursing) or Primary Care Nurse Practitioner or Primary Care Nurse Practitioners or Primary Health Care Nurse Practitioner or (PCNP and Nurse) or (PCNP and Nurses) or (PCNP and Nursing) or Primary Health Care Nurse Practitioners or Primary Healthcare Nurse Practitioner or Primary Health Care Nurse Practitioner or Primary Healthcare Nurse Practitioners or Primary Health care Nurse Practitioners or (PHCNP and Nurse) or (PHCNP and Nurses) or (PHCNP and Nursing)).ab,ti.

10. 8 or 9

11. exp "appointments and schedules"/

12. Biological Markers/

13. Hypertension/

14. exp Cholesterol/

15. exp Hypercholesterolemia/

16. exp chronic disease/

17. exp "costs and cost analysis"/

18. exp "Delivery of Health Care"/

19. exp diagnostic tests, routine/

20. exp hemoglobin a/ or exp hemoglobin a, glycosylated/

21. exp guideline adherence/

22. exp health resources/

23. exp Health Services/

24. exp health status indicators/

25. exp health status/

26. exp job satisfaction/

27. exp mortality/

28. exp "outcome and process assessment (health care)"/

29. exp "outcome assessment (health care)"/

30. pain/

31. exp patient satisfaction/

32. exp personal satisfaction/

33. exp Prescriptions/

34. exp professional role/

35. exp "quality of health care"/

36. exp "referral and consultation"/

37. exp "task performance and analysis"/

38. 11 or 12 or 13 or 14 or 15 or 16 or 17 or 18 or 19 or 20 or 21 or 22 or 23 or 24 or 25 or 26 or 27 or 28 or 29 or 30 or 31 or 32 or 33 or 34 or 35 or 36 or 37

39. exp "appointments and schedules"/ or Biological Markers/ or Hypertension/ or exp Cholesterol/ or exp Hypercholesterolemia/ or exp chronic disease/ or exp "costs and cost analysis"/ or exp "Delivery of Health Care"/ or exp diagnostic tests, routine/ or exp hemoglobin a/ or exp hemoglobin a, glycosylated/ or exp guideline adherence/ or exp health resources/ or exp Health Services/ or exp health status indicators/ or exp health status/ or exp job satisfaction/ or exp mortality/ or exp "outcome and process assessment (health care)"/ or exp "outcome assessment (health care)"/ or pain/ or exp patient satisfaction/ or exp personal satisfaction/ or exp Prescriptions/ or exp professional role/ or exp "quality of health care"/ or exp "referral and consultation"/ or exp "task performance and analysis"/ or exp "task performance and analysis"/

40. (Appointment or Appointments or Attendance or Biomarker or Biomarkers or Hypertension or Care Process or Care Processes or Cholesterol or Hypercholesterolemia or Chronic Condition or Chronic Conditions or Chronic Disease or Chronic Diseases or Chronic Illness or Chronic Illnesses or Clinical Outcome or Clinical Outcomes or Clinical Parameter or Clinical Parameters or Compliance or Compliant or Consultation or Consultations or Consumer Behavior or Consumer Behaviors or Consumer Behaviour or Consumer Behaviours or Cost Analyses or Cost Analysis or Cost or Costs or Death or Deaths or Delivery Of Health Care or Delivery Of Healthcare or Diagnostic Test or Diagnostic Testing or Diagnostic Tests or Economic Analyses or Economic Analysis or Economic Evaluation or Economic Evaluations or Emergency Service or Emergency Services or Follow Up Appointment or Follow Up Appointments or Follow Up Visit or Follow Up Visits or Glycated Haemoglobin A or Glycated Hemoglobin A or Guideline Adherence or HbA1C or Hdl or Health Care or Health Planning or Health Related Quality Of Life or Health Resource or Health Resources or Health Service or Health Services or Health

Status or Healthcare Delivery or Healthcare Process Assessment or Healthcare Quality or Healthcare or High Density Lipoprotein or Hospital Admission or Hospital Admissions or Hospital Referral or Hospital Referrals or Hrqol or Hypertension or Indicator or Indicators or Job Satisfaction or Ldl or Lipid Profile or Lipid Profiles or Low Density Lipoprotein or Manpower or Mortalities or Mortality or Outcome Assessment or Outcome Assessments or Pain or Pains or Patient Satisfaction or Personal Satisfaction or Prefer or Preference or Preferences or Preferred or Prescribe or Prescribed or Prescription or Prescriptions or Process Of Care or Processes Of Care or Professional Role or Professional Roles or Qol or Quality Indicator or Quality Indicators or Quality Of Health Care or Quality Of Healthcare or Quality Of Life or Referral or Referrals or Return Appointment or Return Appointments or Return Visit or Return Visits or Routine Diagnostic or Routine Test or Routine Testing or Routine Tests or Satisfaction or Satisfactory or Satisfied or Schedule or Schedules or Task Analyses or Task Analysis or Task Performance or Treatment Adherence or Treatment Adherent or Treatment Compliance or Treatment Compliant or Wellness).ab,ti.

41. 39 or 40

42. exp anthropometry/

43. exp body weight/

44. diet/

45. exp health education/

46. exp health promotion/

47. exp mental health/

48. exp nutrition assessment/

49. exp nutritional status/

50. exp poverty/

51. exp preventive health services/

52. exp smoking cessation/

53. exp smoking/

54. exp teaching/

55. exp Tobacco Smoke Pollution/

56. exp "Tobacco Use"/

57. 42 or 43 or 44 or 45 or 46 or 47 or 48 or 49 or 50 or 51 or 52 or 53 or 54 or 55 or 56

58. exp anthropometry/ or exp body weight/ or diet/ or exp health education/ or exp health promotion/ or exp mental health/ or exp nutrition assessment/ or exp nutritional status/ or exp poverty/ or exp preventive health services/ or exp smoking cessation/ or exp smoking/ or exp teaching/ or exp Tobacco Smoke Pollution/ or exp "Tobacco Use"/

59. (Anthropometric or Anthropometrical or Anthropometrically or Anthropometrics or Anthropometry or Body Weight or Condition Specific or Culturally Competent Care or Culturally Diverse or Diet or Dietary or Dietetical or Dietetically or Dietetics or Diets or Emotional Health or Exercise or Food Regimen or Food Regimens

or Health Education or Health Prevention or Health Promotion or Healthy Habit or Healthy Habits or Healthy Life or Healthy Life Habits or Healthy Living or Life Style or Lifestyle or Low Income or Mental Health or Nutrition Assessment or Nutrition or Nutritional Status or Nutritional or Physical Activity or Poverty or Preventive Health Service or Preventive Health Services or Promotion of Health or Promotion or Quit Smoking or Quitting Smoking or Second Hand Smoke or Smoke or Smoked or Smoker or Smokers or Smokes or Smoking or Socioeconomic Status or Stop Smoking or Stopping Smoking or Stress Level or Stress Levels or Taught or Teach or Teaching or Tobacco or Weight).ab,ti.

60. 58 or 59

61. exp adolescent/

62. exp child development/

63. exp Human Development/

64. immunization/

65. exp maternal health services/

66. exp pediatrics/

67. exp primary prevention/

68. exp secondary prevention/

69. Sexually Transmitted Diseases/

70. exp vaccination/

71. 61 or 62 or 63 or 64 or 65 or 66 or 67 or 68 or 69 or 70

72. exp adolescent/ or exp child development/ or exp Human Development/ or immunization/ or exp maternal health services/ or exp pediatrics/ or exp primary prevention/ or exp secondary prevention/ or Sexually Transmitted Diseases/ or exp vaccination/

73. (Adolescence or Adolescent or Adolescents or Child Development or Childhood or Healthy Development or Human Development or Immunisation or Immunise or Immunised or Immunises or Immunization or Immunize or Immunized or Immunizes or Laboratory Test or Maternal Health Service or Maternal Health Services or Paediatric or Paediatrics or Pediatric or Pediatrics or Pregnancy or Prevent or Prevented or Prevention or Prevents or Primary Prevention or Secondary Prevention or Sexually Transmitted or Vaccinate or Vaccinated or Vaccinates or Vaccination or Vaccine or Vaccines or Youth or Youths).ab,ti.

74. (Adolescence or Adolescent or Adolescents or Child Development or Childhood or Healthy Development or Human Development or Immunisation or Immunise or Immunised or Immunises or Immunization or Immunize or Immunized or Immunizes or Laboratory Test or Maternal Health Service or Maternal Health Services or Paediatric or Paediatrics or Pediatric or Pediatrics or Pregnancy or Prevent or Prevented or Prevention or Prevents or Primary Prevention or Secondary Prevention or Sexually Transmitted or Vaccinate or Vaccinated or Vaccinates or Vaccination or Vaccine or Vaccines or Youth or Youths).ab,ti.

75. 72 or 74

76. acute coronary syndrome/

77. exp Adjustment Disorders/

78. exp alcohol drinking/

79. exp Alcohol-Related Disorders/

80. exp Anxiety Disorders/

81. exp blood glucose/

82. exp hyperglycemia/

83. exp Hypoglycemia/

84. cardiovascular diseases/

85. coronary disease/

86. dementia/

87. exp depressive disorder/

88. Diabetes Mellitus/

89. exp diabetes, gestational/

90. exp Diabetic Foot/

91. exp health care evaluation mechanisms/

92. Heart Failure/

93. exp myocardial infarction/

94. exp mental disorders/

95. neoplasms/

96. exp pain management/

97. pain/

98. exp papanicolaou test/

99. exp physical examination/

100. exp Pulmonary Disease, Chronic Obstructive/

101. exp Quality Indicators, Health Care/

102. exp Rehabilitation/

103. exp self care/

104. exp self efficacy/

105. Stroke/

106. Acute Coronary Syndrome/ or exp Adjustment Disorders/ or exp Alcohol Drinking/ or exp Alcohol-Related Disorders/ or exp Anxiety Disorders/ or exp Blood Glucose/ or exp Hyperglycemia/ or exp Hypoglycemia/ or Cardiovascular Diseases/ or coronary disease/ or Dementia/ or exp Depressive Disorder/ or

Diabetes Mellitus/ or exp Diabetes, Gestational/ or Diabetic Foot/ or exp Health Care Evaluation Mechanisms/ or Heart Failure/ or exp Myocardial Infarction/ or exp Mental Disorders/ or Neoplasms/ or exp Pain Management/ or Pain/ or exp Papanicolaou Test/ or exp Physical Examination/ or exp Pulmonary Disease, Chronic Obstructive/ or exp Quality Indicators, Health Care/ or exp Rehabilitation/ or exp Self Care/ or exp Self Efficacy/ or Stroke/

107. acute coronary syndrome/ or exp Adjustment Disorders/ or exp alcohol drinking/ or exp Alcohol-Related Disorders/ or exp Anxiety Disorders/ or exp blood glucose/ or exp hyperglycemia/ or exp Hypoglycemia/ or cardiovascular diseases/ or coronary disease/ or dementia/ or exp depressive disorder/ or Diabetes Mellitus/ or exp diabetes, gestational/ or exp Diabetic Foot/ or exp health care evaluation mechanisms/ or Heart Failure/ or exp myocardial infarction/ or exp mental disorders/ or neoplasms/ or exp pain management/ or pain/ or exp papanicolaou test/ or exp physical examination/ or exp Pulmonary Disease, Chronic Obstructive/ or exp Quality Indicators, Health Care/ or exp Rehabilitation/ or exp self care/ or exp self efficacy/ or Stroke/

108. (Acs or Acss or Acute Coronary Syndrome or Acute Coronary Syndromes or Adjustment Disorder or Adjustment Disorders or Alcohol Consumption or Alcohol Drinking or Alcohol Related Disorder or Alcohol Related Disorders or Alcoholic or Alcoholism or Anxiety or Anxious or Blood Glucose or Blood Sugar or Blood Sugars or Hyperglycemia or Hypoglycemia or Cancer or Cardiovascular Disease or Cardiovascular Diseases or Cerebrovascular Accident or Cerebrovascular Accidents or Chf or Chronic Obstructive Pulmonary Disease or Chronic Obstructive Pulmonary Disorder or Chronic Obstructive Pulmonary Disorders or Complication or Complications or Congestive Heart Failure or Copd or Coronary Artery Disease or Coronary Artery Diseases or Coronary Disease or Coronary Diseases or Cva or Cvas or Cvd or Cvds or Dementia or Dementias or Depressed or Depression or Depressive Disorder or Depressive Disorders or Depressive or Diabetes Mellitus or Diabetes or Diabetic Foot or Foot Complication or Foot Complications or Gestational Diabetes or Glycaemia or Glycaemic or Glycemia or Glycemic or Health Care Evaluation Mechanisms or Health Care Quality Indicator or Health Care Quality Indicators or Health Information Interoperability or Healthcare Evaluation Mechanisms or Healthcare Quality Indicator or Healthcare Quality Indicators or Heart Failure or Interoperability or Interoperable or Managed Pain or Management Of Pain or Managing Pain or Mental Disorder or Mental Disorders or Mental Health or Mental Illness or Mental Illnesses or Mentally Healthy or Mentally Ill or Myocardial Infarction or Neoplasm or Neoplasms or Oncologist or Oncologists or Oncology or Pain Management or Pain or Pains or Pap Test or Pap Tests or Papanicolaou Test or Papanicolaou Tests or Patient Reported Experience Measure or Patient Reported Experience Measures or Patient Reported Outcome Measure or Patient Reported Outcome Measures or Physical Exam or Physical Examination or Physical Examinations or Physical Exams or Prem or Prems or Prom or Promotion or Proms or Psycho Social or Psychosocial or Quality Indicator or Quality indicators Of Health Care Quality Indicators or Rehabilitation or Self Care or Self Efficacy or Stroke or Strokes).ab,ti.

109. 107 or 108

110. exp Allied Health Personnel/

111. exp Ambulatory Care Information Systems/

112. ambulatory care/

113. exp "appointments and schedules"/

114. exp clinical competence/

115. exp patient education as topic/

116. exp evidence-based practice/

117. exp harm reduction/

118. exp health behavior/

119. exp health care costs/

120. exp "health care economics and organizations"/

121. exp "health care facilities, manpower, and services"/

122. exp "health care quality, access, and evaluation"/

123. exp health knowledge, attitudes, practice/

124. exp health plan implementation/

125. exp health services accessibility/

126. exp healthcare disparities/

127. exp Hospital Shared Services/

128. exp house calls/

129. exp "length of stay"/

130. exp office visits/

131. exp "patient acceptance of health care"/

132. exp patient admission/

133. exp Patient Advocacy/

134. exp Prescriptions/

135. exp "referral and consultation"/

136. 110 or 111 or 112 or 113 or 114 or 115 or 116 or 117 or 118 or 119 or 120 or 121 or 122 or 123 or 124 or 125 or 126 or 127 or 128 or 129 or 130 or 131 or 132 or 133 or 134 or 135

137. exp Allied Health Personnel/ or exp Ambulatory Care Information Systems/ or ambulatory care/ or exp "appointments and schedules"/ or exp clinical competence/ or exp patient education as topic/ or exp evidence-based practice/ or exp harm reduction/ or exp health behavior/ or exp health care costs/ or exp "health care economics and organizations"/ or exp "health care facilities, manpower, and services"/ or exp "health care quality, access, and evaluation"/ or exp health knowledge, attitudes, practice/ or exp health plan implementation/ or exp health services accessibility/ or exp healthcare disparities/ or exp Hospital Shared Services/ or exp house calls/ or exp "length of stay"/ or exp office visits/ or exp "patient acceptance of health care"/ or exp patient admission/ or exp Patient Advocacy/ or exp Prescriptions/ or exp "referral and consultation"/

138. (Access To Care or Access To Health Care or Access To Health Services or Access To Healthcare or Accessibility Of Health Services or Allied Health Personnel or Ambulatory Care Information System or Ambulatory Care Information Systems or Ambulatory Care or Appointment or Appointments or Care Access or Clinical Competence or Clinical Competencies or Clinical Competency or Consultation or Consultations or Cost or Costs or Duration Of Therapy or Patient Education or Patient Teaching or Evaluated Health Care or Evaluated Healthcare or Evaluating Health Care or Evaluating Healthcare or Evaluation Of Health Care or Evaluations Of Health Care or Harm Prevention or Harm Reduction or Health Attitude or Health Attitudes or Health Behavior or

Health Behaviors or Health Behaviour or Health Behaviours or HealthCare or Health Care or Health Knowledge or Health Personnel or Health Plan Implementation or Health Service Accessibility or Health Services Accessibility or Healthy Environment or Healthy Environments or Hospital Shared Services or House Call or House Calls or Indirect Cost or indirect Costs or Knowledge Of Health or Length Of Stay or Length Of Therapy or Lengths Of Stay or Los or Office Visit or Office Visits or Patient Acceptance or Patient Admission or Patient Admissions or Patient Advocacy or Prescription or Prescriptions or Prevention Of Harm or Quality Health Care or Quality Healthcare or Reduction Of Harm or Referral or Referrals or Return Visit or Return Visits or Schedule or Scheduled or Schedules or Therapy Duration or Therapy Durations or Therapy Length or Universal Health Care or Universal Healthcare or Unplanned).ab,ti.

139. 137 or 138

140. exp "Delivery of Health Care"/

141. exp health literacy/

142. exp health status disparities/

143. exp income/

144. exp Insurance Coverage/

145. exp "international classification of functioning, disability and health"/

146. exp marital status/

147. exp Minority Health/

148. exp oceanic ancestry group/

149. exp patient selection/

150. exp population dynamics/

151. exp Population Groups/

152. exp poverty areas/

153. exp refusal to treat/

154. religion/

155. exp social discrimination/

156. exp socioeconomic factors/

157. exp treatment failure/

158. 140 or 141 or 142 or 143 or 144 or 145 or 146 or 147 or 148 or 149 or 150 or 151 or 152 or 153 or 154 or 155 or 156 or 157

159. exp "Delivery of Health Care"/ or exp health literacy/ or exp health status disparities/ or exp income/ or exp Insurance Coverage/ or exp "international classification of functioning, disability and health"/ or exp marital status/ or exp Minority Health/ or exp oceanic ancestry group/ or exp patient selection/ or exp population dynamics/ or exp Population Groups/ or exp poverty areas/ or exp refusal to treat/ or religion/ or exp social discrimination/ or exp socioeconomic factors/ or exp treatment failure/

160. (Aboriginal or Communication Barriers or Delivery Of Health Care or Delivery Of Healthcare or Disability Evaluation or Discrimination or Equity or Frailty or Gender Minorities or Gender Minority or Global Health or Health Care Delivery or Health Care Disparities or Health Care Disparity or Health Equity or Health Literacy or Health Status Disparities or Health Status Disparity or Healthcare Delivery or Healthcare Disparities or Healthcare Disparity or Income or Incomes or Inequal or Inequalities or Inequality or Insurance Cover or Insurance Coverage or Insurance Coverages or Insurance Covered or "International Classification Of Functioning Disability and Health" or Marital Status or Minority Health or Oceanic Ancestry Group or Oceanic Ancestry Groups or Patient Selection or Population Dynamics or Population Group or Population Groups or Poverty Area or Poverty Areas or Poverty or Refusal To Treat or Religion or Religions or Safety Net or Sexual Minorities or Sexual Minority or Sexual Orientation or Sexual Orientations or Social Discrimination or Social Discriminations or Social Support or Treatment Failure or Treatment Failures or Treatment Preference or Treatment Preferences or Vulnerabilities or Vulnerability or Vulnerable).ab,ti.

161. 159 or 160

162. 41 or 60 or 75 or 109 or 139 or 161

163. exp meta-analysis/

164. Meta Analy*.tw.

165. Metanaly*.tw.

166. Metaanaly*.tw.

167. Met Analy*.tw.

168. systematic.sb.

169. ((overview$ or review or synthesis or summary or cochrane or analysis) and (reviews or meta-analyses or articles)).ti.

170. meta-analysis.pt.

171. (meta-review or metareview).ti,ab.

172. ((overview$ or reviews) and (systematic or cochrane)).ti.

173. (reviews adj2 (meta or published or quality or included or summar$)).ab.

174. cochrane reviews.ab.

175. (evidence and (reviews or meta-analyses)).ti.

176. 3 and 10 and 162

6. CINAHL

S252	S251<br>Limiters - Published Date: 20100101-20221231; Exclude MEDLINE records
S251	S47 AND S57 AND S244 AND S248
S250	S49 AND S58 AND S247 AND S249
S249	S230 OR S231 OR S232 OR S233 OR S234 OR S235 OR S236 OR S237 OR S238 OR S239 OR S240 OR S241 OR S242 OR S243<br>Limiters - Published Date: 20100101-20221231; Exclude MEDLINE records
S248	S94 OR S123 OR S134 OR S168 OR S203 OR S227
S247	S95 OR S124 OR S135 OR S169 OR S204 OR S228
S246	S229 AND S244<br>Limiters - Published Date: 20100101-20221231; Exclude MEDLINE records
S245	S229 AND S244
S244	S230 OR S231 OR S232 OR S233 OR S234 OR S235 OR S236 OR S237 OR S238 OR S239 OR S240 OR S241 OR S242 OR S243
S243	TI (evidence) AND (TI (reviews OR meta-analyses))
S242	AB (cochrane reviews)
S241	AB (reviews N2 (meta OR published OR quality OR included OR summar*))
S240	(TI (overview* OR reviews)) AND (TI (systematic OR cochrane)))
S239	(TI metareview OR AB metareview)
S238	(TI meta-review OR AB meta-review)
S237	(TI (overview* OR review OR synthesis OR summary OR cochrane OR analysis)) AND (TI (reviews OR meta-analyses OR articles)))
S236	(TX (meta analy* OR metanaly* OR metaanaly* OR met analy*))
S235	(MH "Systematic Review")
S234	(MH "Meta Analysis")
S233	PT (meta analysis OR meta-analysis)

S232	SU ((Systematic* N3 (Review* OR Overview*)) OR (Methodologic* N3 (Review* OR Overview*)))
S231	AB ((Systematic* N3 (Review* OR Overview*)) OR (Methodologic* N3 (Review* OR Overview*)))
S230	TI ((Systematic* N3 (Review* OR Overview*)) OR (Methodologic* N3 (Review* OR Overview*)))
S229	S49 AND S58 AND (S95 OR S124 OR S135 OR S169 OR S204 OR S228)<br>Limiters - Published Date: 20100101-20221231; Exclude MEDLINE records
S228	S205 OR S206 OR S207 OR S208 OR S209 OR S210 OR S211 OR S212 OR S213 OR S214 OR S215 OR S216 OR S217 OR S218 OR S219 OR S220 OR S221 OR S222 OR S223 OR S224 OR S225 OR S226<br>Limiters - Published Date: 20100101-20221231; Exclude MEDLINE records
S227	S205 OR S206 OR S207 OR S208 OR S209 OR S210 OR S211 OR S212 OR S213 OR S214 OR S215 OR S216 OR S217 OR S218 OR S219 OR S220 OR S221 OR S222 OR S223 OR S224 OR S225 OR S226
S226	(MH "Health Care Delivery+")
S225	(MH "Frailty Syndrome")
S224	(MH "World Health")
S223	(MH "Health Literacy")
S222	(MH "Health Status Disparities")
S221	(MH "Healthcare Disparities")
S220	(MH "Income+")
S219	(MH "Insurance Coverage")
S218	(MH "International Classification of Functioning, Disability, and Health")
S217	(MH "Marital Status+")
S216	(MH "Minority Groups")
S215	(MH "Ethnic Groups+")
S214	(MH "Patient Selection")
S213	(MH "Population+")
S212	(MH "Population Characteristics+")
S211	(MH "Poverty Areas")

S210	(MH "Refusal to Treat")	
S209	(MH "Religion and Religions")	
S208	(MH "Discrimination+")	
S207	(MH "Socioeconomic Factors+")	
S206	(MH "Treatment Failure")	
S205	(TI (Aboriginal OR Communication Barriers OR Delivery of Health Care OR Delivery of HealthCare OR Disability Evaluation OR Discrimination OR equity OR Frailty OR Gender Minorities OR Gender Minority OR Global Health OR Health Care Delivery OR Health care Disparities OR Health Care Disparity OR Health Equity OR Health Literacy OR Health Status Disparities OR Health Status Disparity OR HealthCare Delivery OR Healthcare Disparities OR Healthcare Disparity OR Income OR Incomes OR inequal OR inequalities OR inequality OR Insurance cover OR Insurance coverage OR Insurance coverages OR Insurance covered OR "International Classification of Functioning, Disability and Health" OR Marital status OR Minority Health OR Oceanic Ancestry Group OR Oceanic Ancestry Groups OR Patient selection OR population dynamics OR Population group OR Population groups OR Poverty area OR Poverty areas OR Poverty OR Refusal to Treat OR Religion OR Religions OR safety net OR Sexual Minorities OR Sexual Minority OR Sexual Orientation OR Sexual Orientations OR Social Discrimination OR Social Discriminations OR Social support OR treatment failure OR treatment failures OR Treatment preference OR Treatment preferences OR Vulnerabilities OR Vulnerability OR Vulnerable)) OR (AB (Aboriginal OR Communication Barriers OR Delivery of Health Care OR Delivery of HealthCare OR Disability Evaluation OR Discrimination OR equity OR Frailty OR Gender Minorities OR Gender Minority OR Global Health OR Health Care Delivery OR Health care Disparities OR Health Care Disparity OR Health Equity OR Health Literacy OR Health Status Disparities OR Health Status Disparity OR HealthCare Delivery OR Healthcare Disparities OR Healthcare Disparity OR Income OR Incomes OR inequal OR inequalities OR inequality OR Insurance cover OR Insurance coverage OR Insurance coverages OR Insurance covered OR "International Classification of Functioning, Disability and Health" OR Marital status OR Minority Health OR Oceanic Ancestry Group OR Oceanic Ancestry Groups OR Patient selection OR population dynamics OR Population group OR Population groups OR Poverty area OR Poverty areas OR Poverty OR Refusal to Treat OR Religion OR Religions OR safety net OR Sexual Minorities OR Sexual Minority OR Sexual Orientation OR Sexual Orientations OR Social Discrimination OR Social Discriminations OR Social support OR treatment failure OR treatment failures OR Treatment preference OR Treatment preferences OR Vulnerabilities OR Vulnerability OR Vulnerable))	
S204	S170 OR S171 OR S172 OR S173 OR S174 OR S175 OR S176 OR S177 OR S178 OR S179 OR S180 OR S181 OR S182 OR S183 OR S184 OR S185 OR S186 OR S187 OR S188 OR S189 OR S190 OR S191 OR S192 OR S193 OR S194 OR S195 OR S196 OR S197 OR S198 OR S199 OR S200 OR S201 OR S202<br>Limiters - Published Date: 20100101-20221231; Exclude MEDLINE records	
S203	S170 OR S171 OR S172 OR S173 OR S174 OR S175 OR S176 OR S177 OR S178 OR S179 OR S180 OR S181 OR S182 OR S183 OR S184 OR S185 OR S186 OR S187 OR S188 OR S189 OR S190 OR S191 OR S192 OR S193 OR S194 OR S195 OR S196 OR S197 OR S198 OR S199 OR S200 OR S201 OR S202	

S202	(MH "Allied Health Personnel+")
S201	(MH "Ambulatory Care Information Systems")
S200	(MH "Ambulatory Care")
S199	(MH "Appointments and Schedules+")
S198	(MH "Appointment and Scheduling Information Systems")
S197	(MH "Clinical Competence+")
S196	(MH "Treatment Duration")
S195	(MH "Patient Education+")
S194	(MH "Professional Practice, Evidence-Based+")
S193	(MH "Harm Reduction")
S192	(MH "Health Behavior+")
S191	(MH "Health Care Costs+")
S190	(MH "Economics, Organizations, Control+")
S189	(MH "Facilities, Manpower and Services+")
S188	(MH "Attitude of Health Personnel+")
S187	(MH "Health Knowledge")
S186	(MH "Attitude to Health")
S185	(MH "Professional Practice+")
S184	(MH "Health and Welfare Planning+")
S183	(MH "Health Services Accessibility+")
S182	(MH "Healthcare Disparities")
S181	(MH "Shared Services, Health Care")
S180	(MH "Home Visits")
S179	(MH "Length of Stay")
S178	(MH "Office Visits")

S177	(MH "Patient Attitudes")
S176	(MH "Patient Admission")
S175	(MH "Patient Advocacy")
S174	(MH "Prescriptions, Non-Drug")
S173	(MH "Prescriptions, Drug+")
S172	(MH "Referral and Consultation+")
S171	(MH "Universal Health Care")
S170	(TI (Access to care OR Access to health care OR Access to Health Services OR Access to healthcare OR Accessibility of Health Services OR Allied Health Personnel OR Ambulatory Care Information System OR Ambulatory Care Information Systems OR ambulatory care OR Appointment OR Appointments OR care access OR Clinical Competence OR Clinical Competencies OR Clinical Competency OR Consultation OR Consultations OR Cost OR Costs OR Duration of Therapy OR Patient Education as Topic OR Patient teaching OR Evaluated Health Care OR Evaluated HealthCare OR Evaluating Health Care OR Evaluating HealthCare OR Evaluation of Health Care OR Evaluations of Health Care OR Harm prevention OR Harm Reduction OR Health Attitude OR Health Attitudes OR Health Behavior OR Health Behaviors OR Health Behaviour OR Health Behaviours OR HealthCare OR Health Care OR Health Knowledge OR Health Personnel OR Health Plan Implementation OR Health Service Accessibility OR Health Services Accessibility OR healthy environment OR healthy environments OR Hospital Shared Services OR House Call OR House Calls OR indirect cost OR indirect costs OR Knowledge of Health OR Length of Stay OR Length of therapy OR Lengths of Stay OR LOS OR Office Visit OR Office Visits OR Patient Acceptance OR patient admission OR patient admissions OR Patient Advocacy OR Prescription OR Prescriptions OR Prevention of harm OR Quality Health Care OR Quality HealthCare OR Reduction of harm OR Referral OR Referrals OR return visit OR return visits OR Schedule OR Scheduled OR Schedules OR Therapy duration OR Therapy durations OR Therapy length OR Universal Health Care OR Universal HealthCare OR unplanned)) OR (AB (Access to care OR Access to health care OR Access to Health Services OR Access to healthcare OR Accessibility of Health Services OR Allied Health Personnel OR Ambulatory Care Information System OR Ambulatory Care Information Systems OR ambulatory care OR Appointment OR Appointments OR care access OR Clinical Competence OR Clinical Competencies OR Clinical Competency OR Consultation OR Consultations OR Cost OR Costs OR Duration of Therapy OR Patient Education as Topic OR Patient teaching OR Evaluated Health Care OR Evaluated HealthCare OR Evaluating Health Care OR Evaluating HealthCare OR Evaluation of Health Care OR Evaluations of Health Care OR Harm prevention OR Harm Reduction OR Health Attitude OR Health Attitudes OR Health Behavior OR Health Behaviors OR Health Behaviour OR Health Behaviours OR HealthCare OR Health Care OR Health Knowledge OR Health Personnel OR Health Plan Implementation OR Health Service Accessibility OR Health Services Accessibility OR healthy environment OR healthy environments OR Hospital Shared Services OR House Call OR House Calls OR indirect cost OR indirect costs OR Knowledge of Health OR Length of Stay OR Length of therapy OR Lengths of Stay OR LOS OR Office Visit OR Office Visits OR Patient Acceptance OR patient admission OR patient admissions OR Patient Advocacy OR Prescription OR Prescriptions OR Prevention of harm OR Quality Health Care OR Quality HealthCare OR Reduction of harm OR Referral OR Referrals OR return visit OR return visits OR Schedule OR Scheduled OR Schedules

	OR Therapy duration OR Therapy durations OR Therapy length OR Universal Health Care OR Universal HealthCare OR unplanned))
S169	S136 OR S137 OR S138 OR S139 OR S140 OR S141 OR S142 OR S143 OR S144 OR S145 OR S146 OR S147 OR S148 OR S149 OR S150 OR S151 OR S152 OR S153 OR S154 OR S155 OR S156 OR S157 OR S158 OR S159 OR S160 OR S161 OR S162 OR S163 OR S164 OR S165 OR S166 OR S167<br>Limiters - Published Date: 20100101-20221231; Exclude MEDLINE records
S168	S136 OR S137 OR S138 OR S139 OR S140 OR S141 OR S142 OR S143 OR S144 OR S145 OR S146 OR S147 OR S148 OR S149 OR S150 OR S151 OR S152 OR S153 OR S154 OR S155 OR S156 OR S157 OR S158 OR S159 OR S160 OR S161 OR S162 OR S163 OR S164 OR S165 OR S166 OR S167
S167	(MH "Acute Coronary Syndrome")
S166	(MH "Adjustment Disorders+")
S165	(MH "Alcohol Drinking+")
S164	(MH "Alcohol-Related Disorders+")
S163	(MH "Anxiety Disorders+")
S162	(MH "Blood Glucose")
S161	(MH "Hyperglycemia")
S160	(MH "Hypoglycemia+")
S159	(MH "Cardiovascular Diseases")
S158	(MH "Coronary Disease")
S157	(MH "Dementia")
S156	(MH "Affective Disorders+")
S155	(MH "Diabetes Mellitus")
S154	(MH "Diabetes Mellitus, Gestational")
S153	(MH "Diabetic Foot")
S152	(MH "Quality of Health Care+")
S151	(MH "Heart Failure")
S150	(MH "Myocardial Infarction+")

S149	(MH "Mental Disorders+")	
S148	(MH "Neoplasms")	
S147	(MH "Pain Management")	
S146	(MH "Pain")	
S145	(MH "Cervical Smears+")	
S144	(MH "Patient-Reported Outcomes+")	
S143	(MH "Physical Examination+")	
S142	(MH "Pulmonary Disease, Chronic Obstructive+")	
S141	(MH "Quality of Health Care+")	
S140	(MH "Rehabilitation+")	
S139	(MH "Self Care+")	
S138	(MH "Self-Efficacy")	
S137	(MH "Stroke")	
S136	(TI (ACS OR ACSs OR Acute Coronary Syndrome OR Acute Coronary Syndromes OR Adjustment Disorder OR Adjustment Disorders OR Alcohol consumption OR Alcohol Drinking OR Alcohol Related Disorder OR Alcohol Related Disorders OR Alcoholic OR Alcoholism OR Anxiety OR anxious OR Blood Glucose OR Blood sugar OR Blood sugars OR Hyperglycemia OR Hypoglycemia OR Cancer OR Cardiovascular disease OR Cardiovascular diseases OR cerebrovascular accident OR cerebrovascular accidents OR CHF OR Chronic Obstructive Pulmonary Disease OR Chronic Obstructive Pulmonary Disorder OR Chronic Obstructive Pulmonary Disorders OR complication OR complications OR Congestive heart failure OR copd OR Coronary artery disease OR Coronary artery diseases OR Coronary Disease OR Coronary Diseases OR CVA OR CVAs OR CVD OR CVDs OR Dementia OR Dementias OR depressed OR depression OR Depressive Disorder OR Depressive Disorders OR depressive OR Diabetes Mellitus OR Diabetes OR Diabetic Foot OR foot complication OR foot complications OR gestational diabetes OR glycaemia OR glycaemic OR glycemia OR glycemic OR Health Care Evaluation Mechanisms OR Health Care Quality Indicator OR Health Care Quality Indicators OR Health Information Interoperability OR HealthCare Evaluation Mechanisms OR Healthcare Quality Indicator OR Healthcare Quality Indicators OR Heart Failure OR Interoperability OR Interoperable OR managed pain OR management of pain OR managing pain OR Mental Disorder OR Mental Disorders OR mental health OR Mental illness OR Mental illnesses OR mentally healthy OR Mentally ill OR Myocardial Infarction OR Neoplasm OR Neoplasms OR Oncologist OR Oncologists OR Oncology OR pain management OR pain OR pains OR Pap test OR Pap tests OR Papanicolaou Test OR Papanicolaou Tests OR Patient Reported Experience Measure OR Patient Reported Experience Measures OR Patient Reported Outcome Measure OR Patient Reported Outcome Measures OR Physical Exam OR Physical Examination OR Physical Examinations OR Physical Exams OR PREM OR PREMs OR PROM OR promotion OR PROMs OR psycho social OR psychosocial OR Quality	

	Indicator OR Quality indicators of health care OR Quality Indicators OR Rehabilitation OR self care OR Self Efficacy OR Stroke OR Strokes)) OR (AB (ACS OR ACSs OR Acute Coronary Syndrome OR Acute Coronary Syndromes OR Adjustment Disorder OR Adjustment Disorders OR Alcohol consumption OR Alcohol Drinking OR Alcohol Related Disorder OR Alcohol Related Disorders OR Alcoholic OR Alcoholism OR Anxiety OR anxious OR Blood Glucose OR Blood sugar OR Blood sugars OR Hyperglycemia OR Hypoglycemia OR Cancer OR Cardiovascular disease OR Cardiovascular diseases OR cerebrovascular accident OR cerebrovascular accidents OR CHF OR Chronic Obstructive Pulmonary Disease OR Chronic Obstructive Pulmonary Disorder OR Chronic Obstructive Pulmonary Disorders OR complication OR complications OR Congestive heart failure OR copd OR Coronary artery disease OR Coronary artery diseases OR Coronary Disease OR Coronary Diseases OR CVA OR CVAs OR CVD OR CVDs OR Dementia OR Dementias OR depressed OR depression OR Depressive Disorder OR Depressive Disorders OR depressive OR Diabetes Mellitus OR Diabetes OR Diabetic Foot OR foot complication OR foot complications OR gestational diabetes OR glycaemia OR glycaemic OR glycemia OR glycemic OR Health Care Evaluation Mechanisms OR Health Care Quality Indicator OR Health Care Quality Indicators OR Health Information Interoperability OR HealthCare Evaluation Mechanisms OR Healthcare Quality Indicator OR Healthcare Quality Indicators OR Heart Failure OR Interoperability OR Interoperable OR managed pain OR management of pain OR managing pain OR Mental Disorder OR Mental Disorders OR mental health OR Mental illness OR Mental illnesses OR mentally healthy OR Mentally ill OR Myocardial Infarction OR Neoplasm OR Neoplasms OR Oncologist OR Oncologists OR Oncology OR pain management OR pain OR pains OR Pap test OR Pap tests OR Papanicolaou Test OR Papanicolaou Tests OR Patient Reported Experience Measure OR Patient Reported Experience Measures OR Patient Reported Outcome Measure OR Patient Reported Outcome Measures OR Physical Exam OR Physical Examination OR Physical Examinations OR Physical Exams OR PREM OR PREMs OR PROM OR promotion OR PROMs OR psycho social OR psychosocial OR Quality Indicator OR Quality indicators of health care OR Quality Indicators OR Rehabilitation OR self care OR Self Efficacy OR Stroke OR Strokes))
S135	S125 OR S126 OR S127 OR S128 OR S129 OR S130 OR S131 OR S132 OR S133<br>Limiters - Published Date: 20100101-20221231; Exclude MEDLINE records
S134	S125 OR S126 OR S127 OR S128 OR S129 OR S130 OR S131 OR S132 OR S133
S133	(MH "Adolescence+")
S132	(MH "Child Development")
S131	(MH "Human Development+")
S130	(MH "Immunization+")
S129	(MH "Maternal Health Services+")
S128	(MH "Pediatrics+")
S127	(MH "Preventive Health Care+")
S126	(MH "Sexually Transmitted Diseases")

S125	(TI (adolescence OR adolescent OR adolescents OR Child development OR Childhood OR healthy development OR Human development OR Immunisation OR Immunise OR Immunised OR Immunises OR Immunization OR Immunize OR Immunized OR Immunizes OR laboratory test OR Maternal Health Service OR Maternal Health Services OR Paediatric OR Paediatrics OR Pediatric OR Pediatrics OR pregnancy OR Prevent OR Prevented OR Prevention OR Prevents OR Primary Prevention OR Secondary Prevention OR sexually transmitted OR Vaccinate OR Vaccinated OR Vaccinates OR Vaccination OR Vaccine OR Vaccines OR youth OR youths)) OR (AB (adolescence OR adolescent OR adolescents OR Child development OR Childhood OR healthy development OR Human development OR Immunisation OR Immunise OR Immunised OR Immunises OR Immunization OR Immunize OR Immunized OR Immunizes OR laboratory test OR Maternal Health Service OR Maternal Health Services OR Paediatric OR Paediatrics OR Pediatric OR Pediatrics OR pregnancy OR Prevent OR Prevented OR Prevention OR Prevents OR Primary Prevention OR Secondary Prevention OR sexually transmitted OR Vaccinate OR Vaccinated OR Vaccinates OR Vaccination OR Vaccine OR Vaccines OR youth OR youths))	
S124	S96 OR S97 OR S98 OR S99 OR S100 OR S101 OR S102 OR S103 OR S104 OR S105 OR S106 OR S107 OR S108 OR S109 OR S110 OR S111 OR S112 OR S113 OR S114 OR S115 OR S116 OR S117 OR S118 OR S119 OR S120 OR S121 OR S122<br><br>Limiters - Published Date: 20100101-20221231; Exclude MEDLINE records	
S123	S96 OR S97 OR S98 OR S99 OR S100 OR S101 OR S102 OR S103 OR S104 OR S105 OR S106 OR S107 OR S108 OR S109 OR S110 OR S111 OR S112 OR S113 OR S114 OR S115 OR S116 OR S117 OR S118 OR S119 OR S120 OR S121 OR S122	
S122	(MH "Anthropometry+")	
S121	(MH "Body Weight+")	
S120	(MH "Body Weights and Measures+")	
S119	(MH "Cultural Competence")	
S118	(MH "Transcultural Care")	
S117	(MH "Transcultural Nursing")	
S116	(MH "Diet")	
S115	(MH "Food Habits")	
S114	(MH "Nutritional Physiology+")	
S113	(MH "Food and Beverages+")	
S112	(MH "Exercise")	
S111	(MH "Health Education+")	

S110	(MH "Health Promotion+")	
S109	(MH "Life Style+")	
S108	(MH "Mental Health")	
S107	(MH "Nutritional Assessment")	
S106	(MH "Nutritional Status")	
S105	(MH "Poverty+")	
S104	(MH "Preventive Health Care+")	
S103	(MH "Smoking Cessation")	
S102	(MH "Smoking Cessation Programs")	
S101	(MH "Smoking+")	
S100	(MH "Teaching+")	
S99	(MH "Passive Smoking")	
S98	(MH "Tobacco Use Cessation Products+")	
S97	(MH "Tobacco Products+")	
S96	(TI (anthropometric OR anthropometrical OR anthropometrically OR anthropometrics OR Anthropometry OR Body Weight OR Condition specific OR Culturally Competent Care OR culturally diverse OR Diet OR Dietary OR Dietetical OR Dietetically OR Dietetics OR Diets OR emotional health OR Exercise OR Food regimen OR Food regimens OR Health Education OR Health prevention OR Health Promotion OR healthy habit OR healthy habits OR Healthy life OR Healthy life habits OR healthy living OR Life style OR Lifestyle OR low income OR Mental Health OR Nutrition Assessment OR Nutrition OR Nutritional Status OR nutritional OR physical activity OR poverty OR Preventive Health Service OR Preventive Health Services OR promotion of health OR Promotion OR Quit smoking OR Quitting smoking OR second hand smoke OR Smoke OR Smoked OR Smoker OR Smokers OR Smokes OR Smoking OR socioeconomic status OR Stop smoking OR Stopping smoking OR stress level OR stress levels OR Taught OR Teach OR Teaching OR Tobacco Use OR Weight)) OR (AB (anthropometric OR anthropometrical OR anthropometrically OR anthropometrics OR Anthropometry OR Body Weight OR Condition specific OR Culturally Competent Care OR culturally diverse OR Diet OR Dietary OR Dietetical OR Dietetically OR Dietetics OR Diets OR emotional health OR Exercise OR Food regimen OR Food regimens OR Health Education OR Health prevention OR Health Promotion OR healthy habit OR healthy habits OR Healthy life OR Healthy life habits OR healthy living OR Life style OR Lifestyle OR low income OR Mental Health OR Nutrition Assessment OR Nutrition OR Nutritional Status OR nutritional OR physical activity OR poverty OR Preventive Health Service OR Preventive Health Services OR promotion of health OR Promotion OR Quit smoking OR Quitting smoking OR second hand smoke OR Smoke OR Smoked OR	

	Smoker OR Smokers OR Smokes OR Smoking OR socioeconomic status OR Stop smoking OR Stopping smoking OR stress level OR stress levels OR Taught OR Teach OR Teaching OR Tobacco Use OR Weight))
S95	S59 OR S60 OR S61 OR S62 OR S63 OR S64 OR S65 OR S66 OR S67 OR S68 OR S69 OR S70 OR S71 OR S72 OR S73 OR S74 OR S75 OR S76 OR S77 OR S78 OR S79 OR S80 OR S81 OR S82 OR S83 OR S84 OR S85 OR S86 OR S87 OR S88 OR S89 OR S90 OR S91 OR S92 OR S93<br>Limiters - Published Date: 20100101-20221231; Exclude MEDLINE records
S94	S59 OR S60 OR S61 OR S62 OR S63 OR S64 OR S65 OR S66 OR S67 OR S68 OR S69 OR S70 OR S71 OR S72 OR S73 OR S74 OR S75 OR S76 OR S77 OR S78 OR S79 OR S80 OR S81 OR S82 OR S83 OR S84 OR S85 OR S86 OR S87 OR S88 OR S89 OR S90 OR S91 OR S92 OR S93
S93	(MH "Appointments and Schedules+")
S92	(MH "Biological Markers")
S91	(MH "Hypertension")
S90	(MH "Cholesterol+")
S89	(MH "Hypercholesterolemia+")
S88	(MH "Chronic Disease+")
S87	(MH "Consumer Satisfaction+")
S86	(MH "Costs and Cost Analysis+")
S85	(MH "Health Care Delivery+")
S84	(MH "Diagnostic Tests, Routine")
S83	(MH "Hemoglobin A, Glycosylated")
S82	(MH "Guideline Adherence")
S81	(MH "Health Resource Utilization")
S80	(MH "Health Resource Allocation")
S79	(MH "Health Services+")
S78	(MH "Health Status Indicators")
S77	(MH "Health Status+")
S76	(MH "Job Satisfaction+")

S75	(MH "Mortality+")
S74	(MH "Outcomes (Health Care)+")
S73	(MH "Process Assessment (Health Care)+")
S72	(MH "Pain")
S71	(MH "Patient Satisfaction+")
S70	(MH "Personal Satisfaction+")
S69	(MH "Prescriptions, Non-Drug")
S68	(MH "Prescriptions, Drug+")
S67	(MH "Professional Role+")
S66	(MH "Quality of Health Care+")
S65	(MH "Referral and Consultation+")
S64	(MH "Task Performance and Analysis+")
S63	(MH "Medication Compliance")
S62	(MH "Patient Compliance+")
S61	(MH "Treatment Refusal")
S60	(MH "Patient Dropouts")
S59	(TI (Appointment OR Appointments OR Attendance OR Biomarker OR Biomarkers OR Hypertension OR Care Process OR Care Processes OR Cholesterol OR Hypercholesterolemia OR Chronic condition OR Chronic conditions OR Chronic disease OR Chronic diseases OR Chronic illness OR Chronic illnesses OR clinical outcome OR clinical outcomes OR clinical parameter OR clinical parameters OR Compliance OR Compliant OR Consultation OR Consultations OR Consumer Behavior OR Consumer Behaviors OR Consumer Behaviour OR Consumer Behaviours OR Cost analyses OR Cost analysis OR Cost OR Costs OR Death OR Deaths OR Delivery of health care OR Delivery of healthcare OR Diagnostic Test OR Diagnostic Testing OR Diagnostic Tests OR economic analyses OR economic analysis OR economic evaluation OR economic evaluations OR Emergency Service OR Emergency Services OR Follow up appointment OR Follow up appointments OR follow up visit OR follow up visits OR Glycated Haemoglobin A OR Glycated Hemoglobin A OR Guideline Adherence OR HbA1C OR hdl OR health care OR Health Planning OR Health related quality of life OR Health Resource OR Health Resources OR Health Service OR Health Services OR Health status OR Healthcare delivery OR Healthcare process assessment OR Healthcare Quality OR healthcare OR high density lipoprotein OR hospital admission OR hospital admissions OR hospital referral OR hospital referrals OR HRQOL OR Hypertension OR indicator OR indicators OR Job Satisfaction OR ldl OR Lipid profile OR Lipid profiles OR low density lipoprotein OR Manpower OR Mortalities OR

	Mortality OR Outcome Assessment OR Outcome Assessments OR Pain OR Pains OR Patient Satisfaction OR Personal Satisfaction OR prefer OR preference OR preferences OR preferred OR Prescribe OR Prescribed OR prescription OR prescriptions OR Process of care OR Processes of care OR Professional Role OR Professional Roles OR QOL OR quality indicator OR quality indicators OR Quality of Health Care OR Quality of Healthcare OR Quality of life OR Referral OR Referrals OR return appointment OR return appointments OR return visit OR return visits OR Routine diagnostic OR Routine test OR Routine testing OR Routine tests OR satisfaction OR satisfactory OR satisfied OR Schedule OR Schedules OR Task Analyses OR Task Analysis OR Task Performance OR Treatment Adherence OR treatment adherent OR Treatment Compliance OR Treatment compliant OR Wellness)) OR (AB (Appointment OR Appointments OR Attendance OR Biomarker OR Biomarkers OR Hypertension OR Care Process OR Care Processes OR Cholesterol OR Hypercholesterolemia OR Chronic condition OR Chronic conditions OR Chronic disease OR Chronic diseases OR Chronic illness OR Chronic illnesses OR clinical outcome OR clinical outcomes OR clinical parameter OR clinical parameters OR Compliance OR Compliant OR Consultation OR Consultations OR Consumer Behavior OR Consumer Behaviors OR Consumer Behaviour OR Consumer Behaviours OR Cost analyses OR Cost analysis OR Cost OR Costs OR Death OR Deaths OR Delivery of health care OR Delivery of healthcare OR Diagnostic Test OR Diagnostic Testing OR Diagnostic Tests OR economic analyses OR economic analysis OR economic evaluation OR economic evaluations OR Emergency Service OR Emergency Services OR Follow up appointment OR Follow up appointments OR follow up visit OR follow up visits OR Glycated Haemoglobin A OR Glycated Hemoglobin A OR Guideline Adherence OR HbA1C OR hdl OR health care OR Health Planning OR Health related quality of life OR Health Resource OR Health Resources OR Health Service OR Health Services OR Health status OR Healthcare delivery OR Healthcare process assessment OR Healthcare Quality OR healthcare OR high density lipoprotein OR hospital admission OR hospital admissions OR hospital referral OR hospital referrals OR HRQOL OR Hypertension OR indicator OR indicators OR Job Satisfaction OR ldl OR Lipid profile OR Lipid profiles OR low density lipoprotein OR Manpower OR Mortalities OR Mortality OR Outcome Assessment OR Outcome Assessments OR Pain OR Pains OR Patient Satisfaction OR Personal Satisfaction OR prefer OR preference OR preferences OR preferred OR Prescribe OR Prescribed OR prescription OR prescriptions OR Process of care OR Processes of care OR Professional Role OR Professional Roles OR QOL OR quality indicator OR quality indicators OR Quality of Health Care OR Quality of Healthcare OR Quality of life OR Referral OR Referrals OR return appointment OR return appointments OR return visit OR return visits OR Routine diagnostic OR Routine test OR Routine testing OR Routine tests OR satisfaction OR satisfactory OR satisfied OR Schedule OR Schedules OR Task Analyses OR Task Analysis OR Task Performance OR Treatment Adherence OR treatment adherent OR Treatment Compliance OR Treatment compliant OR Wellness))
S58	S57<br><br>Limiters - Published Date: 20100101-20221231; Exclude MEDLINE records
S57	S50 OR S51 OR S52 OR S53 OR S54 OR S55 OR S56
S56	(MH "Advanced Nursing Practice+")
S55	(MH "Advanced Practice Nurses+")
S54	(MH "Family Nurse Practitioners")

S53	(MH "Nurse Practitioners+")
S52	(MH "Emergency Nurse Practitioners")
S51	(MH "Adult Nurse Practitioners")
S50	(TI (advanced nurse practitioners OR "ANP" OR advanced nursing practice OR advanced nursing practices OR advanced practice nurse OR advanced practice nurses OR advanced practice nursing OR APN OR advanced practice registered nurse OR advanced practice registered nurses OR advanced practice registered nursing OR APRN OR Emergency nurse practitioner OR Emergency nurse practitioners OR ENP OR Family nurse practitioner OR Family nurse practitioners OR FNP OR Nurse practitioner OR Nurse practitioners OR "NP" OR Primary care nurse practitioner OR Primary care nurse practitioners OR Primary health care nurse practitioner OR PCNP OR Primary health care nurse practitioners OR Primary healthcare nurse practitioner OR Primary health care nurse practitioner OR Primary healthcare nurse practitioners OR Primary health care nurse practitioners OR PHCNP)) OR (AB (advanced nurse practitioners OR "ANP" OR advanced nursing practice OR advanced nursing practices OR advanced practice nurse OR advanced practice nurses OR advanced practice nursing OR APN OR advanced practice registered nurse OR advanced practice registered nurses OR advanced practice registered nursing OR APRN OR Emergency nurse practitioner OR Emergency nurse practitioners OR ENP OR Family nurse practitioner OR Family nurse practitioners OR FNP OR Nurse practitioner OR Nurse practitioners OR "NP" OR Primary care nurse practitioner OR Primary care nurse practitioners OR Primary health care nurse practitioner OR PCNP OR Primary health care nurse practitioners OR Primary healthcare nurse practitioner OR Primary health care nurse practitioner OR Primary healthcare nurse practitioners OR Primary health care nurse practitioners OR PHCNP))
S49	S1 OR S2 OR S3 OR S4 OR S5 OR S6 OR S7 OR S8 OR S9 OR S10 OR S11 OR S12 OR S13 OR S14 OR S15 OR S16 OR S17 OR S18 OR S19 OR S20 OR S21 OR S22 OR S23 OR S24 OR S25 OR S26 OR S27 OR S28 OR S29 OR S30 OR S31 OR S32 OR S33 OR S34 OR S35 OR S36 OR S37 OR S38 OR S39 OR S40 OR S41 OR S42 OR S43 OR S44 OR S45 OR S46<br>Limiters - Published Date: 20100101-20221231; Exclude MEDLINE records
S48	S1 OR S2 OR S3 OR S4 OR S5 OR S6 OR S7 OR S8 OR S9 OR S10 OR S11 OR S12 OR S13 OR S14 OR S15 OR S16 OR S17 OR S18 OR S19 OR S20 OR S21 OR S22 OR S23 OR S24 OR S25 OR S26 OR S27 OR S28 OR S29 OR S30 OR S31 OR S32 OR S33 OR S34 OR S35 OR S36 OR S37 OR S38 OR S39 OR S40 OR S41 OR S42 OR S43 OR S44 OR S45 OR S46<br>Limiters - Published Date: 20100101-20221231
S47	S1 OR S2 OR S3 OR S4 OR S5 OR S6 OR S7 OR S8 OR S9 OR S10 OR S11 OR S12 OR S13 OR S14 OR S15 OR S16 OR S17 OR S18 OR S19 OR S20 OR S21 OR S22 OR S23 OR S24 OR S25 OR S26 OR S27 OR S28 OR S29 OR S30 OR S31 OR S32 OR S33 OR S34 OR S35 OR S36 OR S37 OR S38 OR S39 OR S40 OR S41 OR S42 OR S43 OR S44 OR S45 OR S46
S46	(TI (ambulatory care OR Care continuity OR CHSLD OR CHSLDs OR CLSC OR CLSC's OR CLSCs OR Community care OR Community clinic OR Community clinics OR Community doctor OR Community doctors OR Community facilities OR Community facility OR Community health center OR Community health centers OR Community health centre OR Community health centres OR Community health nurse

	OR Community health nurses OR Community health nursing OR Community health plan OR Community health planning OR Community health plans OR Community health service OR Community health services OR Community medicine OR Community mental health service OR Community mental health services OR Community Nurse OR Community Nurses OR Community Nursing OR Community physician OR Community physicians OR Community practice OR Community practices OR Community program OR Community programs OR Community service OR Community services OR Continuity of care OR Counseling OR Counselling OR Delivery of health care OR Delivery of healthcare OR Distance Counseling OR Distance Counselling OR Elder care OR Elderly nursing home OR Elderly nursing homes OR Family clinic OR Family clinics OR Family doctor OR Family doctors OR Family medicine group OR Family medicine groups OR Family medicine OR Family physician OR Family physicians OR Family practice OR Family practices OR general practice OR general practices OR general practitioner OR general practitioners OR Group practice OR Group practices OR Health care delivery OR Health care professional OR Health care professionals OR Health care provider OR Health care providers OR Health care team OR Health care teams OR Health personnel OR Health professional OR Health professionals OR Health Services administration OR Healthcare delivery OR Healthcare professional OR Healthcare professionals OR Healthcare provider OR Healthcare providers OR Healthcare team OR Healthcare teams OR Home care OR Home Health OR Home Nurse OR Home Nurses OR Home Nursing OR interactive consultation OR interactive consultations OR interactive service OR interactive services OR interdisciplinary team OR interdisciplinary teams OR Intermediate care facility OR intermediate care OR interprofessional team OR interprofessional teams OR Local community service center OR Local community service centers OR Local community service centre OR Local community service centres OR Long-term care center OR Long-term care centers OR Long-term care centre OR Long-term care centres OR Long-term care home OR Long-term care homes OR multidisciplinary team OR multidisciplinary teams OR Multidiscipline team OR multidiscipline teams OR Nurse management OR Nurse OR Nurse-delivered OR Nurse-led OR Nurse-managed OR Nurse-run OR Nurses management OR Nurses OR Nurses-led OR Nursing care OR Nursing delivered OR Nursing home OR Nursing homes OR Nursing management OR Nursing OR Nursing-delivered OR Nursing-led OR Nursing-managed OR Nursing-run OR Office nurse OR Office nurses OR Office nursing OR Outpatient OR Outpatients OR Patient care team OR Patient care teams OR Primary care OR Primary health care OR Primary healthcare OR Professional autonomy OR Professional practice OR Professional practices OR Remote OR Residential care center OR Residential care centers OR Residential care centre OR Residential care centres OR Residential care OR Respite Care OR Retirement home OR Retirement homes OR rural health OR Rural nurse OR Rural nurses OR Rural nursing OR Staff Nurse OR Staff Nurses OR Suburban Health Service OR Suburban OR team OR teams OR Teamwork OR Telemedicine OR tele-medicine OR Telenurse OR Tele-nurse OR Telenurses OR Tele-nurses OR Telenursing OR Tele-nursing OR Telerehabilitation OR Tele-rehabilitation OR Walk-in clinic OR Walk-in clinics)) OR (AB (ambulatory care OR Care continuity OR CHSLD OR CHSLDs OR CLSC OR CLSC's OR CLSCs OR Community care OR Community clinic OR Community clinics OR Community doctor OR Community doctors OR Community facilities OR Community facility OR Community health center OR Community health centers OR Community health centre OR Community health centres OR Community health nurse OR Community health nurses OR Community health nursing OR Community health plan OR Community health planning OR Community health plans OR Community health service OR Community health services OR Community medicine OR Community mental health service OR Community mental health services OR Community Nurse OR Community Nurses OR Community Nursing OR Community physician OR Community physicians OR Community practice OR Community practices OR Community program OR Community programs OR Community service OR Community services OR Continuity of care OR Counseling OR Counselling OR Delivery of health care OR Delivery of healthcare OR Distance

	Counseling OR Distance Counselling OR Elder care OR Elderly nursing home OR Elderly nursing homes OR Family clinic OR Family clinics OR Family doctor OR Family doctors OR Family medicine group OR Family medicine groups OR Family medicine OR Family physician OR Family physicians OR Family practice OR Family practices OR general practice OR general practices OR general practitioner OR general practitioners OR Group practice OR Group practices OR Health care delivery OR Health care professional OR Health care professionals OR Health care provider OR Health care providers OR Health care team OR Health care teams OR Health personnel OR Health professional OR Health professionals OR Health Services administration OR Healthcare delivery OR Healthcare professional OR Healthcare professionals OR Healthcare provider OR Healthcare providers OR Healthcare team OR Healthcare teams OR Home care OR Home Health OR Home Nurse OR Home Nurses OR Home Nursing OR interactive consultation OR interactive consultations OR interactive service OR interactive services OR interdisciplinary team OR interdisciplinary teams OR Intermediate care facility OR intermediate care OR interprofessional team OR interprofessional teams OR Local community service center OR Local community service centers OR Local community service centre OR Local community service centres OR Long-term care center OR Long-term care centers OR Long-term care centre OR Long-term care centres OR Long-term care home OR Long-term care homes OR multidisciplinary team OR multidisciplinary teams OR Multidiscipline team OR multidiscipline teams OR Nurse management OR Nurse OR Nurse-delivered OR Nurse-led OR Nurse-managed OR Nurse-run OR Nurses management OR Nurses OR Nurses-led OR Nursing care OR Nursing delivered OR Nursing home OR Nursing homes OR Nursing management OR Nursing OR Nursing-delivered OR Nursing-led OR Nursing-managed OR Nursing-run OR Office nurse OR Office nurses OR Office nursing OR Outpatient OR Outpatients OR Patient care team OR Patient care teams OR Primary care OR Primary health care OR Primary healthcare OR Professional autonomy OR Professional practice OR Professional practices OR Remote OR Residential care center OR Residential care centers OR Residential care centre OR Residential care centres OR Residential care OR Respite Care OR Retirement home OR Retirement homes OR rural health OR Rural nurse OR Rural nurses OR Rural nursing OR Staff Nurse OR Staff Nurses OR Suburban Health Service OR Suburban OR team OR teams OR Teamwork OR Telemedicine OR tele-medicine OR Telenurse OR Tele-nurse OR Telenurses OR Tele-nurses OR Telenursing OR Tele-nursing OR Telerehabilitation OR Tele-rehabilitation OR Walk-in clinic OR Walk-in clinics))
S45	(MH "Telerehabilitation")
S44	(MH "Telenursing")
S43	(MH "Telemedicine+")
S42	(MH "Rural Health Nursing")
S41	(MH "Rural Health")
S40	(MH "Rural Health Centers")
S39	(MH "Rural Health Services")
S38	(MH "Respite Care")
S37	(MH "Remote Consultation")

S36	(MH "Professional Practice+")
S35	(MH "Professional Autonomy")
S34	(MH "Primary Health Care")
S33	(MH "Physicians, Family")
S32	(MH "Multidisciplinary Care Team+")
S31	(MH "Outpatients")
S30	(MH "Office Nursing")
S29	(MH "Team Nursing")
S28	(MH "Nursing Practice+")
S27	(MH "Nursing Home Patients")
S26	(MH "Nursing Home Personnel")
S25	(MH "Nursing Homes+")
S24	(MH "Nursing Care+")
S23	(MH "Long Term Care")
S22	(MH "Halfway Houses")
S21	(MH "Health Services for the Aged")
S20	(MH "Housing for the Elderly")
S19	(MH "Home Visits")
S18	(MH "Home Nursing, Professional")
S17	(MH "Home Nursing")
S16	(MH "Home Health Care+")
S15	(MH "Health Services Administration+")
S14	(MH "Health Personnel+")
S13	(MH "Group Practice")
S12	(MH "Practitioner's Office")

S11	(MH "Family Practice")
S10	(MH "Health Care Delivery+")
S9	(MH "Community Mental Health Nursing")
S8	(MH "Community Mental Health Services+")
S7	(MH "Community Medicine")
S6	(MH "Community Health Services+")
S5	(MH "Community Health Nursing+")
S4	(MH "Community Health Centers+")
S3	(MH "Ambulatory Care Nursing")
S2	(MH "Ambulatory Care")
S1	(MH "Ambulatory Care Facilities+")

7. Web of Science Core Collection

#20 #19 AND #9 AND #2 AND #1
Indexes=SCI-EXPANDED, SSCI, A&HCI, CPCI-S, SPCI-SSH Timespan=2010-2022

#19 #18 OR #17 OR #16 OR #15 OR #14 OR #13 OR #12 OR #11 OR #10
Indexes=SCI-EXPANDED, SSCI, A&HCI, CPCI-S, SPCI-SSH Timespan=2010-2022

#18 TI=evidence AND (TI=(reviews OR meta-analyses))
Indexes=SCI-EXPANDED, SSCI, A&HCI, CPCI-S, SPCI-SSH Timespan=2010-2022

#17 AB=cochrane reviews
Indexes=SCI-EXPANDED, SSCI, A&HCI, CPCI-S, SPCI-SSH Timespan=2010-2022

#16 AB=(reviews NEAR/2 (meta OR published OR quality OR included OR summar*))
Indexes=SCI-EXPANDED, SSCI, A&HCI, CPCI-S, SPCI-SSH Timespan=2010-2022

#15 (TI=(overview* OR reviews)) AND (TI=(systematic OR cochrane))
Indexes=SCI-EXPANDED, SSCI, A&HCI, CPCI-S, SPCI-SSH Timespan=2010-2022

#14 (TI=meta-review OR AB=meta-review) OR (TI=metareview OR AB=metareview)
Indexes=SCI-EXPANDED, SSCI, A&HCI, CPCI-S, SPCI-SSH Timespan=2010-2022

#13 (TI=(overview* OR review OR synthesis OR summary OR cochrane OR analysis) AND TI=(reviews OR meta-analyses OR articles))
Indexes=SCI-EXPANDED, SSCI, A&HCI, CPCI-S, SPCI-SSH Timespan=2010-2022

#12 TS=(meta analy* OR metanaly* OR metaanaly* OR met analy*)
Indexes=SCI-EXPANDED, SSCI, A&HCI, CPCI-S, SPCI-SSH Timespan=2010-2022

#11 TS=(meta-analysis OR systematic review)
Indexes=SCI-EXPANDED, SSCI, A&HCI, CPCI-S, SPCI-SSH Timespan=2010-2022

#10 TS=((Systematic* NEAR/3 (Review* OR Overview*)) OR (Methodologic* NEAR/3 (Review* OR Overview*)))
Indexes=SCI-EXPANDED, SSCI, A&HCI, CPCI-S, SPCI-SSH Timespan=2010-2022

#9 #8 OR #7 OR #6 OR #5 OR #4 OR #3
Indexes=SCI-EXPANDED, SSCI, A&HCI, CPCI-S, SPCI-SSH Timespan=2010-2022

#8 TS=(Aboriginal OR Communication Barriers OR Delivery of Health Care OR Delivery of HealthCare OR Disability Evaluation OR Discrimination OR equity OR Frailty OR Gender Minorities OR Gender Minority OR Global Health OR Health Care Delivery OR Health care Disparities OR Health Care Disparity OR Health Equity OR Health Literacy OR Health Status Disparities OR Health Status Disparity OR HealthCare Delivery OR Healthcare Disparities OR Healthcare Disparity OR Income OR Incomes OR inequal OR inequalities OR inequality OR Insurance cover OR Insurance coverage OR Insurance coverages OR Insurance covered OR "International Classification of Functioning, Disability and Health" OR Marital status OR Minority Health OR Oceanic Ancestry Group OR Oceanic Ancestry Groups OR Patient selection OR population dynamics OR Population group OR Population groups OR Poverty area OR Poverty areas OR Poverty OR Refusal to Treat OR Religion OR Religions OR safety net OR Sexual Minorities OR Sexual

Minority OR Sexual Orientation OR Sexual Orientations OR Social Discrimination OR Social Discriminations OR Social support OR treatment failure OR treatment failures OR Treatment preference OR Treatment preferences OR Vulnerabilities OR Vulnerability OR Vulnerable)
Indexes=SCI-EXPANDED, SSCI, A&HCI, CPCI-S, SPCI-SSH Timespan=2010-2022

#7 TS=(Access to care OR Access to health care OR Access to Health Services OR Access to healthcare OR Accessibility of Health Services OR Allied Health Personnel OR Ambulatory Care Information System OR Ambulatory Care Information Systems OR ambulatory care OR Appointment OR Appointments OR care access OR Clinical Competence OR Clinical Competencies OR Clinical Competency OR Consultation OR Consultations OR Cost OR Costs OR Duration of Therapy OR Patient Education as Topic OR Patient teaching OR Evaluated Health Care OR Evaluated HealthCare OR Evaluating Health Care OR Evaluating HealthCare OR Evaluation of Health Care OR Evaluations of Health Care OR Harm prevention OR Harm Reduction OR Health Attitude OR Health Attitudes OR Health Behavior OR Health Behaviors OR Health Behaviour OR Health Behaviours OR HealthCare OR Health Care OR Health Knowledge OR Health Personnel OR Health Plan Implementation OR Health Service Accessibility OR Health Services Accessibility OR healthy environment OR healthy environments OR Hospital Shared Services OR House Call OR House Calls OR indirect cost OR indirect costs OR Knowledge of Health OR Length of Stay OR Length of therapy OR Lengths of Stay OR LOS OR Office Visit OR Office Visits OR Patient Acceptance OR patient admission OR patient admissions OR Patient Advocacy OR Prescription OR Prescriptions OR Prevention of harm OR Quality Health Care OR Quality HealthCare OR Reduction of harm OR Referral OR Referrals OR return visit OR return visits OR Schedule OR Scheduled OR Schedules OR Therapy duration OR Therapy durations OR Therapy length OR Universal Health Care OR Universal HealthCare OR unplanned)
Indexes=SCI-EXPANDED, SSCI, A&HCI, CPCI-S, SPCI-SSH Timespan=2010-2022

#6 TS=(ACS OR ACSs OR Acute Coronary Syndrome OR Acute Coronary Syndromes OR Adjustment Disorder OR Adjustment Disorders OR Alcohol consumption OR Alcohol Drinking OR Alcohol Related Disorder OR Alcohol Related Disorders OR Alcoholic OR Alcoholism OR Anxiety OR anxious OR Blood Glucose OR Blood sugar OR Blood sugars OR Hyperglycemia OR Hypoglycemia OR Cancer OR Cardiovascular disease OR Cardiovascular diseases OR cerebrovascular accident OR cerebrovascular accidents OR CHF OR Chronic Obstructive Pulmonary Disease OR Chronic Obstructive Pulmonary Disorder OR Chronic Obstructive Pulmonary Disorders OR complication OR complications OR Congestive heart failure OR copd OR Coronary artery disease OR Coronary artery diseases OR Coronary Disease OR Coronary Diseases OR CVA OR CVAs OR CVD OR CVDs OR Dementia OR Dementias OR depressed OR depression OR Depressive Disorder OR Depressive Disorders OR depressive OR Diabetes Mellitus OR Diabetes OR Diabetic Foot OR foot complication OR foot complications OR gestational diabetes OR glycaemia OR glycaemic OR glycemia OR glycemic OR Health Care Evaluation Mechanisms OR Health Care Quality Indicator OR Health Care Quality Indicators OR Health Information Interoperability OR HealthCare Evaluation Mechanisms OR Healthcare Quality Indicator OR Healthcare Quality Indicators OR Heart Failure OR Interoperability OR Interoperable OR managed pain OR management of pain OR managing pain OR Mental Disorder OR Mental Disorders OR mental health OR Mental illness OR Mental illnesses OR mentally healthy OR Mentally ill OR Myocardial Infarction OR Neoplasm OR Neoplasms OR Oncologist OR Oncologists OR Oncology OR pain management OR pain OR pains OR Pap test OR Pap tests OR Papanicolaou Test OR Papanicolaou Tests OR Patient Reported Experience Measure OR Patient Reported Experience Measures OR Patient Reported Outcome Measure OR Patient Reported Outcome Measures OR Physical Exam OR Physical Examination OR Physical Examinations OR Physical Exams OR PREM OR PREMs OR PROM OR promotion OR PROMs OR psycho social OR psychosocial OR Quality Indicator OR Quality indicators of health care OR Quality Indicators OR Rehabilitation OR self care OR Self Efficacy OR Stroke OR Strokes)
Indexes=SCI-EXPANDED, SSCI, A&HCI, CPCI-S, SPCI-SSH Timespan=2010-2022

#5 TS=(adolescence OR adolescent OR adolescents OR Child development OR Childhood OR healthy development OR Human development OR Immunisation OR Immunise OR Immunised OR Immunises OR Immunization OR Immunize OR Immunized OR Immunizes OR laboratory test OR Maternal Health Service OR Maternal Health Services OR Paediatric OR Paediatrics OR Pediatric OR Pediatrics OR pregnancy OR Prevent OR Prevented OR Prevention OR Prevents OR Primary Prevention OR Secondary Prevention OR sexually transmitted OR Vaccinate OR Vaccinated OR Vaccinates OR Vaccination OR Vaccine OR Vaccines OR youth OR youths)
Indexes=SCI-EXPANDED, SSCI, A&HCI, CPCI-S, SPCI-SSH Timespan=2010-2022

#4 TS=(anthropometric OR anthropometrical OR anthropometrically OR anthropometrics OR Anthropometry OR Body Weight OR Condition specific OR Culturally Competent Care OR culturally diverse OR Diet OR Dietary OR Dietetical OR Dietetically OR Dietetics OR Diets OR emotional health OR Exercise OR Food regimen OR Food regimens OR Health Education OR Health prevention OR Health Promotion OR healthy habit OR healthy habits OR Healthy life OR Healthy life habits OR healthy living OR Life style OR Lifestyle OR low income OR Mental Health OR Nutrition Assessment OR Nutrition OR Nutritional Status OR nutritional OR physical activity OR poverty OR Preventive Health Service OR Preventive Health Services OR promotion of health OR Promotion OR Quit smoking OR Quitting smoking OR second hand smoke OR Smoke OR Smoked OR Smoker OR Smokers OR Smokes OR Smoking OR socioeconomic status OR Stop smoking OR Stopping smoking OR stress level OR stress levels OR Taught OR Teach OR Teaching OR Tobacco Use OR Weight)
Indexes=SCI-EXPANDED, SSCI, A&HCI, CPCI-S, SPCI-SSH Timespan=2010-2022

#3 TS=(Appointment OR Appointments OR Attendance OR Biomarker OR Biomarkers OR Hypertension OR Care Process OR Care Processes OR Cholesterol OR Hypercholesterolemia OR Chronic condition OR Chronic conditions OR Chronic disease OR Chronic diseases OR Chronic illness OR Chronic illnesses OR clinical outcome OR clinical outcomes OR clinical parameter OR clinical parameters OR Compliance OR Compliant OR Consultation OR Consultations OR Consumer Behavior OR Consumer Behaviors OR Consumer Behaviour OR Consumer Behaviours OR Cost analyses OR Cost analysis OR Cost OR Costs OR Death OR Deaths OR Delivery of health care OR Delivery of healthcare OR Diagnostic Test OR Diagnostic Testing OR Diagnostic Tests OR economic analyses OR economic analysis OR economic evaluation OR economic evaluations OR Emergency Service OR Emergency Services OR Follow up appointment OR Follow up appointments OR follow up visit OR follow up visits OR Glycated Haemoglobin A OR Glycated Hemoglobin A OR Guideline Adherence OR HbA1C OR hdl OR health care OR Health Planning OR Health related quality of life OR Health Resource OR Health Resources OR Health Service OR Health Services OR Health status OR Healthcare delivery OR Healthcare process assessment OR Healthcare Quality OR healthcare OR high density lipoprotein OR hospital admission OR hospital admissions OR hospital referral OR hospital referrals OR HRQOL OR Hypertension OR indicator OR indicators OR Job Satisfaction OR ldl OR Lipid profile OR Lipid profiles OR low density lipoprotein OR Manpower OR Mortalities OR Mortality OR Outcome Assessment OR Outcome Assessments OR Pain OR Pains OR Patient Satisfaction OR Personal Satisfaction OR prefer OR preference OR preferences OR preferred OR Prescribe OR Prescribed OR prescription OR prescriptions OR Process of care OR Processes of care OR Professional Role OR Professional Roles OR QOL OR quality indicator OR quality indicators OR Quality of Health Care OR Quality of Healthcare OR Quality of life OR Referral OR Referrals OR return appointment OR return appointments OR return visit OR return visits OR Routine diagnostic OR Routine test OR Routine testing OR Routine tests OR satisfaction OR satisfactory OR satisfied OR Schedule OR Schedules OR Task

Analyses OR Task Analysis OR Task Performance OR Treatment Adherence OR treatment adherent OR Treatment Compliance OR Treatment compliant OR Wellness)
Indexes=SCI-EXPANDED, SSCI, A&HCI, CPCI-S, SPCI-SSH Timespan=2010-2022

#2 TS=(advanced nurse practitioner OR advanced nurse practitioners OR (ANP AND nurse) OR (ANP AND nurses) OR (ANP AND nursing) OR advanced nursing practice OR advanced nursing practices OR advanced practice nurse OR advanced practice nurses OR advanced practice nursing OR (APN AND nurse) OR (APN AND nurses) OR (APN AND nursing) OR advanced practice registered nurse OR advanced practice registered nurses OR advanced practice registered nursing OR (APRN AND nurse) OR (APRN AND nurses) OR (APRN AND nursing) OR Emergency nurse practitioner OR Emergency nurse practitioners OR (ENP AND Nurse) OR (ENP AND Nurses) OR (ENP AND Nursing) OR Family nurse practitioner OR Family nurse practitioners OR (FNP AND Nurse) OR (FNP AND Nurses) OR (FNP AND Nursing) OR Nurse practitioner OR Nurse practitioners OR (NP AND nurse) OR (NP AND nurses) OR (NP AND nursing) OR Primary care nurse practitioner OR Primary care nurse practitioners OR Primary health care nurse practitioner OR (PCNP AND Nurse) OR (PCNP AND Nurses) OR (PCNP AND Nursing) OR Primary health care nurse practitioners OR Primary healthcare nurse practitioner OR Primary health care nurse practitioner OR Primary healthcare nurse practitioners OR Primary health care nurse practitioners OR (PHCNP AND Nurse) OR (PHCNP AND Nurses) OR (PHCNP AND Nursing))
Indexes=SCI-EXPANDED, SSCI, A&HCI, CPCI-S, SPCI-SSH Timespan=2010-2022

#1 TS=(ambulatory care OR Care continuity OR CHSLD OR CHSLDs OR CLSC OR CLSC's OR CLSCs OR Community care OR Community clinic OR Community clinics OR Community doctor OR Community doctors OR Community facilities OR Community facility OR Community health center OR Community health centers OR Community health centre OR Community health centres OR Community health nurse OR Community health nurses OR Community health nursing OR Community health plan OR Community health planning OR Community health plans OR Community health service OR Community health services OR Community medicine OR Community mental health service OR Community mental health services OR Community Nurse OR Community Nurses OR Community Nursing OR Community physician OR Community physicians OR Community practice OR Community practices OR Community program OR Community programs OR Community service OR Community services OR Continuity of care OR Counseling OR Counselling OR Delivery of health care OR Delivery of healthcare OR Distance Counseling OR Distance Counselling OR Elder care OR Elderly nursing home OR Elderly nursing homes OR Family clinic OR Family clinics OR Family doctor OR Family doctors OR Family medicine group OR Family medicine groups OR Family medicine OR Family physician OR Family physicians OR Family practice OR Family practices OR general practice OR general practices OR general practitioner OR general practitioners OR Group practice OR Group practices OR Health care delivery OR Health care professional OR Health care professionals OR Health care provider OR Health care providers OR Health care team OR Health care teams OR Health personnel OR Health professional OR Health professionals OR Health Services administration OR Healthcare delivery OR Healthcare professional OR Healthcare professionals OR Healthcare provider OR Healthcare providers OR Healthcare team OR Healthcare teams OR Home care OR Home Health OR Home Nurse OR Home Nurses OR Home Nursing OR interactive consultation OR interactive consultations OR interactive service OR interactive services OR interdisciplinary team OR interdisciplinary teams OR Intermediate care facility OR intermediate care OR interprofessional team OR interprofessional teams OR Local community service center OR Local community service centers OR Local community service centre OR Local community service centres OR Long-term care center OR Long-term care centers OR Long-term care centre OR Long-term care centres OR Long-term care home OR Long-term care homes OR multidisciplinary team OR multidisciplinary teams OR Multidiscipline team OR

multidiscipline teams OR Nurse management OR Nurse OR Nurse-delivered OR Nurse-led OR Nurse-managed OR Nurse-run OR Nurses management OR Nurses OR Nurses-led OR Nursing care OR Nursing delivered OR Nursing home OR Nursing homes OR Nursing management OR Nursing OR Nursing-delivered OR Nursing-led OR Nursing-managed OR Nursing-run OR Office nurse OR Office nurses OR Office nursing OR Outpatient OR Outpatients OR Patient care team OR Patient care teams OR Primary care OR Primary health care OR Primary healthcare OR Professional autonomy OR Professional practice OR Professional practices OR Remote OR Residential care center OR Residential care centers OR Residential care centre OR Residential care centres OR Residential care OR Respite Care OR Retirement home OR Retirement homes OR rural health OR Rural nurse OR Rural nurses OR Rural nursing OR Staff Nurse OR Staff Nurses OR Suburban Health Service OR Suburban OR team OR teams OR Teamwork OR Telemedicine OR tele-medicine OR Telenurse OR Tele-nurse OR Telenurses OR Tele-nurses OR Telenursing OR Tele-nursing OR Telerehabilitation OR Tele-rehabilitation OR Walk-in clinic OR Walk-in clinics)
Indexes=SCI-EXPANDED, SSCI, A&HCI, CPCI-S, SPCI-SSH Timespan=2010-2022

8. Ovid Healthstar

1. exp Ambulatory Care Facilities/
2. Ambulatory Care/
3. exp Community Health Centers/
4. exp Community Health Nursing/
5. exp Community Health Planning/
6. exp Community Health services/
7. exp Community medicine/
8. Community Mental Health Services/
9. exp Delivery of Health Care/
10. exp family practice/
11. exp General Practice/
12. exp General Practitioners/
13. exp group practice/
14. exp Health Personnel/
15. exp Health Services Administration/
16. exp Home Care Services/
17. exp Homes for the Aged/
18. exp Housing for the Elderly/
19. exp Intermediate Care Facilities/
20. exp Long-Term Care/
21. exp Nurses, Community Health/
22. exp Nursing Care/
23. exp Nursing Homes/
24. exp Nursing Stations/
25. exp Nursing/
26. exp Nursing, team/
27. exp Office Nursing/
28. exp outpatients/
29. exp Patient Care Team/
30. exp Physicians, Family/
31. exp Physicians, Primary Care/
32. exp Primary Care Nursing/
33. exp Primary Health Care/
34. exp Professional Autonomy/
35. exp Professional Practice/
36. exp Remote Consultation/
37. exp Respite Care/
38. exp Rural Health Services/
39. exp Rural Health/
40. exp Rural Nursing/
41. exp Suburban Health Services/
42. Telemedicine/
43. exp Telenursing/

44. exp Telerehabilitation/

45. (ambulatory care or Care Continuity or Chsld or Chslds or Clsc or Clsc or Community Care or Community Clinic or Community Clinics or Community Doctor or Community Doctors or Community Facilities or Community Facility or Community Health Center or Community Health Centers or Community Health Centre or Community Health Centres or Community Health Nurse or Community Health Nurses or Community Health Nursing or Community Health Plan or Community Health Planning or Community Health Plans or Community Health Service or Community Health Services or Community Medicine or Community Mental Health Service or Community Mental Health Services or Community Nurse or Community Nurses or Community Nursing or Community Physician or Community Physicians or Community Practice or Community Practices or Community Program or Community Programs or Community Service or Community Services or Continuity of Care or Counseling or Counselling or Delivery of Health Care or Delivery of Healthcare or Distance Counseling or Distance Counselling or Elder Care or Elderly Nursing Home or Elderly Nursing Homes or Family Clinic or Family Clinics or Family Doctor or Family Doctors or Family Medicine Group or Family Medicine Groups or Family Medicine or Family Physician or Family Physicians or Family Practice or Family Practices or General Practice or General Practices or General Practitioner or General Practitioners or Group Practice or Group Practices or Health Care Delivery or Health Care Professional or Health Care Professionals or Health Care Provider or Health Care Providers or Health Care Team or Health Care Teams or Health Personnel or Health Professional or Health Professionals or Health Services Administration or Healthcare Delivery or Healthcare Professional or Healthcare Professionals or Healthcare Provider or Healthcare Providers or Healthcare Team or Healthcare Teams or Home Care or Home Health or Home Nurse or Home Nurses or Home Nursing or Interactive Consultation or Interactive Consultations or Interactive Service or Interactive Services or Interdisciplinary Team or Interdisciplinary Teams or Intermediate Care Facility or Intermediate Care or Interprofessional Team or Interprofessional Teams or Local Community Service Center or Local Community Service Centers or Local Community Service Centre or Local Community Service Centres or Long-Term Care Center or Long-Term Care Centers or Long-Term Care Centre or Long-Term Care Centres or Long-Term Care Home or Long-Term Care Homes or Multidisciplinary Team or Multidisciplinary Teams or Multidiscipline Team or Multidiscipline Teams or Nurse Management or Nurse or Nurse-Delivered or Nurse-Led or Nurse-Managed or Nurse-Run or Nurses Management or Nurses or Nurses-Led or Nursing Care or Nursing Delivered or Nursing Home or Nursing Homes or Nursing Management or Nursing or Nursing-Delivered or Nursing-Led or Nursing-Managed or Nursing-Run or Office Nurse or Office Nurses or Office Nursing or Outpatient or Outpatients or Patient Care Team or Patient Care Teams or Primary Care or Primary Health Care or Primary Healthcare or Professional Autonomy or Professional Practice or Professional Practices or Remote or Residential Care Center or Residential Care Centers or Residential Care Centre or Residential Care Centres or Residential Care or Respite Care or Retirement Home or Retirement Homes or Rural Health or Rural Nurse or Rural Nurses or Rural Nursing or Staff Nurse or Staff Nurses or Suburban Health Service or Suburban or Team or Teams or Teamwork or Telemedicine or Tele-Medicine or Telenurse or Tele-Nurse or Telenurses or Tele-Nurses or Telenursing or Tele-Nursing or Telerehabilitation or Tele-Rehabilitation or Walk-In Clinic or Walk-In Clinics).ab,ti.

46. 1 or 2 or 3 or 4 or 5 or 6 or 7 or 8 or 9 or 10 or 11 or 12 or 13 or 14 or 15 or 16 or 17 or 18 or 19 or 20 or 21 or 22 or 23 or 24 or 25 or 26 or 27 or 28 or 29 or 30 or 31 or 32 or 33 or 34 or 35 or 36 or 37 or 38 or 39 or 40 or 41 or 42 or 43 or 44 or 45

47. limit 46 to yr="2010 -Current"

48. exp Advanced Practice Nursing/

49. exp Family Nurse Practitioners/
50. exp Nurse Practitioners/
51. (Advanced Nurse Practitioner or Advanced Nurse Practitioners or (ANP and Nurse) or (ANP and Nurses) or (ANP and Nursing) or Advanced Nursing Practice or Advanced Nursing Practices or Advanced Practice Nurse or Advanced Practice Nurses or Advanced Practice Nursing or (APN and Nurse) or (APN and Nurses) or (APN and Nursing) or Advanced Practice Registered Nurse or Advanced Practice Registered Nurses or Advanced Practice Registered Nursing or (APRN and Nurse) or (APRN and Nurses) or (APRN and Nursing) or Emergency Nurse Practitioner or Emergency Nurse Practitioners or (APRN and Nurse) or (APRN and Nurses) or (APRN and Nursing) or Family nurse practitioner or Family nurse practitioners or (FNP and Nurse) or (FNP and Nurses) or (FNP and Nursing) or Nurse Practitioner or Nurse Practitioners or (NP and Nurse) or (NP and Nurses) or (NP and Nursing) or Primary Care Nurse Practitioner or Primary Care Nurse Practitioners or Primary Health Care Nurse Practitioner or (PCNP and Nurse) or (PCNP and Nurses) or (PCNP and Nursing) or Primary Health Care Nurse Practitioners or Primary Healthcare Nurse Practitioner or Primary Health Care Nurse Practitioner or Primary Healthcare Nurse Practitioners or Primary Health care Nurse Practitioners or (PHCNP and Nurse) or (PHCNP and Nurses) or (PHCNP and Nursing)).ab,ti.
52. 48 or 49 or 50 or 51
53. limit 52 to yr="2010 -Current"
54. exp "Appointments and Schedules"/
55. Biomarkers/
56. Hypertension/
57. exp Cholesterol/
58. exp Hypercholesterolemia/
59. exp Chronic Disease/
60. exp Consumer Behavior/
61. exp "Costs and Cost Analysis"/
62. exp Delivery of Health Care/
63. exp Diagnostic Tests, Routine/
64. exp Glycated Hemoglobin A/
65. exp Guideline Adherence/
66. exp Health Resources/
67. exp Health Services/
68. exp Health Status Indicators/
69. exp Health Status/
70. exp Job Satisfaction/
71. exp Mortality/
72. exp "Outcome and Process Assessment, Health Care"/
73. exp Outcome Assessment, Health Care/
74. Pain/
75. exp Patient Satisfaction/
76. exp Personal Satisfaction/
77. exp Prescriptions/
78. exp Professional Role/
79. exp Quality of Health Care/
80. exp "Referral and Consultation"/

81. exp "Task Performance and Analysis"/
82. exp "Treatment Adherence and Compliance"/
83. (Appointment or Appointments or Attendance or Biomarker or Biomarkers or Hypertension or Care Process or Care Processes or Cholesterol or Hypercholesterolemia or Chronic Condition or Chronic Conditions or Chronic Disease or Chronic Diseases or Chronic Illness or Chronic Illnesses or Clinical Outcome or Clinical Outcomes or Clinical Parameter or Clinical Parameters or Compliance or Compliant or Consultation or Consultations or Consumer Behavior or Consumer Behaviors or Consumer Behaviour or Consumer Behaviours or Cost Analyses or Cost Analysis or Cost or Costs or Death or Deaths or Delivery Of Health Care or Delivery Of Healthcare or Diagnostic Test or Diagnostic Testing or Diagnostic Tests or Economic Analyses or Economic Analysis or Economic Evaluation or Economic Evaluations or Emergency Service or Emergency Services or Follow Up Appointment or Follow Up Appointments or Follow Up Visit or Follow Up Visits or Glycated Haemoglobin A or Glycated Hemoglobin A or Guideline Adherence or HbA1C or Hdl or Health Care or Health Planning or Health Related Quality Of Life or Health Resource or Health Resources or Health Service or Health Services or Health Status or Healthcare Delivery or Healthcare Process Assessment or Healthcare Quality or Healthcare or High Density Lipoprotein or Hospital Admission or Hospital Admissions or Hospital Referral or Hospital Referrals or Hrqol or Hypertension or Indicator or Indicators or Job Satisfaction or Ldl or Lipid Profile or Lipid Profiles or Low Density Lipoprotein or Manpower or Mortalities or Mortality or Outcome Assessment or Outcome Assessments or Pain or Pains or Patient Satisfaction or Personal Satisfaction or Prefer or Preference or Preferences or Preferred or Prescribe or Prescribed or Prescription or Prescriptions or Process Of Care or Processes Of Care or Professional Role or Professional Roles or Qol or Quality Indicator or Quality Indicators or Quality Of Health Care or Quality Of Healthcare or Quality Of Life or Referral or Referrals or Return Appointment or Return Appointments or Return Visit or Return Visits or Routine Diagnostic or Routine Test or Routine Testing or Routine Tests or Satisfaction or Satisfactory or Satisfied or Schedule or Schedules or Task Analyses or Task Analysis or Task Performance or Treatment Adherence or Treatment Adherent or Treatment Compliance or Treatment Compliant or Wellness).ab,ti.
84. 54 or 55 or 56 or 57 or 58 or 59 or 60 or 61 or 62 or 63 or 64 or 65 or 66 or 67 or 68 or 69 or 70 or 71 or 72 or 73 or 74 or 75 or 76 or 77 or 78 or 79 or 80 or 81 or 82 or 83
85. limit 84 to yr="2010 -Current"
86. exp Anthropometry/
87. exp Body Weight/
88. exp Culturally Competent Care/
89. Diet/
90. exp "Diet, Food, and Nutrition"/
91. Exercise/
92. exp Health Education/
93. exp Health Promotion/
94. exp Healthy Lifestyle/
95. exp Mental Health/
96. exp Nutrition Assessment/
97. exp Nutritional Status/
98. exp Poverty/
99. exp Preventive Health Services/
100. exp Smoking Cessation/
101. exp Smoking Prevention/

102. exp Smoking/
103. exp Teaching/
104. exp Tobacco Smoke Pollution/
105. (Anthropometric or Anthropometrical or Anthropometrically or Anthropometrics or Anthropometry or Body Weight or Condition Specific or Culturally Competent Care or Culturally Diverse or Diet or Dietary or Dietetical or Dietetically or Dietetics or Diets or Emotional Health or Exercise or Food Regimen or Food Regimens or Health Education or Health Prevention or Health Promotion or Healthy Habit or Healthy Habits or Healthy Life or Healthy Life Habits or Healthy Living or Life Style or Lifestyle or Low Income or Mental Health or Nutrition Assessment or Nutrition or Nutritional Status or Nutritional or Physical Activity or Poverty or Preventive Health Service or Preventive Health Services or Promotion of Health or Promotion or Quit Smoking or Quitting Smoking or Second Hand Smoke or Smoke or Smoked or Smoker or Smokers or Smokes or Smoking or Socioeconomic Status or Stop Smoking or Stopping Smoking or Stress Level or Stress Levels or Taught or Teach or Teaching or Tobacco or Weight).ab,ti.
106. 86 or 87 or 88 or 89 or 90 or 91 or 92 or 93 or 94 or 95 or 96 or 97 or 98 or 99 or 100 or 101 or 102 or 103 or 104 or 105
107. limit 106 to yr="2010 -Current"
108. exp Adolescent/
109. exp Child Development/
110. exp Human Development/
111. exp Immunization/
112. exp Maternal Health Services/
113. exp Pediatrics/
114. exp Primary Prevention/
115. exp Secondary Prevention/
116. Sexually Transmitted Diseases/
117. exp Vaccination/
118. (Adolescence or Adolescent or Adolescents or Child Development or Childhood or Healthy Development or Human Development or Immunisation or Immunise or Immunised or Immunises or Immunization or Immunize or Immunized or Immunizes or Laboratory Test or Maternal Health Service or Maternal Health Services or Paediatric or Paediatrics or Pediatric or Pediatrics or Pregnancy or Prevent or Prevented or Prevention or Prevents or Primary Prevention or Secondary Prevention or Sexually Transmitted or Vaccinate or Vaccinated or Vaccinates or Vaccination or Vaccine or Vaccines or Youth or Youths).ab,ti.
119. 108 or 109 or 110 or 111 or 112 or 113 or 114 or 115 or 116 or 117 or 118
120. limit 119 to yr="2010 -Current"
121. Acute Coronary Syndrome/
122. exp Adjustment Disorders/
123. exp Alcohol Drinking/
124. exp Alcohol-Related Disorders/
125. exp Anxiety Disorders/
126. exp Blood Glucose/
127. exp Hyperglycemia/
128. exp Hypoglycemia/
129. Cardiovascular Diseases/
130. Dementia/

131. exp Depressive Disorder/
132. Diabetes Mellitus/
133. exp Diabetes, Gestational/
134. Diabetic Foot/
135. exp Health Information Interoperability/
136. heart failure/
137. exp Myocardial Infarction/
138. exp Mental Disorders/
139. Neoplasms/
140. exp Pain Management/
141. Pain/
142. exp Papanicolaou Test/
143. exp Patient Reported Outcome Measures/
144. exp Physical Examination/
145. exp Pulmonary Disease, Chronic Obstructive/
146. exp Quality Indicators, Health Care/
147. exp Rehabilitation/
148. exp Self Care/
149. exp Self Efficacy/
150. Stroke/

(Acs or Acss or Acute Coronary Syndrome or Acute Coronary Syndromes or Adjustment Disorder or Adjustment Disorders or Alcohol Consumption or Alcohol Drinking or Alcohol Related Disorder or Alcohol Related Disorders or Alcoholic or Alcoholism or Anxiety or Anxious or Blood Glucose or Blood Sugar or Blood Sugars or Hyperglycemia or Hypoglycemia or Cancer or Cardiovascular Disease or Cardiovascular Diseases or Cerebrovascular Accident or Cerebrovascular Accidents or Chf or Chronic Obstructive Pulmonary Disease or Chronic Obstructive Pulmonary Disorder or Chronic Obstructive Pulmonary Disorders or Complication or Complications or Congestive Heart Failure or Copd or Coronary Artery Disease or Coronary Artery Diseases or Coronary Disease or Coronary Diseases or Cva or Cvas or Cvd or Cvds or Dementia or Dementias or Depressed or Depression or Depressive Disorder or Depressive Disorders or Depressive or Diabetes Mellitus or Diabetes or Diabetic Foot or Foot Complication or Foot Complications or Gestational Diabetes or Glycaemia or Glycaemic or Glycemia or Glycemic

151. or Health Care Evaluation Mechanisms or Health Care Quality Indicator or Health Care Quality Indicators or Health Information Interoperability or Healthcare Evaluation Mechanisms or Healthcare Quality Indicator or Healthcare Quality Indicators or Heart Failure or Interoperability or Interoperable or Managed Pain or Management Of Pain or Managing Pain or Mental Disorder or Mental Disorders or Mental Health or Mental Illness or Mental Illnesses or Mentally Healthy or Mentally Ill or Myocardial Infarction or Neoplasm or Neoplasms or Oncologist or Oncologists or Oncology or Pain Management or Pain or Pains or Pap Test or Pap Tests or Papanicolaou Test or Papanicolaou Tests or Patient Reported Experience Measure or Patient Reported Experience Measures or Patient Reported Outcome Measure or Patient Reported Outcome Measures or Physical Exam or Physical Examination or Physical Examinations or Physical Exams or Prem or Prems or Prom or Promotion or Proms or Psycho Social or Psychosocial or Quality Indicator or Quality indicators Of Health Care Quality Indicators or Rehabilitation or Self Care or Self Efficacy or Stroke or Strokes).ab,ti.

152. 121 or 122 or 123 or 124 or 125 or 126 or 127 or 128 or 129 or 130 or 131 or 132 or 133 or 134 or 135 or 136 or 137 or 138 or 139 or 140 or 141 or 142 or 143 or 144 or 145 or 146 or 147 or 148 or 149 or 150 or 151
153. limit 152 to yr="2010 -Current"
154. exp Allied Health Personnel/
155. exp Ambulatory Care Information Systems/
156. Ambulatory Care/
157. exp "Appointments and Schedules"/
158. exp Clinical Competence/
159. exp "Duration of Therapy"/
160. exp Patient Education as Topic/
161. exp Evidence-Based Practice/
162. exp Harm Reduction/
163. exp Health Behavior/
164. exp Health Care Costs/
165. exp "Health Care Economics and Organizations"/
166. exp "Health Care Facilities, Manpower, and Services"/
167. exp "Health Care Quality, Access, and Evaluation"/
168. exp Health Knowledge, Attitudes, Practice/
169. exp Health Plan Implementation/
170. exp Health Services Accessibility/
171. exp Healthcare Disparities/
172. exp Hospital Shared Services/
173. exp House Calls/
174. exp Length of Stay/
175. exp Office Visits/
176. exp Patient Acceptance of Health Care/
177. exp Patient Admission/
178. exp Patient Advocacy/
179. exp Prescriptions/
180. exp "Referral and Consultation"/
181. exp Universal Health Care/
182. (Access To Care or Access To Health Care or Access To Health Services or Access To Healthcare or Accessibility Of Health Services or Allied Health Personnel or Ambulatory Care Information System or Ambulatory Care Information Systems or Ambulatory Care or Appointment or Appointments or Care Access or Clinical Competence or Clinical Competencies or Clinical Competency or Consultation or Consultations or Cost or Costs or Duration Of Therapy or Patient Education or Patient Teaching or Evaluated Health Care or Evaluated Healthcare or Evaluating Health Care or Evaluating Healthcare or Evaluation Of Health Care or Evaluations Of Health Care or Harm Prevention or Harm Reduction or Health Attitude or Health Attitudes or Health Behavior or Health Behaviors or Health Behaviour or Health Behaviours or HealthCare or Health Care or Health Knowledge or Health Personnel or Health Plan Implementation or Health Service Accessibility or Health Services Accessibility or Healthy Environment or Healthy Environments or Hospital Shared Services or House Call or House Calls or Indirect Cost or indirect Costs or Knowledge Of Health or Length Of Stay or Length Of Therapy or Lengths Of Stay or Los or Office Visit or Office Visits or Patient Acceptance or Patient Admission or Patient Admissions or

Patient Advocacy or Prescription or Prescriptions or Prevention Of Harm or Quality Health Care or Quality Healthcare or Reduction Of Harm or Referral or Referrals or Return Visit or Return Visits or Schedule or Scheduled or Schedules or Therapy Duration or Therapy Durations or Therapy Length or Universal Health Care or Universal Healthcare or Unplanned).ab,ti.

183. 154 or 155 or 156 or 157 or 158 or 159 or 160 or 161 or 162 or 163 or 164 or 165 or 166 or 167 or 168 or 169 or 170 or 171 or 172 or 173 or 174 or 175 or 176 or 177 or 178 or 179 or 180 or 181 or 182
184. limit 183 to yr="2010 -Current"
185. exp Delivery of Health Care/
186. exp Frailty/
187. exp Global Health/
188. exp Health Equity/
189. exp Health Literacy/
190. exp Health Status Disparities/
191. exp Healthcare Disparities/
192. exp Income/
193. exp Insurance Coverage/
194. exp "International Classification of Functioning, Disability and Health"/
195. exp Marital Status/
196. exp Minority Health/
197. exp Oceanic Ancestry Group/
198. exp Patient Selection/
199. exp Population Dynamics/
200. exp Population Groups/
201. exp Poverty Areas/
202. exp Refusal to Treat/
203. Religion/
204. exp Social Discrimination/
205. exp Socioeconomic Factors/
206. exp Treatment Failure/
207. (Aboriginal or Communication Barriers or Delivery Of Health Care or Delivery Of Healthcare or Disability Evaluation or Discrimination or Equity or Frailty or Gender Minorities or Gender Minority or Global Health or Health Care Delivery or Health Care Disparities or Health Care Disparity or Health Equity or Health Literacy or Health Status Disparities or Health Status Disparity or Healthcare Delivery or Healthcare Disparities or Healthcare Disparity or Income or Incomes or Inequal or Inequalities or Inequality or Insurance Cover or Insurance Coverage or Insurance Coverages or Insurance Covered or "International Classification Of Functioning Disability and Health" or Marital Status or Minority Health or Oceanic Ancestry Group or Oceanic Ancestry Groups or Patient Selection or Population Dynamics or Population Group or Population Groups or Poverty Area or Poverty Areas or Poverty or Refusal To Treat or Religion or Religions or Safety Net or Sexual Minorities or Sexual Minority or Sexual Orientation or Sexual Orientations or Social Discrimination or Social Discriminations or Social Support or Treatment Failure or Treatment Failures or Treatment Preference or Treatment Preferences or Vulnerabilities or Vulnerability or Vulnerable).ab,ti.
208. 185 or 186 or 187 or 188 or 189 or 190 or 191 or 192 or 193 or 194 or 195 or 196 or 197 or 198 or 199 or 200 or 201 or 202 or 203 or 204 or 205 or 206 or 207

209. limit 208 to yr="2010 -Current"
210. 84 or 106 or 119 or 152 or 183 or 208
211. ((Systematic* adj3 (Review* or Overview*)) or (Methodologic* adj3 (Review* or Overview*))).ti,ab,kf,kw.
212. Meta-Analysis.pt.
213. meta-analysis/ or systematic review/ or meta-analysis as topic/ or "meta analysis (topic)"/ or "systematic review (topic)"/
214. exp meta-analysis/
215. Meta Analy*.tw.
216. Metanaly*.tw.
217. Metaanaly*.tw.
218. Met Analy*.tw.
219. ((overview$ or review or synthesis or summary or cochrane or analysis) and (reviews or meta-analyses or articles)).ti.
220. (meta-review or metareview).ti,ab.
221. ((overview$ or reviews) and (systematic or cochrane)).ti.
222. (reviews adj2 (meta or published or quality or included or summar$)).ab.
223. cochrane reviews.ab.
224. (evidence and (reviews or meta-analyses)).ti.
225. 211 or 212 or 213 or 214 or 215 or 216 or 217 or 218 or 219 or 220 or 221 or 222 or 223 or 224
226. 46 and 52 and 210 and 225
227. limit 226 to yr="2010 -Current"

9. Ovid Global Health

1. exp health centres/

2. exp health care/

3. exp community health/ or exp community programmes/

4. exp community health services/

5. exp health programmes/ or exp health services/

6. exp general practitioners/

7. exp personnel/

8. exp health care workers/

9. exp home care/

10. exp long term care/

11. exp nurses/

12. exp nursing homes/

13. exp nursing/

14. exp outpatient services/

15. exp patient care/

16. exp physicians/

17. exp Primary Health Care/

18. exp practice/ or exp "code of practice"/

19. exp rural communities/

20. exp rural health/

21. exp rural areas/

22. exp suburban areas/

23. telemedicine/

24. (ambulatory care or Care Continuity or Chsld or Chslds or Clsc or Clsc or Community Care or Community Clinic or Community Clinics or Community Doctor or Community Doctors or Community Facilities or Community Facility or Community Health Center or Community Health Centers or Community Health Centre or Community Health Centres or Community Health Nurse or Community Health Nurses or Community Health Nursing or Community Health Plan or Community Health Planning or Community Health Plans or Community Health Service or Community Health Services or Community Medicine or Community Mental Health Service or Community Mental Health Services or Community Nurse or Community Nurses or Community Nursing or Community Physician or Community Physicians or Community Practice or Community Practices or Community Program or Community Programs or Community Service or Community Services or Continuity of Care or Counseling or Counselling or Delivery of Health Care or Delivery of Healthcare or Distance Counseling or Distance Counselling

or Elder Care or Elderly Nursing Home or Elderly Nursing Homes or Family Clinic or Family Clinics or Family Doctor or Family Doctors or Family Medicine Group or Family Medicine Groups or Family Medicine or Family Physician or Family Physicians or Family Practice or Family Practices or General Practice or General Practices or General Practitioner or General Practitioners or Group Practice or Group Practices or Health Care Delivery or Health Care Professional or Health Care Professionals or Health Care Provider or Health Care Providers or Health Care Team or Health Care Teams or Health Personnel or Health Professional or Health Professionals or Health Services Administration or Healthcare Delivery or Healthcare Professional or Healthcare Professionals or Healthcare Provider or Healthcare Providers or Healthcare Team or Healthcare Teams or Home Care or Home Health or Home Nurse or Home Nurses or Home Nursing or Interactive Consultation or Interactive Consultations or Interactive Service or Interactive Services or Interdisciplinary Team or Interdisciplinary Teams or Intermediate Care Facility or Intermediate Care or Interprofessional Team or Interprofessional Teams or Local Community Service Center or Local Community Service Centers or Local Community Service Centre or Local Community Service Centres or Long-Term Care Center or Long-Term Care Centers or Long-Term Care Centre or Long-Term Care Centres or Long-Term Care Home or Long-Term Care Homes or Multidisciplinary Team or Multidisciplinary Teams or Multidiscipline Team or Multidiscipline Teams or Nurse Management or Nurse or Nurse-Delivered or Nurse-Led or Nurse-Managed or Nurse-Run or Nurses Management or Nurses or Nurses-Led or Nursing Care or Nursing Delivered or Nursing Home or Nursing Homes or Nursing Management or Nursing or Nursing-Delivered or Nursing-Led or Nursing-Managed or Nursing-Run or Office Nurse or Office Nurses or Office Nursing or Outpatient or Outpatients or Patient Care Team or Patient Care Teams or Primary Care or Primary Health Care or Primary Healthcare or Professional Autonomy or Professional Practice or Professional Practices or Remote or Residential Care Center or Residential Care Centers or Residential Care Centre or Residential Care Centres or Residential Care or Respite Care or Retirement Home or Retirement Homes or Rural Health or Rural Nurse or Rural Nurses or Rural Nursing or Staff Nurse or Staff Nurses or Suburban Health Service or Suburban or Team or Teams or Teamwork or Telemedicine or Tele-Medicine or Telenurse or Tele-Nurse or Telenurses or Tele-Nurses or Telenursing or Tele-Nursing or Telerehabilitation or Tele-Rehabilitation or Walk-In Clinic or Walk-In Clinics).ab,ti.

25. 1 or 2 or 3 or 4 or 5 or 6 or 7 or 8 or 9 or 10 or 11 or 12 or 13 or 14 or 15 or 16 or 17 or 18 or 19 or 20 or 21 or 22 or 23 or 24

26. exp nursing/

27. exp nurses/

28. exp careproviders/

29. (Advanced Nurse Practitioner or Advanced Nurse Practitioners or (ANP and Nurse) or (ANP and Nurses) or (ANP and Nursing) or Advanced Nursing Practice or Advanced Nursing Practices or Advanced Practice Nurse or Advanced Practice Nurses or Advanced Practice Nursing or (APN and Nurse) or (APN and Nurses) or (APN and Nursing) or Advanced Practice Registered Nurse or Advanced Practice Registered Nurses or Advanced Practice Registered Nursing or (APRN and Nurse) or (APRN and Nurses) or (APRN and Nursing) or Emergency Nurse Practitioner or Emergency Nurse Practitioners or (APRN and Nurse) or (APRN and Nurses) or (APRN and Nursing) or Family nurse practitioner or Family nurse practitioners or (FNP and Nurse) or (FNP and Nurses) or (FNP and Nursing) or Nurse Practitioner or Nurse Practitioners or (NP and Nurse) or (NP and Nurses) or (NP and Nursing) or Primary Care Nurse Practitioner or Primary Care Nurse Practitioners or Primary Health Care Nurse Practitioner or (PCNP and Nurse) or (PCNP and Nurses) or (PCNP and Nursing) or Primary Health Care Nurse Practitioners or Primary Healthcare Nurse Practitioner or Primary Health Care Nurse Practitioner or Primary Healthcare Nurse Practitioners or Primary Health care Nurse Practitioners or (PHCNP and Nurse) or (PHCNP and Nurses) or (PHCNP and Nursing)).ab,ti.

30. 26 or 27 or 28 or 29

31. biomarkers/

32. hypertension/

33. exp cholesterol/

34. exp hypercholesterolaemia/

35. exp blood lipids/

36. exp low density lipoprotein/

37. exp high density lipoprotein/

38. exp chronic disease/

39. exp consumer behaviour/

40. exp "cost analysis"/ or exp "cost benefit analysis"/ or exp "cost effectiveness analysis"/

41. exp costs/ or exp health care costs/

42. exp health care/

43. exp diagnostic techniques/

44. exp screening/

45. exp haemoglobin a1/ or exp blood sugar/

46. exp guidelines/

47. exp resource allocation/ or exp resource management/ or exp resources/ or exp task shifting/ or exp resource utilization/

48. exp health services/

49. exp health/

50. exp health indicators/

51. exp job satisfaction/

52. exp mortality/

53. exp health care/ or exp "quality of care"/

54. exp assessment/ or exp health impact assessment/

55. pain/

56. exp work satisfaction/ or exp consumer satisfaction/

57. exp prescriptions/

58. exp roles/

59. exp performance/ or exp productivity/

60. exp task analysis/

61. exp patient compliance/

62. (Appointment or Appointments or Attendance or Biomarker or Biomarkers or Hypertension or Care Process or Care Processes or Cholesterol or Hypercholesterolemia or Chronic Condition or Chronic Conditions or Chronic Disease or Chronic Diseases or Chronic Illness or Chronic Illnesses or Clinical Outcome or Clinical Outcomes or Clinical Parameter or Clinical Parameters or Compliance or Compliant or Consultation or Consultations or Consumer Behavior or Consumer Behaviors or Consumer Behaviour or Consumer Behaviours or Cost Analyses or Cost Analysis or Cost or Costs or Death or Deaths or Delivery Of Health Care or Delivery Of Healthcare or Diagnostic Test or Diagnostic Testing or Diagnostic Tests or Economic Analyses or Economic Analysis or Economic Evaluation or Economic Evaluations or Emergency Service or Emergency Services or Follow Up Appointment or Follow Up Appointments or Follow Up Visit or Follow Up Visits or Glycated Haemoglobin A or Glycated Hemoglobin A or Guideline Adherence or HbA1C or Hdl or Health Care or Health Planning or Health Related Quality Of Life or Health Resource or Health Resources or Health Service or Health Services or Health Status or Healthcare Delivery or Healthcare Process Assessment or Healthcare Quality or Healthcare or High Density Lipoprotein or Hospital Admission or Hospital Admissions or Hospital Referral or Hospital Referrals or Hrqol or Hypertension or Indicator or Indicators or Job Satisfaction or Ldl or Lipid Profile or Lipid Profiles or Low Density Lipoprotein or Manpower or Mortalities or Mortality or Outcome Assessment or Outcome Assessments or Pain or Pains or Patient Satisfaction or Personal Satisfaction or Prefer or Preference or Preferences or Preferred or Prescribe or Prescribed or Prescription or Prescriptions or Process Of Care or Processes Of Care or Professional Role or Professional Roles or Qol or Quality Indicator or Quality Indicators or Quality Of Health Care or Quality Of Healthcare or Quality Of Life or Referral or Referrals or Return Appointment or Return Appointments or Return Visit or Return Visits or Routine Diagnostic or Routine Test or Routine Testing or Routine Tests or Satisfaction or Satisfactory or Satisfied or Schedule or Schedules or Task Analyses or Task Analysis or Task Performance or Treatment Adherence or Treatment Adherent or Treatment Compliance or Treatment Compliant or Wellness).ab,ti.

63. 31 or 32 or 33 or 34 or 35 or 36 or 37 or 38 or 39 or 40 or 41 or 42 or 43 or 44 or 45 or 46 or 47 or 48 or 49 or 50 or 51 or 52 or 53 or 54 or 55 or 56 or 57 or 58 or 59 or 60 or 61 or 62

64. exp anthropometric dimensions/ or exp body measurements/

65. exp body weight/

66. diet/

67. exp food/

68. exp nutrition/

69. exercise/

70. exp health education/

71. exp health promotion/

72. exp lifestyle/

73. exp mental health/

74. exp nutritional assessment/

75. exp nutritional status/

76. exp poverty/

77. exp disease prevention/

78. exp preventive medicine/

79. exp smoking cessation/

80. exp tobacco smoking/ or exp cigarettes/ or exp passive smoking/ or exp tobacco/ or exp tobacco control/

81. exp smoking/

82. exp teaching/

83. (Anthropometric or Anthropometrical or Anthropometrically or Anthropometrics or Anthropometry or Body Weight or Condition Specific or Culturally Competent Care or Culturally Diverse or Diet or Dietary or Dietetical or Dietetically or Dietetics or Diets or Emotional Health or Exercise or Food Regimen or Food Regimens or Health Education or Health Prevention or Health Promotion or Healthy Habit or Healthy Habits or Healthy Life or Healthy Life Habits or Healthy Living or Life Style or Lifestyle or Low Income or Mental Health or Nutrition Assessment or Nutrition or Nutritional Status or Nutritional or Physical Activity or Poverty or Preventive Health Service or Preventive Health Services or Promotion of Health or Promotion or Quit Smoking or Quitting Smoking or Second Hand Smoke or Smoke or Smoked or Smoker or Smokers or Smokes or Smoking or Socioeconomic Status or Stop Smoking or Stopping Smoking or Stress Level or Stress Levels or Taught or Teach or Teaching or Tobacco or Weight).ab,ti.

84. exp adolescent/

85. exp child development/

86. exp adolescent development/ or exp adult development/

87. exp immunization/

88. exp prenatal care/

89. exp maternity services/

90. exp paediatrics/

91. exp prevention/

92. exp disease prevention/

93. sexually transmitted diseases/

94. exp vaccination/

95. (Adolescence or Adolescent or Adolescents or Child Development or Childhood or Healthy Development or Human Development or Immunisation or Immunise or Immunised or Immunises or Immunization or Immunize or Immunized or Immunizes or Laboratory Test or Maternal Health Service or Maternal Health Services or Paediatric or Paediatrics or Pediatric or Pediatrics or Pregnancy or Prevent or Prevented or Prevention or Prevents or Primary Prevention or Secondary Prevention or Sexually Transmitted or Vaccinate or Vaccinated or Vaccinates or Vaccination or Vaccine or Vaccines or Youth or Youths).ab,ti.

96. exp heart diseases/

97. exp alcohol intake/ or exp alcoholic beverages/ or exp alcoholism/

98. exp anxiety/

99. exp blood sugar/

100. exp hyperglycaemia/

101. exp hypoglycaemia/

102. cardiovascular diseases/

103. dementia/

104. exp depression/ or exp mental disorders/

105. diabetes mellitus/

106. diabetes/

107. exp myocardial infarction/

108. neoplasms/

109. pain/

110. exp Papanicolaou Test/

111. exp chronic obstructive pulmonary disease/

112. exp "quality of care"/

113. exp indicators/

114. exp health indicators/

115. exp health care/

116. exp rehabilitation/

117. exp self care/

118. stroke/

119. (Acs or Acss or Acute Coronary Syndrome or Acute Coronary Syndromes or Adjustment Disorder or Adjustment Disorders or Alcohol Consumption or Alcohol Drinking or Alcohol Related Disorder or Alcohol Related Disorders or Alcoholic or Alcoholism or Anxiety or Anxious or Blood Glucose or Blood Sugar or Blood Sugars or Hyperglycemia or Hypoglycemia or Cancer or Cardiovascular Disease or Cardiovascular Diseases or Cerebrovascular Accident or Cerebrovascular Accidents or Chf or Chronic Obstructive Pulmonary Disease or Chronic Obstructive Pulmonary Disorder or Chronic Obstructive Pulmonary Disorders or Complication or Complications or Congestive Heart Failure or Copd or Coronary Artery Disease or Coronary Artery Diseases or Coronary Disease or Coronary Diseases or Cva or Cvas or Cvd or Cvds or Dementia or Dementias or Depressed or Depression or Depressive Disorder or Depressive Disorders or Depressive or Diabetes Mellitus or Diabetes or Diabetic Foot or Foot Complication or Foot Complications or Gestational Diabetes or Glycaemia or Glycaemic or Glycemia or Glycemic or Health Care Evaluation Mechanisms or Health Care Quality Indicator or Health Care

Quality Indicators or Health Information Interoperability or Healthcare Evaluation Mechanisms or Healthcare Quality Indicator or Healthcare Quality Indicators or Heart Failure or Interoperability or Interoperable or Managed Pain or Management Of Pain or Managing Pain or Mental Disorder or Mental Disorders or Mental Health or Mental Illness or Mental Illnesses or Mentally Healthy or Mentally Ill or Myocardial Infarction or Neoplasm or Neoplasms or Oncologist or Oncologists or Oncology or Pain Management or Pain or Pains or Pap Test or Pap Tests or Papanicolaou Test or Papanicolaou Tests or Patient Reported Experience Measure or Patient Reported Experience Measures or Patient Reported Outcome Measure or Patient Reported Outcome Measures or Physical Exam or Physical Examination or Physical Examinations or Physical Exams or Prem or Prems or Prom or Promotion or Proms or Psycho Social or Psychosocial or Quality Indicator or Quality indicators Of Health Care Quality Indicators or Rehabilitation or Self Care or Self Efficacy or Stroke or Strokes).ab,ti.

120. 96 or 97 or 98 or 99 or 100 or 101 or 102 or 103 or 104 or 105 or 106 or 107 or 108 or 109 or 110 or 111 or 112 or 113 or 114 or 115 or 116 or 117 or 118 or 119

121. exp health care workers/

122. exp personnel/

123. exp primary health care/

124. exp outpatient services/

125. exp professional competence/

126. exp duration/

127. exp therapy/

128. exp patient education/

129. exp health behaviour/

130. exp health care costs/

131. exp economics/

132. exp organizations/

133. exp health care workers/ or exp health centres/ or exp health clinics/ or exp health services/

134. exp health maintenance organizations/

135. exp access/

136. exp "quality of care"/

137. exp knowledge/

138. exp attitudes/

139. exp project implementation/

140. exp health inequalities/

141. exp hospital stay/

142. exp health care utilization/

143. exp acceptability/

144. exp patient care/

145. exp hospital admission/ or exp hospital care/

146. exp health policy/

147. exp prescriptions/

148. exp universal health coverage/

149. (Access To Care or Access To Health Care or Access To Health Services or Access To Healthcare or Accessibility Of Health Services or Allied Health Personnel or Ambulatory Care Information System or Ambulatory Care Information Systems or Ambulatory Care or Appointment or Appointments or Care Access or Clinical Competence or Clinical Competencies or Clinical Competency or Consultation or Consultations or Cost or Costs or Duration Of Therapy or Patient Education or Patient Teaching or Evaluated Health Care or Evaluated Healthcare or Evaluating Health Care or Evaluating Healthcare or Evaluation Of Health Care or Evaluations Of Health Care or Harm Prevention or Harm Reduction or Health Attitude or Health Attitudes or Health Behavior or Health Behaviors or Health Behaviour or Health Behaviours or HealthCare or Health Care or Health Knowledge or Health Personnel or Health Plan Implementation or Health Service Accessibility or Health Services Accessibility or Healthy Environment or Healthy Environments or Hospital Shared Services or House Call or House Calls or Indirect Cost or indirect Costs or Knowledge Of Health or Length Of Stay or Length Of Therapy or Lengths Of Stay or Los or Office Visit or Office Visits or Patient Acceptance or Patient Admission or Patient Admissions or Patient Advocacy or Prescription or Prescriptions or Prevention Of Harm or Quality Health Care or Quality Healthcare or Reduction Of Harm or Referral or Referrals or Return Visit or Return Visits or Schedule or Scheduled or Schedules or Therapy Duration or Therapy Durations or Therapy Length or Universal Health Care or Universal Healthcare or Unplanned).ab,ti.

150. 121 or 122 or 123 or 124 or 125 or 126 or 127 or 128 or 129 or 130 or 131 or 132 or 133 or 134 or 135 or 136 or 137 or 138 or 139 or 140 or 141 or 142 or 143 or 144 or 145 or 146 or 147 or 148 or 149

151. exp health care/

152. exp literacy/

153. exp health education/

154. exp health inequalities/

155. exp disparity/

156. exp income/

157. exp health insurance/

158. exp marital status/

159. exp minorities/ or exp ethnic groups/

160. exp patients/

161. exp population dynamics/

162. exp populations/

163. exp poverty/

164. religion/

165. exp discrimination/

166. exp socioeconomics/

167. exp treatment failure/

168. (Aboriginal or Communication Barriers or Delivery Of Health Care or Delivery Of Healthcare or Disability Evaluation or Discrimination or Equity or Frailty or Gender Minorities or Gender Minority or Global Health or Health Care Delivery or Health Care Disparities or Health Care Disparity or Health Equity or Health Literacy or Health Status Disparities or Health Status Disparity or Healthcare Delivery or Healthcare Disparities or Healthcare Disparity or Income or Incomes or Inequal or Inequalities or Inequality or Insurance Cover or Insurance Coverage or Insurance Coverages or Insurance Covered or "International Classification Of Functioning Disability and Health" or Marital Status or Minority Health or Oceanic Ancestry Group or Oceanic Ancestry Groups or Patient Selection or Population Dynamics or Population Group or Population Groups or Poverty Area or Poverty Areas or Poverty or Refusal To Treat or Religion or Religions or Safety Net or Sexual Minorities or Sexual Minority or Sexual Orientation or Sexual Orientations or Social Discrimination or Social Discriminations or Social Support or Treatment Failure or Treatment Failures or Treatment Preference or Treatment Preferences or Vulnerabilities or Vulnerability or Vulnerable).ab,ti.

169. 151 or 152 or 153 or 154 or 155 or 156 or 157 or 158 or 159 or 160 or 161 or 162 or 163 or 164 or 165 or 166 or 167 or 168

170. ((Systematic* adj3 (Review* or Overview*)) or (Methodologic* adj3 (Review* or Overview*))).af.

171. Meta-Analysis.af.

172. meta-analysis/ or systematic review/ or meta-analysis as topic/ or "meta analysis (topic)"/ or "systematic review (topic)"/

173. exp meta-analysis/

174. Meta Analy*.tw.

175. Metanaly*.tw.

176. Metaanaly*.tw.

177. Met Analy*.tw.

178. ((overview$ or review or synthesis or summary or cochrane or analysis) and (reviews or meta-analyses or articles)).ti.

179. (meta-review or metareview).ti,ab.

180. ((overview$ or reviews) and (systematic or cochrane)).ti.

181. (reviews adj2 (meta or published or quality or included or summar$)).ab.

182. cochrane reviews.ab.

183. (evidence and (reviews or meta-analyses)).ti.

184. 64 or 65 or 66 or 67 or 68 or 69 or 70 or 71 or 72 or 73 or 74 or 75 or 76 or 77 or 78 or 79 or 80 or 81 or 82 or 83

185. 84 or 85 or 86 or 87 or 88 or 89 or 90 or 91 or 92 or 93 or 94 or 95

186. 63 or 120 or 150 or 169 or 184 or 185

187. 170 or 171 or 172 or 173 or 174 or 175 or 176 or 177 or 178 or 179 or 180 or 181 or 182 or 183

188. 25 and 30 and 186 and 187

189. limit 188 to yr="2010 -Current"

10. Joanna Briggs Institute (JBI) EBP

1. exp Ambulatory Care Facilities/
2. Ambulatory Care Facilities.af.
3. Ambulatory Care/
4. Ambulatory Care.af.
5. exp Community Health Centers/
6. Community Health Centers.af.
7. exp Community Health Nursing/
8. Community Health Nursing.af.
9. exp Community Health Planning/
10. Community Health Planning.af.
11. exp Community Health services/
12. Community Health services.af.
13. exp Community medicine/
14. Community medicine.af.
15. Community Mental Health Services/
16. Community Mental Health Services.af.
17. exp Delivery of Health Care/
18. Delivery of Health Care.af.
19. exp family practice/
20. family practice.af.
21. exp General Practice/
22. General Practice.af.
23. exp General Practitioners/
24. General Practitioners.af.
25. exp group practice/
26. group practice.af.
27. exp Health Personnel/
28. Health Personnel.af.
29. exp Health Services Administration/
30. Health Services Administration.af.
31. exp Home Care Services/
32. Home Care Services.af.
33. exp Homes for the Aged/
34. Homes for the Aged.af.
35. exp Housing for the Elderly/
36. Housing for the Elderly.af.
37. exp Intermediate Care Facilities/
38. Intermediate Care Facilities.af.
39. exp Long-Term Care/
40. Long-Term Care.af.
41. exp Nurses, Community Health/

42. Community Health Nurses.af.
43. exp Nursing Care/
44. Nursing Care.af.
45. exp Nursing Homes/
46. Nursing Homes.af.
47. exp Nursing Stations/
48. Nursing Stations.af.
49. exp Nursing/
50. Nursing.af.
51. exp Nursing, team/
52. team nursing.af.
53. exp Office Nursing/
54. office nursing.af.
55. exp outpatients/
56. outpatients.af.
57. exp Patient Care Team/
58. Patient Care Team.af.
59. exp Physicians, Family/
60. Family Physicians.af.
61. exp Physicians, Primary Care/
62. Primary Care Physicians.af.
63. exp Primary Care Nursing/
64. Primary Care Nursing.af.
65. exp Primary Health Care/
66. Primary Health Care.af.
67. exp Professional Autonomy/
68. Professional Autonomy.af.
69. exp Professional Practice/
70. Professional Practice.af.
71. exp Remote Consultation/
72. Remote Consultation.af.
73. exp Respite Care/
74. Respite Care.af.
75. exp Rural Health Services/
76. Rural Health Services.af.
77. exp Rural Health/
78. Rural Health.af.
79. exp Rural Nursing/
80. Rural Nursing.af.
81. exp Suburban Health Services/
82. Suburban Health Services.af.
83. Telemedicine/
84. Telemedicine.af.

85. exp Telenursing/
86. Telenursing.af.
87. exp Telerehabilitation/
88. Telerehabilitation.af.
89. (ambulatory care or Care Continuity or Chsld or Chslds or Clsc or Clsc or Community Care or Community Clinic or Community Clinics or Community Doctor or Community Doctors or Community Facilities or Community Facility or Community Health Center or Community Health Centers or Community Health Centre or Community Health Centres or Community Health Nurse or Community Health Nurses or Community Health Nursing or Community Health Plan or Community Health Planning or Community Health Plans or Community Health Service or Community Health Services or Community Medicine or Community Mental Health Service or Community Mental Health Services or Community Nurse or Community Nurses or Community Nursing or Community Physician or Community Physicians or Community Practice or Community Practices or Community Program or Community Programs or Community Service or Community Services or Continuity of Care or Counseling or Counselling or Delivery of Health Care or Delivery of Healthcare or Distance Counseling or Distance Counselling or Elder Care or Elderly Nursing Home or Elderly Nursing Homes or Family Clinic or Family Clinics or Family Doctor or Family Doctors or Family Medicine Group or Family Medicine Groups or Family Medicine or Family Physician or Family Physicians or Family Practice or Family Practices or General Practice or General Practices or General Practitioner or General Practitioners or Group Practice or Group Practices or Health Care Delivery or Health Care Professional or Health Care Professionals or Health Care Provider or Health Care Providers or Health Care Team or Health Care Teams or Health Personnel or Health Professional or Health Professionals or Health Services Administration or Healthcare Delivery or Healthcare Professional or Healthcare Professionals or Healthcare Provider or Healthcare Providers or Healthcare Team or Healthcare Teams or Home Care or Home Health or Home Nurse or Home Nurses or Home Nursing or Interactive Consultation or Interactive Consultations or Interactive Service or Interactive Services or Interdisciplinary Team or Interdisciplinary Teams or Intermediate Care Facility or Intermediate Care or Interprofessional Team or Interprofessional Teams or Local Community Service Center or Local Community Service Centers or Local Community Service Centre or Local Community Service Centres or Long-Term Care Center or Long-Term Care Centers or Long-Term Care Centre or Long-Term Care Centres or Long-Term Care Home or Long-Term Care Homes or Multidisciplinary Team or Multidisciplinary Teams or Multidiscipline Team or Multidiscipline Teams or Nurse Management or Nurse or Nurse-Delivered or Nurse-Led or Nurse-Managed or Nurse-Run or Nurses Management or Nurses or Nurses-Led or Nursing Care or Nursing Delivered or Nursing Home or Nursing Homes or Nursing Management or Nursing or Nursing-Delivered or Nursing-Led or Nursing-Managed or Nursing-Run or Office Nurse or Office Nurses or Office Nursing or Outpatient or Outpatients or Patient Care Team or Patient Care Teams or Primary Care or Primary Health Care or Primary Healthcare or Professional Autonomy or Professional Practice or Professional Practices or Remote or Residential Care Center or Residential Care Centers or Residential Care Centre or Residential Care Centres or Residential Care or Respite Care or Retirement Home or Retirement Homes or Rural Health or Rural Nurse or Rural Nurses or Rural Nursing or Staff Nurse or Staff Nurses or Suburban Health Service or Suburban or Team or Teams or Teamwork or Telemedicine or Tele-Medicine or Telenurse or Tele-Nurse or Telenurses or Tele-Nurses or Telenursing or Tele-Nursing or Telerehabilitation or Tele-Rehabilitation or Walk-In Clinic or Walk-In Clinics).ab,ti.
90. 1 or 2 or 3 or 4 or 5 or 6 or 7 or 8 or 9 or 10 or 11 or 12 or 13 or 14 or 15 or 16 or 17 or 18 or 19 or 20 or 21 or 22 or 23 or 24 or 25 or 26 or 27 or 28 or 29 or 30 or 31 or 32 or 33 or 34 or 35 or 36 or 37 or 38 or 39 or 40 or 41 or 42 or 43 or 44 or 45 or 46 or 47 or 48 or 49 or 50 or 51 or 52 or 53 or

54 or 55 or 56 or 57 or 58 or 59 or 60 or 61 or 62 or 63 or 64 or 65 or 66 or 67 or 68 or 69 or 70 or 71 or 72 or 73 or 74 or 75 or 76 or 77 or 78 or 79 or 80 or 81 or 82 or 83 or 84 or 85 or 86 or 87 or 88 or 89

91. limit 90 to yr="2010 -Current"
92. exp Advanced Practice Nursing/
93. Advanced Practice Nursing.af.
94. exp Family Nurse Practitioners/
95. Family Nurse Practitioners.af.
96. exp Nurse Practitioners/
97. Nurse Practitioners.af.
98. (Advanced Nurse Practitioner or Advanced Nurse Practitioners or (ANP and Nurse) or (ANP and Nurses) or (ANP and Nursing) or Advanced Nursing Practice or Advanced Nursing Practices or Advanced Practice Nurse or Advanced Practice Nurses or Advanced Practice Nursing or (APN and Nurse) or (APN and Nurses) or (APN and Nursing) or Advanced Practice Registered Nurse or Advanced Practice Registered Nurses or Advanced Practice Registered Nursing or (APRN and Nurse) or (APRN and Nurses) or (APRN and Nursing) or Emergency Nurse Practitioner or Emergency Nurse Practitioners or (APRN and Nurse) or (APRN and Nurses) or (APRN and Nursing) or Family nurse practitioner or Family nurse practitioners or (FNP and Nurse) or (FNP and Nurses) or (FNP and Nursing) or Nurse Practitioner or Nurse Practitioners or (NP and Nurse) or (NP and Nurses) or (NP and Nursing) or Primary Care Nurse Practitioner or Primary Care Nurse Practitioners or Primary Health Care Nurse Practitioner or (PCNP and Nurse) or (PCNP and Nurses) or (PCNP and Nursing) or Primary Health Care Nurse Practitioners or Primary Healthcare Nurse Practitioner or Primary Health Care Nurse Practitioner or Primary Healthcare Nurse Practitioners or Primary Health care Nurse Practitioners or (PHCNP and Nurse) or (PHCNP and Nurses) or (PHCNP and Nursing)).ab,ti.
99. 92 or 93 or 94 or 95 or 96 or 97 or 98
100. limit 99 to yr="2010 -Current"
101. exp "Appointments and Schedules"/
102. "Appointments and Schedules".af.
103. Biomarkers/
104. Biomarkers.af.
105. Hypertension/
106. Hypertension.af.
107. exp Cholesterol/
108. Cholesterol.af.
109. exp Hypercholesterolemia/
110. Hypercholesterolemia.af.
111. exp Chronic Disease/
112. Chronic Disease.af.
113. exp Consumer Behavior/
114. Consumer Behavior.af.
115. exp "Costs and Cost Analysis"/
116. "Costs and Cost Analysis".af.
117. exp Delivery of Health Care/
118. Delivery of Health Care.af.

119. exp Diagnostic Tests, Routine/
120. Routine Diagnostic Tests.af.
121. exp Glycated Hemoglobin A/
122. Glycated Hemoglobin A.af.
123. exp Guideline Adherence/
124. Guideline Adherence.af.
125. exp Health Resources/
126. Health Resources.af.
127. exp Health Services/
128. Health Services.af.
129. exp Health Status Indicators/
130. Health Status Indicators.af.
131. exp Health Status/
132. Health Status.af.
133. exp Job Satisfaction/
134. Job Satisfaction.af.
135. exp Mortality/
136. Mortality.af.
137. exp "Outcome and Process Assessment, Health Care"/
138. "Health Care Outcome and Process Assessment".af.
139. exp Outcome Assessment, Health Care/
140. Health Care Outcome Assessment.af.
141. Pain/
142. Pain.af.
143. exp Patient Satisfaction/
144. Patient Satisfaction.af.
145. exp Personal Satisfaction/
146. Personal Satisfaction.af.
147. exp Prescriptions/
148. Prescriptions.af.
149. exp Professional Role/
150. Professional Role.af.
151. exp Quality of Health Care/
152. Quality of Health Care.af.
153. exp "Referral and Consultation"/
154. "Referral and Consultation".af.
155. exp "Task Performance and Analysis"/
156. "Task Performance and Analysis".af.
157. exp "Treatment Adherence and Compliance"/
158. "Treatment Adherence and Compliance".af.
159. (Appointment or Appointments or Attendance or Biomarker or Biomarkers or Hypertension or Care Process or Care Processes or Cholesterol or Hypercholesterolemia or Chronic Condition or Chronic Conditions or Chronic Disease or Chronic Diseases or Chronic Illness or Chronic

Illnesses or Clinical Outcome or Clinical Outcomes or Clinical Parameter or Clinical Parameters or Compliance or Compliant or Consultation or Consultations or Consumer Behavior or Consumer Behaviors or Consumer Behaviour or Consumer Behaviours or Cost Analyses or Cost Analysis or Cost or Costs or Death or Deaths or Delivery Of Health Care or Delivery Of Healthcare or Diagnostic Test or Diagnostic Testing or Diagnostic Tests or Economic Analyses or Economic Analysis or Economic Evaluation or Economic Evaluations or Emergency Service or Emergency Services or Follow Up Appointment or Follow Up Appointments or Follow Up Visit or Follow Up Visits or Glycated Haemoglobin A or Glycated Hemoglobin A or Guideline Adherence or HbA1C or Hdl or Health Care or Health Planning or Health Related Quality Of Life or Health Resource or Health Resources or Health Service or Health Services or Health Status or Healthcare Delivery or Healthcare Process Assessment or Healthcare Quality or Healthcare or High Density Lipoprotein or Hospital Admission or Hospital Admissions or Hospital Referral or Hospital Referrals or Hrqol or Hypertension or Indicator or Indicators or Job Satisfaction or Ldl or Lipid Profile or Lipid Profiles or Low Density Lipoprotein or Manpower or Mortalities or Mortality or Outcome Assessment or Outcome Assessments or Pain or Pains or Patient Satisfaction or Personal Satisfaction or Prefer or Preference or Preferences or Preferred or Prescribe or Prescribed or Prescription or Prescriptions or Process Of Care or Processes Of Care or Professional Role or Professional Roles or Qol or Quality Indicator or Quality Indicators or Quality Of Health Care or Quality Of Healthcare or Quality Of Life or Referral or Referrals or Return Appointment or Return Appointments or Return Visit or Return Visits or Routine Diagnostic or Routine Test or Routine Testing or Routine Tests or Satisfaction or Satisfactory or Satisfied or Schedule or Schedules or Task Analyses or Task Analysis or Task Performance or Treatment Adherence or Treatment Adherent or Treatment Compliance or Treatment Compliant or Wellness).ab,ti.

160. 101 or 102 or 103 or 104 or 105 or 106 or 107 or 108 or 109 or 110 or 111 or 112 or 113 or 114 or 115 or 116 or 117 or 118 or 119 or 120 or 121 or 122 or 123 or 124 or 125 or 126 or 127 or 128 or 129 or 130 or 131 or 132 or 133 or 134 or 135 or 136 or 137 or 138 or 139 or 140 or 141 or 142 or 143 or 144 or 145 or 146 or 147 or 148 or 149 or 150 or 151 or 152 or 153 or 154 or 155 or 156 or 157 or 158 or 159
161. limit 160 to yr="2010 -Current"
162. exp Anthropometry/
163. Anthropometry.af.
164. exp Body Weight/
165. Body Weight.af.
166. exp Culturally Competent Care/
167. Culturally Competent Care.af.
168. Diet/
169. Diet.af.
170. exp "Diet, Food, and Nutrition"/
171. "Diet, Food, and Nutrition".af.
172. Exercise/
173. Exercise.af.
174. exp Health Education/
175. Health Education.af.
176. exp Health Promotion/
177. Health Promotion.af.
178. exp Healthy Lifestyle/

179. Healthy Lifestyle.af.
180. exp Mental Health/
181. Mental Health.af.
182. exp Nutrition Assessment/
183. Nutrition Assessment.af.
184. exp Nutritional Status/
185. Nutritional Status.af.
186. exp Poverty/
187. Poverty.af.
188. exp Preventive Health Services/
189. Preventive Health Services.af.
190. exp Smoking Cessation/
191. Smoking Cessation.af.
192. exp Smoking Prevention/
193. Smoking Prevention.af.
194. exp Smoking/
195. Smoking.af.
196. exp Teaching/
197. Teaching.af.
198. exp Tobacco Smoke Pollution/
199. Tobacco Smoke Pollution.af.
200. (Anthropometric or Anthropometrical or Anthropometrically or Anthropometrics or Anthropometry or Body Weight or Condition Specific or Culturally Competent Care or Culturally Diverse or Diet or Dietary or Dietetical or Dietetically or Dietetics or Diets or Emotional Health or Exercise or Food Regimen or Food Regimens or Health Education or Health Prevention or Health Promotion or Healthy Habit or Healthy Habits or Healthy Life or Healthy Life Habits or Healthy Living or Life Style or Lifestyle or Low Income or Mental Health or Nutrition Assessment or Nutrition or Nutritional Status or Nutritional or Physical Activity or Poverty or Preventive Health Service or Preventive Health Services or Promotion of Health or Promotion or Quit Smoking or Quitting Smoking or Second Hand Smoke or Smoke or Smoked or Smoker or Smokers or Smokes or Smoking or Socioeconomic Status or Stop Smoking or Stopping Smoking or Stress Level or Stress Levels or Taught or Teach or Teaching or Tobacco or Weight).ab,ti.
201. 162 or 163 or 164 or 165 or 166 or 167 or 168 or 169 or 170 or 171 or 172 or 173 or 174 or 175 or 176 or 177 or 178 or 179 or 180 or 181 or 182 or 183 or 184 or 185 or 186 or 187 or 188 or 189 or 190 or 191 or 192 or 193 or 194 or 195 or 196 or 197 or 198 or 199 or 200
202. limit 201 to yr="2010 -Current"
203. exp Adolescent/
204. Adolescent.af.
205. exp Child Development/
206. Child Development.af.
207. exp Human Development/
208. Human Development.af.
209. exp Immunization/
210. Immunization.af.

211. exp Maternal Health Services/
212. Maternal Health Services.af.
213. exp Pediatrics/
214. Pediatrics.af.
215. exp Primary Prevention/
216. Primary Prevention.af.
217. exp Secondary Prevention/
218. Secondary Prevention.af.
219. Sexually Transmitted Diseases/
220. Sexually Transmitted Diseases.af.
221. exp Vaccination/
222. Vaccination.af.
223. (Adolescence or Adolescent or Adolescents or Child Development or Childhood or Healthy Development or Human Development or Immunisation or Immunise or Immunised or Immunises or Immunization or Immunize or Immunized or Immunizes or Laboratory Test or Maternal Health Service or Maternal Health Services or Paediatric or Paediatrics or Pediatric or Pediatrics or Pregnancy or Prevent or Prevented or Prevention or Prevents or Primary Prevention or Secondary Prevention or Sexually Transmitted or Vaccinate or Vaccinated or Vaccinates or Vaccination or Vaccine or Vaccines or Youth or Youths).ab,ti.
224. 203 or 204 or 205 or 206 or 207 or 208 or 209 or 210 or 211 or 212 or 213 or 214 or 215 or 216 or 217 or 218 or 219 or 220 or 221 or 222 or 223
225. limit 224 to yr="2010 -Current"
226. Acute Coronary Syndrome/
227. Acute Coronary Syndrome.af.
228. exp Adjustment Disorders/
229. Adjustment Disorders.af.
230. exp Alcohol Drinking/
231. Alcohol Drinking.af.
232. exp Alcohol-Related Disorders/
233. Alcohol-Related Disorders.af.
234. exp Anxiety Disorders/
235. Anxiety Disorders.af.
236. exp Blood Glucose/
237. Blood Glucose.af.
238. exp Hyperglycemia/
239. Hyperglycemia.af.
240. exp Hypoglycemia/
241. Hypoglycemia.af.
242. Cardiovascular Diseases/
243. Cardiovascular Diseases.af.
244. Dementia/
245. Dementia.af.
246. exp Depressive Disorder/
247. Depressive Disorder.af.

248. Diabetes Mellitus/
249. Diabetes Mellitus.af.
250. exp Diabetes, Gestational/
251. Gestational Diabetes.af.
252. Diabetic Foot/
253. Diabetic Foot.af.
254. exp Health Information Interoperability/
255. Health Information Interoperability.af.
256. heart failure/
257. heart failure.af.
258. exp Myocardial Infarction/
259. Myocardial Infarction.af.
260. exp Mental Disorders/
261. Mental Disorders.af.
262. Neoplasms/
263. Neoplasms.af.
264. exp Pain Management/
265. Pain Management.af.
266. Pain/
267. Pain.af.
268. exp Papanicolaou Test/
269. Papanicolaou Test.af.
270. exp Patient Reported Outcome Measures/
271. Patient Reported Outcome Measures.af.
272. exp Physical Examination/
273. Physical Examination.af.
274. exp Pulmonary Disease, Chronic Obstructive/
275. Chronic Obstructive Pulmonary Disease.af.
276. exp Quality Indicators, Health Care/
277. Health Care Quality Indicators.af.
278. exp Rehabilitation/
279. Rehabilitation.af.
280. exp Self Care/
281. Self Care.af.
282. exp Self Efficacy/
283. Self Efficacy.af.
284. Stroke/
285. Stroke.af.
286. (Acs or Acss or Acute Coronary Syndrome or Acute Coronary Syndromes or Adjustment Disorder or Adjustment Disorders or Alcohol Consumption or Alcohol Drinking or Alcohol Related Disorder or Alcohol Related Disorders or Alcoholic or Alcoholism or Anxiety or Anxious or Blood Glucose or Blood Sugar or Blood Sugars or Hyperglycemia or Hypoglycemia or Cancer or Cardiovascular Disease or Cardiovascular Diseases or Cerebrovascular Accident or

Cerebrovascular Accidents or Chf or Chronic Obstructive Pulmonary Disease or Chronic Obstructive Pulmonary Disorder or Chronic Obstructive Pulmonary Disorders or Complication or Complications or Congestive Heart Failure or Copd or Coronary Artery Disease or Coronary Artery Diseases or Coronary Disease or Coronary Diseases or Cva or Cvas or Cvd or Cvds or Dementia or Dementias or Depressed or Depression or Depressive Disorder or Depressive Disorders or Depressive or Diabetes Mellitus or Diabetes or Diabetic Foot or Foot Complication or Foot Complications or Gestational Diabetes or Glycaemia or Glycaemic or Glycemia or Glycemic or Health Care Evaluation Mechanisms or Health Care Quality Indicator or Health Care Quality Indicators or Health Information Interoperability or Healthcare Evaluation Mechanisms or Healthcare Quality Indicator or Healthcare Quality Indicators or Heart Failure or Interoperability or Interoperable or Managed Pain or Management Of Pain or Managing Pain or Mental Disorder or Mental Disorders or Mental Health or Mental Illness or Mental Illnesses or Mentally Healthy or Mentally Ill or Myocardial Infarction or Neoplasm or Neoplasms or Oncologist or Oncologists or Oncology or Pain Management or Pain or Pains or Pap Test or Pap Tests or Papanicolaou Test or Papanicolaou Tests or Patient Reported Experience Measure or Patient Reported Experience Measures or Patient Reported Outcome Measure or Patient Reported Outcome Measures or Physical Exam or Physical Examination or Physical Examinations or Physical Exams or Prem or Prems or Prom or Promotion or Proms or Psycho Social or Psychosocial or Quality Indicator or Quality indicators Of Health Care Quality Indicators or Rehabilitation or Self Care or Self Efficacy or Stroke or Strokes).ab,ti.

287. 226 or 227 or 228 or 229 or 230 or 231 or 232 or 233 or 234 or 235 or 236 or 237 or 238 or 239 or 240 or 241 or 242 or 243 or 244 or 245 or 246 or 247 or 248 or 249 or 250 or 251 or 252 or 253 or 254 or 255 or 256 or 257 or 258 or 259 or 260 or 261 or 262 or 263 or 264 or 265 or 266 or 267 or 268 or 269 or 270 or 271 or 272 or 273 or 274 or 275 or 276 or 277 or 278 or 279 or 280 or 281 or 282 or 283 or 284 or 285 or 286
288. limit 287 to yr="2010 -Current"
289. exp Allied Health Personnel/
290. Allied Health Personnel.af.
291. exp Ambulatory Care Information Systems/
292. Ambulatory Care Information Systems.af.
293. Ambulatory Care/
294. Ambulatory Care.af.
295. exp "Appointments and Schedules"/
296. "Appointments and Schedules".af.
297. exp Clinical Competence/
298. Clinical Competence.af.
299. exp "Duration of Therapy"/
300. "Duration of Therapy".af.
301. exp Patient Education as Topic/
302. Patient Education as Topic.af.
303. exp Evidence-Based Practice/
304. Evidence-Based Practice.af.
305. exp Harm Reduction/
306. Harm Reduction.af.
307. exp Health Behavior/
308. Health Behavior.af.

309. exp Health Care Costs/
310. Health Care Costs.af.
311. exp "Health Care Economics and Organizations"/
312. "Health Care Economics and Organizations".af.
313. exp "Health Care Facilities, Manpower, and Services"/
314. "Health Care Facilities, Manpower, and Services".af.
315. exp "Health Care Quality, Access, and Evaluation"/
316. "Health Care Quality, Access, and Evaluation".af.
317. exp Health Knowledge, Attitudes, Practice/
318. Health Knowledge, Attitudes, Practice.af.
319. exp Health Plan Implementation/
320. Health Plan Implementation.af.
321. exp Health Services Accessibility/
322. Health Services Accessibility.af.
323. exp Healthcare Disparities/
324. Healthcare Disparities.af.
325. exp Hospital Shared Services/
326. Hospital Shared Services.af.
327. exp House Calls/
328. House Calls.af.
329. exp Length of Stay/
330. Length of Stay.af.
331. exp Office Visits/
332. Office Visits.af.
333. exp Patient Acceptance of Health Care/
334. Patient Acceptance of Health Care.af.
335. exp Patient Admission/
336. Patient Admission.af.
337. exp Patient Advocacy/
338. Patient Advocacy.af.
339. exp Prescriptions/
340. Prescriptions.af.
341. exp "Referral and Consultation"/
342. "Referral and Consultation".af.
343. exp Universal Health Care/
344. Universal Health Care.af.
345. (Access To Care or Access To Health Care or Access To Health Services or Access To Healthcare or Accessibility Of Health Services or Allied Health Personnel or Ambulatory Care Information System or Ambulatory Care Information Systems or Ambulatory Care or Appointment or Appointments or Care Access or Clinical Competence or Clinical Competencies or Clinical Competency or Consultation or Consultations or Cost or Costs or Duration Of Therapy or Patient Education or Patient Teaching or Evaluated Health Care or Evaluated Healthcare or Evaluating Health Care or Evaluating Healthcare or Evaluation Of Health Care or Evaluations Of Health Care or Harm Prevention or Harm Reduction or Health Attitude or Health Attitudes or Health Behavior

or Health Behaviors or Health Behaviour or Health Behaviours or HealthCare or Health Care or Health Knowledge or Health Personnel or Health Plan Implementation or Health Service Accessibility or Health Services Accessibility or Healthy Environment or Healthy Environments or Hospital Shared Services or House Call or House Calls or Indirect Cost or indirect Costs or Knowledge Of Health or Length Of Stay or Length Of Therapy or Lengths Of Stay or Los or Office Visit or Office Visits or Patient Acceptance or Patient Admission or Patient Admissions or Patient Advocacy or Prescription or Prescriptions or Prevention Of Harm or Quality Health Care or Quality Healthcare or Reduction Of Harm or Referral or Referrals or Return Visit or Return Visits or Schedule or Scheduled or Schedules or Therapy Duration or Therapy Durations or Therapy Length or Universal Health Care or Universal Healthcare or Unplanned).ab,ti.

346. 289 or 290 or 291 or 292 or 293 or 294 or 295 or 296 or 297 or 298 or 299 or 300 or 301 or 302 or 303 or 304 or 305 or 306 or 307 or 308 or 309 or 310 or 311 or 312 or 313 or 314 or 315 or 316 or 317 or 318 or 319 or 320 or 321 or 322 or 323 or 324 or 325 or 326 or 327 or 328 or 329 or 330 or 331 or 332 or 333 or 334 or 335 or 336 or 337 or 338 or 339 or 340 or 341 or 342 or 343 or 344 or 345
347. limit 346 to yr="2010 -Current"
348. exp Delivery of Health Care/
349. Delivery of Health Care.af.
350. exp Frailty/
351. Frailty.af.
352. exp Global Health/
353. Global Health.af.
354. exp Health Equity/
355. Health Equity.af.
356. exp Health Literacy/
357. Health Literacy.af.
358. exp Health Status Disparities/
359. Health Status Disparities.af.
360. exp Healthcare Disparities/
361. Healthcare Disparities.af.
362. exp Income/
363. Income.af.
364. exp Insurance Coverage/
365. Insurance Coverage.af.
366. exp "International Classification of Functioning, Disability and Health"/
367. "International Classification of Functioning, Disability and Health".af.
368. exp Marital Status/
369. Marital Status.af.
370. exp Minority Health/
371. Minority Health.af.
372. exp "Native Hawaiian or Other Pacific Islander"/
373. Oceanic Ancestry Group.af.
374. exp Patient Selection/
375. Patient Selection.af.

376. exp Population Dynamics/
377. Population Dynamics.af.
378. exp Population Groups/
379. Population Groups.af.
380. exp Poverty Areas/
381. Poverty Areas.af.
382. exp Refusal to Treat/
383. Refusal to Treat.af.
384. Religion/
385. Religion.af.
386. exp Social Discrimination/
387. Social Discrimination.af.
388. exp Socioeconomic Factors/
389. Socioeconomic Factors.af.
390. exp Treatment Failure/
391. Treatment Failure.af.
392. (Aboriginal or Communication Barriers or Delivery Of Health Care or Delivery Of Healthcare or Disability Evaluation or Discrimination or Equity or Frailty or Gender Minorities or Gender Minority or Global Health or Health Care Delivery or Health Care Disparities or Health Care Disparity or Health Equity or Health Literacy or Health Status Disparities or Health Status Disparity or Healthcare Delivery or Healthcare Disparities or Healthcare Disparity or Income or Incomes or Inequal or Inequalities or Inequality or Insurance Cover or Insurance Coverage or Insurance Coverages or Insurance Covered or "International Classification Of Functioning Disability and Health" or Marital Status or Minority Health or Oceanic Ancestry Group or Oceanic Ancestry Groups or Patient Selection or Population Dynamics or Population Group or Population Groups or Poverty Area or Poverty Areas or Poverty or Refusal To Treat or Religion or Religions or Safety Net or Sexual Minorities or Sexual Minority or Sexual Orientation or Sexual Orientations or Social Discrimination or Social Discriminations or Social Support or Treatment Failure or Treatment Failures or Treatment Preference or Treatment Preferences or Vulnerabilities or Vulnerability or Vulnerable).ab,ti.
393. 348 or 349 or 350 or 351 or 352 or 353 or 354 or 355 or 356 or 357 or 358 or 359 or 360 or 361 or 362 or 363 or 364 or 365 or 366 or 367 or 368 or 369 or 370 or 371 or 372 or 373 or 374 or 375 or 376 or 377 or 378 or 379 or 380 or 381 or 382 or 383 or 384 or 385 or 386 or 387 or 388 or 389 or 390 or 391 or 392
394. limit 393 to yr="2010 -Current"
395. ((Systematic* adj3 (Review* or Overview*)) or (Methodologic* adj3 (Review* or Overview*))).af.
396. Systematic review.pt.
397. meta-analysis/ or systematic review/ or meta-analysis as topic/ or "meta analysis (topic)"/ or "systematic review (topic)"/
398. exp meta-analysis/
399. Meta Analy*.tw.
400. Metanaly*.tw.
401. Metaanaly*.tw.
402. Met Analy*.tw.

403. ((overview$ or review or synthesis or summary or cochrane or analysis) and (reviews or meta-analyses or articles)).ti.
404. (meta-review or metareview).ti,ab.
405. ((overview$ or reviews) and (systematic or cochrane)).ti.
406. (reviews adj2 (meta or published or quality or included or summar$)).ab.
407. cochrane reviews.ab.
408. (evidence and (reviews or meta-analyses)).ti.
409. 160 or 201 or 224 or 287 or 346 or 393
410. 395 or 396 or 397 or 398 or 399 or 400 or 401 or 402 or 403 or 404 or 405 or 406 or 407 or 408
411. 90 and 99 and 409 and 410
412. limit 411 to yr="2010 -Current"
