# Supplementary material for: Identifying indicators sensitive to primary healthcare nurse practitioner practice: A review of systematic reviews
Source: PLoS One. 2023 Sep 7;18(9):e0290977. doi: 10.1371/journal.pone.0290977 (PMC10484467; doi:10.1371/journal.pone.0290977)
Supplement: S2 Appendix — (PDF) [file pone.0290977.s003.pdf]

S2 Appendix. Search strategies for the grey literature

Sample grey literature search terms:
indicator AND (primary care) AND (nurse practitioner)

CADTH Information Services, Grey Matters: a practical tool for searching health-related grey literature
https://www.cadth.ca/resources/finding-evidence/grey-matters

Organization for Economic Co-operation and Development (OECD)
http://www.oecd.org/

ProQuest Dissertation and Theses
https://about.proquest.com/products-services/pqdtglobal.html

World Health Organization
https://www.who.int/

Health Evidence
https://www.healthevidence.org/search-tips.aspx

Health Systems Evidence
https://www.healthsystemsevidence.org/

PDQ-Evidence
https://www.pdq-evidence.org/

Prospero International Prospective Register of Systematic Reviews
https://www.crd.york.ac.uk/prospero/

00 - GENERAL, MULTIDISCIPLINARY

East View Information Services
http://www.eastview.com

GreyNet International, Grey literature Network Service
http://www.greynet.org

Grijze Literatuur in Nederland, GLIN
http://www.publiekwijzer.nl/bestanden.php?id=zoeknaar&db=3.2

Italian Grey Literature Database
http://polarcnr.area.ge.cnr.it/cataloghi/bice/index.php?type=Grigia

National Repository of Grey Literature, NRGL
http://www.nusl.cz/?lang=en

OpenGrey Repository, System for Information on Grey Literature in Europe
http://www.opengrey.eu

NEW ENTRY

06 - BIOLOGICAL & MEDICAL SCIENCES

Cochrane Reviews
http://www.cochrane.org/reviews/en/mr000010.html

Doctor of Nursing Practice, DNP
http://libguides.rutgers.edu/content.php?pid=449135&sid=3680427

Duke University Medical Center Library
http://guides.mclibrary.duke.edu/greyliterature

Gray Literature in Health Research
http://researchguides.dml.georgetown.edu/content.php?pid=352972&sid=2887419

Grey Horizon, A Grey Literature Current Awareness Tool in Cancer Care
http://grey-horizon.blogspot.nl/

Grey Literature for Dentistry
http://guides.library.utoronto.ca/dentistry

Grey Literature in the Health Sciences
http://guides.library.upenn.edu/healthgreylit?hs=a

Grey Literature Report - New York Academy of Medicine
http://www.greylit.org

HealthKnowledge
http://www.healthknowledge.org.uk/public-health-textbook/research-methods/1a-epidemiology/grey-literature

Lister Hill Library of the Health Sciences
http://libguides.lhl.uab.edu/GreyLit

Mesothelioma Guide
https://www.mesotheliomaguide.com/mesothelioma/causes/asbestos-and-cancer/

Mesothelioma.net
http://www.mesothelioma.net

Norris Medical Library - Grey Literature Resource Guide
http://norris.usc.libguides.com/grey_literature

Nursing and Allied Health Resource Section, NAHRS
http://sites.google.com/site/nahrsnursingresources/Home/grey-literature-1

Social Policy and Practice
http://bathhealthnews.blogspot.com/2009/11/new-database-social-policy-practice.html

Health Sciences Library and Informatics Center
http://libguides.health.unm.edu/content.php?pid=200149

University of Waterloo
http://www.lib.uwaterloo.ca/discipline/health_kin/grey_literature.html
