# Supplementary material for: Identifying indicators sensitive to primary healthcare nurse practitioner practice: A review of systematic reviews
Source: PLoS One. 2023 Sep 7;18(9):e0290977. doi: 10.1371/journal.pone.0290977 (PMC10484467; doi:10.1371/journal.pone.0290977)
Supplement: S3 Appendix — (PDF) [file pone.0290977.s004.pdf]

S3 Appendix. Record of review-related decisions.

Excluded for wrong design:

1. Koch S, Fleischer S. [Potential analysis for research on Advanced Practice Nursing (APN) for persons with dementia living in long-term care facilities]. Zeitschrift fur Evidenz Fortbildung und Qualitat im Gesundheitswesen. 2014;108:S-S19.
2. Barnett M, Balkissoon C, Sandhu J. The level of quality care nurse practitioners provide compared with their physician colleagues in the primary care setting: A systematic review. J Am Assoc Nurse Pract. 2022;34:457-64. doi:10.1097/jxx.0000000000000660.
3. Nikpour J, Franklin M, Calhoun N, Broome M. Influence of provider type on chronic pain prescribing patterns A systematic review. J Am Assoc Nurse Pract. 2022;34:474-88. doi:10.1097/JXX.0000000000000673.

Excluded for wrong role:

4. Crowe M, Jones V, Stone MA, Coe G. The clinical effectiveness of nursing models of diabetes care: A synthesis of the evidence. Int J Nurs Stud. 2019;93:119-28. doi: 10.1016/j.ijnurstu.2019.03.004. PubMed PMID: 30908959.
5. Abraham CM, Norful AA, Stone PW, Poghosyan L. Cost-effectiveness of advanced practice nurses compared to physician-led care for chronic diseases: A systematic review. Nursing Economics. 2019;37(6):293-305. PubMed PMID: 34616101; PubMed Central PMCID: PMCPMC8491992.
6. Chen CC, Chen Y, Liu X, Wen Y, Ma DY, Huang YY, et al. The Efficacy of a Nurse-Led Disease Management Program in Improving the Quality of Life for Patients with Chronic Kidney Disease: A Meta-Analysis. PloS one. 2016;11(5):e0155890. doi: 10.1371/journal.pone.0155890. PubMed PMID: 27191392; PubMed Central PMCID: PMCPMC4871412.
7. Bohner K, Zeller H, Saxer S. Effectiveness and efficiency of advanced practice nurses in patients with chronic heart failure. Pflegewissenschaft. 2012;14(6):342-8.
8. Hyde R, MacVicar S, Humphrey T. Advanced practice for children and young people: A systematic review with narrative summary. Journal of Advanced Nursing. 2020;76(1):135-46. doi: 10.1111/jan.14243. PubMed PMID: 31642083.
9. McCrory G, Patton D, Moore Z, O'Connor T, Nugent L. The impact of advanced nurse practitioners on patient outcomes in chronic kidney disease: A systematic review. Journal of Renal Care. 2018;44(4):197-209. doi: 10.1111/jorc.12245. PubMed PMID: 29888444
10. Fry MM. A systematic review of the impact of afterhours care models on emergency departments, ambulance and general practice services. Australasian Emergency Nursing Journal. 2011;14(4):217-25.
11. Kobleder A, Mayer H, Gehrig L, Ott S, Senn B. Wirksamkeit von APN-Interventionen in der gynäkologischen Onkologie. Eine systematische Literaturübersicht. Klinische Pflegeforschung. 2017;3:85-101. doi: 10.6094/KlinPfleg.3.85.
12. Cross AJ, Liang J, Thomas D, Zairina E, Abramson MJ, George J. Educational interventions for health professionals managing chronic obstructive pulmonary disease in primary care (Review). Cochrane Database Syst Rev. 2022;5(5):CD012652. doi:10.1002/14651858.CD012652.pub2.

13. Fichadiya P, Then KL, Rankin JA. The impact of nurse practitioners on health outcomes in outpatient heart failure management: A systematic review. Can J Cardiovasc Nurs. 2021;31(2):20-7.
14. van Erp RMA, van Doorn AL, van den Brink GT, Peters JWB, Laurant MGH, van Vught AJ. Physician assistants and nurse practitioners in primary care plus: A systematic review. Int J Integr Care. 2021;21(1):6. doi:10.5334/ijic.5485.
15. Veenema TG, Lavin RP, Thornton CP, Schneider-Firestone S, Seal S. Alignment of nurse practitioner educational preparation and scope of practice in United States emergency departments: A systematic review of the literature. J Emerg Nurs. 2021;47(4):563-81. doi:10.1016/j.jen.2021.04.005.
16. Watkins AK, Clark AP, Champion JD. Telemedicine practices in adult patients with atrial fibrillation. J Am Assoc Nurse Pract. 2022;34(8):957-62. doi:10.1097/jxx.0000000000000743.
17. Soster CB, Anschau F, Rodrigues NH, da Silva LGA, Klafke A. Advanced triage protocols in the emergency department: A systematic review and meta-analysis. Rev Latino-Am Enfermagem. 2022;30:e3511. doi:10.1590/1518-8345.5479.3511.
18. Stefan MS, Knee AB, Ready A, Rastegar V, Seaman JB, Gunn B, et al. Efficacy of models of palliative care delivered beyond the traditional physician-led, subspecialty consultation service model: A systematic review and meta-analysis. BMJ Support Palliat Care. 2022; Online First: 19 April 2022. doi: 10.1136/bmjspcare-2021-003507.

Excluded for wrong/no outcomes:

19. Chan SS, Cheung NK, Graham CA, Rainer TH. Strategies and solutions to alleviate access block and overcrowding in emergency departments. Hong Kong Medical Journal. 2015;21(4):345-52. doi: 10.12809/hkmj144399. PubMed PMID: 26087756.
20. Tabesh M, Magliano DJ, Koye DN, Shaw JE. The effect of nurse prescribers on glycaemic control in type 2 diabetes: A systematic review and meta-analysis. Int J Nurs Stud. 2018;78:37-43. doi: 10.1016/j.ijnurstu.2017.08.018. PubMed PMID: 28939342
