# Supplementary material for: Identifying indicators sensitive to primary healthcare nurse practitioner practice: A review of systematic reviews
Source: PLoS One. 2023 Sep 7;18(9):e0290977. doi: 10.1371/journal.pone.0290977 (PMC10484467; doi:10.1371/journal.pone.0290977)
Supplement: S1 Table — (PDF) [file pone.0290977.s005.pdf]

S1 Table. Extraction of review results by indicator category.

Level of indicator	Indicator category	Number of studies	Author (Year)	Ref	Results
**Patient**					
	**Activities of daily living (ADL)**	7	Donald (2015)	[27]	**Functional independence:** Estimates favoured nurse practitioner care
			Lovink (2017)	[32]	**Activities of daily living in LTC :** significant improvement in 1/1 study (p = 0.04)
			Lovink (2017)	[32]	**Health status and functional ability** 14 other outcomes, no significant effects were found in 1/1 study in LTC
			Morilla-Herrera (2016)	[34]	**Functional status (daily living)**<br>2/5 studies found significantly better functional status based on the independence to develop IADL [OR (95%CI):0.6(0.3–1.0); P = 0.04]; [OR(95%CI):3.0 (0.6–5.4); P: 0.02]and combined BADL and IADL[OR (95% CI):2.9(4.0–5.4); P: 0.03]; 2/5 found no differences and 1/5 found a trend in improvement (no p-value reported)
			Newhouse/ Stanik-Hutt (2013)	[35/36]	**Functional status.** Eight studies (five RCTs); 2/8 studies reported findings favouring the NP group and 6/8 reported no differences between groups (no p-values reported).
			Osakwe (2020)	[37]	**Dependency in basic activity of daily living**: The odds were significantly lower in the intervention group compared to the control group (adjusted odds ratio, 0.4; 95% CI, 0.2 – 0.8; p = 0.02). Nine people in the intervention group (4%) and 20 people in the control group (10%) were permanently admitted to nursing homes (P = 0.02). (1/1 study)
			McParland (2022)	[52]	**Physical Functioning (1 study)**<br>Trend towards improved function in the intervention group across the three time points while control declined (no p value reported)
			Sun (2022)	[53]	**School attendance of children with special needs (1 study)**<br>Providing NP home visits to children with special needs significantly improved school

					and work attendance for children and parents. Parents missed less work (pre vs post 26.3% s 14.1%, p <.05); missed schools >20 days (pre vs post 10.4% vs 11.7%, p >.05).
	**Adaptation to health conditions**	5	Donald (2013)	[54]	**Adaptation-related goals (1 study)**<br>Differed significantly for the nursing home subgroup in favour of the NP group, (P < 0.05)
			Donald (2013)	[54]	**Overall mean goal attainment** scores did not differ between groups (1/1)
			Donald (2013)	[54]	**Advance directive measures**: no significant differences between the groups (1/1)
			Fung (2014)	[55]	**Disempowerment in treatment**: significant reduction (p< 0.001).
			Fung (2014)	[55]	**Disempowerment in the early** (M1 = 13.073, SD = 7.73) **and later in treatment** (M2 = 9.14, SD = 6.04): significant reduction (p value not reported).
			Fung (2014)	[55]	**Life review therapy** conducted by psychiatric APNs could lead to a decrease in negative themes and might be an effective therapy for the homebound elderly with depression. A significant decline in depression among the treatment group was noted as measured by both the Hamilton Depression Scale (decrease of 6.3, 95% confidence interval (CI) = 4.2–8.3] and the Geriatric Depression Scale (a decline of 4.4, 95% CI = 2.5–6.4).
			Fung (2014)	[55]	**Uncertainty in the ambiguity:** When comparing the depressed women with cancer in high distress (n=32) with those in the placebo group, significantly less subscale of the Mishel Uncertainty in Illness Scale (P = 0.0181) (1/1)
			Fung (2014)	[55]	**Distress:** When comparing depressed women with cancer in high distress with those in the placebo group, less symptom in the Symptom Distress Scale (P < 0.0001) (1/1)
			Fung (2014)	[55]	**Distress in adolescents** at baseline and at 6, 12, 18, 24, 30, and 36 months post-intervention. At the first two time points (N = 1030), a significant effect was seen (P = 0.0001). Taking the first four time points into consideration (N = 810; 21% attrition), a significant intervention effect was noted from the SCSI (P = 0.006). When all six time points were used (N = 669; 17% attrition), a marginally significant intervention effect was seen (P = 0.06). Adolescents experienced less mental distress in the first 2 years, but this difference dissipated by 30 and 36 months.

			Garner	[56]	**Arthritis self-efficacy and empowerment:** 2 studies. In the AMBRA study, nurse-led care was found to be superior to rheumatologist-led care regarding self-efficacy at 12 months, but this was not statistically significant at 24 months (1/1). Arvidsson, et al's phenomenographic study noted that nurse-led care patients were empowered to solve their own problems (1/1).
			Morilla-Herrera (2016)	[34]	**Care planning**<br>1/1 study showed higher perceived quality of care planning (p = 0.005)
			Morilla-Herrera (2016)	[34]	**Caregiver Distress**<br>½ studies found positive results for mental health [IG: 19(13.48)vs.CG(28 (21.54); P: 0.05]; ½ studies showed significant improvements in distress as measured by the caregiver NPI at 12 months (no p-values reported)
			McParland (2022)	[52]	**Caregiver Burden (3 studies)**<br>No significant differences in caregiver strain or burden in 3/3 studies
	**Clinical Health Outcomes**	8	Milesky (2020)	[47]	NPs improved health outcomes in 19.85% of theme occurences
			Newhouse/ Stanik-Hutt (2013)	[35/36]	**Self-reported perceived health**. 5 studies: 1/5studies reported findings in favour of the NP group; 4/5 studies reported no differences between groups (no p-values reported).<br><br>Full details if need to revisit authors' finding: Counsell (phys SF36 no diff, mental SF36 favours NP), Lenz 2002 (SF36 mental and physical no diff), Litaker 2003 (SF12 physical and mental no diff), Mundinger (SF physical and mental no diff), Ahern (SF36 physical and mental no diff)
			Smigorowsky (2020)	[57]	**Effect of NP-led care on SF 36 physical composite score (2 studies)**<br>Mean difference ([MD] = 0.17, 95% CI: −0.89, 1.23; Z = 0.32), p = .75, inference no significant difference. I2 = 0% indicating low risk for heterogeneity

			Swan (2015)	[12]	**Physiologic measures (3 studies)**<br>Between-group differences were generally not significant, with the exception of the cholesterol/high-density lipoprotein (HDL) ratio and the diastolic blood pressure at 6 months with both favoring the APN group. Additional physiologic measures were investigated in single studies with no differences between APN and physician groups for mortality, change in body mass index (BMI), change in LDL or peak expiratory flow rate.
			Tsiachristas (2015)	[41]	**Clinical outcomes (5 studies)**<br>5/5 studies reported improvements in clinical outcomes
			Van Vliet (2020)	[42]	**Clinical outcomes**<br>Little is know about the effects of NPs in ambulance care on patient outcomes or care provider outcomes
			Yang (2020)	[58]	**Health outcomes**: 5/5 studies reported positive health outcomes and significant improvements in health status
			Sun (2022)	[53]	**Health Risk reduction for community dwelling adults with intellectual and developmental disability (1 study)**<br>Health risk reduction (pre vs post 4.7 vs 3.5, p <.05)
	**Clinical-Cardiovascu lar Outcomes**	10	Carranza (2021)	[59]	**Metabolic outcomes:** Overall improvement in metabolic outcomes, but no p-values reported.<br>NP vs. MD, within-group decrease (between-group difference):<br>-HA1C: 2.5% vs 0.2% (2.3%)<br>-BP: No difference within and between groups<br>-Weight loss: 8.3 lbs. vs. 7.4 lbs. (0.9 lbs.)<br>-Glucose: 83.7 mg/dl vs. 27.4 mg/dl (56.3 mg/dl)
			HQO (2013)	[60]	**Blood pressure:**<br>Model 1: 139 mmHg vs 139 mm Hg (p= 0.82) (1/1)<br>Model 2: effect not assessed (4/4)
			HQO (2013)	[60]	**Clinical examination:**<br>Model 2: Patients in the specialized nursing group received significantly more measures of blood pressure (P < 0.001) among CAD patients (3/3).

			HQO (2013)	[60]	**Clinical examination:**<br>Model 2: Among CAD patients (3/3), no significant difference between groups in the **proportion of individuals with cholesterol measured** (P = 0.48).
			Lovink (2017)	[32]	**Composite score for heart failure (1 study)**<br>intervention improved the composite endpoint of heart failure patients from -37 to 25 (P = 0.01). (Agvall, 2013, 2014)
			Lovink (2017)	[32]	**Ejection Fraction (EF) (1 study)**<br>difference in favour of the intervention: 33 patients compared with 45 in the control group (P = 0.03) had an EF <40%. (Agvall, 2013, 2014) (1/1)
			Lovink (2017)	[32]	**N-terminal pro brain natriuretic peptide (NTproBNP) levels (1 study)**<br>change in NT-proBNP level before and after the intervention was significant in the intervention group; it decreased from 1091 to 895 ng/L (P = 0.01). There was no significant before/after difference in the control group [588 vs. 671 ng/L (P = 0.5)] (Agvall, 2013, 2014).
			Martin-Misener (2015)	[11]	**Hypertension (1 study)**<br>the drop in diastolic blood pressure at 6 months was larger in the nurse practitioner group (356 patients) (mean difference: −3.0 mm Hg (95% CI −5.54 to −0.46); p=0.04) (LQE).
			Martinez-Gonzalez (2014)	[33]	**Meta-analysis: Blood Pressure (5 RCTs)**<br>Compared to physician-led care, the pooled weighted mean differences (WMD) revealed a significant SBP-reducing effect of nurse led care interventions (SBP, mmHg: WMD -4.27, 95%CI -6.31 to -2.23; p <0.0001).<br>The pooled weighted mean differences (WMD) also favoured a DBP-reducing effect of nurse-led care interventions but the confidence intervals crossed the line of no effect (DBP, mmHg: WMD -1.48, 95%CI -3.05 to -0.09; p = 0.06).<br>There was no significant heterogeneity between trials (SBP: I2= 0%, p= 0.53; DBP: I2 = 38%, p = 0.19).

			Martinez-Gonzalez (2014)	[33]	**Meta-analysis**<br>**Nurse-led care and physician-led care in reducing the mean levels of total cholesterol (TC) at follow up** (4 studies)<br>No significant heterogeneity between trials (TC, mmol/l: weighted mean differences (WMD -0.08, 95%CI -0.22 to 0.07, p = 0.29; I2 = 0%).<br>Individual trial estimates showed significantly more patients with nurse-led care had a positive decrease or regression in TC and low density lipoprotein (LDL) levels than did patients in the group of physicians [22].<br>3/5 trial estimates showed no significant differences between groups in the reduction of LDL, high density lipoprotein (HDL), TC/HDL ratio or triglycerides.
			Martinez-Gonzalez (2014)	[33]	**Meta-analysis**<br>**Cardiac function (1 study)**<br>Compared to physician-led care, there were significantly more patients with nurse-led care who had a decrease or regression in the levels of functional exercise capacity (p = 0.001), N-terminal pro-brain natriuretic peptide (p= 0.004) or in the left ventricular end-diastolic volume index (p = 0.040).<br><br>There were no significant differences between groups in the levels of C-reactive protein, left atrial size index, and left ventricular mass index or in the ratio of early to late mitral valve flow velocity.
			Newhouse/ Stanik-Hutt (2013)	[35/36]	**Lipid control**. Three studies; 3/3 studies reported findings favouring the NP group (no p-values reported).<br><br>Full details if need to revisit authors' finding as written above à 3 studies reporting diff lipids, but at least 1 fav NP in each study: Becker (LDL-C fav. NP; no diff HDL-C et TG), Litaker (no diff TC, HDL fav NP), Paez (Chol + LDL-C both fav NP)
			Newhouse/ Stanik-Hutt (2013)	[35/36]	**Blood pressure**. (4 studies); 2/4 reported findings on blood pressure control favouring the NP group and 2/4 no difference between groups (no p-values reported)<br>Full details if need to revisit authors' finding as written above à 4 studies reporting diff BP measures, with 2/4 with at least 1 fav NP: Becker (SBP + DBP both fav NP), Lenz 2004 (SBP + DBP both no diff), Litaker (BP ctrl no diff), Mundinger (SBP no diff, DBP fav. NP)
			Norful (2019)	[61]	**Hyperlipidemia**: sig more in the co-management group (p = .007)
			Norful (2019)	[61]	**Blood pressure monitoring**: no sig differences (p = .63).

			Norful (2019)	[61]	**HDL**: sig findings in 1/1 (p = 0.02)
			Schadewaldt (2011)	[39]	**Blood pressure** 1 study<br>Blood pressure reduction is possible for the short term (3 months). Effect not maintained 12 weeks after intense nurse-led support stopped. Random effect meta-analysis not sig at 6 to 8 months from the patient's attendance to a nurse-led clinic (P = 0.26 systolic blood pressure and P = 0.25 diastolic blood pressure. Significant heterogeneity (P = 0.002, I2 = 90% for systolic blood pressure and P = 0.00001, I2 = 96% for diastolic blood pressure).
			Schadewaldt (2011)	[39]	**Long-term blood pressure outcomes (up to 1 year)** (4 studies)<br>Three studies found significant improvements in blood pressure less than 140/85 mmHg after 1 year participating in a disease management program (P < 0.001). and target 160/95, P < 0.001).
			Schadewaldt (2011)	[39]	**Blood lipid outcomes**<br>Reduction but no sig difference between groups for total cholesterol (TC) and high-density lipoprotein (HDL) at 6, 8, 12 and 24 months.<br>When examining the target value of less than 5 mmol/L: patients benefited from the nurse-led clinics after 1 year (P < 0.00001) 2/2 studies<br>**HDL**: no sig difference in the meta-analysis in 2/2 studies at 3, 6, 12, and 24 months<br>**LDL-C and Triglyceride**: sig results at 3 and 6 months in 2/3 studies (p = 0.001, p = 0.008) and no sig results at 12 and 18 months in 3/3 studies
			Scott (2011)	[62]	**Estimated incidence rates of previously undetected hypertension** ranged from 14.3% to 63.1% no sig. specified. (4 studies)
			Smigorowsky (2020)	[57]	**Effect of NP-led care on vascular risk reduction (1 study)**<br>The NP-led group had a higher Framingham risk score at baseline. Therefore, the baseline Framingham risk score was adjusted, to produce a relative change of −12% (−22% to −3%) versus usual care −8% (−18% to 2%). That is, patients in the NP-led group had a 12% decrease in risk of developing coronary heart disease over the next 10 years. Secondary endpoints were absolute changes in the levels of risk factors. In the NP-led group 18.4% of patients reached low-density lipoprotein cholesterol (LDL) targets and 19% stopped smoking.
	**Clinical-Respiratory Outcomes**	6	Donald (2015)	[27]	**Experience of asthma exacerbation**: (relative risk (RR): 0.93, 95%CI: 0.65–1.33, p = 0.67)

			Donald (2015)	[27]	**Change in maximal peak flow in asthma** (mean difference (MD): 1.39, 95%CI: 6.63 to 3.85, p = 0.12),
			Donald (2015)	[27]	**Emergency nebulisations** (RR: 1.60, 95%CI: 0.79–3.24, p = 0.19),
			HQO (2013)	[60]	**Disease specific measure: peak flow**<br>Model 1: no difference (p = 0.82) (1/1)<br>Model 2: (no data)
			Kuethe (2013)	[29]	**Frequency of exacerbations**<br>No sig difference between the groups in 4/4 studies (Nathan, Kamps, 2003; Kuethe, 2011; Van Son, 2004)<br>rate ratio 1.23; 95% CI 0.91 to 1.66; P =0.368) (Nathan, 2006)<br>No sig difference at 6-month follow up (OR 0.86; 95% CI 0.44 to 1.71;p = 0.674). (Nathan)
			Kuethe (2013)	[29]	**Asthma severity**<br>No sig difference in asthma control between the nurse and generalist (P = 0.18) and specialist physicians P = 0.28)<br>Non-inferiority for the asthma control in the nurse-led group
			Kuethe (2013)	[29]	**Asthma symptoms**<br>No sig difference in asthma control between the nurse and generalist (P = 0.18) and specialist physicians P = 0.28)<br>Non-inferiority for the asthma control in the nurse-led group
			Kuethe (2013)	[29]	**Absence from school/work due to asthma (3 studies)**<br>Median of 0 days school absence in nurse group for Kamps; Kuethe; Pilotto . In Pilotto et al.with 20.6% of patients (in an adult population) in the physician group had more than one day of absence in the physician group (p = 0,04)
			Kuethe (2013)	[29]	**Forced expiratory volume in 1 second (FEV1)** (3 studies)<br>(Kamps 2003; Kuethe 2011; Pilotto 2004). No difference found (MD -0.54% predicted; 95% CI -4.20 to 3.12). Sensitivity analysis using a fixed-effect model showed the same results (MD -0.54% predicted; 95% CI -4.20 to 3.12).

			Kuethe (2013)	[29]	**Peak expiratory flow rate (PEF)** (2 studies, Nathan; van son<br>Nathan found a decrease of PEF over time. Mean drop was 2.53% (SD 11.5) in the physician-led group and 3.92% (SD 12.4) in the nurse-led group,<br>No sig difference in the change in PEF between the two groups (P = 0.122)<br><br>Van Son showed an increase over time (5.3% in the nurse-led group and 3.94% in the physician-led group), but no statistical significant between group differences were found (P = 0.66).
			Kuethe (2013)	[29]	**Airway hyper-reactivity (including PD/PC20 methacholine/histamine)** (2 studies, Kuethe and Kamps)<br>No statistically sig differences between groups
			Martinez-Gonzalez (2014)	[33]	**Meta-analysis**<br>**Lung Function (3 studies)**<br>Individual trial estimates showed no significant differences between groups in various parameters of lung function including measurements of peak flow at six months, and PD20, lung function (%FEV1) or FENO either at 12 or 24 months.
			Newhouse/ Stanik-Hutt (2013)	[35/36]	**Peak flow** (1 study). No difference between groups, no p-value reported.
			Sun (2022)	[53]	**Asthma control in children (1 study)**<br>Children with asthma had fewer nighttime symptoms (t test 3.966 df 19 p <.05), fewer course of oral steroids (t test 3.750 df 18, p <.05), better adherence to therapy (z score -3.272, p <.05), level of control (z score -4.132, p <.001).
			Sun (2022)	[53]	**School attendance, asthma control in children (1 study)**<br>A trend towards 4 fewer missed school days was experienced but not significant (t test 1.928 df 15 p >.05)).
	**Clinical-Renal Outcomes**	2	HQO (2013)	[60]	**Process indicator: urinalysis**<br>Model 1: (p < 0.01)<br>Model 2: (no data)

			Martinez-Gonzalez (2014)	[33]	**Meta-analysis**<br>**Kidney Function (3 studies)**<br>No significant differences between groups in the parameters of kidney function including the levels of urine sodium excretion and serum creatinine at six months. The reported median (IQR) levels of urinary albumin excretion tested to detect renal complications were higher in the nurse-led care group [UAER, mmol/day: nurse-led care, median 39.2 (IQR 16.0 to 200.0) vs. physician-led care, median 30.5 (IQR 14.5 to 147.2)].
	**Clinical-Cancer Care**	2	Loescher (2018)	[31]	**Clinical examination**<br>Six studies had some description of NPs' performance of clinical skin examination (Ali et al., 2014; Blake & Malone, 2014; Bradley, 2012; DeKoninck & Christenbery, 2015; Roebuck et al., 2015; Shelby, 2014).<br>In one study, NP-led skin cancer clinic over a 4-year period provided approximately a 14% increase in clinical skin examination. Over the four years, the authors found that the number of patients reviewed by the dermatologist dropped from 53% to 31%, and the number of patients requiring biopsy or surgery decreased from 22% to 6.5%.
			Smith (2014)	[49]	**Post-treatment survivorship care**<br>No study of post-treatment survivorship care identified.
	**Clinical-Diabetes**	7	Carranza (2021)	[59]	**Metabolic outcomes:** Overall improvement in metabolic outcomes, but no p-values reported.<br>NP vs. MD, within-group decrease (between-group difference):<br>-HA1C: 2.5% vs 0.2% (2.3%)<br>-BP: No difference within and between groups<br>-Weight loss: 8.3 lbs. vs. 7.4 lbs. (0.9 lbs.)<br>-Glucose: 83.7 mg/dl vs. 27.4 mg/dl (56.3 mg/dl)
			Carranza (2021)	[59]	**Metabolic outcomes:** Overall improvement in metabolic outcomes, but no p-values reported.<br>NP vs. MD, within-group decrease (between-group difference):<br>-HA1C: 2.5% vs 0.2% (2.3%)<br>-BP: No difference within and between groups<br>-Weight loss: 8.3 lbs. vs. 7.4 lbs. (0.9 lbs.)<br>-Glucose: 83.7 mg/dl vs. 27.4 mg/dl (56.3 mg/dl)

			HQO (2013)	[60]	**HbA1C**<br>Model 1: mean 9.72% intervention vs 9.84%: no difference (p = 0.82) (1/1)<br>Model 2: significant decrease after 12 months favouring specialized nurse-MD team (p value not reported) (1/2); non-sig difference in HbA1C (MD, -0.12; 95% CI -0.44 to 0.20) (2/2)
			HQO (2013)	[60]	**Process indicator: HbA1C**<br>Model 1: (p < 0.05)<br>Model 2: (no data)
			HQO (2013)	[60]	**Model 1: Process indicator**: no sig difference in patient history, weight, blood pressure, foot health, blood glucose level, creatinine level, and referrals to ophthalmologist.<br>Model 2: (no data)
			HQO (2013)	[60]	**Clinical examination:**<br>Model 2: Patients in the specialized nursing group received significantly more foot exams (p<0.05) among CAD patients (3/3).
			HQO (2013)	[60]	**Clinical examination:**<br>Model 2: Patients in the specialized nursing group received significantly more retinal exam > 24 months (p = 0.01) among CAD patients (3/3).
			Lovink (2017)	[32]	**Mean HbA1c** of patients with diabetes: no significant difference **in 1/1 study**
			Lovink (2017)	[32]	**Two or more HbA1C tests**: no significant differences in 1/1 study
			Martinez-Gonzalez (2014)	[33]	**Meta-analysis**<br>**Glycosylated Haemoglobin Concentration (4 studies)**<br>No significant differences between nurse-led care and physician-led care in reducing glycosylated haemoglobin concentrations (HbA1c) at follow up.<br>No significant heterogeneity between trials (HbA1c, %: weighted mean difference (WMD) 0.12, 95%CI -0.13 to 0.37, p =0.33; I2 = 0%).<br>One trial/4 estimates showed no significant differences in the number of patients with a positive decrease or regression in blood glucose levels.
			Newhouse/ Stanik-Hutt (2013)	[35/36]	**Blood glucose control**: 4 studies; ¼ studies reported findings favouring the NP group; ¾ reported no difference between groups (no p-values reported)

			Norful (2019)	[61]	**Annual eye exams:** no sig difference in annual exams by ophthalmologists
			Norful (2019)	[61]	**Diabetic control**: sig more in the co-management group (p < .001)
			Norful (2019)	[61]	**HbA1C** : 1/2 sig findings (p=0.02); ½ non sig findings
			Sun (2022)	[53]	**HbA1C (1 study)**<br>Greater decrease in A1C at 3 months (intervention vs control 1.7% vs. 0.7%, p <.001), 6 months (1.7% vs 0.8%, p <.001) in 1/1 study.
	**Clinical-Rheumatoid Arthritis**	2	Carranza (2021)	[59]	**Disease progression**: 1/1 no sig difference between NP and physician groups
			Carranza (2021)	[59]	**Disease severity**: 1/1 no sig difference between NP and physician groups
			Carranza (2021)	[59]	**Disease progression:** Sig reduced with NP group (no p-values reported) (1/1)
			Garner	[56]	**Disease activity** in rheumatoid arthritis assessed in 5/5 studies. Results include equal to superior care. Significant reduction (p < 0.05).
			Garner	[56]	**Morning stiffness** assessed in 2 studies with significant increase in ½ studies, rheumatologist-led care change 6.67 vs nurse-Led care –5.98, p = 0.01)
	**Clinical-Mental Health**	8	Donald (2015)	[27]	**Anxiety (STAI) Rehabilitation** (mean difference: -15.7(95%CI: -20.73 to -10.67) <0.0001 (low quality)
			Fung (2014)	[55]	**Depression symptoms:** Reduced in primary care and home care (3/4)
			Fung (2014)	[55]	**Psychological stress:** Significant reduction for individuals (women, students, HIV) 5/5
			Fung (2014)	[55]	**Efficacy self-esteem with women with depression:** Increase in the mean efficacy self-esteem score post-intervention and a significant difference between pre- and post-intervention BDI scores (t = 8.765, d.f. = 29, P = 0.0005) in a paired samples t-test. (1/1)
			Fung (2014)	[55]	**Uncertainty for depressed women with cancer including those in high distress:** significantly less than those in the placebo group at 6 months after surgery. (1/1)
			Morilla-Herrera (2016)	[34]	**Depression**<br>1/1 study showed no significant group differences in depression

			Schadewaldt (2011)	[39]	**Depression and anxiety**<br>No sig differences in 1/1 study at 1- and 4-years. Suspected cross-over effect for patients in the control group at 4 years with decreased odds for depression for clinic attendees (P = 0.001).
			Smigorowsky (2020)	[57]	**Effect of NP-led care on SF 36 mental composite score (2 studies)**<br>Mean difference for SF36 mental composite score (mean difference [MD] = −1.11, 95% CI: −4.19, 1.98; Z = 0.70, p = .48; suggests no statistical difference. I2 Statistic is 80%, therefore, there is a high risk of heterogeneity. These results must be interpreted with care because there is considerable variation in the combined or pooled results
			Swan (2015)	[12]	**Subjective health status** (e.g., SF-36, burden of illness): with no differences between groups
			McParland (2022)	[52]	**Mental health (2 studies)**<br>Reductions in cognitive impairment [Pfeiffer Score] over 12 months with telephone follow-up in 1/1 study (p value not reported)<br>Reductions in severe depression at 12 months [Geriatric Depression Scale] and loneliness [UCLA Loneliness Scale] in 1/1 study<br>Significant improvement in cognitive impairment [Orientation Memory Cognition Tool] in 1/1 study
			Turi (2023)	[44]	**Depressive Symptoms and Self-Management**<br>Eight studies evaluated depressive symptoms as outcomes of NP care for depression. Two studies found that when PMHNPs provided measurement-based care, depression symptoms decreased at or near statistical significance (p = 0.001 and 0.08; McGuinness et al., 2019; Tave et al., 2017). PMHNP-delivered cognitive behavioral and problem-solving therapy was associated with no significant difference in Hamilton Rating Depression Scale scores compared to usual care (F-statistic = 1.07, p = 0.3; Kirkness et al., 2017).<br><br>PMHNP-delivered motivational interviewing was associated with a significant increase in patient depression self-management (pretest = 49.23, SD = 10.71; posttest = 70.65, SD = 13.74; t = -8.84; p < 0.001). 1/1 study
			Turi (2023)	[44]	**SUD Symptoms (2 studies)**<br>PMHNP delivered measurement-based care was not associated with changes in Alcohol Use Disorders Identification Test scores (3.17 to 2.79, p = 0.211). Talley et al.

					(2021) found that NP-led collaborative care was associated with decreased illicit drug use symptoms among patients who were actively engaged and those that declined services in one clinic (p = 0.029, p = 0.046)
			Turi (2023)	[44]	**Anxiety Symptoms (4 studies)**<br>2/2 studies found that PMHNP delivered measurement-based care was associated with decreases in Generalized Anxiety Disorder (GAD-7) scores that achieved or neared statistical significance (p = 0.001 and 0.08)<br>2/2 studies found that NP-led collaborative care was associated with decreased GAD-7-scores (p < 0.001 and p = 0.028, Talley et al., 2021; GAD-7 scores dropped from 10.0 to 8.1, no p-value reported, 47% of patients had a drop below 5 or 50% of original score).
	**Diagnosis**	3	Carranza (2021)	[59]	**Diagnostic accuracy**: Sig improved with NP group (1/1). Adenoma detection NP vs. MD: 42% vs. 17% (p = .0001)
			Elder (2015)	[63]	**Nurse-initiated X-Rays reduced time to diagnosis and treatment** (from 102.7 min to 65.5 min, P <0.001). 1/3
			Galiana-Camacho	[64]	**Most common diagnoses**: soft tissue (both trauma and loss of skin integrity) (around 35%), and bone fractures in second place (around 11%) (6/6)
	**Education_Patient**	7	Carranza (2021)	[59]	**Patient education**: 2/3 studies favour NP group (no p-values reported), 1/3 no sig differences with care provided by physicians
			Galiana-Camacho	[64]	**Health educatio**n: 98.6% of patients with pharmacological treatment at discharge received information on correct intake; 69.8% received health education; in 41.3% of cases they were given advice on their health problems in written form, and 90.1% received information on who to turn to if they needed help (1/1)
			HQO (2013)	[60]	**Process indicator: Patient education**<br>Model 1: more likely in NP group (p < 0.001)<br>Model 2: (no data)
			Martin-Misener (2015)	[11]	**Patient education** (1 study)<br>Patients had been told the cause of their illness (relative risk (RR) 1.12; 95% CI 1.06 to 1.19; p=0.0001) (HQE), how to relieve their symptoms (RR 1.27; 95% CI 1.19 to 1.34; p<0.00001) (HQE), and what to do if the problem persisted (RR 1.06; 95% CI 1.02 to 1.09; p=0.002) (HQE)

			Morilla-Herrera (2016)	[34]	**Increased knowledge**<br>1/1 study showed increased knowledge of services and who to contact at 3 and 12 months ($p < 0.03$)
			Norful (2019)	[61]	**Patient education**: more likely to be completed for dietary and activity recommendations that included sodium reduction ($p < .001$), moderation in alcohol consumption ($p < .001$), and weight control or reduction ($p <.001$).
			Tsiachristas (2015)	[41]	**Patient information (4 studies)**<br>was found to be higher in 3/4 studies.
	**Mortality**	11	Barker (2018)	[10]	**Mortality** (4 studies)<br>3/4: NS difference in mortality between the groups.<br>1/4: One of the control groups has a lower risk of mortality than the intervention group ($p = 0.013$).
			Carranza (2021)	[59]	**Mortality**: Sig reduced with NP group (1/1). NP vs. MD: 2 (0.3%) vs. 32 (1.5%); HR 0.2, 95% CI [0.04, 0.8]
			Donald (2015)	[27]	**Mortality:** outcomes favoured usual care
			Driscoll (2015) (Ansari paper)	[28]	**Deaths:** $p = 0.05$ (1 NP study)
			Garner (2017)	[56]	**Mortality**: no difference between nurse-led care and rheumatologist-led care in 3/3 studies
			Leduc (2021)	[30]	**Mortality**: no sig difference in 4/4 studies; most studies (3/4) trended toward a slight but insignificant increase in mortality in the intervention groups, with the exception of one intervention (1/4) that had a slight but insignificant decrease in mortality
			Lovink (2017)	[32]	**Mortality** in LTC: no sig difference in deaths in 1/1 study
			Morilla-Herrera (2016)	[34]	**Mortality**<br>3/3 studies reported as non significant results with a trend to increased rate of survival (no p value)

			Newhouse/ Stanik-Hutt (2013)	[35/36]	**Mortality** (1 study). No difference between groups, no p-value reported.
			Schadewaldt (2011)	[39]	**Coronary events, coronary mortality and total mortality (1 study)**<br>**Total mortality** significantly reduced in the intervention group at the 4-year follow up (P = 0.038) and the occurrence of coronary events revealed a borderline difference (P = 0.052) even though some patients of the control group had, by that stage, attended nurse-led clinics.
			Schadewaldt (2011)	[39]	**Coronary events, coronary mortality and total mortality (1 study)**<br>**Total mortality:**<br>No significant differences occurred between the groups concerning all cause mortality, coronary events or deaths due to coronary events at 10-year follow up. However, because of the cross-over effect all patients had the chance to attend the nurse-led clinics within 10 years and detecting differences between the groups was unlikely.
			McParland (2022)	[52]	**Mortality at 90 days (1 study)**<br>Hospital case-managers did not detect any reduction in mortality at 90 days
			McParland (2022)	[52]	**Mortality at 12 and 24 months (1 study)**<br>At 12 months, a community-based nurse case-manager intervention (n = 3432 participants) found significant differences in the proportion of deaths favouring the intervention group at 1 year.<br>At 24 months, trend towards reduction at 2 years was not significant
	**Patient Adherence**	3	Donald (2015)	[27]	**Attendance at cardiac rehabilitation:** Estimates favoured nurse practitioner care
			Kuethe (2013)	[29]	**Compliance with medication** No data presented for patient
			Swan (2015)	[12]	**Follow-up adherence: (two studies)**<br>APNs more frequently requested a return visit and their patients were more likely to keep the appointment.

	**Quality of life**	12	Carranza (2021)	[59]	**Quality of life**: 4/4 no sig difference between NP and physician groups
			Donald (2015)	[27]	**Change in asthma-related quality of life** (Asthma Questionnaire-20; MD: 0.8, 95%CI: 2.22 to 0.62, p = 0.28)
			Donald (2015)	[27]	**Quality of life-respiratory**-related (St. George Respiratory Questionnaire; MD: 1.08, 95%CI: 4.93 to 7.09, p = 0.72).
			Fung (2014)	[55]	**QOL**: When comparing depressed women with cancer in high distress with those in the placebo group, achieved better results in the Short-Form Health Survey-12 mental (P = 0.0001) and physical (P < 0.0001) QOL over time (1/1)
			HQO (2013)	[60]	**HRQOL:**<br>SF-36 score:<br>Model 1: sig improvement from baseline for all participants; no sig difference between groups on the physical and mental components (1/1)<br>Model 2: no difference in the mental component for 2/2 studies and sig deterioration in the physical component for specialized nursing care (MD –3.1; P = 0.04) (1/2).
			HQO (2013)	[60]	**HRQOL diabetes:**<br>Model 2: sig. improvement in diabetes (MD, 5.42; 95% CI, 4.3–10.41) (1/1). No sig. differences for other elements indicating higher QOL. (1/1)
			HQO (2013)	[60]	**HRQOL CAD and CHF:**<br>Model 2: improvement in exertional capacity (MD, 5.25; P = 0.001) and angina frequency (MD, 2.37; P = 0.04) among the nurse-led clinic group (2/2), significant decrease in worsening chest pain (OR, 0.59; 95% CI, 0.37–0.94; P = 0.02). No differences in angina stability, treatment satisfaction, QOL.
			Kuethe (2013)	[29]	**Quality of life (3/3)**<br>QOL improved over time. No statistically significant difference between the nurse-led groups and the physician-led groups<br>After meta-analysis of 2 studies using the same instrument, no effect was found (SMD -0.03; 95% CI -0.23 to 0.17; Figure 3). Sensitivity analysis using a fixed-effect model yielded the same results (SMD -0.03; 95% CI -0.23 to 0.17).
			Lovink (2017)	[32]	**QOL (1 study)**<br>No sig difference between groups (Agvall, 2013, 2014)

			Lovink (2017)	[32]	**Quality adjusted life-years (1 study)**<br>No sig difference between groups (Agvall, 2013, 2014)
			Morilla-Herrera (2016)	[34]	**Quality of life**<br>2/4 studies indicated significant improvements in QoL; 1/4 showed a non significant result with a trend to improved QoL; ¼ showed no significant differences between groups.
			Norful (2019)	[61]	**QOL**: sig findings in 1/3 studies (p = 0.04); non sig changes in 2/3 studies
			Osakwe (2020)	[37]	**NP home visits and QOL:** 1/1 study: At recovery, the total COPD specific questionnaire to assess QOL score decreased for patients who received NP-home visits, it did not reach significance (p = 0.06).
			Osakwe (2020)	[37]	**NP home visits and QOL**: improvement in the activity domain was significant (p< 0.05). 1/1 study
			Schadewaldt (2011)	[39]	**Quality of life and general health perception**<br>Data pooled for 3 of the 4 studies: attending a nurse-led clinic is beneficial for patients in the following domains: physical functioning and physical role limitation with P-values of 0.01 and 0.05, respectively, emotional role limitation (P = 0.03) and general health perception (P = 0.02).<br>No difference between the groups was seen in bodily pain and mental health.<br><br>In the 4th study: improvements for physical functioning, mental health and general health perception, achieving the most significant results for energy and vitality (P = 0.0001) and social functioning (P = 0.0002).<br><br>Across studies, no stat difference at 18 months and 4 years
			Smigorowsky (2020)	[57]	**HRQOL: Effect of NP-led care on length of stay after cardiac surgery (2 studies**)<br>The mean difference for length of stay indicates no significant difference between NP-led care and usual care on length of stay in postoperative cardiac surgery (mean difference [MD] = −0.89, 95% CI: −2.44, 0.66, Z = 1.13, p = .26,) I2 statistic is 0%, therefore low risk of heterogeneity
			Tsiachristas (2015)	[41]	**Quality of life (4 studies)**<br>improved in 2/4 studies and not significantly different in 2/4 studies.

	**Satisfaction: Patient and family**	18	Ansell (2017)	[65]	**Patient satisfaction with open access**: 3 studies. 16% increase post-intervention implementation in 1/3; no change in 2/3 studies. No p-values reported.
			Carranza (2021)	[59]	**Patient satisfaction:** sig. higher with NP group in 4/7 studies (p-values reported for ¾ studies); not sig difference in 3/7
			Donald (2015)	[27]	**Patient satisfaction post hysterectomy (effect size: 14 (**95%CI: **3.5 to 24.5) <0.01** (low quality)
			Donald (2013)	[54]	**Resident satisfaction** (1 study) with medical services did not differ statistically
			Donald (2013)	[54]	**Family members satisfaction** significantly improved with 3 items including: [resident] seen often enough to treat problems, physician/ NP spends enough time with patient and one person in charge (1/1).
			Donald (2013)	[54]	**Family member dissatisfaction** lower in the intervention group (1/1).
			Galiana-Camacho	[64]	**Patient satisfaction**: 97.3% of the patients were satisfied with the time devoted to their care, to deal with everything related to their health process. 83.8% of patients would return to return to the APN for similar problems, 14.4% would not, and 96.4% would recommend this care to a friend.<br>The results of the satisfaction in the 5 categories of the questionnaire reached the highest level of satisfaction in 82% of the cases. Regarding the overall evaluation of care received, 82% rated it as "excellent", 15.3% as "good" and 15.3% as "good", and 0.9% as "poor"(2/2)
			HQO (2013)	[60]	**Patient satisfaction:**<br>Model 1: no sig differences between groups (p = 0.87) (1/1)<br>Model 2: increase in patient satisfaction for patients receiving specialized nursing care, one study assessed significance (2/2)
			Ismail (2013)	[66]	**Satisfaction:** rates with the scheme from A&E doctors, health workers, and residential care staff was high, but cost data were not reported. (Codde, et al., 2010)

			Jennings (2015)	[67]	**Satisfaction** (3/3): higher satisfaction.<br>After adjusting for waiting time the emergency NP service still maintained a 1.5 x higher mean total satisfaction score (beta coefficient = 1.5, p = 0.004, 95% CI, 0.48–2.5) (Dinh 2011)<br>Responses to 12 out of 16 questions in a survey demonstrated a significant between the two groups in favour of the emergency nurse practitioner services (p < 0.05) (Jennings 2009)<br>100% of patients seen by emergency nurse practitioner services in the fast-track area scored their care as good or excellent (Nash 2006)
			Martin-Misener (2015)	[11]	**Patient satisfaction** (2 studies)<br>meta-analysis of two studies in which the nurse practitioners had at least 1 year experience, nurse practitioner care was associated with higher patient satisfaction (1515 patients; I2=0%) (mean difference: 0.15 (95% CI 0.11 to 0.20); p<0.0001) and parent satisfaction (804 parents; I2=0%) (mean difference: 0.23 (95% CI 0.16 to 0.30); p<0.0001).
			Martin-Misener (2015)	[11]	**Patient satisfaction with newly established NP roles (1 study)**<br>nurse practitioner care was associated with higher patient satisfaction in the subgroup of patients with chronic disease (583 patients) (mean difference: 0.24 (95% CI 0.05 to 0.43); p=0.02) (11-point Likert scale; LQE).
			Morilla-Herrera (2016)	[34]	**Patient satisfaction with ED discharge care**<br>1/2 studies found increased satisfaction with ED discharge (3.41 versus 3.03; mean difference 0.37; 95% CI 0.13– 0.62). The intervention was more effective for high-risk than low risk aged people. ½ studies found no significant group differences in patient satisfaction.
			Newhouse/ Stanik-Hutt (2013)	[35/36]	**Patient satisfaction with provider/care**: 4 studies; 1/4 studies reported findings favouring NP group, 3/4 reported no diff. between groups (no p-values reported)
			Patel (2019)	[38]	**NP SOP and patient satisfaction with care**: 2 studies<br>One study reported a smaller percentage of the population not seeking care due to costs in two states with less restrictive versus two other states' most restrictive NP SOP policies (Sonenberg & Knepper, 2017), contradictory findings from another study reported increased patient difficulties with cost in states with the least restrictive NP SOP policies (Cross & Kelly, 2015). This study also found that patient

					satisfaction with usual source of care and wait times was worse in states with the least restrictive NP SOP policies (Cross & Kelly, 2015).
			Swan (2015)	[12]	**Patient satisfaction (4 studies)**<br>Three studies/4 demonstrated higher patient satisfaction among patients who received care from APNs; one/4 study reported higher satisfaction among patients who received care from APNs at three of their ten study sites.
			Thomas (2019)	[40]	**Patient satisfaction at three months**: The CNP intervention may have improved participant satisfaction at three months (dissatisfaction: 13/109 (12%) in the treatment group versus 17/45 (38%) in the control group; RR 0.32, 95% CI 0.17 to 0.59
			Tsiachristas (2015)	[41]	**Patient satisfaction (8 studies)**<br>5/8 studies showed positive results, no p values reported
			Jeyaraman (2022)	[45]	**Patient satisfaction (3 studies)**<br>2/3 studies reported high level of patient satisfaction with NP-led triage model (p values not reported).<br>1/3 studies reported a slight decrease in patient satisfaction with NP led-triage without reaching statistical significance
			McParland (2022)	[52]	**Patient satisfaction (1 study)**<br>Significant improvement in communication 1/1 study, no changes in other dimensions of patient satisfaction
			Sun (2022)	[53]	**Patient-family satisfaction (4 studies)**<br>More than 95% of patient participants were satisfied with the NP intervention in 2/2 studies. (p value not reported)<br>Caregiver satisfaction was significantly higher in the intervention group (99.8 vs 88.8, p <.05) in ¼ studies. Caregiver satisfaction increased 0.2 points on a 10-point scale postintervention, although it was not statistically significant.
	**Signs and Symptoms**	9	Carranza (2021)	[59]	**Symptom management**: (4/4 no sig difference between NP and physician groups)
			Carranza (2021)	[59]	**Symptom improvement:** Sig improved with NP group (1/1)
			Donald (2015)	[27]	**Urinary tract infections:** outcomes favoured usual care

			Garner (2017)	[56]	**Pain** assessed in 4/4 studies. Sig reduction in ¾ studies and no difference in ¼ studies at 12 and 24 months
			Garner (2017)	[56]	**Fatigue** assessed in 3 studies. Sig. less fatigue in 2/3 studies and no difference noted at 12 months and 24 months in 1/3 studies.
			Garner (2017)	[56]	**Arthritis impact** assessed in 3 studies. No differences noted in 3/3 between nurse-led care and rheumatologist-led care or trainee rheumatologist-led care.
			Kuethe (2013)	[29]	**Symptom-free days**<br>No sig difference between the paediatrician-led group and the nurse-led group (p =0.54). (1/1, Kamps, 2003)
			Morilla-Herrera (2016)	[34]	**Symptoms of dementia (NPI score)**<br>1/1 study showed significantly fewer behavioral and psychological symptoms of dementia as measured by the total NPI score at 12 months (mean difference, -5.6; P: 0.01) and at 18 months (mean difference, -5.4; P: 0.01)
			Norful (2019)	[61]	**Cognitive and behavioural changes**: 2/2 studies: no significative cognitive and behavioral changes.<br>Significant change when using different assessment tools (p=0.01).
			Schadewaldt (2011)	[39]	**Angina symptoms**<br>1/2 studies: Statistically significant differences between the groups at 1-year follow up were found in exertional capacity (P = 0.0014) and angina frequency (P = 0.0452), where the intervention group scored higher and therefore had less angina symptoms.<br><br>No differences between usual care and nurse-led clinics were identified in other domains of the questionnaire such as stability of angina symptoms, satisfaction with treatment and quality of life.<br>½ studies: No significant difference between the groups at 1-year and 4-year follow up, except that worsening chest pain was experienced significantly less frequently in the intervention group (P = 0.025) after attending a nurse-led clinic for 1 year
			Schadewaldt (2011)	[39]	**Angina symptoms**<br>No differences in hospital admissions at the 10 year follow up in one study

			Thomas (2019)	[40]	**Number of participants continent after treatment**: Structured assessment and management of incontinence probably made little or no difference to the number of people continent three months after treatment (risk ratio (RR) 1.28, 95% CI 0.81 to 2.02; 121 participants; equivalent to an increase from 354 to 453 per 1000, 95% CI 287 to 715). At six months, the CNP intervention may have made little or no difference to the number of people continent after treatment (16/91 (17.5%) in the treatment group versus 8/55 (14.6%) in the control group; RR 0.96, 95% CI 0.83 to 1.11).
			Thomas (2019)	[40]	**Urinary symptoms (frequency, nocturia, urgency and urinary incontinence) at three months**: number of participants cured of all four urinary symptoms was 24.7% in the treatment group versus 17.9% in the control group (147 participants). At six months, Brittain 2000b found a larger proportion of people were cured of all four urinary symptoms in the treatment group (41/89; 46.1%) compared with the control group (16/54; 29.6%) (RR 1.55, 95% CI 0.97 to 2.48)
			Thomas (2019)	[40]	**Changes in daytime and night-time leakage**: no data provided
			Thomas (2019)	[40]	**Daytime severity of leakage at three months:** (P = 0.038) possibly improved following the intervention
			Thomas (2019)	[40]	**Total number of symptoms experienced at three** months: may have slightly reduced after the intervention (P < 0.01).
			Thomas (2019)	[40]	**Total number of overall symptoms at six months:** little or no difference (P = 0.06).
			Thomas (2019)	[40]	**Urinary frequency at three months**: No evidence that the CNP intervention made any difference 98/120 (82%) in the treatment group versus 59/67 (88%) in the control group; RR 0.93, 95% CI 0.82 to 1.05; 187 participants.
			Thomas (2019)	[40]	**Urinary frequency at six months**: The CNP intervention may have made little or no difference to urinary frequency (73/89 (82%) in the treatment group versus 47/54 (87%) in the control group; RR 0.94, 95% CI 0.82 to 1.09).
			Thomas (2019)	[40]	**Urinary urgency at three months**: no difference The number of people reporting urgency was 95/121 (79%) in the treatment group compared with 50/67 (75%) in the control group (RR 1.05, 95% CI 0.89 to 1.24; 188 participants).

			Thomas (2019)	[40]	**Urinary urgency at six months:** the CNP intervention may have made little or no difference to (65/91 (71.4%) in the treatment group versus 40/54 (74%) in the control group; RR 0.96, 95% CI 0.79 to 1.18).
			Thomas (2019)	[40]	**Nocturia at three months**: the CNP intervention may have made little or no difference to nocturia (Brittain 2000b). The number of people reporting nocturia at three months was 102/119 (86%) in the treatment group versus 60/67 (90%) in the control group (RR 0.96, 95% CI 0.86 to 1.07).
			Thomas (2019)	[40]	**Nocturia at six months:** At six months, the CNP intervention may also have made little or no difference to nocturia (77/89 (87%) in the treatment group versus 46/53 (87%) in the control group; RR 1.00, 95% CI 0.87 to 1.14).
			Wu (2019)	[68]	**Management of asymptomatic urinary tract infection**: improvement in following supportive strategies: increased fluids (p < .001), frequent toileting (p < .001), and cranberry juice (p < .05).
**Provider**					
	**Adherence to best practice-Providers**	6	Donald (2015)	[27]	**Compliance with aspirin:** Outcomes favoured usual care
			Donald (2015)	[27]	**Compliance with clopidogrel:** outcomes favoured usual care
			Donald (2015)	[27]	**Compliance with beta-blockers :**estimates favoured nurse practitioner care
			Donald (2015)	[27]	**Compliance with statins:** estimates favoured nurse practitioner care
			Donald (2015)	[27]	**Compliance with angiotensin-converting enzyme inhibitors:** estimates favoured nurse practitioner care
			Lovink (2017)	[32]	**Renin–angiotensin system blockade (1 study)**<br>68 patients on treatment with renin–angiotensin system blockade in the control group compared with 79 in the intervention group (P = 0.002).<br>Percentage mean dosage of renin–angiotensin system blockade of the **optimal dosage** was 94% compared with 69% in the control group (P < 0.001). (Agvall, 2013, 2014)

			Lovink (2017)	[32]	**Adherence and compliance to guidelines, protocols and quality of healthcare**: reported in 4 studies.<br>2 studies: not stat. sig. (Aigner and Everett et al. (2013a,b) and 2 studies: stat. sign. (Cardozo et al. (1998a,b) and Reuben et al. 2013).<br>In LTC: No significant difference was found in the number of annual mandatory histories and physical examinations performed. 1/1 study
			Lovink (2017)	[32]	**Assessing Care of Vulnerable Elders-3 (ACOVE-3)** quality indicators in favour of the intervention, (P < 0.001), 1/1 study
			Lovink (2017)	[32]	**Overall performance rate on secondary prevention** performance : In primary care, sig improvement in 1/1 study, (p < 0.001),
			Norful (2019)	[61]	**Adherence to recommended care**: Indicators including completed guidelines for patients with dementia (p < .001), falls (p = .00), incontinence (p = .01), and all diagnoses (p < .001). 4/4 studies found sig results for adherence to care and co-management
			Norful (2019)	[61]	**Medication compliance**: no sig differences between the groups (1/1)
			Schadewaldt (2011)	[39]	**Compliance:**<br>No sig difference between groups in ½.<br>Sig difference in ½ studies: compliance with aspirin intake at 1-year follow up adherence to correct aspirin intake was significantly higher in the intervention group (P < 0.001).
			Swan (2015)	[12]	**Clinician guideline adherence (3 studies)**<br>One/3 found that APNs had higher rates of providing disease-appropriate care across five of six indicators examined (p value not reported).<br>There were no differences regarding the proportion reporting that they were advised of the likely duration of their illness and how to reduce the chances of recurrence.<br>Kinnersley et al., reported that patients assigned to the physician group were less likely to report having been told the cause of their illness (odds ratio [OR] 0.58, 95% confidence interval [CI] 0.44–0.76), how to relieve symptoms (OR 0.32, 95% CI 0.24–0.43) and what to do if the problem persisted (OR 0.61, 95% CI 0.41–0.90).

			Turi (2023)	[44]	**Mental Health Guideline-Recommended Care (4 studies)**<br>No significant differences in the care provided by NPs related to adherence to guidelines for medications, counselling, cognitive behavioral and problem-solving therapy to older adults, patient monitoring and motivational interviewing in 4/4 studies
	**Education_Provider**	5	Galiana-Camacho (2018)	[64]	**Professional competencies using the Australasian Triage Scale** (ATS) with scores of between 4-5 (minimum of 5 and maximum 1) (6/6)
			Hyer (2019)	[69]	**Patient counseling on obesity:** 65% of primary care physicians, obstetricians, and NPs surveyed believe this is a shared responsibility between the patient and the provider (Petrin et al., 2017). (1/1)
			Loescher (2018)	[31]	**Current knowledge**<br>Five studies reported on NPs' knowledge of skin cancer detection (Blake & Malone, 2014; Bradley, 2012; Chen et al., 2015; Hartnett & O'Keefe, 2016; Shelby, 2014). Correct responses on overall knowledge tests ranged between 26% and 85% (Bradley, 2012; Chen et al., 2015; Shelby, 2014).
			Loescher (2018)	[31]	**NP attitudes towards the early detection of skin cancer.** (3 studies) 41% reported No to mild confidence to most of participants feeling a lack of confidence with basic dermatology examinations. 84% agreed that "dermatology training prepared me for practice." In one study.

			Loescher (2018)	[31]	**Skin Lesion identification by NPs**<br>Knowledge of clinical examination is low but improves with training. Accuracy of skin lesion identification, errors tended to occur with benign skin lesions.<br>One study found poor agreement between a naked-eye examination and a dermoscopic examination by the one NP (Armstrong, 2011)<br>Correct lesion identification percentages ranging from 22% (a KC) to 76% (a melanoma) study by Shelby, 2014).Wray et al. (2013) found that diagnostic skills of one NP, which increased from 44% in the first three months to 67% in the remaining seven months of the study. An NP leading a skin cancer screening program found suspicious lesions in 46% of the participants, including skin cancers and precancers; however, these were not all histologically confirmed (DeKoninck & Christenbery, 2015).<br>Improved skin lesion identification scores on five image items from pretest to posttest in 2 studies (Bradley (2012); Hartnett and O'Keefe (2016)).<br>Improving the accuracy of diagnosis (Wray et al., 2013).<br>Improving the practitioner–dermatologist agreement of diagnosis (Armstrong, 2011)
			Loescher (2018)	[31]	**Skin cancer detection training**<br>**Didactic training in 3/5 studies and informal training in 2/5**<br>Didactic educational program improved skin lesion recognition and resulted in a 223% increase in participants' proper documentation of skin cancer screening and patient education in one study (Bradley, 2012)
			Lovink (2017)	[32]	**Orientation in LTC**: significant improvement in 1/1 study ($p = 0.02$),
			Stratton (2014)	[70]	**Intervention components and activities for NPs performing clinical skin examination**: (10 studies)<br>10/10 studies reported a didactic and a clinical portion or clinical apprenticeship with a dermatologist, with 8/10 8 focused on head-to-toe skin examination<br>6/10 studies did not specify the strategy for conducting skin lesion assessment<br>Feedback on the NPs prior dermatology referrals to guide her education 1/1 study
			Stratton (2014)	[70]	**Intervention dosing**<br>The length of their didactic sessions (1 session of 14 min up to 6 months), timing and frequency were not specified, most sessions occurred one time (4 studies)<br>Not mentioned in 3 studies.

			Stratton (2014)	[70]	**Mode of delivery of the intervention**:<br>7/10 reported using face to face and observations by experts
	**Illness Prevention**	7	Carranza (2021)	[59]	**Metabolic outcomes:** Overall improvement in metabolic outcomes, but no p-values reported.<br>NP vs. MD, within-group decrease (between-group difference):<br>-HA1C: 2.5% vs 0.2% (2.3%)<br>-BP: No difference within and between groups<br>-Weight loss: 8.3 lbs. vs. 7.4 lbs. (0.9 lbs.)<br>-Glucose: 83.7 mg/dl vs. 27.4 mg/dl (56.3 mg/dl)
			Donald (2015)	[27]	**Smoking cessation:** Outcomes favoured usual care
			Fung (2014)	[55]	**Feasibility of screening of women with depression (1 study)**<br>Increase in the mean efficacy self-esteem score post-intervention and a significant difference between pre- and post-intervention BDI scores ($t = 8.765$, d.f. = 29, $P = 0.0005$) in a paired samples t-test. (1/1)
			Garner (2017)	[56]	**Health assessment** assessed in 4/4 studies. Equal to superior care indicated at 12 and 24 months
			HQO (2013)	[60]	**Process indicator: height**<br>Model 1: ($p < 0.01$)<br>Model 2: (no data)
			HQO (2013)	[60]	**Clinical examination:**<br>Model 2: Patients in the specialized nursing group received significantly more assesments of smoking status ($P < 0.0001$) among CAD patients (3/3).
			HQO (2013)	[60]	**Clinical examination:**<br>Model 2: Patients in the specialized nursing group received significantly more assesments of BMI/weight ($P < 0.0001$) among CAD patients (3/3).
			Schadewaldt (2011)	[39]	**Smoking**<br>No sig results in 3/3 studies
			Schadewaldt (2011)	[39]	**Smoking cessation in the long term for up to 1 year**<br>½ studies sig for smoking cessation ($p = 0.03$)<br>In 1/3 studies showed sig results at 12 months ($p = 0.01$)

			Schadewaldt (2011)	[39]	**Bodyweight**<br>No sig difference in 3/3 studies
			Schadewaldt (2011)	[39]	**Compliance**:<br>No sig difference between groups in ½.<br>Sig difference in ½ studies: Significant benefits in continuing physical exercise (P = 0.001).
			Schadewaldt (2011)	[39]	**Compliance**:<br>No sig difference between groups in ½.<br>Sig difference in ½ studies: Significant benefits in diet (P = 0.009) after 1 year in nurse-led group.
			Smith (2014)	[49]	**Cervical cancer screening (5 studies)**<br>3/4 studies evaluating Pap tests, most showed that APRN/PAs provide or recommend Pap tests to patients (72% to 98%) and that physicians who currently work with APRN/PAs are amenable to APRN/PAs conducting Pap tests<br>1/1 intervention study compared NPs recommending and performing cervical cancer screening during routine visits to a provider reminder system. At follow-up there was a significant increase in the annual rate of women screened for cervical cancer by a NP at the intervention location (from 17.8% to 56.9%), while the annual rate of screening by physicians at the control location improved less (from 11.8% to 18.2%) during the study time period, $p = 0.006$, after adjusting for patient characteristics.
			Smith (2014)	[49]	**Breast cancer screening (3 studies)**<br>2/3 studies showed that a majority of patients who see NPs receive mammograms (69% to 91%) ($p < 0.01$) and that NPs recommend a similar amount of mammograms as physicians. 1/3 not sig.<br>1/1 a majority of patients who see NPs receive mammograms (69% to 91%) and that NPs recommend a similar amount of mammograms as physicians, p values not reported.
			Smith (2014)	[49]	**Colorectal cancer screening (4 studies)**<br>¾ studies showed a range of reported colorectal cancer screening provided or recommended by APRN/PAs (19% to 95%) ($p < 0.001$). Large variation due to differences in reporting between studies (e.g., chart audit, self report)<br>2/4 studies showed that physicians reported more colorectal cancer screening than APRN/PAs; $p < 0.001$ in one study; not reported in other study.

			Smith (2014)	[49]	**HPV vaccination**<br>No study of HPV vaccination identified.
	**Interprofes - sional Team Functioning**	4	Hyer (2019)	[69]	**Provider perceptions of high functioning interdisciplinary team approach:** (2/2) positively affected the patient provider experience (Asselin et al., 2016; Asselin et al., 2017).
			Lovink (2017)	[32]	**No provider outcomes reported**
			Lovink (2017)	[32]	**No outcomes related to providers**
			Turi (2023)	[44]	**Collaborative Care (6 studies)**<br>NP-led collaborative care where the PCP was an NP and psychiatric support was provided by a PMHNP. NP-led collaborative care where PCP NPs screened patients who screened positive; high-acuity patients were referred from the PCP NP to the PMHNP. After one year, the number of case reviews per month were higher (5 to 15) and the number of referrals to the PMHNP were lower (19 to 5). (no p value, effect measured using depressive symptoms).
			Turi (2023)	[44]	**NP-led collaborative care (5 studies)**<br>NP-led collaborative care led to clinical or statistical improvements in depressive symptoms in 5/5 studies
			McParland (2022)	[52]	**Team Functioning (1 study)**<br>Staff perceived that patient care had improved following the intervention, also identified gaps particularly in communicating with secondary care in 1/1 study
	**Prescribing**	15	Barker (2018)	[10]	**Prescribing** (unchanged 2/3 (one p value not reported); 1/3 reduction)
			Carranza (2021)	[59]	**Treatment success**: 4/4 no sig difference between NP and physician groups

			Driscoll (2015) (Ansari paper)	[28]	**Patients initiated or up-titrated on beta-blockers**: (1 NP study)<br>Proportion of patients with stable CHF in outpatient settings who were initiated or up-titrated and maintained on beta-blockers, was achieved in 67% (36 of 54) of patients in the nurse facilitator group compared with 16% (10 of 64) in the provider/patient notification and 27% (14 of 51) in the control groups (P < 0.001) for the comparisons between the nurse facilitator group and both other groups). (1/1)
			Driscoll (2015) (Ansari paper)	[28]	**Patients initiated on beta-blockers:** (1 NP study)<br>Proportion of patients with stable CHF in outpatient settings who were initiated on beta-blockers (beta-blocker-naïve at the start of the study) was highest in the nurse facilitator group at 61% (22/54), compared with control (29%) and provider/patient notification groups (12%) (P<0.001). (1/1)
			Driscoll (2015) (Ansari paper)	[28]	**Patients at target beta-blocker doses at end of study**: (1 NP study)<br>Proportion of patients with stable CHF in outpatient settings on target beta-blocker doses at the study end (median follow-up, 12 months) was also highest in the nurse facilitator group (43%) compared with the control (10%) and provider/patient notification groups (2%) (P < 0.001). (1/1)
			Garner (2017)	[56]	**Prescribing laboratory investigations:** trainee rheumatologist-led care ordered more laboratory investigations (relative risk 0.46, 95% CI 0.2–0.9; p = 0.03) (Hill 2003)
			HQO (2013)	[60]	**Medication Prescribing**<br>Model 2: sig. differences in prescription to intensify glucose-lowering therapy (p = 0.0005) or blood pressure medication (p = 0.01), number of referrals to internists were higher (p < 0.001), and influenza vaccination (p<0.0001), significant increase in the primary outcome of the appropriate prescribing of beta-blockers among individuals with a prior myocardial infarction (P = 0.03) Diverse results for Aspirin use ½ study with sig results. No sig differences in lipid-lowering agents (p= 0.07), no significant difference in the prescribing of an angiotensin converting enzyme (ACE) inhibitor among patients with confirmed LVSD (P = 0.05). However, it was not stated how many patients were already on insulin or if this increase reflected more appropriate referrals in comparison to physicians (P = 0.03) (4/4).<br><br>Two studies reported on Aspirin use, with Khunti et al finding no significant difference in the proportion of patients receiving aspirin (P = 0.55), and Campbell et al observing a significant increase in use (P < 0.001) (GRADE: low)

			Hyer (2019)	[69]	**Prescribing:** NP prescribe from rarely to half of study participants reporting the practice of using weight-loss pharmaceuticals (3 studies); the reported thresholds for weight-loss pharmaceuticals, that prompt the prescription were not aligned with national guidelines of pharmacotherapy (Granara & Laurent, 2017; Petrin et al., 2016). Referrals for bariatric surgery varied greatly from rarely making a referral to 70% of participants referring out for consultation.
			Kuethe (2013)	[29]	**Use of rescue medication** No sig difference in Kamps (p = 0.40) or Nathan (not sig) in unstable patient group (Mean number of exacerbations requiring emergency treatment was 0.59 in nurse-led group vs 0.43 in the physician-led group)
			Kuethe (2013)	[29]	**Evidence of stepping down therapy** (1/1)<br>No sig differences between the groups
			Lovink (2017)	[32]	**Beta-blockers**: No sig. difference in patients receiving this treatment (Agvall 2013, 2014) (1/1).
			Lovink (2017)	[32]	**Beta-blockers optimal dose**: No sig difference (Agvall 2013, 2014) (1/1)
			Lovink (2017)	[32]	**In LTC: Average number of medications:** no sig difference in 1/1 study
			Martin-Misener (2015)	[11]	**Number of prescriptions.**<br>Nurse practitioner and general practitioner care were equivalent. Non sig.
			Martin-Misener (2015)	[11]	**Number of investigations ordered or carried out.**<br>Nurse practitioner and general practitioner care were equivalent. Non sig.
			Morilla-Herrera (2016)	[34]	**Prescriptions-cholinesterase inhibitors**<br>1/1 study showed that patients were more likely to receive cholinesterase inhibitors (p = 0.002)
			Morilla-Herrera (2016)	[34]	**Prescriptions-antidepressants**<br>1/1 study showed that patients were more likely to receive antidepressants (p = 0.03)
			Ness (2016)	[48]	**Totals and percentages of the type of antimicrobial (AM)** selected, demonstrated that nurse practitioners in this study chose traditional and often low-cost

					antimicrobial medications and that these choices were made because the drugs were first-line choices (1/1)
			Ness (2016)	[48]	**Prescribing decisions** based on: Efficacy (94,2%) and tolerability (77,1%) were most frequently identified as very important influences on their prescribing decisions with guideline recommendation coming third (74%). (2/2)
			Ness (2016)	[48]	**Prescribing decisions** in pediatric settings are: always (3,4%) or very often (51,6%) based on watchful waiting instead of prescribing an antibiotic as the first strategy for managing otitis media. (1/1)
			Ness (2016)	[48]	**Prescribing decisions when no culture is available**: majority of respondents (60,3%) stated that they would start with a broad spectrum antimicrobial and then tailor upon culture results (1/1)
			Ness (2016)	[48]	**Predictors of prescribing an anti-microbial**: strongest negative predicator (i.e. would not be prescribed an AM) within the nurse practitioners was Medicaid insurance (p = 0,012). Patients with Medicaid insurance were 75% less likely to be prescribed an AM. The strongest positive predictors were that NPs were significantly more likely to prescribe an antimicrobial were geographic region (p = 0,001), black non-Hispanic race (p = 0,047) and when both pharyngitis and bronchitis were diagnosed as compared to patients with a nonspecific viral upper respiratory tract infection (p = 0,001). (1/1)
			Swan (2015)	[12]	**Prescription patterns for medications (three studies)**; one study reported: no differences in APN medication prescriptive practices.
			Swan (2015)	[12]	**Prescription of diagnostic tests (three studies)**; one study reported that APNs more frequently ordered diagnostic tests.
			Wu (2019)	[68]	**Antibiotic prescribing rates:** No significant differences
			Zhang (2020)	[43]	**Meta-analysis (26 studies)**<br>**Stage of pre-exposure prophylaxis (PrEP) implementation cascade between NPs and physicians**: being aware of pre-exposure prophylaxis (PrEP) among NPs were 37% (OR = 0.63, 95% CI = 0.46–0.87) less than that among physicians.

			Zhang (2020)	[43]	**Meta-analysis (26 studies)**<br>**Prescribing pre-exposure prophylaxis (PrEP):**<br>Odds of being willing to prescribe pre-exposure prophylaxis (PrEP) among NPs was not different from that among physicians (OR = 1.00, 0.98, 1.02).<br>The odds of prescribing pre-exposure prophylaxis 1.40 (95% CI = 1.02. 1.92) times higher among NPs than that among physicians.<br>No significant difference for location and study design. The odds ratio of prescription between NPs and physicians was highest in 2015 among all available studies (OR = 1.94, 95% CI = 1.12, 3.36)<br><br>Low to moderate between-study heterogeneity was detected across studies evaluating PrEP awareness (I2 = 0.00%), willingness (I2 = 54.00%), and prescription (I2 = 55.50%).<br>Sensitivity analyses, including or excluding studies with extreme weights, showed no significant difference.
			Turi (2023)	[44]	**Prescribing (3 studies)**<br>NPs in community health centers prescribed more antidepressants (70.4% vs. 61.6%, p = 0.03) and the same number of anxiolytics (24% vs. 33%, p>0.05) in 1 study. NPs prescribed more anti-anxiety and antidepressants in rural areas vs. urban areas (p < 0.001) in 1 study.<br><br>No difference in NP-attributed patients in the odds of receiving alcohol use disorder pharmacotherapy compared to physician-attributed patients (primary care NP: AOR = 1.00, 95% CI = 0.69 – 1.46; psychiatry NP: AOR = 1.33, 95% CI = 0.67 – 2.65).
**Health system**					
	**Access to care**	5	Ansell (2017)	[65]	**Open access scheduling**: Increases access to care in 11/11 studies. No p-values reported.
			Carranza (2021)	[59]	**Access to care**: reported 2 studies. Pediatric NP saw greater percent urgent appointments (12.5% vs. 6.2%, *p* < .001) (1/1); NPs rated higher: Access between visits (93.1% vs. 63.2%), no p-values reported (1/1).
			Milesky (2020)	[47]	**Increased access to healthcare** in 10.3 % of theme occurrences

			Stratton (2014)	[70]	**treatment for precancerous lesions**: 18.3% of the participating NP's patients (n = 828) had treatment and 6.2% were treated for nonmelanoma skin cancer in NP led surveillance clinic during the first two years of the clinic's implementation. No treatment available before clinic implementation. (1 study)
			Yang (2020)	[58]	**Access to care (4 studies)**<br>¾ studies reported significantly greater primary care access (no p-values reported); little difference in 1/4 studies (difference not statistically tested)
	**Consultations**	11	Carranza (2021)	[59]	**Length of consultation times**: no reported measure in supplemental table
			Donald (2015)	[27]	**Number of rehabilitation patient-to-staff consultation calls** ($p < 0.05$): significant reduction
			Donald (2015)	[27]	**Duration of rehabilitation patient-to-staff consultation calls** ($p < 0.05$): significant reduction
			Donald (2015)	[27]	**Total number of consultation calls**: Rehabilitation 1 versus 7 calls $p < 0.05$; reported 1/1
			Donald (2015)	[27]	**Total duration of consultation calls**: Rehabilitation 5 versus 48.5 min $p < 0.05$; reported 1/1
			Garner (2017)	[56]	**Efficiency: referrals, conferrals (consultations). 3 studies**<br>relative risk of a conferral in the Nurse-led arm was 1.45 (95% CI 1.0–2.1; p = 0.04 (Hill 2003) and was 3.22 (95% CI 2.1–5.0; $p < 0.001$) for patients attending nurse-led care (Ndosi).<br>Higher number of visits to a rheumatologist in the nurse-led care arm than in the rheumatologist-led care arm (mean 1.63 and 1.53, respectively), which was attributed to the restriction in a nurse's ability to prescribe and perform joint injection, and more rheumatology nursing visits (mean 2.28 and 1.5, respectively) (Watts).
			Garner (2017)	[56]	**Nurse referrals to other providers (4 studies)**<br>relative risk 5.3, 95% CI 3.4–8.2; $p <0.0001$ (Hill 1994);<br>relative risk 2.8, 95% CI 1.8–4.2; $p < 0.0001$ (Hill 2003);<br>Ndosi, et al documented referrals and found no statistically significant difference between nurse-led care and rheumatologist-led care. Ryan, et al found that the number of referrals by nurse-led care and rheumatologist-led care to rheumatologists or family physicians was similar.
			Garner	[56]	**Consultation time (2 studies)**

			(2017)		Longer in the intervention group 2/2 (no p-values reported).
			HQO (2013)	[60]	**Clinical examination (3 studies)**<br>Model 2: Patients in the specialized nursing group received significantly more referrals for echocardiographs among patients with presumed CHF ($P < 0.01$) among CAD patients (3/3).
			Kuethe (2013)	[29]	**Referrals from primary care hospital**<br>No data presented in any of the included studies
			Kuethe (2013)	[29]	**Duration of consultation with the asthma nurse and the physician**<br>Duration of the first nurse-led follow-up visit was 29.0 (5.2) minutes. The second and third follow-up visits lasted 19.4 (7.2) and 18.3 (6.3) minutes, respectively.<br>Subsequent nurse-led follow-up visits lasted approximately 15 minutes.
			Kuethe (2013)	[29]	**Duration of consultation with the physician** (Kamps, Kuethe, 2011)<br>No data about duration of consultation were presented for the paediatrician-led group.<br>Eight per cent of the children had problems that required more frequent input from the paediatrician.
			Loescher (2018)	[31]	**Decreased the number of unnecessary referrals to dermatologists in 2/2 studies** (Ali et al., 2014; Armstrong, 2011).
			Lovink (2017)	[32]	**In LTC: Unplanned consultations for acute conditions** increased sig. in intervention group, 3.0 vs 1.2 per year ($P < 0.0001$), 1/1 study
			Martin-Misener (2015)	[11]	**Consultation times (3 studies)**<br>Nurse practitioners had longer consultation times than general practitioners.3/3 studies<br>Meta-analysis of two studies with over 2500 patients, the mean total consultation time in the nurse practitioner group was 4.1 min longer per patient (95% CI 3.7 to 4.5; $p<0.0001$).<br>Heterogeneity was high ($I2=97\%$).<br>Nurse practitioner consultations were significantly longer in 8 of 10 practices; the ratio of general practitioner to nurse practitioner consultation times varied from 0.57 (95% CI 0.49 to 0.67) to 0.92 (95% CI 0.7 to 1.21). 1 study
			Martin-Misener (2015)	[11]	**Number of patients who were referred:**<br>Nurse practitioner and general practitioner care were equivalent. Non sig.

			Swan (2015)	[12]	**Number of referrals (3 studies)**<br>Two/ 3 investigated the number of specialty care visits; both found no differences between APNs and physicians.
			Van Vliet (2020)	[42]	**Referral (1 study)**<br>PAs refer 50% of their patients to another health care professional (e.g., a GP or an emergency department (ED)) while nurses referred 73% (p value not reported).
			Van Vliet (2020)	[42]	**Consultation (1 study)**<br>PAs consulted other health care professionals (e.g., a GP, an emergency physician, or a medical specialist) significantly more often compared to nurses (p value not reported).
			Yang (2020)	[58]	**Referral pattern (1 study)**<br>1.8 times higher odds of physician referral in states with FPA versus those in restricted practice states.
	**Costs**	17	Carranza (2021)	[59]	**Costs:** sig. higher for NP group vs physicians in (1/1) for patients with lung disease. Mean difference £1497 (95% CI [688, 2674], p < .001)
			Donald (2015)	[27]	**Cost effectiveness**: no significant differences between groups for any patient outcomes or any health system outcomes (1/1)
			Donald (2015)	[27]	**Total costs** Hysterectomy: 6% savings sig not reported
			Donald (2015)	[27]	**Hospital costs (30 days) Frail elderly**, -$134 (95%CI:-$644 to $376), p = 0.61
			Donald (2015)	[27]	**Hospital costs (90 days) Frail elderly** -$497 (95%CI:-$1216 to $222), p = 0.18
			Donald (2015)	[27]	**Hospital costs (180 days) Frail elderly** -$488 (95%CI:-$1290 to $314), p = 0.23
			Donald (2013)	[54]	**Cost-per-patient** the sum of the costs associated with primary-care encounters, non-hospital, hospital, and nursing home care was nearly the same for both nursing home sub-groups (1/1)
			Fraser (2018)	[46]	**Fees for service:** Annual total savings if APRNs and physician assistants assume all the primary care visits in one state (Florida), total savings are estimated at $339 million
			Fraser (2018)	[46]	**Healthcare costs to organization**: in the first 6 months of introducing an onsite nurse practitioner program. While the program cost $82,716, the organization realized $1.3 million in healthcare cost savings. Chenoweth and associates

					(2005) reported a benefit-to-cost ratio of 15:1 for healthcare costs and 2.4:1 in major diagnostic categories.
			Fraser (2018)	[46]	**Healthcare costs to health insurer and patient:** Using a sample of 9,503 patients, Spetz, Parente, Town, and Bazarko (2013) used costs paid by the health insurer and costs paid by patients in their CMA. Looking at 10 commonly treated conditions, they compared costs accrued over a 14-day period after receiving treatment from an APRN practicing independently, an APRN with a limited scope of practice, or a PCP. Using regression models, estimated mean cost of care was less with APRNs providing the treatment. Spetz and associates estimated a savings of $810 million if states allowed APRNs to practice independently.
			Fraser (2018)	[46]	The Perryman Group estimated Texas would see an annual impact of $24 billion in expenditures, $12 billion in gross product, and 122,735 permanent jobs by 2020; $34.8 billion in expenditures and $17.5 billion in gross product and 151,462 permanent jobs by 2030; and $46.9 billion in expenditures, $23.6 billion in gross product, and 177,220 permanent jobs by 2040, education costs were not included in the estimates
			Garner (2017)	[56]	**Costs (4 studies)**<br>Non sig. differences in 3/4 ; sig difference in ¼ studies related to initial hospital costs
			Jennings (2015)	[67]	**Cost of soft tissue injury management** was equal between medical, nurse practitioner and extended scope physiotherapist services (1/1)
			Kuethe (2013)	[29]	**Healthcare costs, direct and indirect Costs of outpatient visits** were lower in the nurse-led group (outpatient visits costs per patient per year; €156 in the nurse-led group versus €189 in the physician-led group; $P < 0.001$), not statistically significantly to lower total costs in the healthcare sector (total health costs €343 in nurse led group versus €357 in physician-led group; $P = 0.62$).
			Leduc (2021)	[30]	**Cost savings** assessed in 5 studies; Three studies found lower costs per patient in the intervention group while two studies noted higher costs in the intervention group. stat sig not indicated
			Lovink (2017)	[32]	**Costs**: sig. reduction in total costs in 1/1 study (Agvall 2013, 2014). The costs were EUR 6638 in the control group and EUR 4471 in the intervention group ($P = 0.01$)
			Lovink (2017)	[32]	**In LTC: Costs:** no significant difference in emergency department costs and hospital admission costs in 1/1 study

			Martin-Misener (2015)	[11]	**Alternative provider nurse practitioner role in ambulatory primary care (4 non-inferiority trials)**<br>Nurse practitioners in alternative provider primary care roles could function at least at the level of physician comparators, with equal or lower costs in 4/4 studies
			Martin-Misener (2015)	[11]	**Costs (2 studies)**<br>meta-analysis of the only two studies of this role that reported costs (2689 patients) with minimal heterogeneity and high-quality evidence, nurse practitioner care compared to general practitioner care resulted in lower mean health services costs per consultation (mean difference:<br>−€6.41; 95% CI −€9.28 to −€3.55; p<0.0001) (2006 euros). All patient/provider outcomes in these studies were equivalent or better for the nurse practitioner.
			Milesky (2020)	[47]	**Reduced healthcare costs** mentioned in 9 of 136 occurrences of facilitator themes, or 6.6%
			Morilla-Herrera (2016)	[34]	**Cost**<br>At 24 weeks after discharge, total Medicare reimbursements for health services were $1,238,928 in the control group vs $642,595 in the intervention group (P<.001)
			Swan (2015)	[12]	**Cost of care (4 studies)**<br>Three studies/4 estimated cost using provider salary; of these, two/3 found that APN care was less expensive compared with physician provided care.<br>One study/4 examined annual laboratory and monthly medication costs; while APN care was less expensive for laboratory services (64.9 ± 34.5 versus 91.5 ± 36.7 euros, P = 0.001), there were no differences in monthly medication costs. Spitzer et al., 1976, examined cost of care by developing a Utilization and Financial Index in which provider salary was aggregated with laboratory, radiology, hospital costs and out of pocket expenditures; no differences were observed between care provided by APNs and physicians.
			Tsiachristas (2015)	[41]	**Cost of ANPs (5 studies)**<br>2/5 showed reduced costs; 1/5 showed costs not sig. reduced; 2/5 showed that cost were increased

			Yang (2020)	[58]	**Wages (1 study)**<br>change in NP hourly earnings compared with physicians before and after the expansion of NP scope of prescribing practice between 2005 and 2010. The researchers indicated that expanding prescribing authority increased NP hourly earnings yet decreased physician earnings
			Yang (2020)	[58]	**Cost (price) of care (6 studies)**<br>Fewer restrictions of NP practice authority was associated with lower costs, more prescriptions filled in 5/6 studies
			McParland (2022)	[52]	**Costs (3 studies)**<br>Non-significant reductions in costs noted in 2/3 studies.<br>A significant reduction in health care costs in 1/3 studies (no p value reported)
			Sun (2022)	[53]	**Costs in home-based primary care (5 studies)**<br>Trends to cost savings noted in 2/5 studies with no p value reported. 1/5 studies reported significant reductions in mean costs per patients in the post intervention year, No significant reductions at 2 years or mean day cost differences in 1/5 studies.<br>One study reported on program costs.<br>Cost savings of $200,000 for 18 participants but researchers did not report how they calculated savings in 1 study.<br>Mean costs per patient in the postintervention year were significantly less in patients with high risk of hospitalization in the intervention arm than in those in the control arm ($5,088 vs. $6,575, p < .001) in 1 study.<br>**Total cost:** No differences noted in at 2 years or per intervention year in 1 study, mean day-cost differences in 1 study<br>Cost of NP intervention ($24,000 per 100 persons) and of preventing one day of stay in a nursing home ($35) estimated in one study published in 1995.
	**Emergency room visits**	14	Driscoll (2015) (Ansari paper)	[28]	**ER visits:** p = 0.81. (1 NP study)
			Elder (2015)	[63]	**Unexpected representations to ER**: Positive impact in one study (1/1)
			Fung (2014)	[55]	**Visits to the emergency room for post-surgical women with ovarian cancers**: increase in (1/1) but sig not specified

			HQO (2013)	[60]	**Emergency dept visits:**<br>Model 1: Fewer visits at 12 months than 6 months for the NP group; no differences at 12 months (1/1)
			HQO (2013)	[60]	**Visits**<br>Model 2 sig increase in visits (In one study: 6.1 vs 2.8, (p = 0.001)) (2/2)
			Ismail (2013)	[66]	**A&E attendance from older care home residents** (17%): Statistically significant reduction after controlling for seasonal variation.
			Jennings (2015)	[67]	**Unscheduled returns for emergency NP** (2 studies): Lower rate in 2/2<br>*Nash, 200*6: 2.3% compared with 4.2% for the medical patients (p < 0.001).<br>*Colligan, 2011:* 2% for the emergency NP group vs 1% (not sig.)
			Leduc (2021)	[30]	**ED Transports**<br>Reduction in transport to hospital found in 10/10 studies with stat sig in 4/10.
			Lovink (2017)	[32]	**Emergency Dept visits not leading to hospitalization**:<br>Sig reduction in the intervention group in 1/1 study (P = 0.001) (Agvall 2013, 2014)
			Lovink (2017)	[32]	**In LTC: Emergency Dept visit**: sig decrease in 1/2 studies, (p = 0.006), no sig differences in ½ studies
			Lovink (2017)	[32]	**Primary HC: Number of visits to the emergency department** : incidence rate ratio of 1.5 for the intervention group compared with the control group (P = 0.02) in 1/1
			Martin-Misener (2015)	[11]	**Number of patients who had at least one emergency department or urgent care visit**<br>Nurse practitioner and general practitioner care were equivalent. Non sig.
			Newhouse/ Stanik-Hutt (2013)	[35/36]	**Number of unexpected Emergency department (ED) or urgent care visits** (5 studies); 1/5 studies reported results favouring NP group and 4/5 reported no difference between groups (no p-values reported).
			Osakwe (2020)	[37]	**NP-home visits on ED visits**: sig 2/2. Significant reductions in the ED visits by 35.56% and 23.7% after implementation of the home based primary care (HBPC) program after with 6 months (p = 0.001) and 12 months (p = 0.001) and home care patients who received NP-home visits had less ED visits at 2 weeks (p = 0.0005) and 4 weeks (p = 0.0055) compared to those receiving usual care. No significant difference in the number of ED visits between the 2 groups at the 8 week period (p = 0.800).

			Van Vliet (2020)	[42]	**Non-conveyance (ambulance transport) (n=3 studies)**<br>non-conveyance rates ranging from 20% –50% for PAs. Non-conveyance rates for the NP were not described.
			Jeyaraman (2022)	[45]	**Number of ED visits with NP led triage (3 studies)**<br>Trends towards a decrease in ED visits were noted in 2/3 studies (no p value reported).<br>The number of patients visiting ED increased by 51 visits per month compared to the traditional nurse-led triage mode in 1/3 studies. (no p value reported)<br>A 5% decrease in ED visits in the NP team triage group noted in 1/3 studies (no p value reported). The number of ED visits preintervention dropped from 2194 ED visits over 6 weeks to 1699 patient visits over one month during the postintervention period in 1/3 studies. (p value not reported).
			Sun (2022)	[53]	**Emergency department visits (8 studies)**<br>5/8 reported significantly less ED visits among intervention participants; no group difference in 3/8 studies
	**Healthcare Service Delivery**	17	Ansell (2017)	[65]	**No Show rates**: 4 studies. Unchanged ¼; reduction ranging from 1.44% to 5% in ¾ studies). No p-values reported.
			Carranza (2021)	[59]	**Continuity of care**: reported in 1 study. NPs rated higher. Same Provider (62.1% vs.36.8%); Patient inclusion in plan of care) (79.3% vs.47.4%). No p-values reported.
			Donald (2015)	[27]	**Additional interventions** (RR: 1.02, 95%CI: 0.66–1.56, p = 0.93)
			Fung (2014)	[55]	**Feasibility of transitional care model for patients with schizophrenia**, not sig. (1/1)
			Fung (2014)	[55]	**Home-based intervention for individuals with SMI/HIV,** a significant improvement in depression (P = 0.012) and in the physical component of health-related quality of life (QOL) (P = 0.03) from baseline to 12 months.
			Fung (2014)	[55]	**fewer primary care visits** ($\beta = -0.95 \pm 0.16$, $P = 0.0003$) for post-surgical women with ovarian cancers
			Garner (2017)	[56]	**Acceptability of nurse-led care** assessed in 4 studies. Acceptability of nurse-led care superior in ¾ studies and unchanged in ¼ studies
			HQO (2013)	[60]	**Specialist visits:**<br>Model 1: more specialty visits at 12 months compared with 6 months in both groups;

					no differences at 12 months 1/1<br>Model 2: (no data)
			HQO (2013)	[60]	**Primary care visits**<br>Model 1: mean number of visits 3.1 SD = 2.38, no sig differences between groups<br>Model 2: (no data)
			Jennings (2015)	[67]	**Collaborative model of care:** (1/1 study) increased patient throughput with larger numbers of patient presentations being seen
			Leduc (2021)	[30]	**End of life care** in 3 different ways between 5 studies<br>One study implemented a palliative care framework and set of tools in addition to a palliative care nurse providing and modeling good palliative care. They report a 7% reduction in hospital admission in the last eight weeks of life.(Hockley, et al., 2010)<br><br>One author contributed three retrospective cohort studies to this review, one of which was a subgroup analysis of patients with moderate to severe advanced dementia. (Miller et al., 2017) These studies evaluate palliative care consults provided by nurse practitioners that address goals of care and symptom management. All three studies showed a reduction in hospital admission in patients with palliative care consults, and both studies measuring ED visits found a reduction in these as well. Patients who had earlier consults had a hospital admission rate that was 13.2% less than those without consults (p=.003).
			Lovink (2017)	[32]	**Number of outpatient contacts:** no sig differences.<br>Difference is significant once outpatients contacts and primary care contacts are combined: 16.3 per patient in the intervention<br>group vs. 24.3 per patient in the control group (P = 0.04)
			Lovink (2017)	[32]	**Number of primary healthcare contacts**: no sig differences.<br>Difference is significant once outpatients contacts and primary care contacts are combined: 16.3 per patient in the intervention<br>group vs. 24.3 per patient in the control group (P = 0.04)
			Lovink (2017)	[32]	**Process evaluation:** no study was identified where implementation was an outcome measure in its own right

			Martin-Misener (2015)	[11]	**Return visits: (3studies)**<br>nurse practitioners were more likely to ask patients to return than the general practitioners (2562 patients; I2=76% (RR 1.32, 95% CI 1.20 to 1.46); p<0.0001).<br>The number of patients who made return visits within 2 weeks for the index reason was reported in three trials. A meta-analysis, including almost 3500 patients (I2=5%), indicated that more nurse practitioner patients than general practitioner patients made return visits for the same problem or within 2 weeks (RR 1.18; 95% CI 1.06 to 1.32; p=0.002) (HQE). One study examined the number of return visits for any reason over 1 year and found equivalent results (MQE).
			Morilla-Herrera (2016)	[34]	**Service use**<br>1/2 studies At 180-days of follow-up, significant differences in the minutes/month spent by the community nurse between groups [34.5(102.0) vs 96.1 (352.2); P: 0.05]; 1/2 studies the intervention had no effect on overall service use rates at 30 or 120 days
			Osakwe (2020)	[37]	**Transition care and case management**: half as many ED visits compared with the usual care group (mean=0.50, SD=1.2 versus mean =0.99, SD=2.5; P = 0.096) (1 study).
			Swan (2015)	[12]	**Healthcare resource utilization (4 studies)**<br>Consultation length: Four studies [27, 29, 31, 33] examined consultation length; of these, three found that APN consultations were 3.0 [29] to 4.3 [33] minutes longer than those provided by physicians [29, 31, 33].<br><br>Two RCTs and one follow-up study examined total number of primary care visits with conflicting findings at 1 year [27, 32] but fewer visits among APN patients at 2 years [34].<br><br>One RCT [32] and its follow-up study [34] examined hospitalization and emergency department or urgent care visits with no significant differences between groups.
			Tsiachristas (2015)	[41]	**Health care utilization**: Three studies<br>2/3 found no differences and one/3 study found an increase.
			Van Vliet (2020)	[42]	**Follow-up contacts (n= 1 study)**<br>Follow-up contact after the completion of prehospital EMS care also indicated no significant differences between PAs and nurses

			Van Vliet (2020)	[42]	**Resource use** (n=1 study)<br>One study found in 107 cases other EMS resources were released from the scene and put back in service while the NP attended the patient, (by default, two units respond to a call). Eighteen high utilizers of 911 were connected with a social work organization, and 12 of 18 (66.7%) decreased their use of EMS in the 90-days following.
			Yang (2020)	[58]	**NP growth over time (3 studies)**<br>1 study reported that restricted practice regulations reduced NP growth rates by 25% over a 7 year period.<br>1 study showed no sig difference in practice authority and growth in rural communities over a 4-year period.<br>1 study showed that NP growth occurred across most states but that growth was significantly higher in states with reduced practice authority (prescribing)
			Yang (2020)	[58]	**Overall health service utilization (n = 11)**<br>10/11 studies reported higher services utilization under Full Practice Authority<br>**Primary care utilization** included number of NP visits in primary care, routine check ups, rates of cancer screening, chronic disease management and preventable hospitalization, education, counselling, and medication-related visits, reduced use of the emergency, increase in number of psychotropic and opioid prescriptions with no decrease in mental health outcomes, increase in number of opioid misuse treatment admissions.<br>No sig difference in State -level Opioid and benzodiazepine prescription rates with full prescriptive authority.

			Zhang (2020)	[43]	**Perceived barriers of pre-exposure prophylaxis implementation (26 studies) among health providers**<br>the lack of such a request from patients (56, 95% CI = 48–64%),<br>concerns about toxicity and resistance (55, 95% CI = 34, 74%),<br>lack of knowledge about PrEP (47, 95% CI = 27, 66%),<br>concern about the cost to patients (46, 95% CI = 31, 62%),<br>lack of clear guidance (41, 95% CI = 36, 47%),<br>concerns about patients' adherence (38, 95% CI = 26, 50%),<br>concerns about no supporting evidence (33, 95% CI = 9, 63%),<br>time management (29, 95% CI = 13, 49%),<br>risk compensation (28, 95% CI = 17, 40%),<br>lack of comfort regarding PrEP (24, 95% CI = 5, 50%) (Fig. 2).
			Zhang (2020)	[43]	**Ideal location to provide pre-exposure prophylaxis (26 studies)**<br>Health providers reported that the ideal locations for PrEP care included HIV clinics (p = 71, 95% CI = 67, 74%), primary care settings (p = 42, 95% CI = 27, 58%), sexually transmitted disease clinics (p = 40, 95% CI = 8, 78%), and public health departments (p = 21, 95% CI = 16, 27%).
	**Hospitalization**	15	Barker (2018)	[10]	**Unplanned hospital transfer**: reduction 10/12; 7/12 reached statistical significance.
			Carranza (2021)	[59]	**Hospital admission rates** for patients with lung disease: sig. higher in NP group (1/1) 66 NP vs. 42 MD (relative rate of 1.52, p = .03); -43 vs. 23 bronchiectasis-related (relative rate 1.59, p = .22)
			Donald (2015)	[27]	**Re-hospitalisations** (RR: 0.60, 95%CI: 0.21–1.73, p = 0.34)
			Donald (2015)	[27]	**Index re-hospitalisation up to 42 days**: Meta-analyses (n = 766, pooled relative risk (RR): 0.69, 95%CI: 0.34–1.43, I2 = 0%). Not significant.
			Donald (2015)	[27]	**Any re-hospitalisation up to 180 days**: **Meta-analysis:** (n = 800, pooled RR: 0.87, 95%CI: 0.69–1.09, I2 = 32%) were inconclusive (low quality). Not significant.
			Donald (2015)	[27]	**Index re-hospitalisation over 90 days** (RR: 0.55, 95%CI: 0.32–0.94, p = 0.03) in complex care patients (both low quality): significant reduction for complementary provider nurse practitioners.

			Donald (2015)	[27]	**Index re-hospitalisation over 180 days** (RR: 0.62, 95%CI: 0.40–0.95, p = 0.03) in complex care patients (both low quality): significant reduction for complementary provider nurse practitioners.
			Driscoll (2015) (Ansari paper)	[28]	**Hospitalizations**: p = 0.81. (1 NP study)
			Driscoll (2015) (Ansari paper)	[28]	**Hospitalizations for CHF**: p = 0.66 (1 NP study)
			Driscoll (2015) (Ansari paper)	[28]	**Median hospitalizations or ER visits per patient:** p = 0.14 (1 NP study)
			HQO (2013)	[60]	**Hospitalization**<br>Model 1: no sig differences in patients hospitalized at 6 months (2/2) and 12 months (1/1)<br>Model 2: stat sig difference in proportion of patients hospitalized after receiving nurse-led secondary prevention (1/1)
			Kuethe (2013)	[29]	**Hospital admissions** (4 studies) pooled data showed substantial heterogeneity (RD -0.02; 95% CI -0.06 to 0.02; I2= 59%). For patients with stable asthma, no sig difference between groups
			Leduc (2021)	[30]	**Hospital Admission**<br>Reduction in 15/16 studies, effect ranged from 3% to almost 50% reduction with Evercare. Stat sig. in 8/16 studies
			Lovink (2017)	[32]	**Hospital admissions:** sig decrease in the intervention group (p = 0.03) (Agvall 2013, 2014) (1/1)
			Lovink (2017)	[32]	**Hospital days**: no sig differences (Agvall 2013, 2014) (1/1)
			Lovink (2017)	[32]	**In LTC: Hospital admissions:** 2/4 studies found sig reductions (p = 0.03 and p = 0.001); no sig differences in 2/4 studies

			Lovink (2017)	[32]	In LTC: **Number of hospital days**: sig. decrease in 1/1 study, from 4170 per 1000 patient years in the control group to 1310 per 1000 patient years in the intervention (P < 0.001).
			Lovink (2017)	[32]	**Primary HC: Number of hospital admissions**: no significant difference in 1/1 study (Everett 2013a,b)
			Martin-Misener (2015)	[11]	**Number of patients who were hospitalized at least once** Nurse practitioner and general practitioner care were equivalent. Non sig.
			Milesky (2020)	[47]	**Decreased Hospitalization** identified in 27.2 % of theme occurrences
			Morilla-Herrera (2016)	[34]	**Hospital admission (readmission) 4 studies** 2/4 studies indicated sig differences (Number of hospitalizations reduced at 3 month of follow-up [IG:47 (23%) vs CG:68(33%); p = 0.003 (Imhof); By week 24 after the index hospital discharge, control group patients were more likely than intervention group patients to be readmitted at least once (37.1 % vs 20.3 %; P<.001) (Naylor 1999)) and 1/4 studies showed no significant difference, 1/4 reported a reduction (no p-value reported)
			Morilla-Herrera (2016)	[34]	**Patient Institutionalization (residential care)** 3/5 studies indicated significant fewer transfers to residential care; 1/5 no difference; 1/5 trend in favour of intervention (no p-value reported)
			Newhouse/ Stanik-Hutt (2013)	[35/36]	**Hospitalization:** Seven studies. 3/7 studies reported findings favouring the NP intervention and 4/7 reported no differences between groups (no p-values reported)
			Osakwe (2020)	[37]	**NP-home visits on hospitalizations**: not sig. in 2/2 studies. The mean length of stay per hospitalization was 6.3 days in the intervention group and 5.1 days in the control group (p = 0.7) and p value in study 2: (p = 0.514).
			Osakwe (2020)	[37]	**NP home-visits on readmission**: 4 studies. 2/4 studies reported significant decreases in hospital readmissions: for patient following cardiac surgery with the addition of home visits (p = 0.023) in the study by Hall et al.; Coppa et al., also found a 59.42% decrease in readmissions at 6 months (p = 0.001) after enrollment in the home based primary care intervention led by a NP, however the result was not sustained at the 12 month-interval (p = 0.087). Not sig findings in 2/4 studies.

			Smigorowsky (2020)	[57]	**Effect of NP-led care on 30-day readmission rates for HF (2 studies)**<br>The meta-analysis using a model of random effects revealed NP-led care had no statistically difference (Risk Ratio: 0.74, 95% CI: 0.47, 1.17, Z = 1.27, p = .20) on 30-day readmission rates in HF. I2 statistic is 15% and indicates that at the risk of heterogeneity is low
			Sun (2022)	[53]	**Hospitalization (12 studies)**<br>10 /12 studies found that NP home visits led to significantly fewer hospitalizations.<br>A trend of decreased hospitalizations noted in 2/12 studies with non-significant results.
			Sun (2022)	[53]	**Nursing home admissions (3 studies)**<br>Fewer nursing home admissions in intervention groups in 3/3 studies. P values not all reported.
	**Length of stay (LOS)**	7	Elder (2015)	[63]	**Nurse-initiated X-Rays** showed little impact on ED LOS in two studies/ 3.
			Elder (2015)	[63]	**ED LOS**: 1/1 indicated that ED LOS was not impacted by nurse-initiated analgesia.
			Galiana-Camacho (2018)	[64]	**Average length of stay per patient** of 180 minutes, together with 78.5% of patients seen in less than 4 hours.
			HQO (2013)	[60]	**Length of visits**<br>Sig. Increase of 11 minutes in average time in one study and (MD 95 minutes in another study) (p < 0.001) (2/2)
			HQO (2013)	[60]	**Length of stay:**<br>Model 2: no sig. difference in median LOS at 1 year (6 days, p= 0.49) (1/1)
			Leduc (2021)	[30]	**Length of stay:** decrease in 4/4 studies, ranging from 0.2 days fewer (43) to 1.2 days fewer in the intervention group (32). Stat sig not indicated
			Morilla-Herrera (2016)	[34]	**Length of Stay**<br>2/2 studies showed a significant decrease in length of stay in the intervention group (p < 0.002; Huang and Liang); at 24 weeks, mean LoS for readmitted patients in the control group (n=69) was higher than in the intervention group (n=36), (11.0 +/- 10.6 days vs. 7.5 +/- 4.8 days; p<0.001) (Naylor 1999).

			Newhouse/ Stanik-Hutt (2013)	[35/36]	**Hospital Length of stay (LOS)** Two studies. A moderate strength of evidence indicated that care involving NPs was similar to care involving only MDs in terms of hospital LOS (2/2 studies; no differences between groups, no p-values reported)
			Jeyaraman (2022)	[45]	**Emergency Department length of stay (LOS) (9 studies)**<br>All nine studies in the NP team triage model showed a decrease (median = -28.50 minutes) in ED LOS favoring the intervention group. 5/9 studies showed significant decrease in LOS and 4/9 indicated a decrease without reaching statistical significance.
			Jeyaraman (2022)	[45]	**Patient discharge from ED within benchmark times (4 studies)**<br>At 60 minutes, 41% of patients discharged from the ED in the NP team triage vs 16% in the traditional nurse-led triage group (1/1 study)<br>At 90 minutes, 30% of low-acuity patients in the NP team triage group discharged vs 12% in the traditional nurse-led triage group. (1/1 study)<br>At 4 hours, 98.1% of patients discharged under 4 hours in the in the NP team triage group compared to 94.7% in the traditional nurse-led triage group. (1/1 study)<br>Under 6 hours, 85.7% of patients discharged in the NP team triage group vs 80.1% in the traditional nurse-led triage group. (1/1 study)
			Jeyaraman (2022)	[45]	**Time to triage (2 studies)**<br>Statistically significant decrease (pre-intervention time to triage (Median: 4; IQR: (2, 10)); post-intervention time to triage (Median: 3; IQR: (1, 8)) favoring the intervention group noted in ½ studies.<br>98% of patients in the intervention group were triaged within 15 minutes vs 75% of patients in the comparison group. (p value not reported)
	**Patient safety**	11	Carranza (2021)	[59]	**Treatment complications**: 4/4 no sig difference between NP and physician groups
			Carranza (2021)	[59]	**Adverse effects 4/4 studies**<br>no sig difference between NP and physician groups
			Donald (2015)	[27]	**Falls:** estimates favoured nurse practitioner care
			Driscoll (2015) (Ansari paper)	[28]	**Adverse events**: (1 NP study) There were no differences in adverse events among groups. (1/1)

			Galiana-Camacho (2018)	[64]	**Patient safety:**<br>**Medication and medical history:** completed in 97.4% of patients encounters (1/1).
			Galiana-Camacho (2018)	[64]	**Patient safety:**<br>**Medication interaction:** documented in 87.5% of cases (1/1).
			Galiana-Camacho (2018)	[64]	**Patient safety: Sexual health:** documented in 65.9% of cases (1/1).
			Galiana-Camacho (2018)	[64]	**Patient safety:** no unplanned readmissions by the Transitional Emergency Nurse Practitioner (TENP) were adequate (1/1).
			Galiana-Camacho (2018)	[64]	**Patient safety:** all diagnostic tests requested by the Transitional Emergency Nurse Practitioner (TENP) were adequate (1/1).
			Garner (2017)	[56]	**Safety:**<br>**Adherence to lab tests:** no stat. sig. difference between nurse-led care and rheumatologist-led care in 4/4 studies for: mandatory monitoring of laboratories for patients; out-of-range blood tests;.<br>**Hospitalizations**: the number of hospitalizations 5/5 no sig. difference in nurse-led care;<br>**number of unplanned family physician visits**: no diff. at 12 months and 24 months (1/1)
			Garner (2017)	[56]	**Safety:**<br>**Adherence to lab tests:** no stat. sig. difference between nurse-led care and rheumatologist-led care in 4/4 studies for: mandatory monitoring of laboratories for patients; out-of-range blood tests;.<br>**Hospitalizations**: the number of hospitalizations 5/5 no sig. difference in nurse-led care;<br>**number of unplanned family physician visits**: no diff. at 12 months and 24 months (1/1)

			Garner (2017)	[56]	**Safety:**<br>**Adherence to lab tests:** no stat. sig. difference between nurse-led care and rheumatologist-led care in 4/4 studies for: mandatory monitoring of laboratories for patients; out-of-range blood tests;.<br>**Hospitalizations**: the number of hospitalizations 5/5 no sig. difference in nurse-led care;<br>**number of unplanned family physician visits**: no diff. at 12 months and 24 months (1/1)
			HQO (2013)	[60]	**Risk factor management**<br>Model 2: CAD patients receiving care from specialized nurses were 5 times more likely to achieve appropriate blood pressure (P < 0.001) management and 3 times more likely to have appropriate lipid management (P < 0.001) (1/1)
			Jennings (2015)	[67]	**Missed injuries and inappropriate management:** no sig difference (1/1)
			Loescher (2018)	[31]	**Decreased the number of unnecessary biopsies in 2/2 studies** (Ali et al., 2014; Armstrong, 2011).
			Morilla-Herrera (2016)	[34]	**Falls rate**<br>1/2 studies showed significant reduction in falls (p = 0.003); ½ studies found no significant differences in the number of falls
			Yang (2020)	[58]	**Malpractice (1 study)**<br>Between 1999 and 2012: 31% lower malpractice payments per 1,000 physicians in states with FPA compared with those with restricted practice authority
			McParland (2022)	[52]	**Falls (1 study)**<br>Case-finding for referral to other services did not reduce falls risk. (p value not reported)
	**Quality of care**	6	Jennings (2015)	[67]	**Quality of care** (4/4) Emergency NP patient group rated their care as excellent compared with the medical care patient group (68% vs. 50%, Fisher's exact p < 0.02) (1/1)
			Kuethe (2013)	[29]	**Quality of care**<br>No sig difference in 2/3 studies (Kamps; van Son)<br>Asthma-specific and child-specific quality the ratings for the paediatrician-led care and

					nurse-led were higher than for the general practitioner-led care (P < 0.05). (Kuethe, 2011)
			Lovink (2017)	[32]	**Assessing Care of Vulnerable Elders-3 (ACOVE-3) quality indicators** Higher score in favour of the intervention, 54% compared with 34% in the control group (P < 0.001).
			Milesky (2020)	[47]	NPs **improved healthcare quality** in 19.1% of theme occurences
			Tsiachristas (2015)	[41]	**Quality of care: 1 study** reported improvements in Quality of care
			Yang (2020)	[58]	**Care quality**: 3/3 reported no sig differences in overall quality of care by state level of NP practice regulations
	**Scope of practice**	6	Elder (2015)	[63]	**Nurse-Initiated analgesia** was improved with the introduction of nurse-initiated analgesia protocols in three studies (3/3).
			Galiana-Camacho (2018)	[64]	**Degree of autonomy for APNs** at work ranging from a 27% to 84% of patients treated autonomously (3/3)
			Hyer (2019)	[69]	**NP Practice**: NPs identify and assess weight status during an office visit in 7 studies out of 15, and they intervene or counsel patients regarding obesity in 5 studies out of 15
			Hyer (2019)	[69]	**Provider perceived responsibility** for managing obesity: 3/3 studies showed the positive impact (Asselin et al., 2016; Nolan et al., 2012; Petrin et al., 2017).
			Milesky (2020)	[47]	**Unrestrictive or least restrictive scope of practice for NPs** was mentioned in 10 of 136 occurrences of facilitator themes, 7.35%
			Patel (2019)	[38]	**NP SOP and characteristics of the health delivery system:** 8 studies more growth in the number of NPs in states with the least restrictive SOP policies. Patients in states with the least restrictive NP SOP policies were more likely to have an NP as their PC provider (Kuo et al., 2013). The results of most studies showed a positive association between less restrictive NP SOP policy and NP workforce capacity.

					1/8 study reported no significant association between NP SOP policy and number of NPs licensed to practice per 100,000 population.
			Patel (2019)	[38]	**NP SOP and characteristics of the population-at-risk**: 5 studies.<br>4/5 studies reported that NPs with less restrictive Scope of practice were more likely to work in PC, provide care in rural and high-poverty areas.<br>Accept patients under Medicaid; sig difference in 2/5.<br>One study found no sig difference
			Patel (2019)	[38]	**NP SOP and utilization of health services**: 4 studies<br>greater use of preventive services and decreased rates of avoidable hospitalizations, hospital readmissions within 30 days discharge from rehabilitation, and hospitalizations of nursing home patients in states with the least restrictive NP SOP policies in ¾ studies<br>One out of the four studies reported an increased likelihood of patients receiving a referral to a physician from an NP at Community Health<br>Centers in states with SOP policies that allow NPs to practice without physician supervision.<br>¼ studies reported that a larger supply of NPs, without considering other state- and patient-level factors, did not significantly affect healthcare utilization.
			Yang (2020)	[58]	**NP Supply. (6 studies)**<br>5/6 studies showed a positive association between FPA and NP supply. 1/6 (nursing home study), reported an insignificant relationship (Intrator et al., 2015).
			Yang (2020)	[58]	**NP Workforce distribution. (4 studies)**<br>1/3 studies indicate higher odds of NPs in rural areas in states with FPA, compared with those with restricted regulations, though the difference was not statistically significant<br>2/3 studies found significantly greater numbers of NPs in rural areas of states with FPA than those with restricted practice regulations<br>1/1 study evaluating the odds of NPs practicing primary versus specialty care by level of state NP practice regulations and found that NPs had 13% higher odds of practicing primary care in states with FPA than in states without it

			Yang (2020)	[58]	**NP autonomy (2 studies)**<br>NPs had greater day-to-day practice autonomy when they had full independent prescriptive authority, yet having practice independence for diagnosis and treatment only showed little effect on autonomy.<br>2/2 studies: no difference in NP hospital admission privileges by level of state NP practice regulations and hospital admission privileges did not differ by prescriptive or practice independence
			Yang (2020)	[58]	**Mobility (1 study)**<br>odds of moving from one state to another by state NP practice regulations between 1992 and 2004 and found that NPs were 46% more likely to move from a state with no controlled substance prescriptive authority to one that allows NPs to prescribe them.
			Yang (2020)	[58]	**NP-provided health service use (5 studies)**<br>**Full practice authority** led to sig increases in care provided by NPs in 4/5 studies. Neg association in 1/5 studies (services provided in LTC)
	**Wait time**	7	Ansell (2017)	[65]	**Wait times for appointments: reduced wait times in** 11/11 studies. Mean reduction of -11.3 days (SD +/- 8.3 days) for all included studies after implementation.
			Ansell (2017)	[65]	**Open access scheduling**: Reduced wait times in 11/11 studies. No p-values reported.
			Ansell (2017)	[65]	**Wait times for appointments:** Use of NPs reduced wait times for appointments in 2/2 studies. No p-values reported.
			Ansell (2017)	[65]	**Wait times for appointments**: Telephone follow-up consultations reduced wait times for appointments in 2/2 studies. No p-values reported.
			Ansell (2017)	[65]	**Wait times for appointments**: Measures to promote self care reduced wait times for appointments in 2/2 studies. No p-values reported.
			Ansell (2017)	[65]	**Wait times for appointments:** Email consultations reduced wait times for appointments in 2/2 studies. No p-values reported.
			Carranza (2021)	[59]	**Wait times:** reported in 2 studies. Decreased wait time from 46 to 42 days for non-urgent appointments in pediatric NP clinic; no p-values reported (2/2).
			Elder (2015)	[63]	**Did-Not-Wait rate** was reported in one study with a 1% reduction in DNW rates over a 12 month period following the introduction of the clinical initiative nurses (CIN) role ($P < 0.001$). (1/1)

			Elder (2015)	[63]	**Nurse-initiated X-Rays** showed little impact on wait times in two studies/ 3.
			Elder (2015)	[63]	**Wait times:** Reduced in 5/6 studies
			Jennings (2015)	[67]	**Wait time:** Reduction the number of patients' who did not wait for treatment during service delivered by the nurse practitioner service (1/1)
			Jeyaraman (2022)	[45]	**Provider initial assessment (9 studies)**<br>Significant reductions in mean difference in time to provider initial assessment in 8/9 studies (-3.00 min, 95%CI [-3.47, - 2.43] to -50 min, 95%CI [- 53,63, -46.37]<br>Non-significant trend towards a reduction noted in 1/9 studies (-2.30 min [-5.93, 1.33].
			Jeyaraman (2022)	[45]	**Leave without being seen (LWBS) (6 studies)**<br>5/6 studies reported a reduction in percentage of patients who LWBS in the NP team triage. 4/6 studies reported statistically significant decrease in the intervention group; 1/6 studies did not report p value). (p value not reported). 1/6 studies reported a non significant change in patients LWBS.
			Jeyaraman (2022)	[45]	**Leave against medical advice (LAMA) (3 studies)**<br>Non-significant findings reported in 3/3 studies.

			Jennings (2015)	[67]	**Wait times:** 8 studies.<br>**Significant reduction in 2/8 studies favors NP:** Colligan et al. 2011: significant reduction in waiting time for patients managed by the emergency nurse practitioners in comparison to the emergency department medical registrars. Time to be seen (median) NP group 14 min (range 5–27) vs. 50 min (range 21–78) in doctor group (p < 0.0001).<br>Jennings et al. 2008: Significant reduction in waiting times for NP managed patients. Waiting times NP was 12 min (range 5.5–28 min), vs. 31 min (range 11.5–76 min), (p < 0.001) for doctor group.<br><br>**Reduction favors NP in 3/8, p value not reported or no significant difference in 3 studies**<br>*Dinh et al 2012* reported that patients managed by emergency nurse practitioners trended to shorter waiting times when compared with medical officers, with a difference of 7 min (p = 0.06).<br>*Fry et al. 2011*: Median transitional emergency nurse practitioner waiting time 38 min compared with 59.7 min previous year. Did not wait 4.5% vs. 8.1% in previous year.<br>*Considine et al. 2010*: waiting time reduction.<br><br>**Wait times: No significant difference in 3/8 studies**<br>*Van der Linden et al. 2010:* No significant difference in waiting time 19 mins nurse practitioners vs. 20 mins doctors<br>*Steiner et al. 2009:* No significant differences in overall median waiting times. Did not wait rate 11.9% vs. 13.7% (Intervention/Control).<br>*Considine et al. 2006:* No significant differences in median waiting time between nurse practitioner candidate and doctor managed patients.
			Loescher (2018)	[31]	**Numbers of patients seen in a timely manner:** increase (no p value reported)
			Van Vliet (2020)	[42]	**On-scene time (n=2 studies)**<br>One/2 studies reported no significant difference between PAs and nurses regarding the length of on-scene treatment time.<br>½ study: an average length of treatment time on scene of 21.47min but made no comparison with other EMS professionals.
